# Backbone Engineering of Carbon‐Centered NHC‐Derived Diradicals: From Electronic State Tuning to High‐Performance Organic Field‐Effect Transistors

**DOI:** 10.1002/adma.73264

**Published:** 2026-05-05

**Authors:** Xiao‐Xu Liu, Lianghui Li, Xin Li, Can Chen, Man Li, Shun Tian, Paul J. Dyson, Ying‐Feng Han

**Affiliations:** ^1^ College of Chemistry and Materials Science Northwest University Xi'an P. R. China; ^2^ Institut des Sciences et Ingénierie Chimiques École Polytechnique Fédérale de Lausanne (EPFL) Lausanne Switzerland; ^3^ College of Chemistry Zhengzhou University Zhengzhou P. R. China

**Keywords:** carbon‐centered diradical, diradical character, N‐heterocyclic carbene, open‐shell organic semiconductor, organic field‐effect transistor

## Abstract

Diradicals have gained interest for their unique electronic properties and potential applications in organic electronics and semiconductors. However, precise manipulation of electronic states and achieving satisfactory device performance remain challenging. Herein, we report the synthesis and characterization of tetraphenylethylene‐bridged salts and their neutral diradical counterparts obtained via two‐electron reduction. With extended conjugation and electron‐withdrawing N‐heterocyclic carbene (NHC) backbones, enhanced open‐shell character is observed. Through this systematic study, a backbone engineering strategy is established that allows precise control over spin states and diradical character in carbon‐centered NHC diradicals. Leveraging this strategy, compound **2d**, with moderate diradical character induced by extended conjugated structures and strong electron‐withdrawing groups, was employed in a spin‐coated organic field‐effect transistor (OFET) device. The device achieved a record‐high hole mobility of 4.53 cm^2^·V^−1^·s^−1^, representing exceptional performance among open‐shell organic semiconductors for high‐performance OFETs. This not only demonstrates the outstanding charge‐transport capability of **2d** but also underscores the significant potential of this approach for developing functional open‐shell organic semiconductors.

## Graphical abstract

The open‐shell nature of diradicals facilitates charge separation, and compounds with moderate diradical character demonstrate remarkable potential in organic field‐effect transistors (OFET) applications due to their low reorganization energies for hole and electron transport. This study has established a backbone engineering strategy that enables precise control over the spin states and diradical character in carbon‐centered N‐heterocyclic carbene systems. The resulting device using a structure with moderate diradical character achieved a hole mobility of 4.53 cm^2^·V^−1^·s^−1^, setting a new record for open‐shell organic semiconductors in high‐performance OFETs. This work highlights the outstanding charge transport capability of open‐shell diradical systems and offers an effective strategy toward high‐performance functional organic semiconductors.

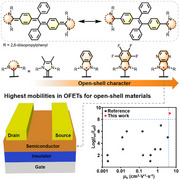

## 1 Introduction

Diradicals have gained widespread attention due to their unique electronic structures and physicochemical properties [1, 2, 3, 4, 5], demonstrating promising applications in luminescent materials [6, 7, 8, 9, 10], spintronics [11, 12, 13, 14, 15], photothermal conversion [16, 17, 18, 19], semiconductors [20, 21, 22, 23, 24], and bioimaging [25, 26, 27, 28]. Understanding the spin characteristics of unpaired electrons in diradicals is crucial for designing radical‐based materials with precisely tuned properties.

**FIGURE 1:**
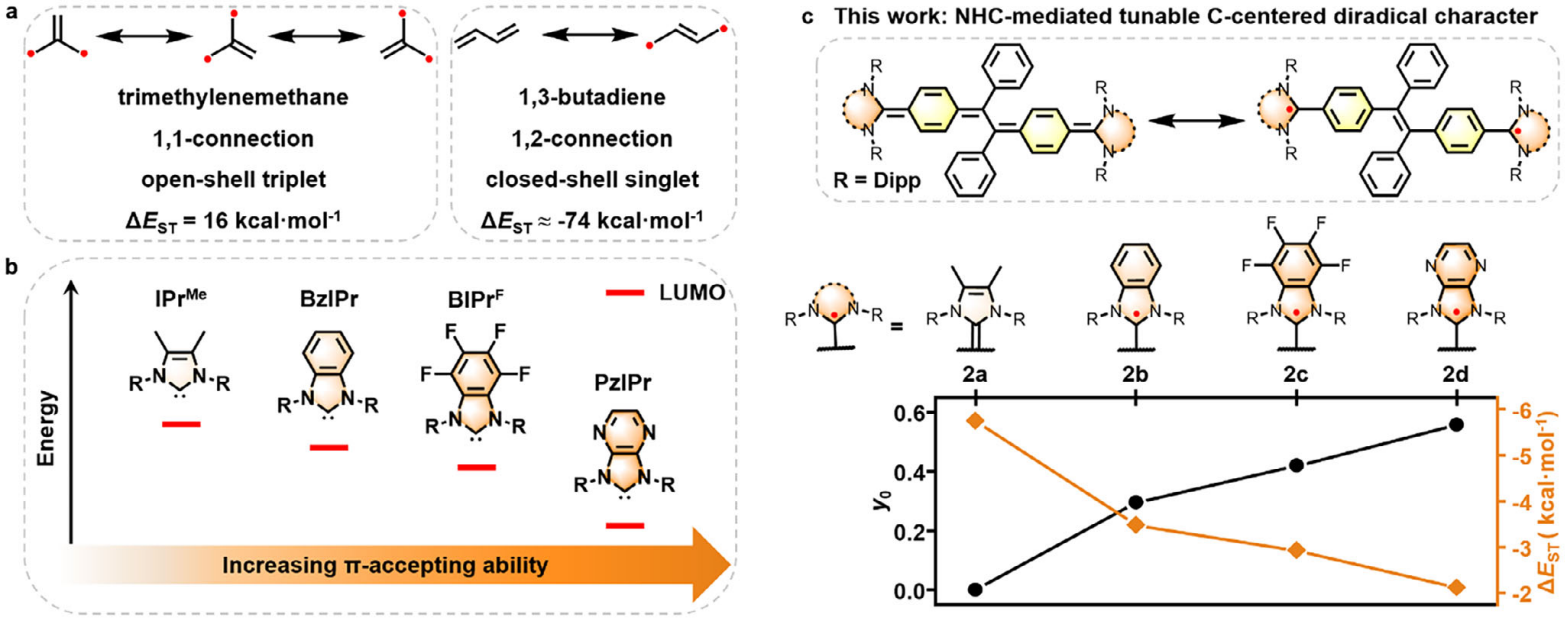
(a) TMM and 1,3‐butadiene diradicals. (b) The lowest unoccupied molecular orbital (LUMO) of NHCs. (c) This work: NHC‐mediated tunable‐C‐centered diradical character.

Trimethylenemethane (TMM) and 1,3‐butadiene are classic examples of carbon‐centered diradicals connected via ethylene units, which are formed from 1,1‐ and 1,2‐connections [29, 30]. The 1,1‐connection generates the non‐Kekulé TMM diradical with a triplet ground state. In contrast, the 1,2‐linkage affords a Kekulé‐type 1,3‐butadiene diradical typically characterized as a closed‐shell singlet ground state with a large singlet‐triplet energy gap (Δ*E*_ST_), restricting the development of analogous diradicals (Figure 1a). Modulation of the butadiene‐based structure leads to a transition from a closed‐shell to a diradical, enabling the fabrication of organic electronic materials with tunable effects. When tetraphenylethylene (TPE) was employed as a central linker to assemble Blatter diradicals, the high electronegativity of nitrogen enabled the formation of nitrogen‐centered 1,3‐butadiene‐type diradicals with an open‐shell singlet ground state [31]. However, the low electronegativity of carbon destabilizes unpaired electrons, significantly hindering the synthesis of carbon‐centered 1,3‐butadiene‐type diradicals with open‐shell ground states.

N‐Heterocyclic carbene (NHC)‐based radicals exhibit higher thermodynamic stability than traditional ones due to the stabilizing effect of the empty *p*‐orbital [32, 33, 34, 35]. Introducing bulky groups such as 2,6‐diisopropylphenyl (Dipp) on the N atoms can physically shield the radical center, resulting in excellent kinetic stability [36, 37, 38]. Additionally, delocalization of the unpaired electrons within the heterocyclic backbone enhances radical stability, which becomes more pronounced with extended conjugation and the introduction of electron‐withdrawing groups. Compared to other carbon‐centered radicals, the unique electronic structure and steric effects of NHCs contribute to superior stability.

Traditional strategies for modulating the ground‐state spin and physicochemical properties of carbon‐centered diradicals primarily rely on three approaches: (1) Extending the conjugation length between the two unpaired electrons in accordance with Clar's rule, which effectively tunes the electronic ground state of diradicals [39, 40, 41, 42]; (2) Modifying the molecular skeleton and altering the positions of substituents [43, 44, 45]; (3) Varying the spacer length between the two radical centers to fine‐tune the open‐shell character [46, 47, 48, 49, 50, 51]. These strategies generally require modifications to the molecular skeleton. In contrast, NHCs allow flexible structural modifications through their backbones, enabling precise control over frontier orbital energy levels and Δ*E*_ST_ of the derived diradicals, allowing the spin characteristics and diradical character, *y*_0_, of carbon‐centered diradicals to be fine‐tuned.

Although numerous NHCs with diverse backbones have been reported [52, 53, 54, 55, 56, 57, 58, 59, 60], and only a few have been successfully used to stabilize carbon‐centered radicals, owing to the synthetic challenges associated with NHCs featuring extended conjugated backbones [61, 62, 63, 64]. N‐Heterocyclic carbene (NHC)‐based quinoids have been reported [65, 66], though open‐shell characteristics have yet to be realized in such systems. In this work, we performed a systematic study employing TPE as a central linker, introducing varying NHC backbones to regulate the orbital energy levels and electrochemical behavior of the radical precursor cation. The conjugated framework of TPE enables two‐electron delocalization across the *π*‐system, thereby enhancing diradical stability [67, 68]. This approach yields a 1,3‐butadiene‐type carbon diradical with either an open‐shell singlet (OS) or a closed‐shell singlet (CS, quinoidal) ground state. As the extent of conjugation and the electron‐withdrawing capacity of the NHC increased, the lowest unoccupied molecular orbital (LUMO) energy levels of the precursor salts decreased, enhancing stability and diradical character *y*_0_ from 0 up to 0.56 in the best case. X‐ray crystallographic analysis revealed distinct structural differences between the precursor cations and the diradicals. The strong electron‐withdrawing ability of [pyrazine]‐[1,3‐bis(2,6‐diisopropylphenyl)imidazol‐2‐ylidene] (PzIPr) (Figure 1b,c) enhances electron delocalization, promoting the formation of an open‐shell singlet ground‐state disjointed diradical (*y*_0_ = 0.56), whereas with the non‐conjugated [dimethyl]‐[1,3‐bis(2,6‐diisopropylphenyl)imidazol‐2‐ylidene] (IPr^Me^) (Figure 1b,c) remains in a closed‐shell singlet ground state. The pronounced open‐shell character thus fosters strong electronic communication along the *π*‐backbone, a feature that is crucial for achieving high charge carrier mobility [69, 70, 71]. Organic semiconductors form the foundation of flexible and low‐cost electronic devices [72, 73, 74, 75, 76, 77], with charge carrier mobility in organic field‐effect transistors (OFETs) being a decisive factor that governs overall performance [78, 79, 80, 81, 82, 83, 84]. OFETs based on compound **2d** (Figure 1c), an engineered structure with an intermediate diradical character of 0.56, achieved a record‐high hole mobility of 4.53 cm^2^·V^−1^·s^−1^, demonstrating the exceptional potential of open‐shell organic semiconductors for high‐performance OFETs.

## 2 Results and Discussion

### 2.1 Synthesis and Characterization of 1,3‐Butadiene‐Type Carbon Diradicals Precursor 1a–d

**FIGURE 2:**
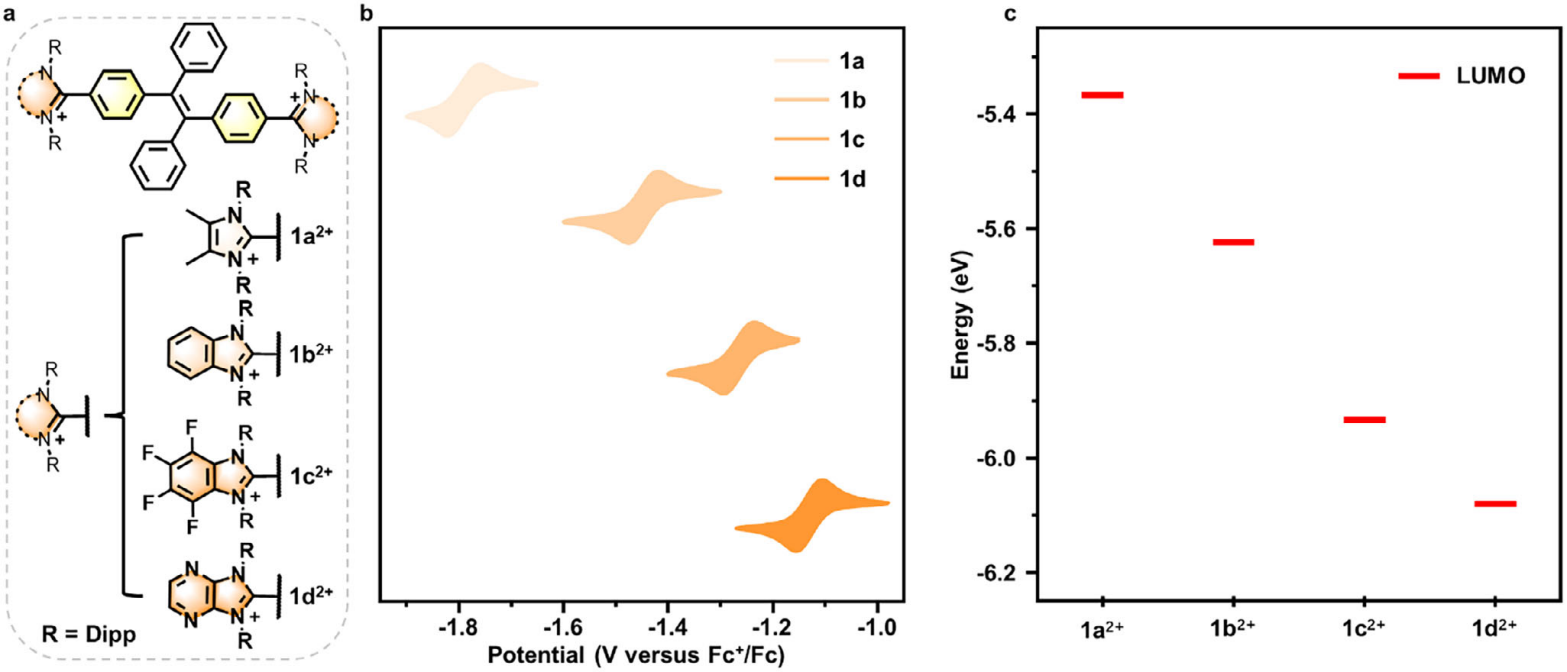
(a) The structures of **1a** ^2+^–**d** ^2+^. (b) Cyclic voltammograms of **1a**–**d** in CH_3_CN with 0.1 m *n*Bu_4_NPF_6_ at a scan rate of 100 mV·s^−1^. (c) Calculated energies of the LUMOs of **1a** ^2+^–**d** ^2+^ at the M06‐2X/6‐311G^**^ level of theory.

Compounds **1a**–**d** (Figure 2a) were synthesized using modified literature protocols [85, 86]. All compounds were comprehensively characterized by ESI mass spectrometry, NMR spectroscopy, and single‐crystal x‐ray diffraction (Figures S1–S17). As the electron‐withdrawing ability of the NHC increases, red shifts in the UV–vis absorption spectra from *λ*_max_ = 360, 274, 235 nm (**1a**) to *λ*_max_ = 384, 298, 254 nm for **1d** are observed, with a 37 nm‐red shift for *λ*_onset_ (Figure S18 and Table S1). The electrochemical properties of **1a**–**d** were evaluated by cyclic voltammetry (CV) in acetonitrile using tetrabutylammonium hexafluorophosphate as the supporting electrolyte at a scan rate of 100 mV·s^−1^ (Figure 2b). The cyclic voltammograms reveal fully reversible redox processes corresponding to a two‐electron reduction from the cationic state to the corresponding neutral radical species. The electronic properties of the NHC backbones strongly influence the redox potentials. Increased conjugation and electronegativity of the NHC result in an anodic shift in the half‐wave potential (*E*_1/2_). Through modulation of the electronic properties of the NHC, an anodic shift of approximately 660 mV in *E*_1/2_ was observed across the series from **1a** to **1d**. Specifically, the *E*_1/2_ value of **1b** (Figure 2a), which features a [benzene]‐[1,3‐bis(2,6‐diisopropylphenyl)imidazol‐2‐ylidene] (BzIPr) moiety, is about 350 mV more positive than that of **1a**, attributable to the enhanced conjugation of the NHC. The introduction of perfluorophenyl in **1c** (Figure 2a) results in a further positive shift of approximately 170 mV compared to **1b**. Conversely, replacement with a pyrazine in **1d** results in an additional anodic shift of roughly 310 mV relative to **1b**. Notably, these compounds are electrochemically stable with no alteration in redox behavior observed after 100 consecutive scanning cycles (Figures S19–S22).

Density functional theory (DFT) calculations were performed on **1a–d** to determine their frontier molecular orbitals. Geometry optimizations were performed at the B3LYP/6‐31G^*^ level, and energy levels were computed using the M06‐2X/6‐311G^**^ method. As both the extent of conjugation and electron‐withdrawing strength of the NHC backbone increase, the LUMO energy levels (Figure 2c) of **1a**–**d** decrease, accompanied by a narrowing of the HOMO–LUMO energy gap (Figure S23 andS24). A lower LUMO level indicates that the molecule can accept electrons more easily, which is a prognostic of the stability of **2a**–**d** derived from **1a**–**d**. The trends of **1a**–**d** are in good agreement with the trends observed in the cyclic voltammetry experiments. The reverse oxidation process of **2a**–**d** takes place following treatment with AgOTf, affording the dicationic precursors **1a** ^2+^–**d** ^2+^, confirmed by mass spectrometry (Figures S25–S28).

### 2.2 X‐Ray Crystal Structures

**FIGURE 3:**
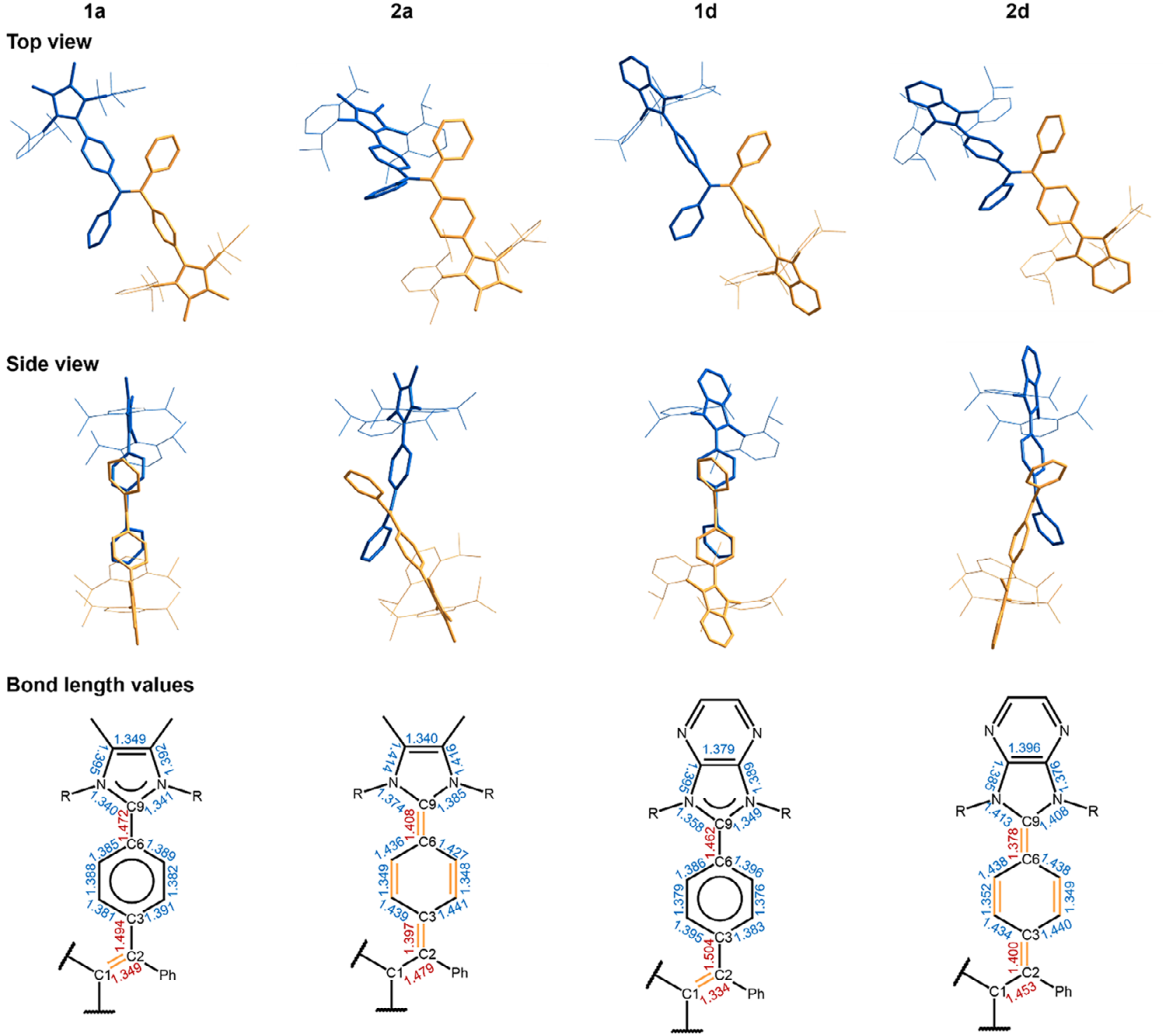
X‐ray solid‐state structures of compounds **1a** ^2+^, **1d** ^2+^, **2a**, and **2d**. Hydrogen atoms, counteranions, and solvates have been omitted for clarity. See Figures S14, S17, S29, and S31 and Table S2 for further details.

Reduction of **1a**–**d** with 2.2 equivalents of KC_8_ in anhydrous THF yields intensely colored solutions, from which diradicals **2a**–**d** were isolated in approximately 90% yield. Compounds **2a**–**d** are stable for extended periods under an inert atmosphere. Single‐crystal x‐ray diffraction data were obtained for both **1a**,**d** and diradicals **2a**,**d** (Figure 3). Complete bond lengths and angles are provided in Figures S14, S17, S29–S32, and Table S2. Crystallographic analysis of **1a** and **1d** reveals well‐defined TPE frameworks. The central C1═C2 bond lengths are consistent with typical double bonds, measuring 1.349(4) Å in **1a** and 1.334(6) Å in **1d**. In contrast, the C2─C3 and C6─C9 bonds exhibit single‐bond character, with distances of 1.494(3) and 1.472(3) Å in **1a**, and 1.504(3) and 1.462(3) Å in **1d** (Table S2). The bonds of the phenyl‐linker show similar lengths of approximately 1.37–1.40 Å, indicating a delocalized *π*‐electron structure. The dihedral angles between the central C1═C2 bond and the adjacent phenyl rings are less than 10°, which represents a nearly coplanar conformation.

The neutral diradicals **2a** and **2d** display markedly different structural features from **1a** and **1d**. The central C─C bond in **2a** is twisted, with a dihedral angle of 54.7°, and elongated to 1.479(4) Å, consistent with a C(*sp* ^2^)–C(*sp* ^2^) single bond. Concurrently, the C2─C3 and C6─C9 bonds are shortened to 1.397(4) and 1.408(4) Å, respectively (Table S2). The phenyl rings of the TPE central linker exhibit bond alternation (ca. 1.44 and 1.35 Å), indicative of a quinoidal distortion and dearomatization. In **1a** and **1d**, the dihedral angle is very small and is similar to that in unfunctionalized TPE (6.5° and 0° vs. 10°). However, the central bond undergoes a transition to a single bond following reduction, accompanied by a reorganization of the *π*‐system into two spatially separated quinoidal subunits. Although **2d** shows similar trends in bond lengths as **2a**, its central dihedral angle is smaller (35.6°), which may be attributed to stronger spin delocalization facilitated by the NHC. This structural feature is consistent with the OS ground state assigned to **2d** (see below).

### 2.3 Photophysical Properties

UV–vis absorption spectra of **1a**–**d** and **2a**–**d** were recorded at room temperature in anhydrous THF (Figures S33–S36). Compounds **1a**–**d** exhibit a short‐wavelength absorption at 372 ± 12 nm. In contrast, diradicals **2a**–**d** display three absorption bands, i.e. a short‐wavelength band in the range 315–365 nm, a broad band between 435 and 480 nm (*ε* = 8662.0 (**2a**), 11032.1 (**2b**), 20513.3 (**2c**), 6743.3 (**2d**) M^−1^·cm^−1^), and a broad absorption band in the visible region with a maximum absorption wavelength (*λ*_max_) in the range 655–690 nm (*ε* = 10783.9 (**2a**), 7711.2 (**2b**), 16443.5 (**2c**), 7193.2 (**2d**) M^−1^·cm^−1^).

Emission spectra of **1a**–**d** display distinct emissions, attributable to the presence of the TPE chromophore within their structures. As expected, diradicals **2a**–**d** exhibit no discernible emissions, which may be attributed to efficient non‐radiative decay pathways that dominate the deactivation process in these radicals (Figures S33–S36).

### 2.4 Spin‐State Analysis of 2a–d

To further elucidate the electronic structures and ground states of **2a**–**d**, DFT calculations were carried out using the unrestricted broken‐symmetry UBHandHLYP/def2‐SVP method. Three electronic ground states were considered, i.e., CS, OS, and open‐shell triplet (T). Notably, for **2a**, the initial structure was set as an OS diradical. However, geometric optimization converged to a CS ground state. The computed singlet–triplet energy gap is small (Δ*E*_ST_ = −5.74 kcal·mol^−1^), suggesting a substantial presence of triplet state character under ambient conditions. This finding is corroborated by variable‐temperature EPR data fitted with the Bleaney–Bowers equation, which yielded a comparable Δ*E*_ST_ value of −2.70 kcal·mol^−1^ (Figure S37, and S44). The absence of hyperfine coupling interactions further indicates the extensive delocalization of the unpaired electrons in **2a** [87]. The consistency between computational and experimental results supports the description of **2a** as a resonance hybrid between triplet diradical and quinoidal structures.

**FIGURE 4:**
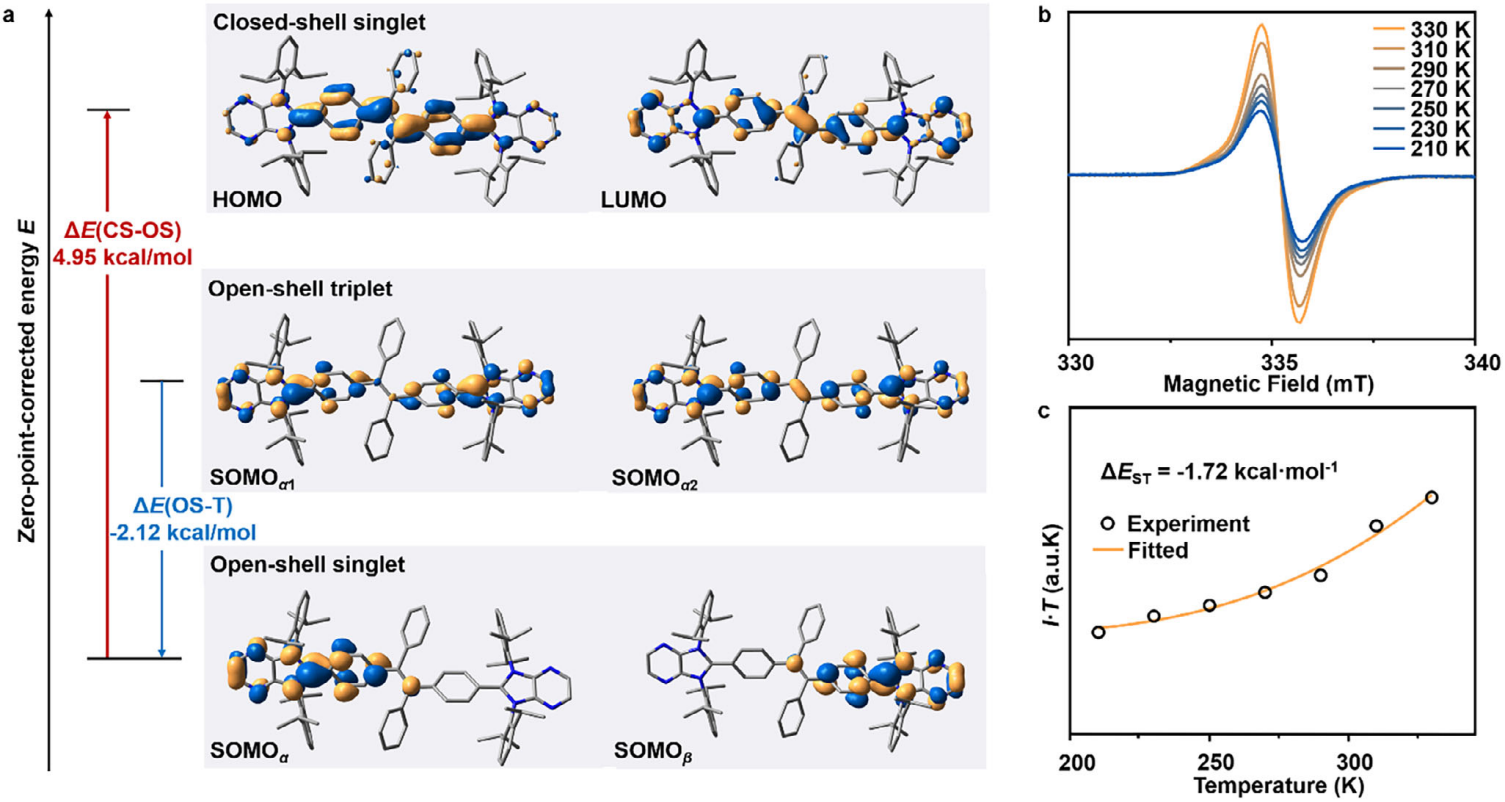
(a) The frontier molecular orbitals (isovalue = 0.033) of **2d** in the CS (top), T (middle), and OS (bottom) states, and relevant adiabatic energy differences Δ*E* calculated at the UBHandHLYP/def2‐SVP level of theory. Color scheme: C gray; N Klein blue. (b) Variable temperature EPR spectra of **2d** in the solid state. (c) The product of EPR signal integrations (*I*) and temperature (*T*) vs. temperature (*T*) of **2d** and the fitted curve from the Bleaney–Bowers equation (orange line). Circles are the experimental results.

In contrast to **2a**, diradicals **2b**–**d** exhibit an OS ground state (Figure S38 and S39, Figure S45 and S46). Quantum chemical calculations reveal that the *α*‐ and *β*‐spin singly occupied molecular orbitals (SOMOs) in **2d** display typical disjointed character, indicative of significant singlet diradical character (Figure 4a). The diradical character *y*_0_ was calculated to be 0.30 for **2b**, 0.42 for **2c**, and 0.56 for **2d**, indicating a moderate level of diradical behavior in **2d**. Further DFT optimization of **2d** in a hypothetical CS configuration showed that this state lies higher in energy than the triplet state, with relative energies of Δ*E*(CS–OS) = 4.95 and Δ*E*(OS–T) = −2.12 kcal·mol^−1^ (Figure 4a). This is consistent with the small Δ*E*_ST_ derived from variable‐temperature EPR data fitted using the Bleaney–Bowers equation, which yielded Δ*E*_ST_ = −1.72 kcal·mol^−1^ (Figure 4b,c).

**FIGURE 5:**
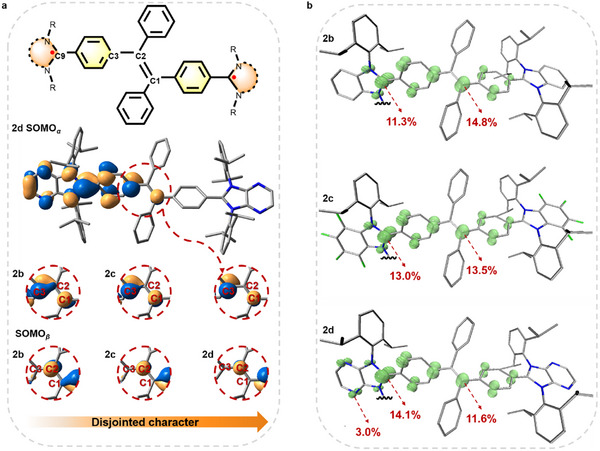
(a) Selected SOMOs (isovalue = 0.033) and (b) alpha spin densities (isovalue = 0.010) of **2b**–**d** in the OS ground state, calculated at the UBHandHLYP/def2‐SVP level of theory. The red dots in the illustration represent single electrons. Partial structures have been omitted for clarity.

As the diradical character *y*_0_ increases, the disjointed character of the SOMOs becomes more evident (Figure 5a; Figure S38 and S39). A progressive decrease in orbital occupancy at C1 and C2 is observed across series **2b**–**d**, as shown by the SOMO*_α_* and SOMO*_β_* orbitals. This trend correlates with increasing *y*_0_ values and reflects reduced overlap between the *α*‐ and *β*‐orbitals (SOMO*_α_* and SOMO*_β_*), further supporting the enhanced diradical character of **2d**. These findings confirm that employing pyrazine (Pz) on IPr effectively stabilizes the carbon‐centered diradical in **2d** by promoting the OS ground state, consistent with our design objective of favoring diradical resonance structures. To our knowledge, this constitutes the first reported instance where systematic modification of NHC backbones tunes the electronic properties of carbon‐centered diradicals.

Spin density analysis was carried out for **2b**–**d** (Figure 5b; Figures S40–S43, Table S3). With increasing electron‐withdrawing ability of the NHC moiety, the spin density on the carbene carbon (C9) increases markedly, from 11.3% in **2b** to 14.1% in **2d**. In contrast, the spin density at C1, which belongs to the TPE unit, decreases progressively (14.8% → 13.5% → 11.6%). This trend indicates that the stronger electron‐withdrawing nature of the NHC fragment stabilizes the unpaired electron on the carbene center, suppressing delocalization to the TPE unit and thereby favoring open‐shell radical character over a quinonoid structure. Moreover, in **2d**, the nitrogen atoms in the pyrazine ring of the PzIPr moiety introduce a pronounced heteroatom effect, as evidenced by the additional spin density localized on N3/N4 (3.0%/3.0%). The contribution from the heteroatoms further stabilizes the radical species through enhanced spin delocalization, ultimately increasing the overall diradical character of the system. Air and thermal stability were also studied. Visible color changes were observed in the solution after exposure to the air, due to the disruption of the radical *π*‐system through oxidation (Figures S47–S51). The thermal stability of **2a**–**d** was evaluated by thermogravimetric analysis (TGA). Among the series, **2d** exhibits the highest thermal stability, with a 5% decomposition temperature of 308°C (Figure S52). It is noteworthy that within this framework, greater diradical character correlates with a longer half‐life and higher thermal stability.

### 2.5 Organic Field‐Effect Transistor (OFET) Devices Based on 2d

**FIGURE 6:**
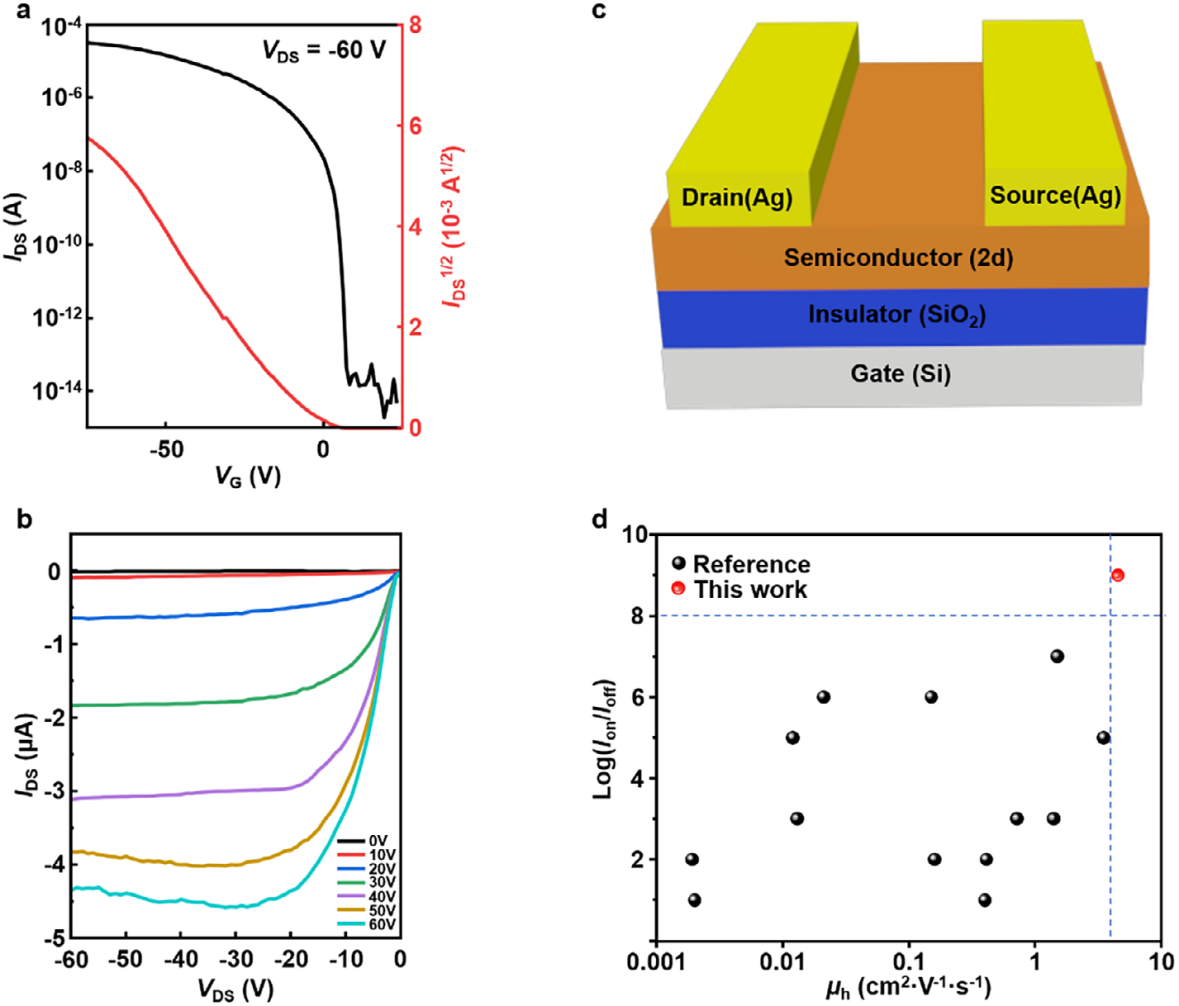
(a) Transfer, (b) output curves, and (c) schematic of the OFET device based on **2d** with a mobility of 4.53 cm^2^·V^−1^·s^−1^. *V*_DS_ = source‐drain voltage, *I*_DS_ = source‐drain current, and *V*_G_ = gate voltage. (d) Scattering plot of log(*I*_on_/*I*_off_) against hole mobility for the best‐performing open‐shell materials in the literature.

The open‐shell nature of diradicals promotes facile charge separation compared to closed‐shell structures. Notably, compounds possessing medium diradical character demonstrate exceptional potential for OFET applications [88], owing to their balanced reorganization energies for hole and electron transport. The energy levels are estimated through cyclic voltammetry and summarized (Table 1, Figure S53). Organic radicals usually suffer from poor on/off ratios due to unpaired electrons acting as intrinsic dopants. In contrast, a spin‐coated bottom‐gate/top‐contact (BG/TC) p‐type OFET device employing **2d** demonstrated exceptional performance, with a record‐high hole mobility of 4.53 cm^2^·V^−1^·s^−1^ among radical‐based OFET devices and an on/off ratio of 10^9^ (Figure 6; Table S4) [20, 21, 22, 69, 79, 88, 89, 90, 91, 92, 93, 94, 95, 96]. In contrast, no distinct field‐effect characteristics were observed in devices based on **2a**. The hole mobilities of **2b** and **2c** were measured to be 1.42 and 1.75 cm^2^·V^−1^·s^−1^, respectively (Figure S54 and S55). This discrepancy in performance may be attributed to their moderate diradical characters, which induce a certain degree of electronic localization (i.e., unpaired electrons), resulting in the formation of quasi‐hole and quasi‐electron particles with similar energies in the frontier orbitals. Subsequent charge extraction induces only minor structural rearrangements [88].

**TABLE 1:** Electrochemical and computational data for compounds **2a**–**d**.

Compound	*E*_OX_ (V)	HOMO/SOMO^a^ (eV)	*E*_RED_ (V)	LUMO/SUMO (eV)	*λ*_int_ ^b^(eV)
**2a**	0.15	−4.95	−1.92	−2.88	0.77
**2b**	0.14	−4.94	−1.62	−3.12	0.37
**2c**	0.10	−4.90	−1.38	−3.42	0.38
**2d**	0.12	−4.92	−1.26	−3.54	0.33

^a^ HOMO/SOMO​ and LUMO/SUMO were estimated from *E*_OX_​ and *E*_RED_ *vs*. Fc/Fc^+^ using the relation HOMO/SOMO = ‐(*E*_OX_​ + 4.8) eV and LUMO/SUMO = ‐(*E*_RED_​ + 4.8) eV. **2a**: HOMO, LUMO; **2b**–**d**: SOMO, SUMO.

^b^ *λ*_int_ = internal reorganization energy (hole) calculated at the UBHandHLYP/def2‐SVP level.

The n‐type transport behavior of **2d** was investigated using Ag as the electrode. No field‐effect transistor characteristics were observed. Based on the electrochemical data, the LUMO level of **2d** is estimated to be −3.54 eV, resulting in an electron‐injection barrier of around 0.8 eV with Ag electrodes (work function = 4.26–4.74 eV) (Figure S53). The absence of n‐type characteristics may be attributed to an energetic mismatch at the electrode interface.

Atomic force microscopy was used to evaluate the thin‐film morphologies of **2a**–**d** (Figure S56). Interestingly, the film topographies correlate with their structural variations. The distinct morphology of **2c** may be mainly attributed to its relatively poor solubility. While **2a**, **2b**, and **2d** possess sufficient solubility to form smooth and continuous films (RMS < 1.2 nm), the limited solubility of **2c** leads to premature precipitation during the spin‐coating process, resulting in a rougher surface (RMS = 2.45 nm), densely populated with pinholes. Grazing‐incidence small‐angle scattering (GIWAXS) was also performed. However, only a broad background peak was observed, indicating no obvious molecular stacking (Figure S57).

To elucidate the charge transport mechanism, internal reorganization energies (*λ*_int_) for **1a**–**d** (electron) and **2a**–**d** (hole) were calculated at the UBHandHLYP/def2‐SVP level of theory (Table 1; Table S5, Figure S58). Remarkably, despite the non‐planar molecular framework of **2d**, it yields a *λ*_int_ value of 0.33 eV, which is comparable to that of the high‐performance benchmark **C8BTBT** (0.32 eV) under the same computational framework [97]. This relatively low value of *λ*_int_ suggests that **2d** maintains a rigid core upon charge excitation, effectively lowering the thermal activation barrier for electron hopping. This favorable energetic factor partially offsets the structural disadvantage of non‐optimal *π*–*π* stacking, thereby sustaining its efficient charge transport characteristics. In contrast, **2a** yields a *λ*_int_ value of 0.77 eV due to its closed‐shell ground state. The electron reorganization energies of **1a**–**d** were also calculated, yielding values exceeding 0.65 eV, which are prohibitively large for efficient charge transport in OFET devices. No field‐effect behavior was observed for **1d**, consistent with the high mobility observed in **2d** arising from its open‐shell radical character, rather than being an inherent property of the molecular framework. The solid‐state packing of the single crystals is shown (Figure S30, and S32). The intermolecular interactions in these systems are relatively weak, with no significant *π*–*π* stacking observed. This suggests that the solid‐state packing is unlikely to be the dominant factor governing the charge transport properties of the diradical species **2b**–**d**.

The low reorganization energy of the non‐planar diradical **2d** may be attributed to its frontier orbitals (the SOMOs), which possess predominantly “non‐bonding” character. Since these non‐bonding orbitals contribute minimally to the structural framework, the addition or removal of an electron during charge transport is expected to have a negligible perturbation on the backbone bond orders, resulting in minimal structural changes to the molecular geometry (Figure S59). These findings highlight the promising charge transport properties of **2d** as a high‐performance material and further demonstrate the effectiveness of modulating the NHC backbone to tune the semiconductor performance of organic diradicals.

## 3 Conclusions

Our results demonstrate that extending conjugation and introducing electron‐deficient groups effectively enhances the *π*‐accepting ability of NHC backbones, leading to lowered orbital energy levels and reduced HOMO–LUMO gaps in the compounds **1a**–**d**. Two‐electron reduction of **1a**–**d** with KC_8_ yields the neutral diradicals **2a**–**d**, whose ground‐state electronic structures were systematically investigated using unrestricted broken‐symmetry DFT calculations alongside experimental techniques. Single‐crystal x‐ray diffraction and DFT analysis provide detailed structural insights. In contrast to the planar geometry of **1a**–**d**, **2a**–**d** exhibit varying degrees of structural distortion, including loss of the original TPE motif. Accompanying this change, the central C─C bond elongates. This length is consistent with C(*sp* ^2^)–C(*sp* ^2^) single bonds. Theoretical studies indicate that **2a** adopts a CS ground state with a small Δ*E*_ST_, consistent with variable‐temperature EPR measurements and enabling room‐temperature paramagnetic behavior. In contrast, **2b**–**d** feature an OS ground state with disjointed SOMOs and moderate diradical character (e.g., *y*_0_ = 0.56 for **2d**), attributed to efficient spin delocalization imparted by the PzIPr moiety. Notably, **2d** demonstrates a value of 4.53 cm^2^·V^−1^·s^−1^ in OFET, which represents a record among open‐shell organic semiconductors. The observation of high mobility in the presence of an unfavorable *π*–*π* stacking geometry can be rationalized by the open‐shell nature of the **2d**, resulting in a reduced reorganization energy. This work highlights the feasibility of tuning diradical electronic ground states through a rational NHC backbone modulation strategy, thereby elucidating key structure–property relationships in this class of diradicals, providing new insights for the design and synthesis of organic diradical compounds, and opening up new avenues for open‐shell materials with potential applications in molecular electronics.

## 4 Experimental Section

### 4.1 General Information

^1^H NMR, ^13^C{^1^H} NMR, and ^19^F NMR spectra were recorded on a Bruker AVANCE I 400 MHz, a Bruker AVANCE III HD 600 MHz with cryoprobe, or a JNM‐ECZ400S spectrometer. Chemical shifts (*δ*) are expressed in ppm downfield from tetramethylsilane using the residual protonated solvent as an internal standard. Coupling constants are expressed in Hertz. Mass spectra were obtained with a Bruker microTOF‐Q II electrospray ionization (ESI) mass spectrometer (Bruker Daltonics Corp., USA). UV–vis spectra were recorded on an Agilent Cary 100 spectrophotometer. Fluorescence spectra were obtained on a Horiba QuantaMaster 8000 spectrometer. Cyclic voltammetry (CV) experiments were performed using a CHI660E electrochemical workstation (CH Instruments, Inc.). All experiments were conducted under a nitrogen atmosphere in anhydrous acetonitrile containing *n*Bu_4_NPF_6_ (0.1 m) at a scan rate of 100 mV·s^−1^. The setup consisted of a glassy carbon working electrode, a platinum wire counter electrode, and a silver wire inserted in a small glass tube fitted with a porous Vycor frit and filled with an AgNO_3_ solution in anhydrous acetonitrile (0.01 m). Ferrocene was used as a standard, and all reduction potentials are reported with respect to the *E*_1/2_ of the Fc^+^/Fc redox couple. Continuous wave (CW) EPR spectra were obtained using an X‐band Bruker E500 spectrometer. The microwave frequency was 9.8 GHz, and the modulation amplitude was 0.1 mT.

DFT calculations were executed using the Gaussian 09 program package [98]. The cation geometries were optimized using the crystal structure coordinates as the starting structure. Calculations of cation were performed using the B3LYP with the 6–31G^*^ basis set, and energy levels were computed using the M06‐2X with the 6–311G^**^ basis set [99, 100]. Calculations of radicals were performed using the unrestricted BHandHLYP with the def2‐SVP basis set [101]. Frequency calculations were carried out to ensure that the optimized geometries were minimal on the potential energy surface and that no imaginary frequencies were observed in any of the compounds. Visualization of the structures was performed using Visual Molecular Dynamics (VMD) software, version 1.9.3 [102].

Single crystals suitable for x‐ray diffraction analysis of **1b** and **1c** were grown by diffusing the antisolvent diethyl ether into saturated acetonitrile solutions of the samples. However, repeated attempts to grow diffraction‐quality single crystals of the bromide salts of **1a** and **1d** were unsuccessful. To address this, anion exchange was performed. For compound **1a**, a two‐step reaction involving AgOTf and tetrabutylammonium chloride was carried out to obtain the chloride salt, which yielded crystals suitable for x‐ray analysis. For compound **1d**, anion exchange was conducted using AgOTf to afford the triflate salt, from which high‐quality single crystals were successfully obtained. Diffraction‐quality single crystals of the radicals **2a**,**d** samples were obtained by adding a large excess of *n*‐pentane to their saturated THF solution, followed by crystallization at −35°C in a glove box. Single crystal x‐ray diffraction data of **1a**–**d** and **2a**,**d** were collected with a Bruker APEX‐II CCD diffractometer x‐ray source. Raw data collection and processing were performed with the APEX III software package. The data were corrected for absorption using the SADABS program. Structure solutions were found with the olex2 program package. Structure refinement was performed with the SHELXL refinement package using Least Squares minimization. Several disordered solvent molecules could not be restrained properly and were removed using the SQUEEZE routine. Non‐hydrogen atoms were refined anisotropically, and hydrogen atoms were treated as idealized contributions [103, 104, 105]. All crystal and data collection details are summarized in Tables S6–S11.

### 4.2 Synthesis and Characterization

The experimental details on the synthesis and characterization of all compounds are presented in the Supporting Information.

[CCDC 2498013, 2498014, 2498015, 2498016, 2498017, and 2498019 contain the supporting crystallographic data for this paper. These data can be obtained free of charge from The Cambridge Crystallographic Data Centre via www.ccdc.cam.ac.uk/data_request/cif.]

### 4.3 Materials

Compound (*E*)‐1,2‐bis(4‐bromophenyl)‐1,2‐diphenylethene, (*E*)‐1,2‐bis(4‐iodophenyl)‐1,2‐diphenylethene and free carbenes were synthesized following literature procedures [106, 107, 108, 109, 110, 111]. All other chemical reagents were purchased from Energy Chemical and were used directly without further purification. All reactions were carried out under an argon atmosphere using standard Schlenk techniques. Solvents were freshly distilled by standard procedures.

### 4.4 OFET Device

BG/TC OFETs were fabricated on SiO_2_/Si substrates. The substrates were sequentially cleaned using deionized water, acetone, and isopropyl alcohol, followed by drying with a nitrogen stream. The silicon dioxide layer functions as the gate dielectric, while the silicon wafer serves as the gate electrode. The organic semiconductor film was deposited by spin‐coating a solution of **2a**–**d** in *o*‐dichlorobenzene (3 mg/mL), first at 200 rpm for 10 s and then at 2000 rpm for 30 s. Top‐contact silver source and drain electrodes (50 nm thick) were thermally evaporated through a shadow mask.

Electrical characterization of the transistors was performed inside a glovebox at room temperature under an inert atmosphere using an Agilent B1500 semiconductor parameter analyzer. The field‐effect mobility (*μ*) was extracted in the saturation region using the equation:

$$\mu_{\mathrm{sat}}=\left(2I_{\mathrm{DS}}L\right)/\left[{\mathrm{WC}}_{i}{\left(V_{G}\;-\;V_{\mathrm{th}}\right)}^{2}\right]$$

where *I*_DS_ denotes the drain‐source saturation current, *C*_i_ is the areal capacitance of the gate dielectric, *V*_G_ represents the gate voltage, and *V*_th_ refers to the threshold voltage. The value of *V*_th_ was determined from the intercept of the linearly extrapolated segment in the plot of *V*_G_ vs. (*I*_DS_)^1/2^.

## Conflicts of Interest

The authors declare no conflicts of interest.

## Supporting information




**Supporting File**: adma73264‐sup‐0001‐SuppMat.docx.

## Data Availability

The data that support the findings of this study are available from the corresponding author upon reasonable request.
