# Supplementary material for: Backbone Engineering of Carbon‐Centered NHC‐Derived Diradicals: From Electronic State Tuning to High‐Performance Organic Field‐Effect Transistors
Source: Adv Mater. 2026 May 5;38(32):e73264. doi: 10.1002/adma.73264 (PMC13244804; doi:10.1002/adma.73264)
Supplement: Supplementary file 1 — Supporting File: adma73264‐sup‐0001‐SuppMat.docx. [file ADMA-38-e73264-s001.docx]

Supporting Information

Backbone Engineering of Carbon-Centered NHC-Derived Diradicals: from Electronic State Tuning to High-Performance Organic Field-Effect Transistors

Xiao-Xu Liu^1,†^, Lianghui Li^2,†^, Xin Li^1,3,†^, Can Chen^1^, Man Li^1^, Shun Tian^2^, Paul J. Dyson^2,^* and Ying-Feng Han^1,3,^*

^1^College of Chemistry and Materials Science, Northwest University, Xi’an, 710127, P. R. China. ^2^Institut des Sciences et Ingénierie Chimiques, École Polytechnique Fédérale de Lausanne (EPFL), CH-1015, Lausanne, Switzerland. ^3^College of Chemistry, Zhengzhou University, Zhengzhou, 450001, P. R. China.

[*paul.dyson@epfl.ch*](mailto:paul.dyson@epfl.ch)*; yfhan@nwu.edu.cn*

Table of Contents

[Synthesis and characterization of **1a**–**d** 1](#_Toc218323183)

[Synthesis and characterization of **2a**–**d** 15](#_Toc218323184)

[Characteristics of OFET compared to the literature 32](#_Toc218323185)

[Single-crystal X-ray diffraction studies 36](#_Toc218323186)

[Cartesian coordinates of the optimized geometries from DFT calculations 42](#_Toc218323187)

[References 79](#_Toc218323188)

# Synthesis and characterization of 1a–d

__

**PzIPr·BF_4_**: *N*^2^,*N*^3^-bis(2,6-diisopropylphenyl)pyrazine-2,3-diamine (2.15 g, 5.0 mmol) and ammonium fluoborate (598.6 mg, 5.5 mmol) were dissolved in triethyl orthoformate (35 mL). The reaction mixture was heated at 120 °C overnight, resulting in a white suspension. The product was isolated by filtration, washed with ethyl acetate, and dried *in vacuo* to give PzIPr·BF_4_ as a white solid. Yield: 1.72 g (3.25 mmol, 65%). The ^1^H NMR spectrum of the compound is consistent with the reported data.

**General procedure for the preparation of free carbenes**

Free carbenes IPr^Me^ & BzIPr: A mixture of the respective imidazolium salt (1.0 mmol, 1.0 equiv.) and potassium tert-butoxide (1.1 mmol, 1.1 equiv.) in dioxane (20 mL) was stirred at room temperature overnight. The reaction mixture was then centrifuged, and the supernatant was used directly in the next step.

Free carbene BIPr^F^: The imidazolium salt (1.0 mmol, 1.0 equiv.) was treated with sodium hydride (1.1 mmol, 1.1 equiv.) in *o*-xylene (20 mL) and stirred at room temperature overnight. The reaction mixture was then centrifuged, and the supernatant was used directly in the next step.

Free carbene PzIPr: Following the procedure for IPr^Me^ & BzIPr, the reaction was conducted in dioxane (20 mL) with potassium tert-butoxide (1.1 mmol, 1.1 equiv.). Critical to this step, the reaction time was limited to 4 hours at room temperature, as prolonged periods lead to degradation of the product. The reaction mixture was then centrifuged, and the supernatant was used directly in the next step.

__

**1a**: (*E*)-1,2-bis(4-bromophenyl)-1,2-diphenylethene (245.1 mg, 0.5 mmol) and Ni(cod)_2_ (27.5 mg, 0.1 mmol) were added to a solution of free carbene IPr^Me^ (416.7 mg, 1.0 mmol) in dioxane (20 mL). The reaction mixture was stirred at 120 °C for 12 h. The mixture was cooled to ambient temperature. Then, the solution was concentrated, and petroleum ether was added to precipitate a solid, which was isolated by filtration, washed with ethyl acetate, and dried *in vacuo* to give **1a** as a yellow solid. Yield: 512.9 mg (0.388 mmol, 78%). ^1^H NMR (400 MHz, CD_3_CN) *δ* = 7.60 (t, *J* = 7.8 Hz, 4H), 7.39 (d, *J* = 7.8 Hz, 8H), 7.04 (t, *J* = 7.5 Hz, 2H), 6.90 (t, *J* = 7.7 Hz, 4H), 6.71 (d, *J* = 8.7 Hz, 4H), 6.64 (d, *J* = 8.7 Hz, 4H), 6.55 (d, *J* = 6.7 Hz, 4H), 2.38 (m, 8H), 2.10 (s, 12H), 1.19 (d, *J* = 6.7 Hz, 24H), 0.87 ppm (d, *J* = 6.7 Hz, 24H). ^13^C{^1^H} NMR (150 MHz, CD_3_CN) *δ* = 147.9, 146.3, 143.6, 142.1, 141.8, 133.2, 132.0, 131.3, 131.2, 129.5, 129.4, 129.1, 128.3, 126.8, 120.3, 29.6, 24.8, 23.6, 10.2 ppm. HRMS (ESI, positive ions): *m*/*z* = 581.3885 (calcd for [C_84_H_98_N_4_]^2+^ 581.3890).

__

**1b**: (*E*)-1,2-bis(4-bromophenyl)-1,2-diphenylethene (245.1 mg, 0.5 mmol) and Ni(cod)_2_ (27.5 mg, 0.1 mmol) were added to a solution of free carbene BzIPr (438.7 mg, 1.0 mmol) in dioxane (20 mL). The reaction mixture was stirred at 120 °C for 12 h. The mixture was cooled to ambient temperature. Then, the solution was concentrated, and petroleum ether was added to precipitate a solid, which was isolated by filtration, washed with ethyl acetate, and dried *in vacuo* to give **1b** as a yellow solid. Yield: 424.8 mg (0.311 mmol, 62%). ^1^H NMR (600 MHz, CD_3_CN) *δ* = 7.77-7.74 (m, 4H), 7.70 (t, *J* = 7.8 Hz, 4H), 7.49-7.46 (m, 12H), 7.08 (t, *J* = 7.4 Hz, 2H), 6.96-6.88 (m, 12H), 6.62 (m, 4H), 2.27 (m, 8H), 1.05 (d, *J* = 6.7 Hz, 24H), 0.92 ppm (d, *J* = 6.7 Hz, 24H). ^13^C{^1^H} NMR (150 MHz, CD_3_CN) *δ* = 150.7, 149.6, 146.8, 142.2, 141.8, 134.0, 133.6, 132.6, 131.4, 130.2, 130.0, 129.2, 128.6, 128.3, 127.0, 119.2, 115.3, 30.0, 25.3, 23.3 ppm. HRMS (ESI, positive ions): *m*/*z* = 603.3707 (calcd for [C_88_H_94_N_4_]^2+^ 603.3734).

__

**1c**: (*E*)-1,2-bis(4-iodophenyl)-1,2-diphenylethene (292.1 mg, 0.5 mmol) and Pd_2_(dba)_3_ (45.8 mg, 0.05 mmol) were added to a solution of free carbene BIPr^F^ (511.6 mg, 1.0 mmol) in *ο*-xylene (20 mL). The reaction mixture was stirred at 150 °C for 12 h. The mixture was cooled to ambient temperature. Then, the solution was concentrated, and petroleum ether was added to precipitate a solid, which was isolated by filtration, washed with ethyl acetate, and dried *in vacuo* to give **1c** as a yellow solid. Yield: 411.4 mg (0.256 mmol, 51%). ^1^H NMR (600 MHz, CD_3_CN) *δ* = 7.68 (t, *J*^2^ = 7.9 Hz, 4H), 7.45 (d, *J*^2^ = 7.9 Hz, 8H), 7.09 (t, 2H), 6.96-6.89 (m, 8H), 6.84 (d, *J*^2^ = 8.7 Hz, 4H), 6.58 (d, *J*^2^ = 7.1 Hz, 4H), 2.40 (m, 8H), 1.12 (d, *J*^2^ = 6.7 Hz, 24H), 0.92 ppm (d, *J*^2^ = 6.7 Hz, 24H). ^13^C{^1^H} NMR (150 MHz, CD_3_CN) *δ* = 153.7, 150.3, 146.5, 142.3, 141.6, 134.2, 132.7, 131.4, 130.3, 129.3, 129.0, 128.8, 127.0, 118.3, 30.1, 25.0, 23.4 ppm. ^19^F NMR (564 MHz, CD_3_CN) *δ* = -154.4 (d, *J*^2^ = 18.1 Hz, 4F), -156.5 ppm (d, *J*^2^ = 17.6 Hz, 4F). HRMS (ESI, positive ions): *m*/*z* = 675.3348 (calcd for [C_88_H_86_F_8_N_4_]^2+^ 675.3357).

__

**1d**: (*E*)-1,2-bis(4-bromophenyl)-1,2-diphenylethene (245.1 mg, 0.5 mmol) and Ni(cod)_2_ (27.5 mg, 0.1 mmol) were added to a solution of free carbene PzIPr (440.6 mg, 1.0 mmol) in dioxane (20 mL). The reaction mixture was stirred at 120 °C for 12 h. The mixture was cooled to ambient temperature. Then, the solution was concentrated, and petroleum ether was added to precipitate a solid, which was isolated by filtration, washed with ethyl acetate, and dried *in vacuo* to give **1d** as a yellow solid. Yield: 371.2 mg (0.271 mmol, 54%). ^1^H NMR (400 MHz, CD_3_CN) *δ* = 8.90 (s, 4H), 7.69 (t, *J* = 7.8 Hz, 4H), 7.47 (d, *J* = 7.8 Hz, 8H), 7.11 (t, *J* = 7.5 Hz, 2H), 7.00-6.88 (m, 12H), 6.63 (d, *J* = 7.0 Hz, 4H), 2.31 (m, 8H), 1.06 (d, *J* = 6.6 Hz, 24H), 0.88 ppm (d, *J* = 6.6 Hz, 24H). ^13^C{^1^H} NMR (150 MHz, CD_3_CN) *δ* = 153.6, 150.7, 147.1, 147.0, 142.4, 141.7, 139.7, 133.9, 132.9, 131.5, 130.9, 129.4, 128.8, 127.2, 126.9, 30.2, 25.4, 23.1 ppm. HRMS (ESI, positive ions): *m*/*z* = 605.3649 (calcd for [C_84_H_90_N_8_]^2+^ 605.3639), 1291.6403 (calcd for [C_84_H_90_N_8_Br]^+^ 1291.6453).


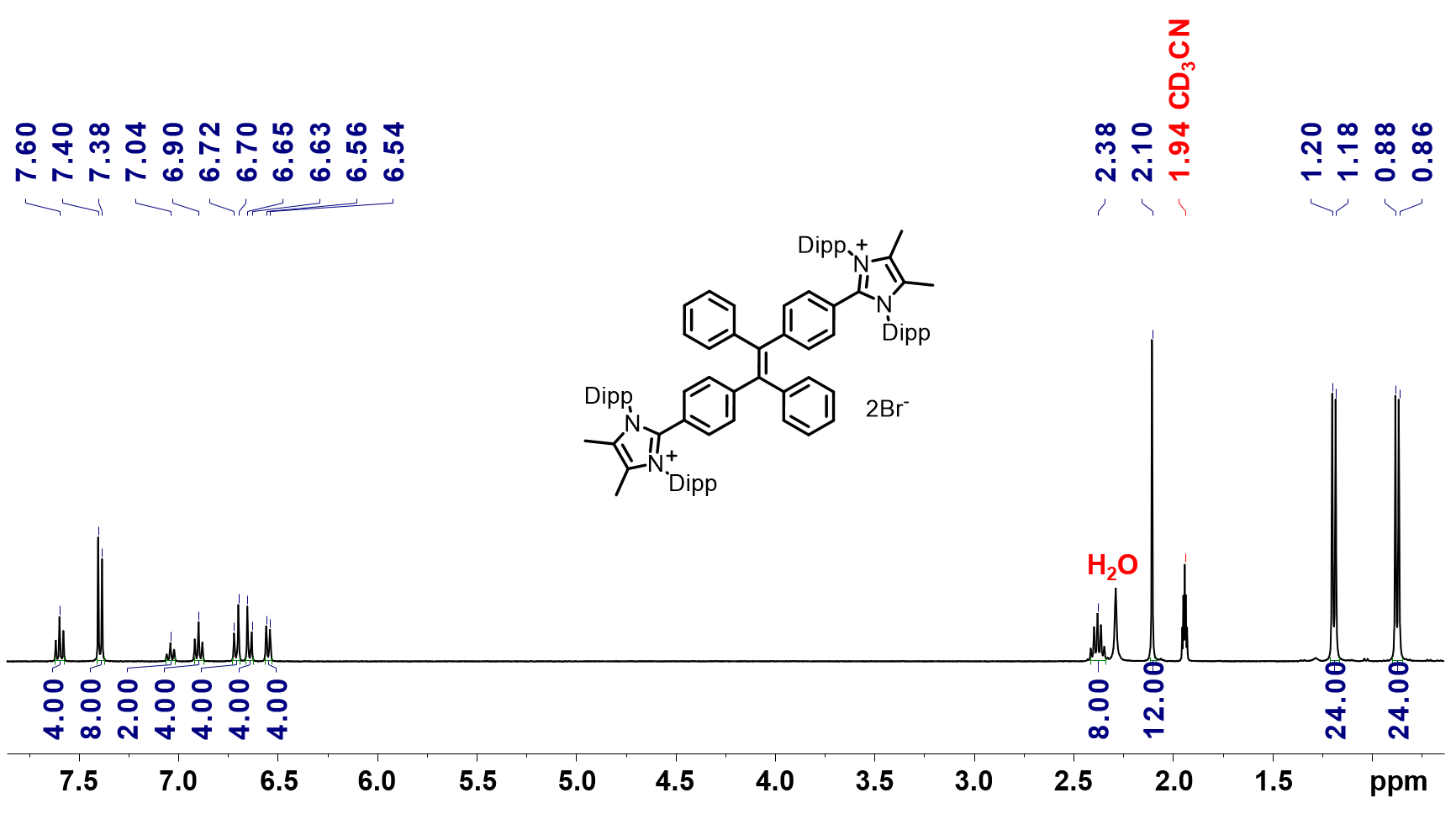


**Figure S1**. ^1^H NMR spectrum (400 MHz, CD_3_CN) of **1a**.


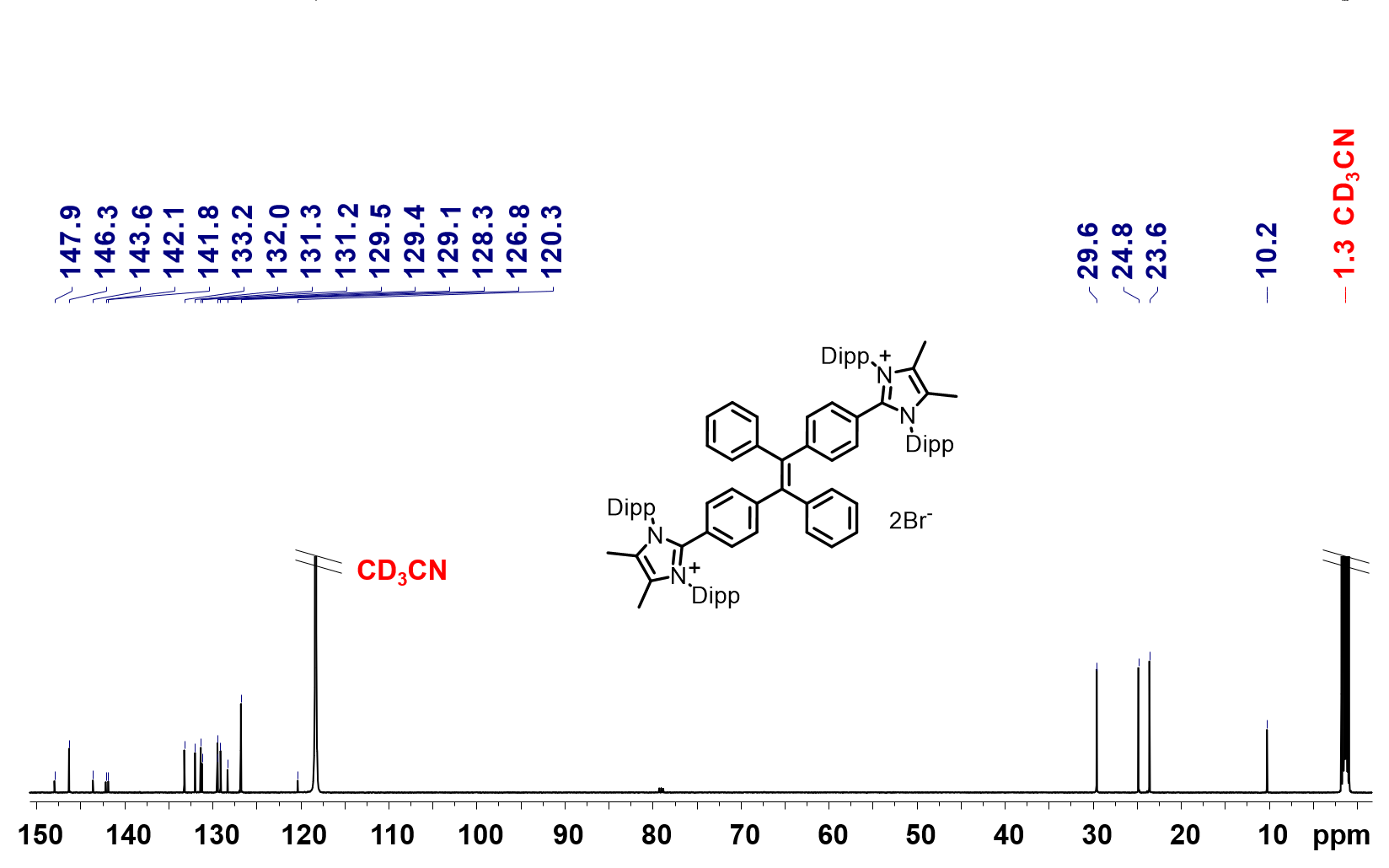


**Figure S2**. ^13^C{^1^H} NMR spectrum (150 MHz, CD_3_CN) of **1a**.


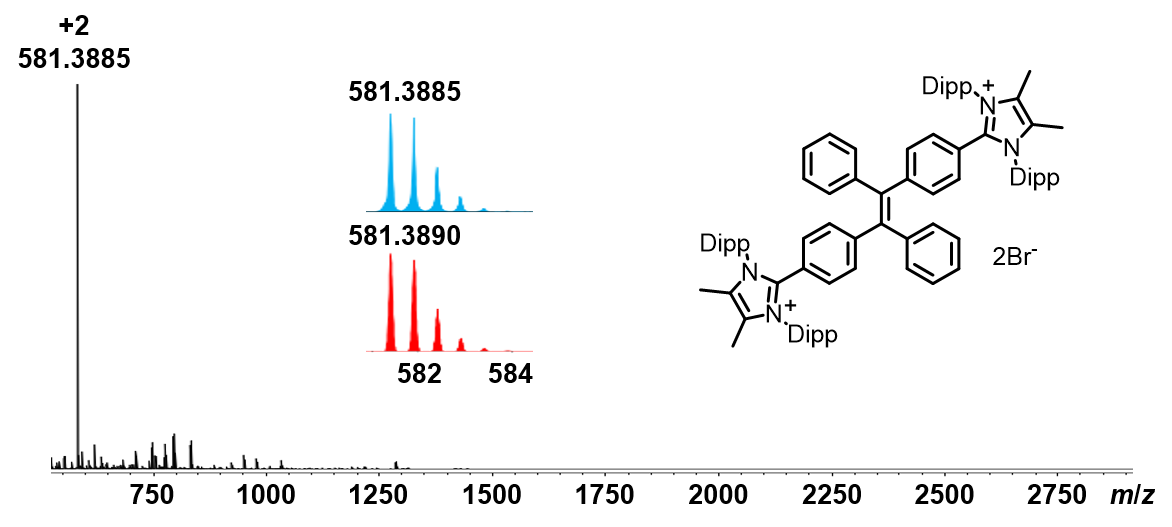


**Figure S3**. HR ESI mass spectrum of **1a** with isotope distribution for selected peaks (experimental, top and calculated, bottom).


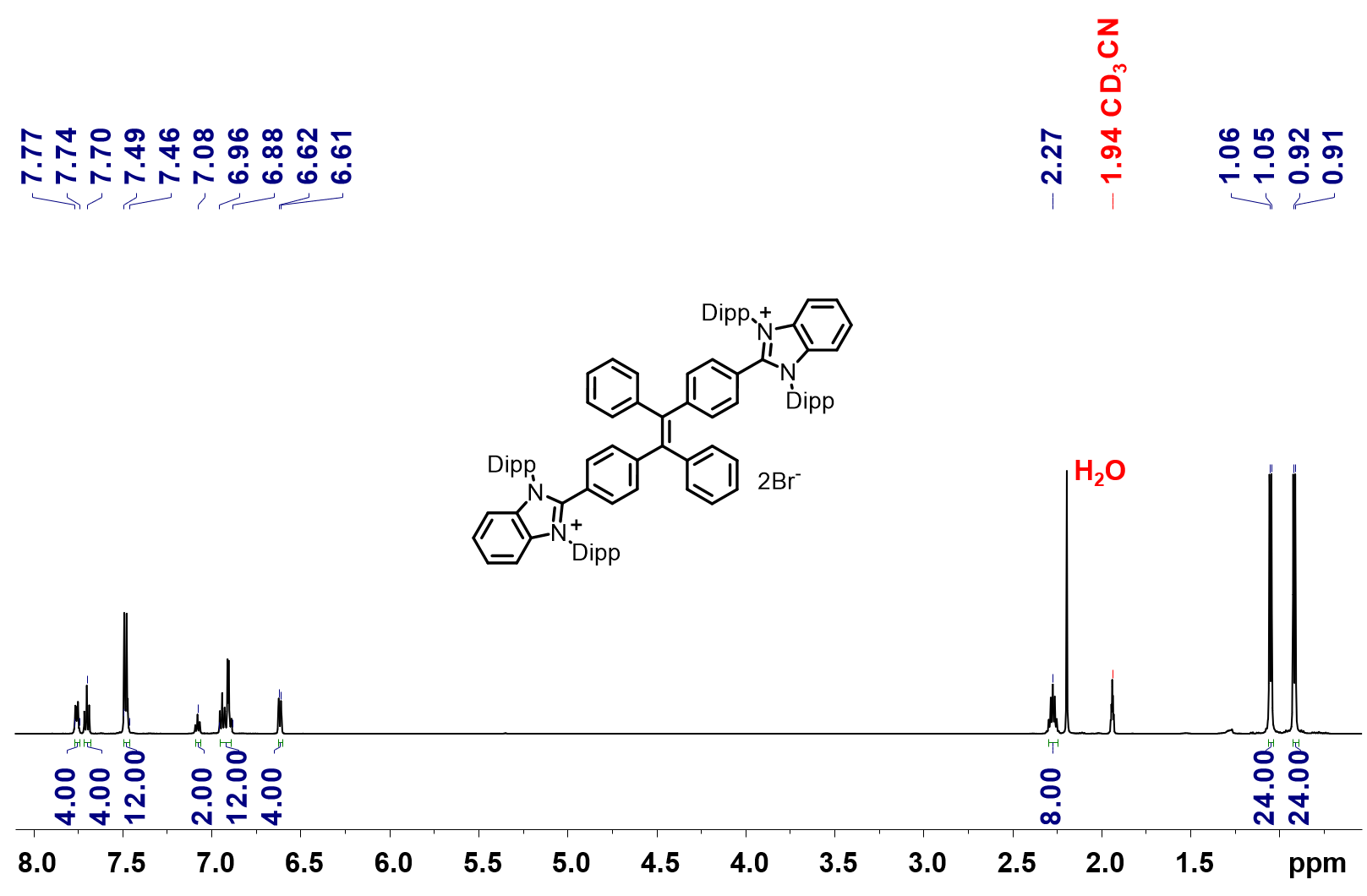


**Figure S4**. ^1^H NMR spectrum (600 MHz, CD_3_CN) of **1b**.


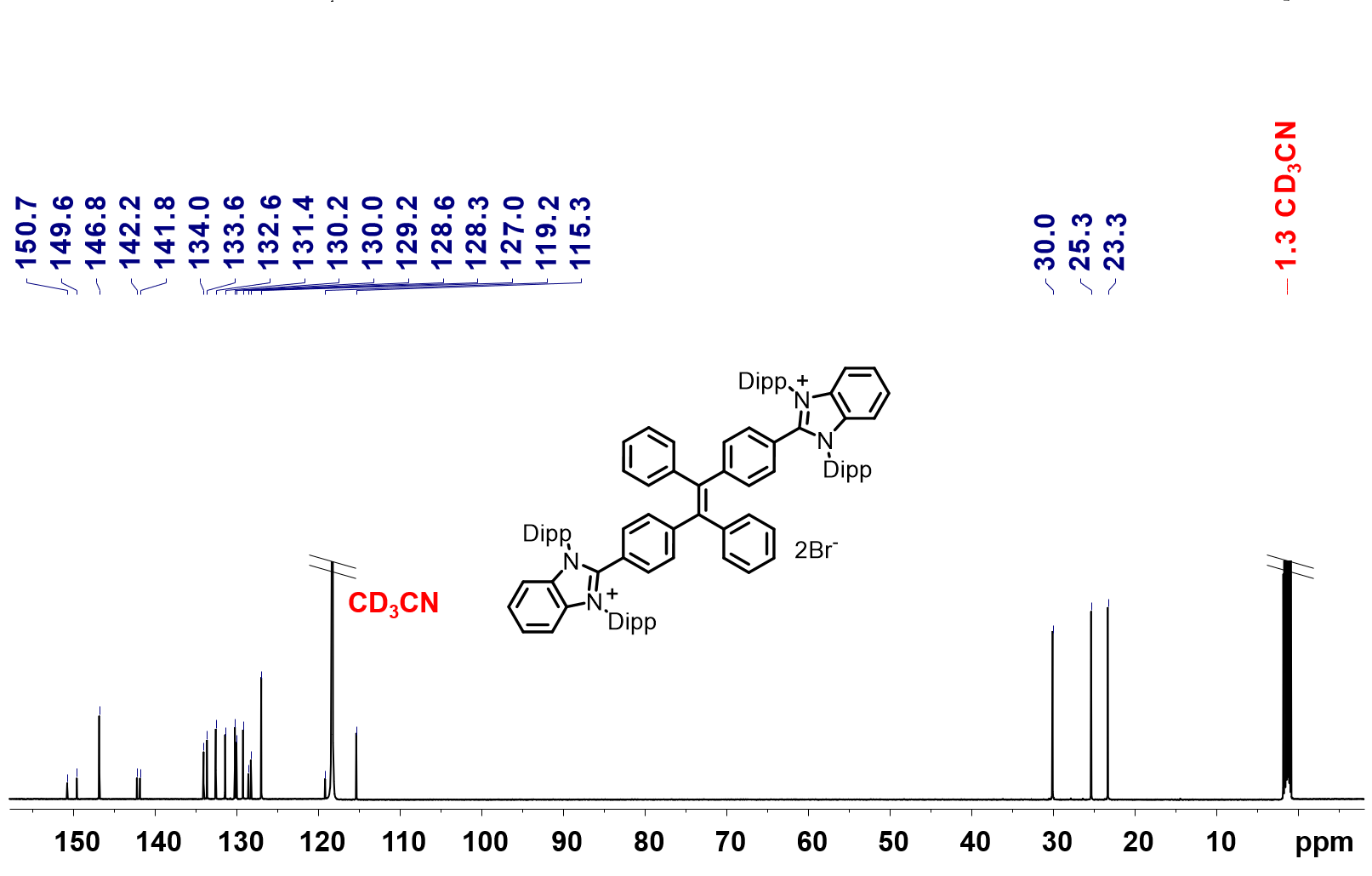


**Figure S5**. ^13^C{^1^H} NMR spectrum (150 MHz, CD_3_CN) of **1b**.


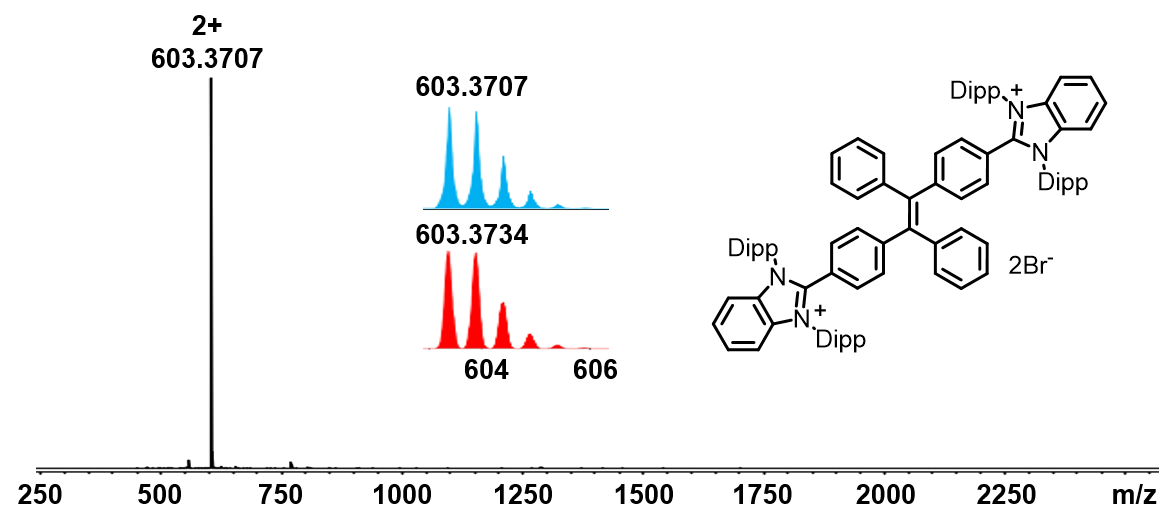


**Figure S6**. HR ESI mass spectrum of **1b** with isotope distribution for selected peaks (experimental, top and calculated, bottom).


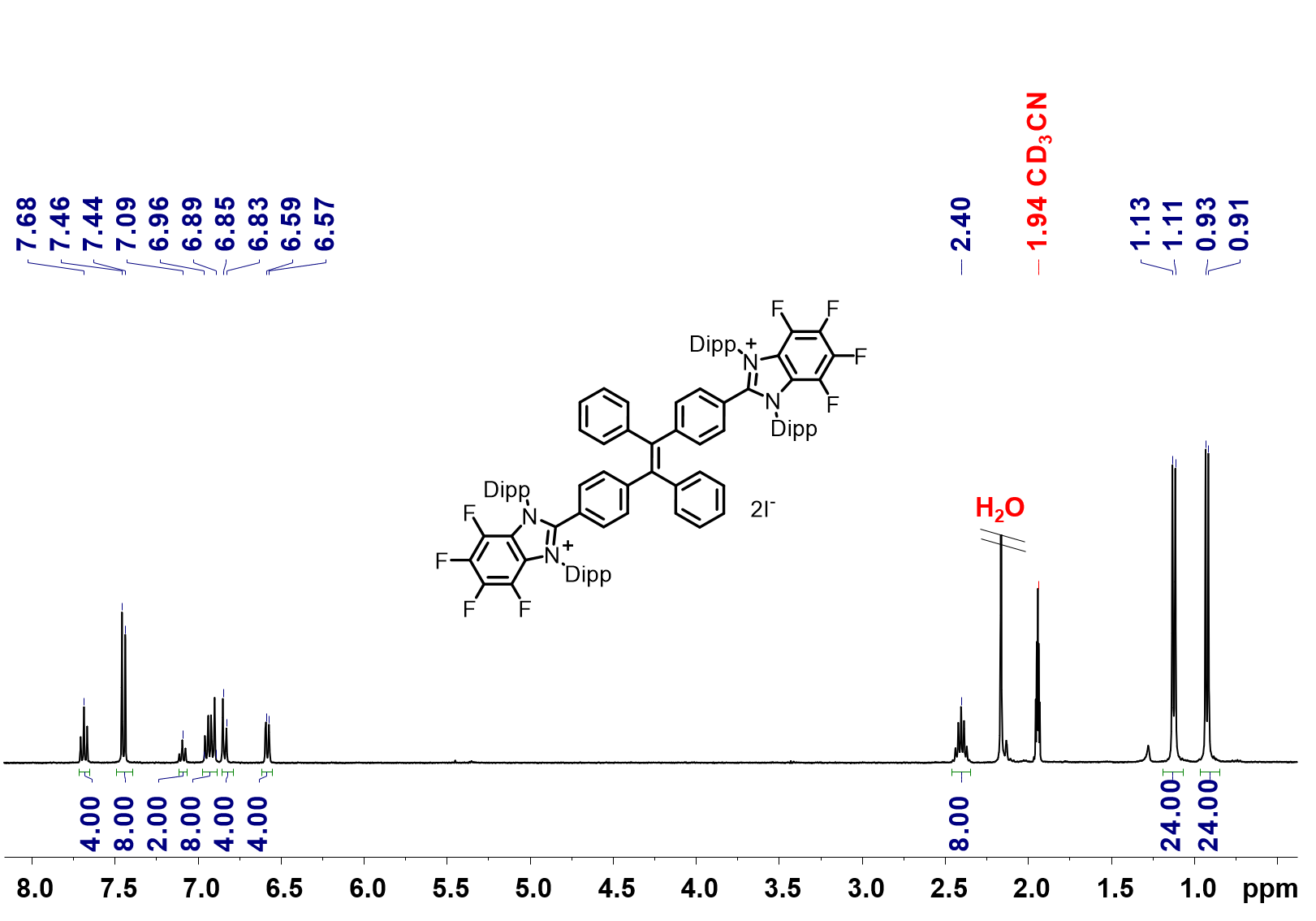


**Figure S7**. ^1^H NMR spectrum (600 MHz, CD_3_CN) of **1c**.


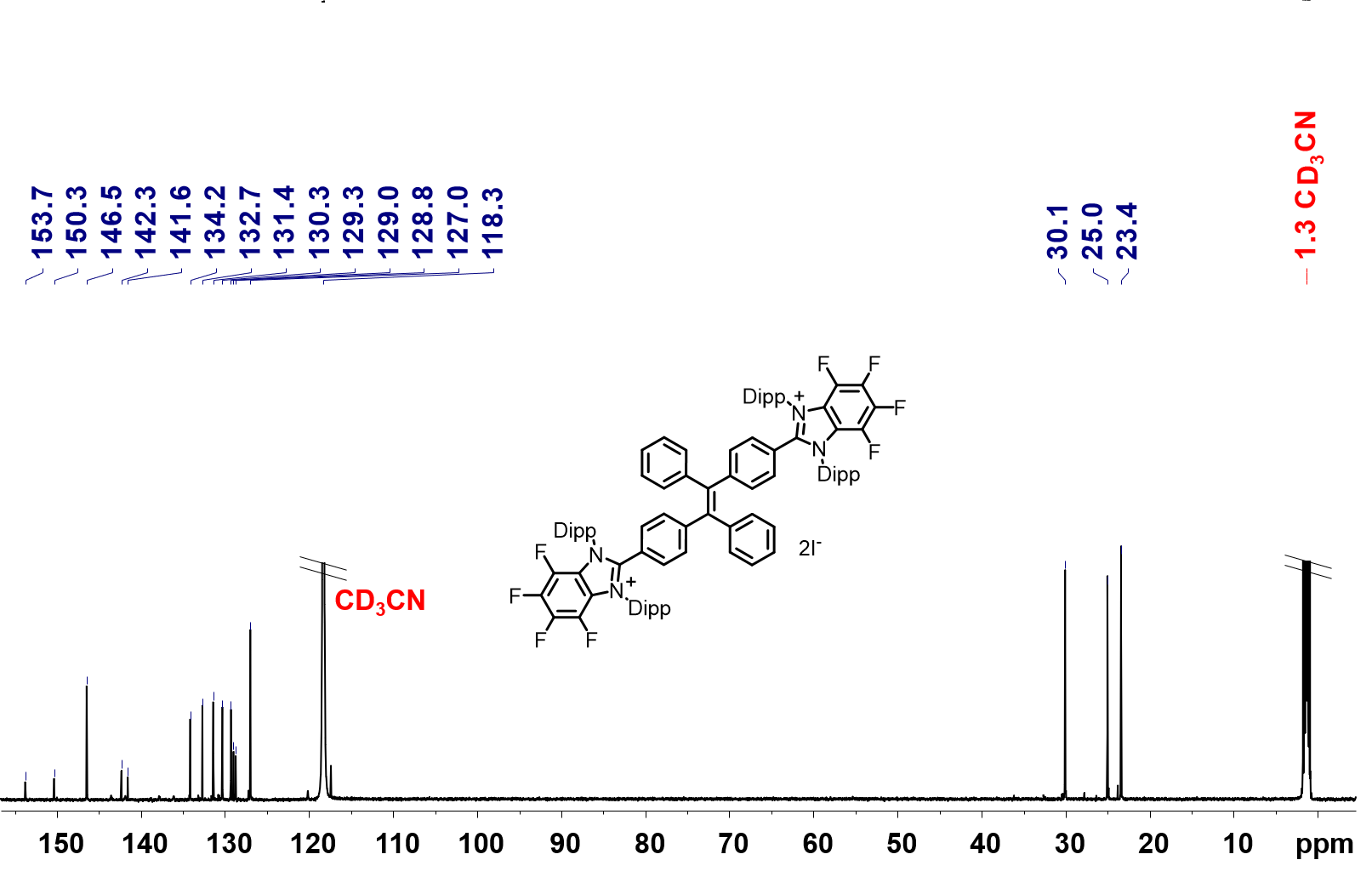


**Figure S8**. ^13^C{^1^H} NMR spectrum (150 MHz, CD_3_CN) of **1c**.


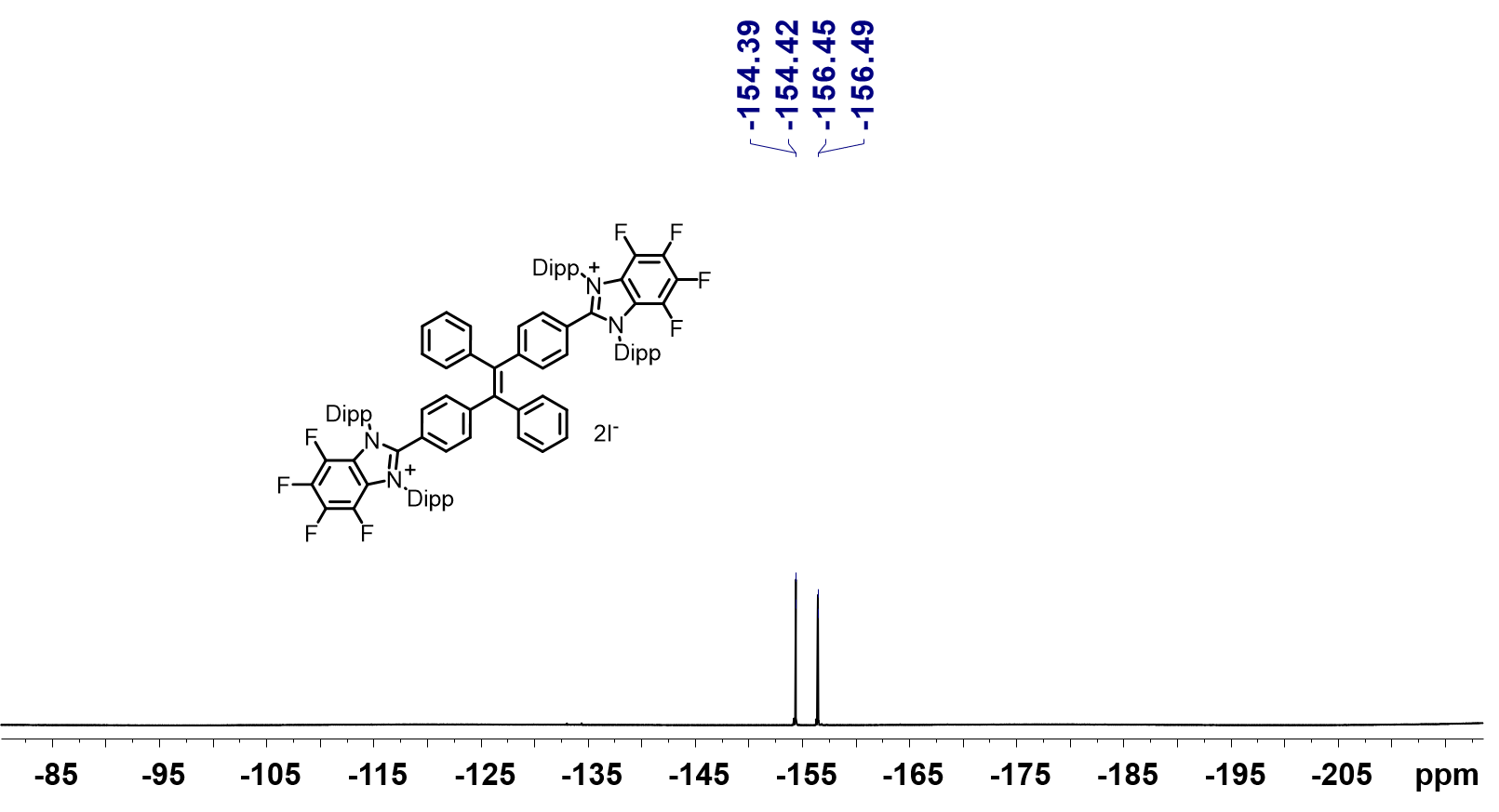


**Figure S9**. ^19^F NMR spectrum (564 MHz, CD_3_CN) of **1c**.


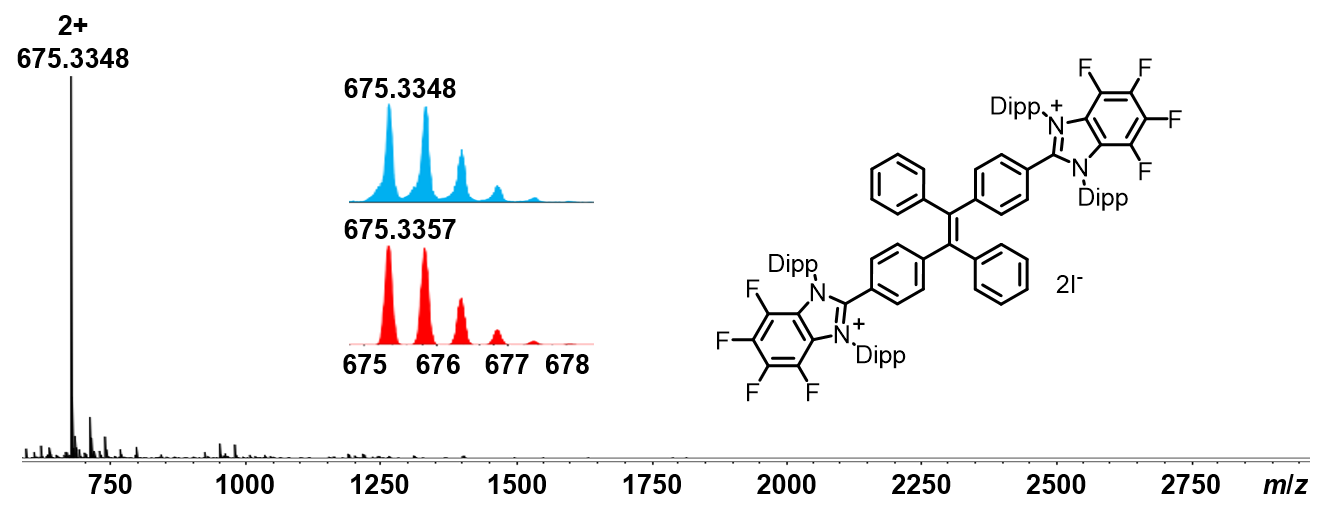


**Figure S10**. HR ESI mass spectrum of **1c** with isotope distribution for selected peaks (experimental, top and calculated, bottom).


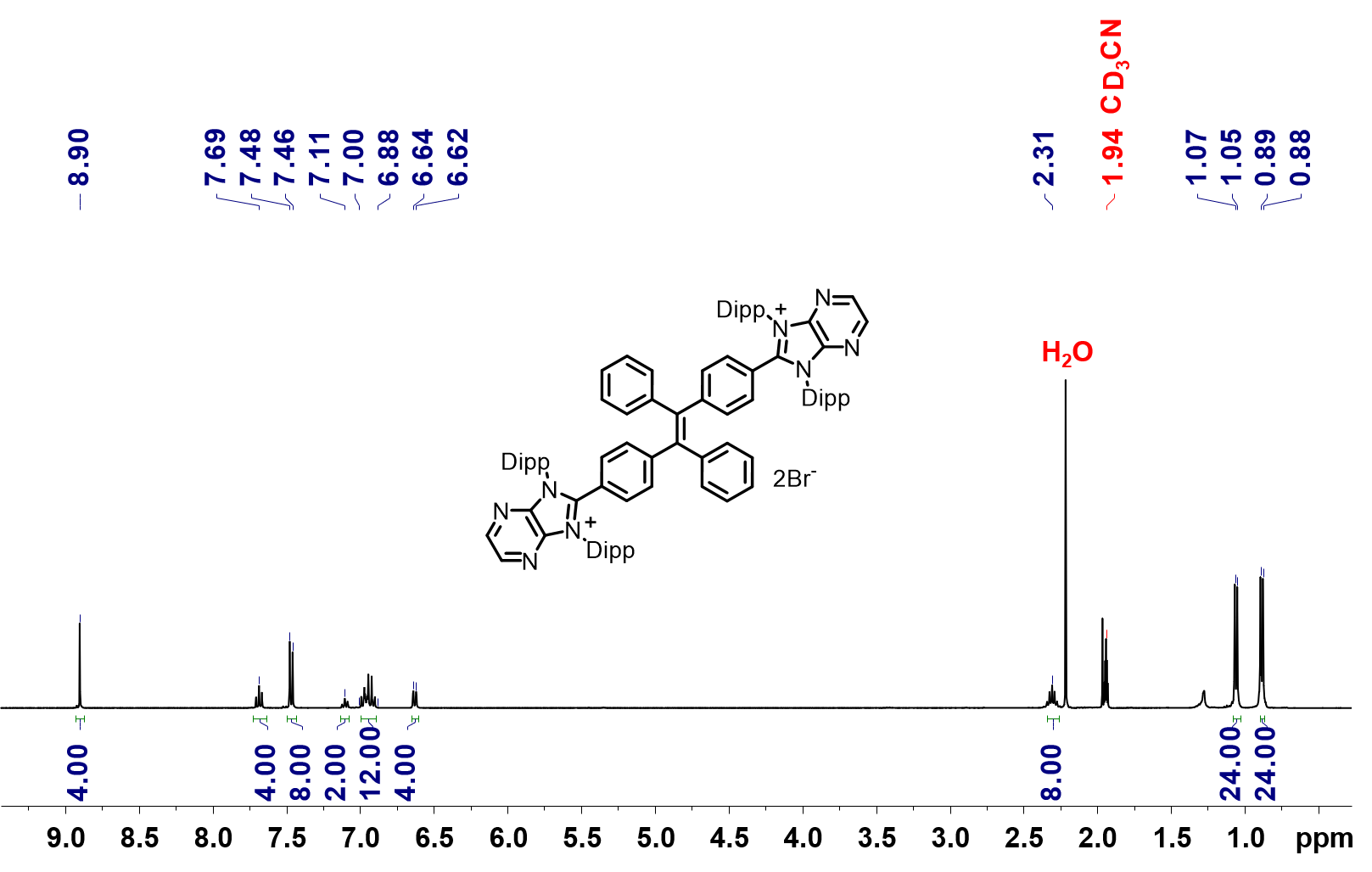


**Figure S11**. ^1^H NMR spectrum (400 MHz, CD_3_CN) of **1d.**


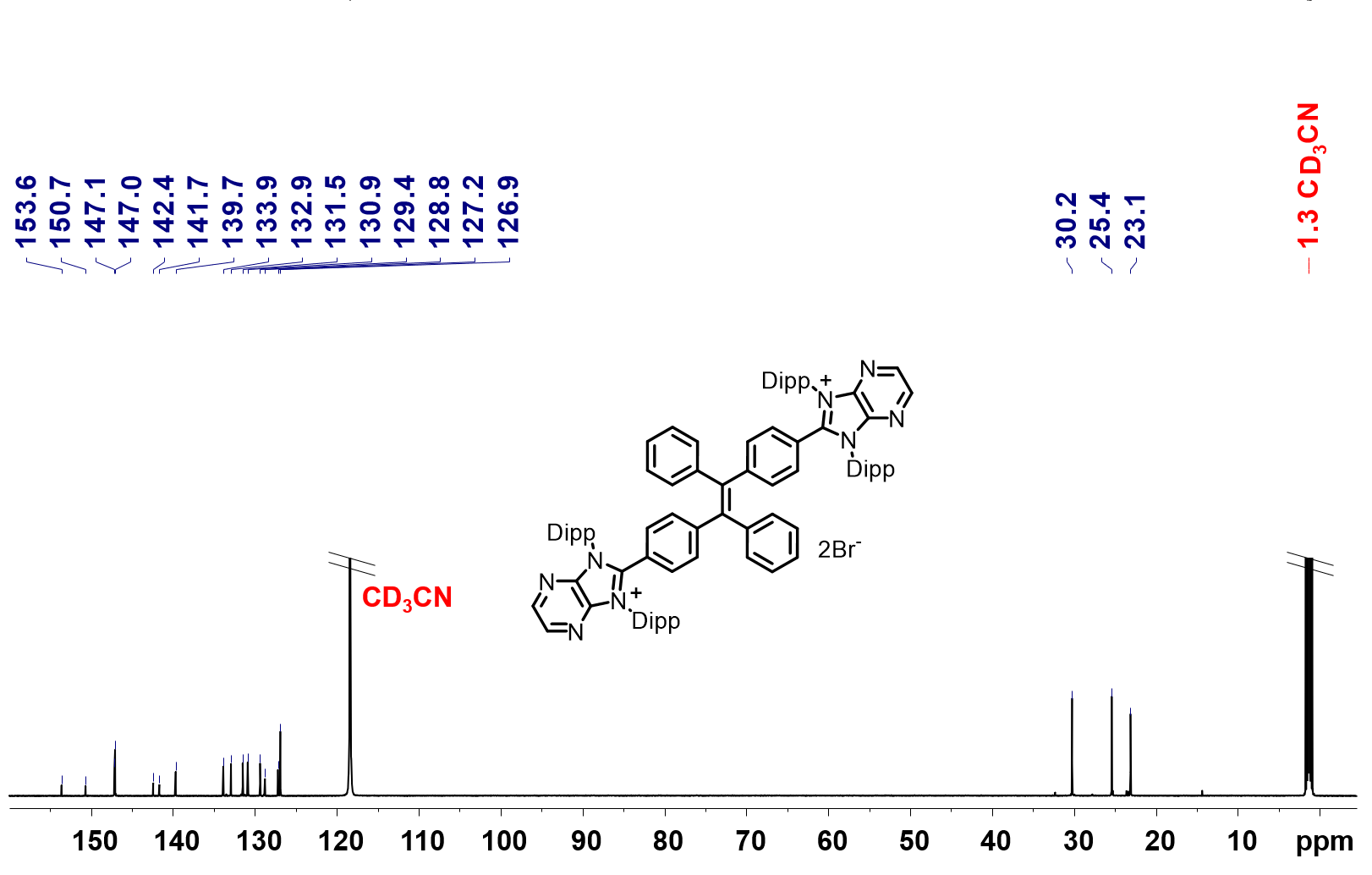


**Figure S12**. ^13^C{^1^H} NMR spectrum (150 MHz, CD_3_CN) of **1d**.


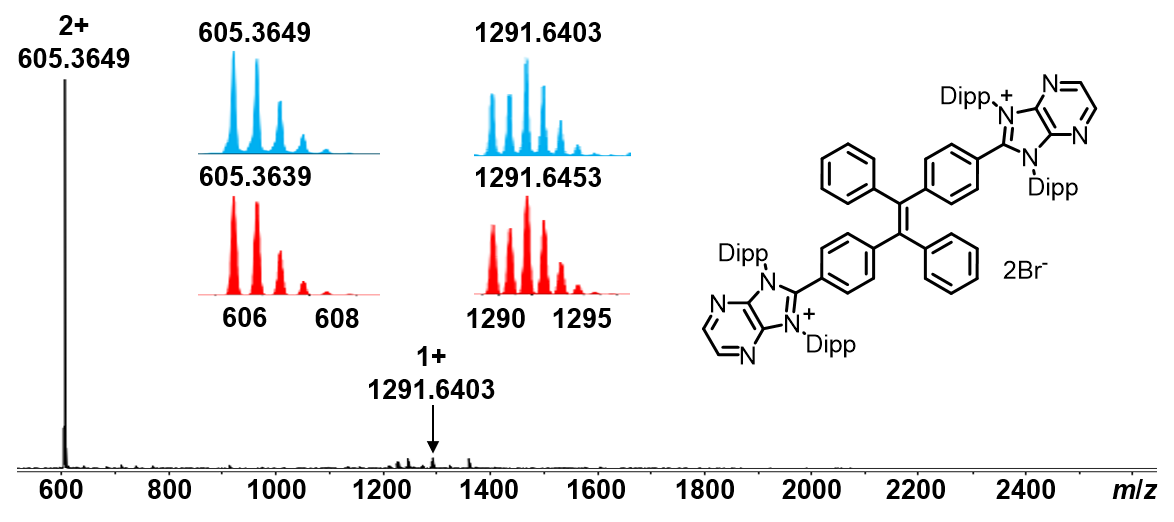


**Figure S13**. HR ESI mass spectrum of **1d** with isotope distribution for selected peaks (experimental, top and calculated, bottom).


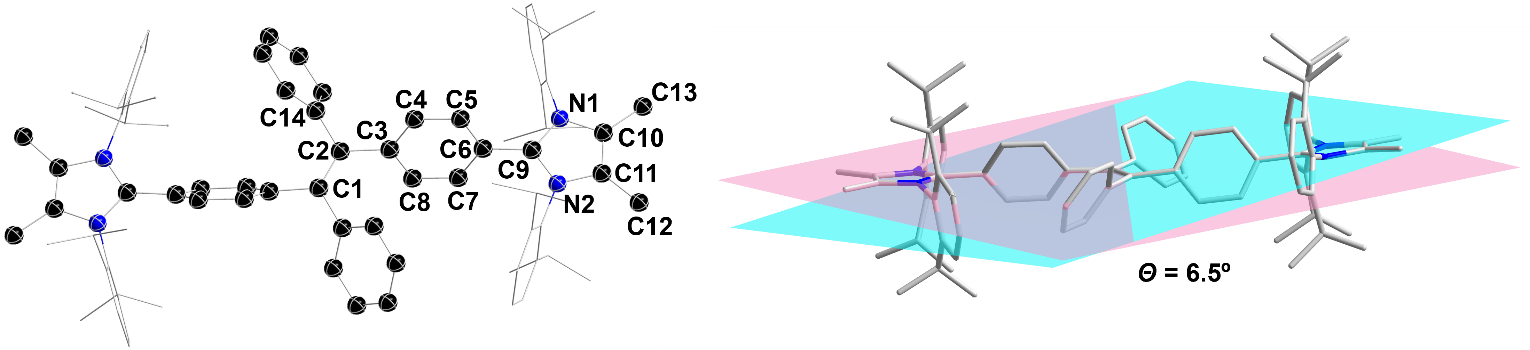


**Figure S14.** Molecular structure of **1a**^2+^ (hydrogen atoms, counter anions and solvates have been omitted for clarity). Selected bond lengths [Å], bond angles [°] and dihedral angle (*θ*) [°]: C1–C2 1.349(4), C2–C3 1.494(3), C6–C9 1.472(3), C3–C4 1.391(4), C4–C5 1.382(4), C5–C6 1.389(4), C6–C7 1.385(4), C7–C8 1.388(4), C3–C8 1.381(4), C9–N1 1.341(3), C9–N2 1.340(3), C10–N1 1.392(4), C11–N2 1.395(3), C10–C11 1.349(4), C10–C13 1.488(4), C11–C12 1.482(4), ∠C3-C2-C14 115.4(2), ∠N1-C9-N2 107.2(2), *θ* 6.5.


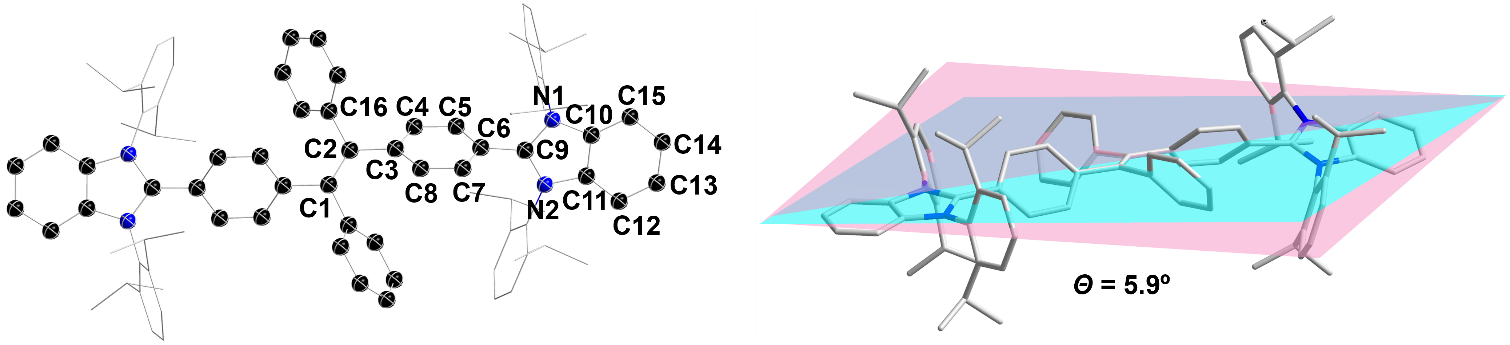


**Figure S15.** Molecular structure of **1b**^2+^ (hydrogen atoms, counter anions and solvates have been omitted for clarity). Selected bond lengths [Å], bond angles [°] and dihedral angle (*θ*) [°]: C1–C2 1.358(4), C2–C3 1.494(4), C6–C9 1.467(4), C3–C4 1.398(4), C4–C5 1.378(4), C5–C6 1.380(4), C6–C7 1.393(4), C7–C8 1.383(4), C3–C8 1.392(4), C9–N1 1.342(3), C9–N2 1.342(3), C10–N1 1.399(3), C11–N2 1.395(3), C10–C11 1.386(4), C11–C12 1.389(4), C12–C13 1.369(4), C13–C14 1.395(5), C14–C15 1.375(4), C10–C15 1.398(4), ∠C3-C2-C16 114.6(2), ∠N1-C9-N2 109.0(2) , *θ* 5.9.


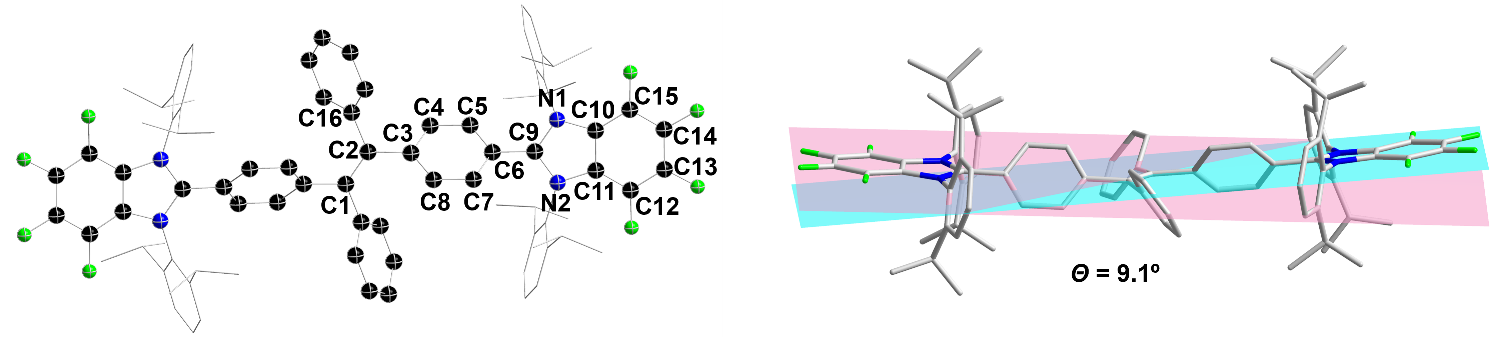


**Figure S16.** Molecular structure of **1c**^2+^ (hydrogen atoms, counter anions and solvates have been omitted for clarity). Selected bond lengths [Å], bond angles [°] and dihedral angle (*θ*) [°]: C1–C2 1.336(9), C2–C3 1.514(8), C6–C9 1.474(7), C3–C4 1.370(8), C4–C5 1.382(7), C5–C6 1.386(7), C6–C7 1.373(8), C7–C8 1.406(8), C3–C8 1.361(8), C9–N1 1.342(6), C9–N2 1.358(6), C10–N1 1.405(5), C11–N2 1.393(6), C10–C11 1.389(6), C11–C12 1.390(6), C12–C13 1.347(7), C13–C14 1.399(7), C14–C15 1.363(7), C10–C15 1.373(7), ∠C3-C2-C16 115.4(5), ∠N1-C9-N2 109.0(4) , *θ* 9.1.


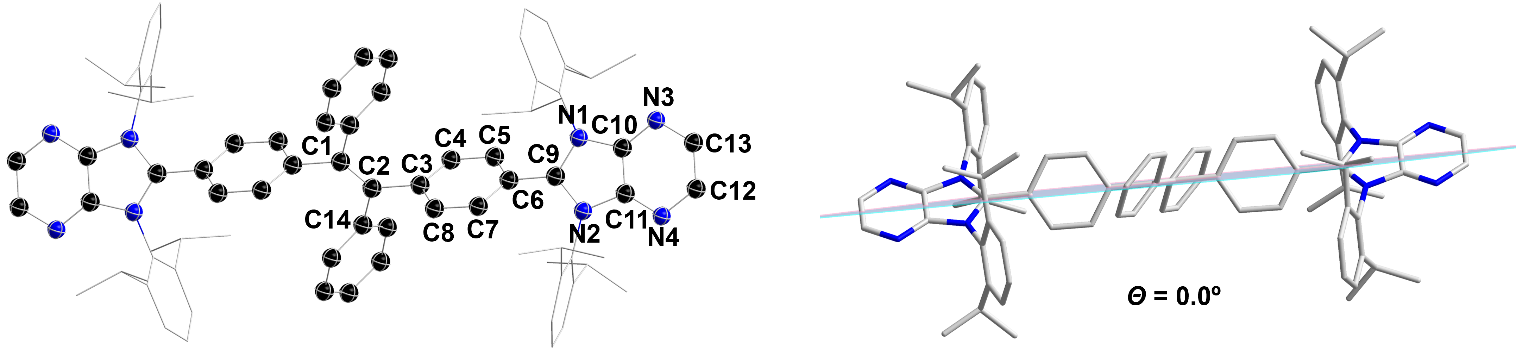


**Figure S17.** Molecular structure of **1d**^2+^ (hydrogen atoms, counter anions and solvates have been omitted for clarity). Selected bond lengths [Å], bond angles [°] and dihedral angle (*θ*) [°]: C1–C2 1.334(6), C2–C3 1.504(3), C6–C9 1.462(3), C3–C4 1.383(4), C4–C5 1.376(4), C5–C6 1.396(4), C6–C7 1.386(4), C7–C8 1.379(4), C3–C8 1.395(4), C9–N1 1.349(3), C9–N2 1.358(3), C10–N1 1.389(3), C11–N2 1.395(3), C10–C11 1.379(4), C10–N3 1.326(3), N3–C13 1.348(4), C12–C13 1.387(5), C12–N4 1.334(4), N4–C11 1.323(3), ∠C3-C2-C14 114.2(2), ∠N1-C9-N2 109.0(2) , *θ* 0.0.


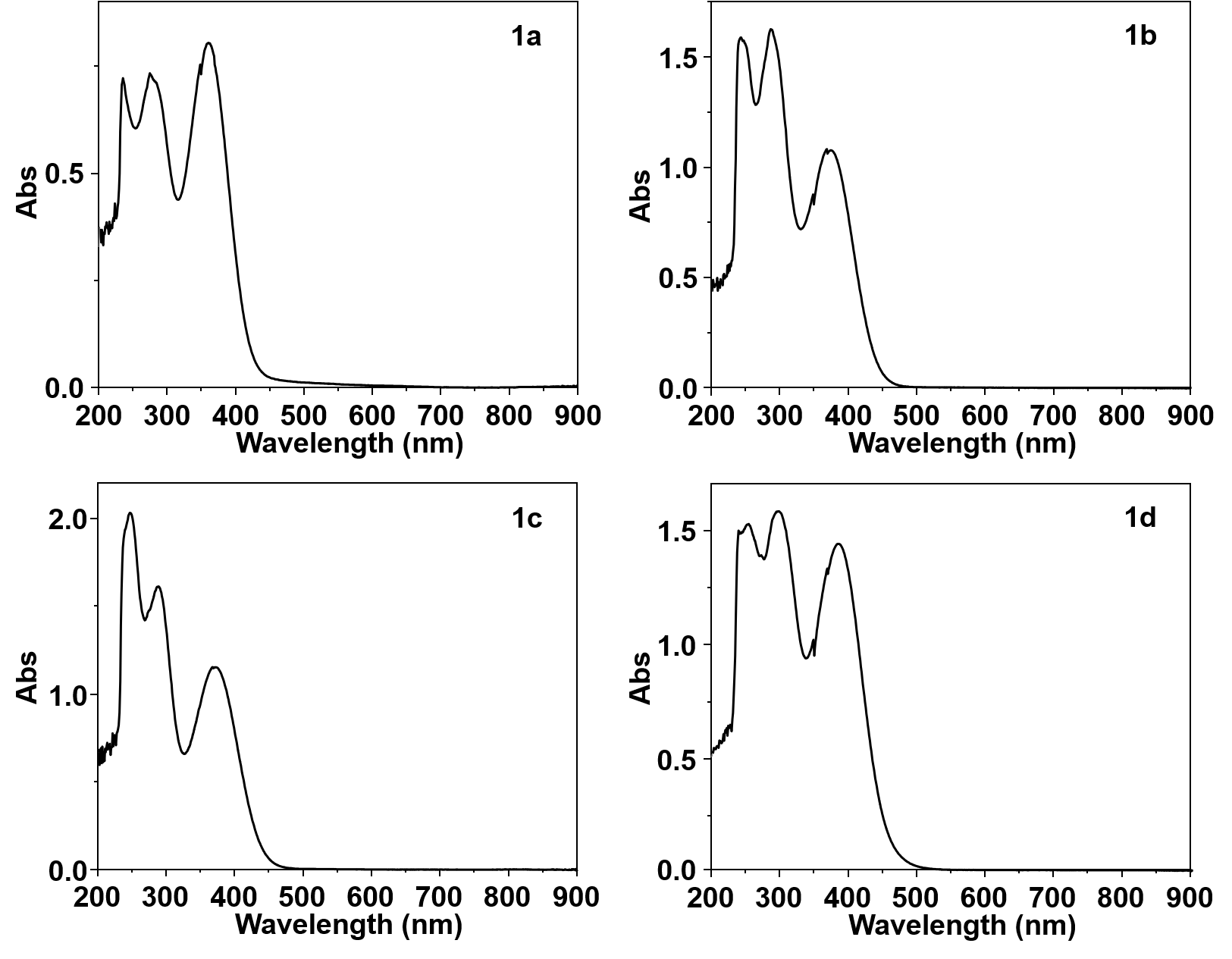


**Figure S18**. UV-vis spectra of **1a**−**d** in THF (*T* = 298 K, *c* = 10^−4^ M).

**Table S1**. UV-vis data for **1a**–**d**.

| Sample*^a^* | *λ*_max_/nm | *λ*_onset_/nm |
| --- | --- | --- |
| **1a** | 360, 274, 235 | 419 |
| **1b** | 374, 286, 246 | 441 |
| **1c** | 372, 289, 247 | 441 |
| **1d** | 384, 298, 254 | 456 |

*^a^*Solution measurements were performed using 10^-4^ M THF solutions (*T* = 298 K).


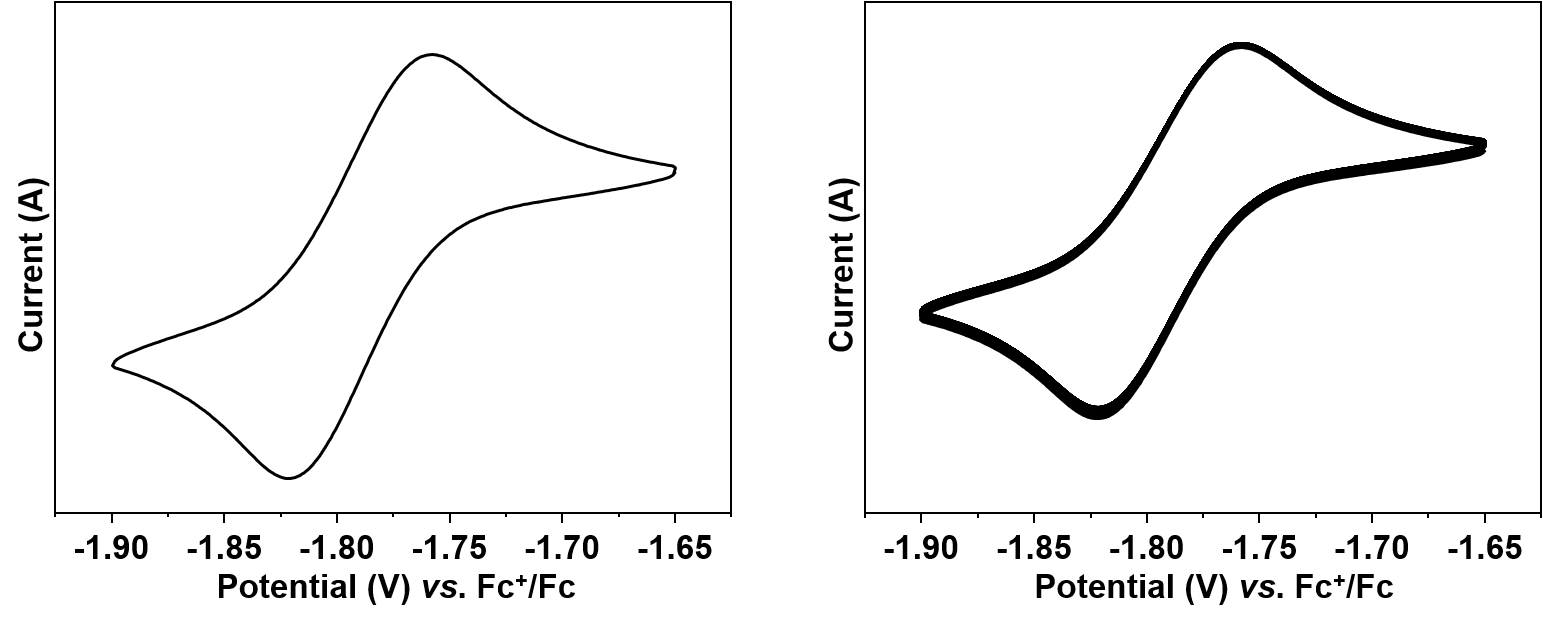


**Figure S19**. Cyclic voltammograms (left) and multicycle (100 cycles) cyclic voltammograms (right) curves of **1a** in CH_3_CN with 0.1 M *n*Bu_4_NPF_6_ at a scan rate of 100 mV·s^−1^.


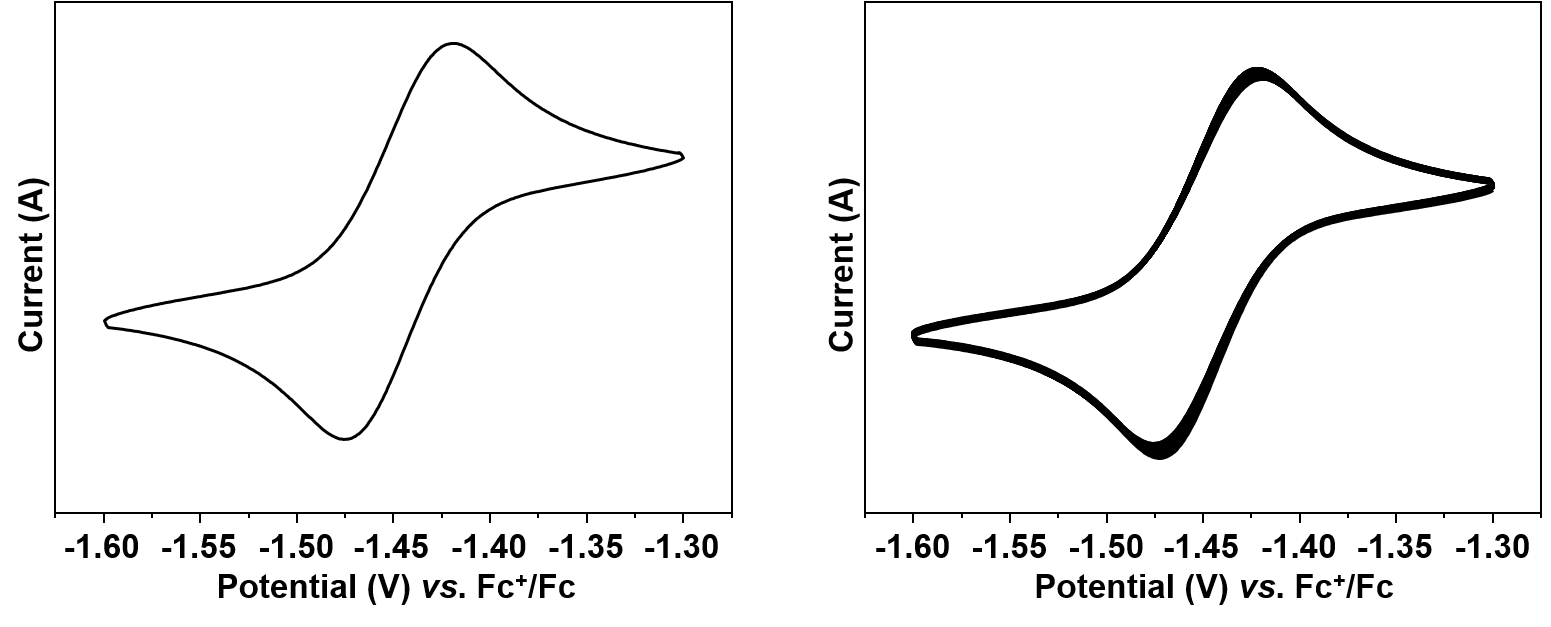


**Figure S20**. Cyclic voltammograms (left) and multicycle (100 cycles) cyclic voltammograms (right) curves of **1b** in CH_3_CN with 0.1 M *n*Bu_4_NPF_6_ at a scan rate of 100 mV·s^−1^.


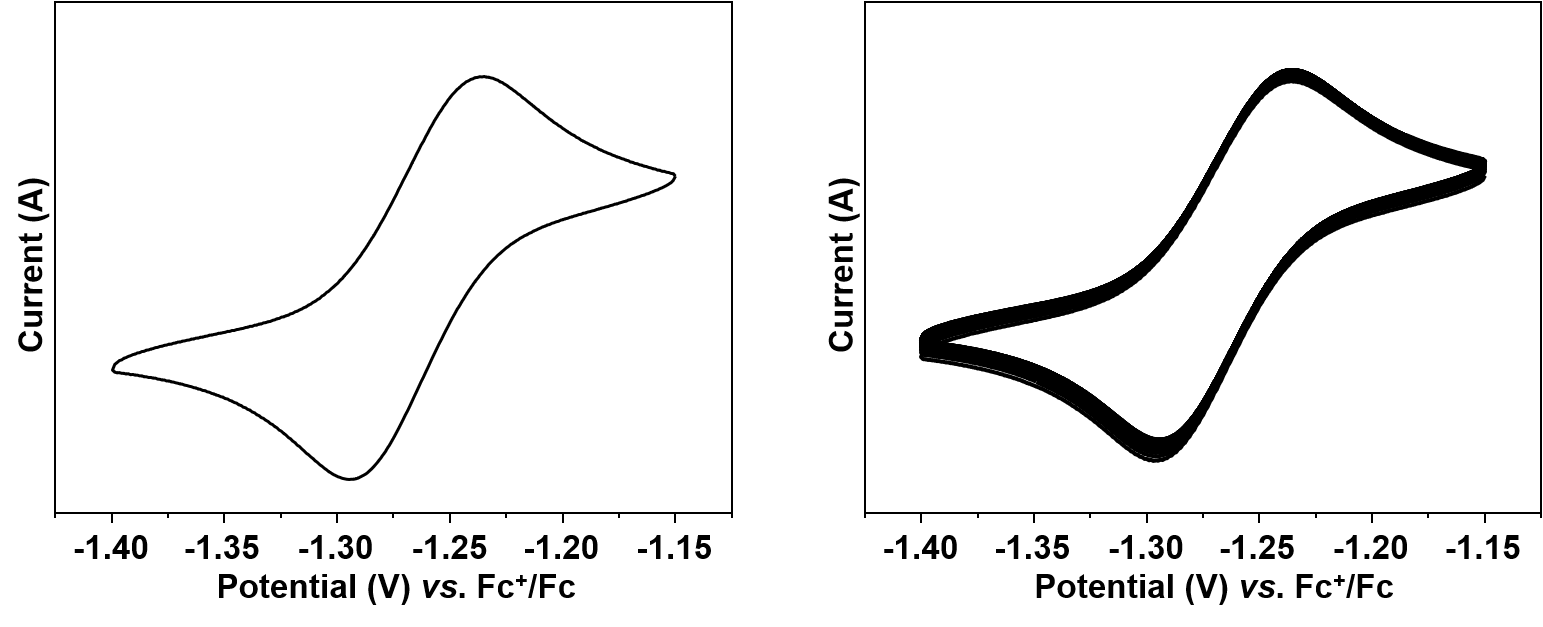


**Figure S21**. Cyclic voltammograms (left) and multicycle (100 cycles) cyclic voltammograms (right) curves of **1c** in CH_3_CN with 0.1 M *n*Bu_4_NPF_6_ at a scan rate of 100 mV·s^−1^.


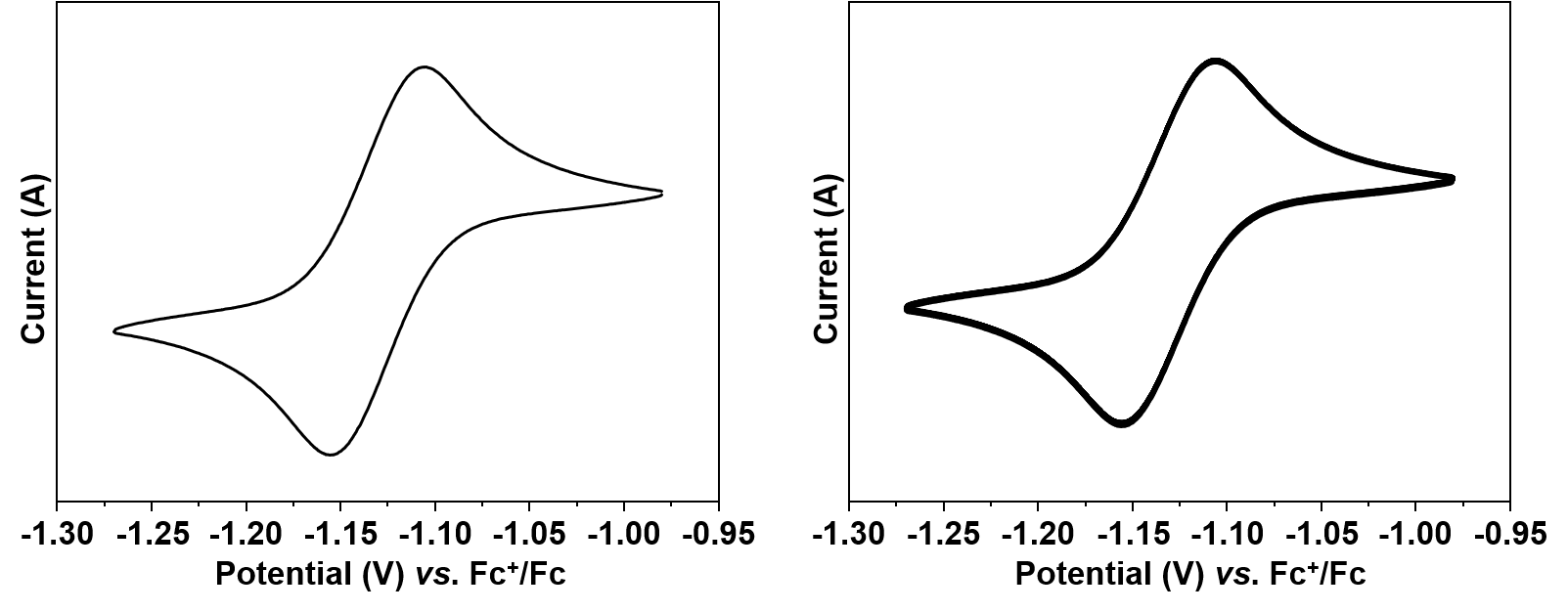


**Figure S22**. Cyclic voltammograms (left) and multicycle (100 cycles) cyclic voltammograms (right) curves of **1d** in CH_3_CN with 0.1 M *n*Bu_4_NPF_6_ at a scan rate of 100 mV·s^−1^.


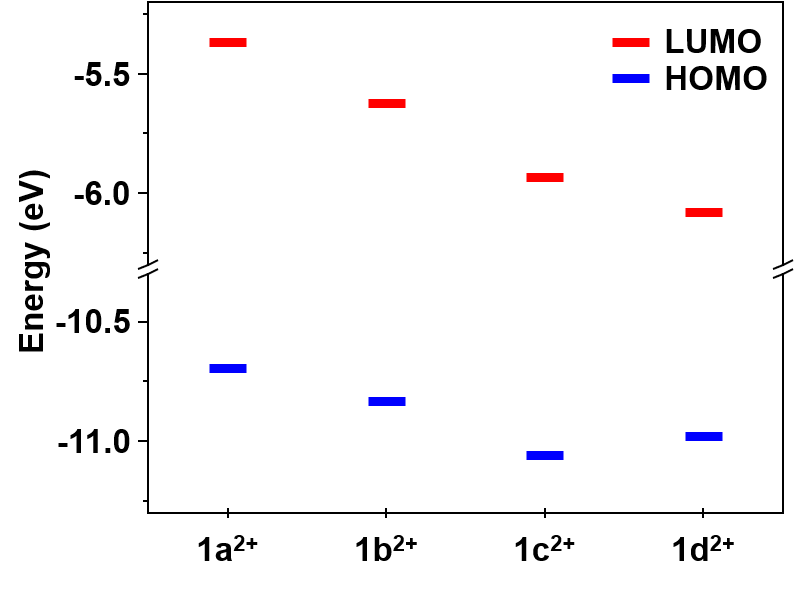


**Figure S23**. DFT calculated energies of the HOMO and LUMO frontier orbitals of **1a**^2+^−**d**^2+^, calculated at the M06-2X/6-311G** level of theory.


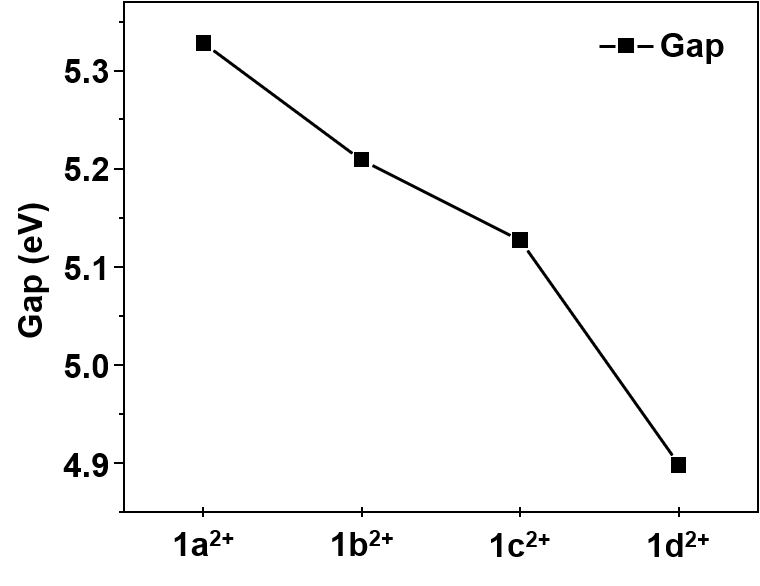


**Figure S24**. DFT calculated energies of the HOMO and LUMO frontier orbitals gap of **1a**^2+^−**d**^2+^, calculated at the M06-2X/6-311G** level of theory.

# Synthesis and characterization of 2a–d

__

**2a**: KC_8_ (54.0 mg, 0.4 mmol) was added to a THF (10.0 mL) solution of precursor **1a** (264.7 mg, 0.2 mmol) at -30 ºC. The reaction mixture was brought to room temperature and further stirred overnight. Filtration through a plug of Celite afforded a dark blue solution. The volatiles were removed, and extraction with diethyl ether and removal of volatiles afforded **2a** as a black powder. Single crystals were grown by cooling a saturated THF and *n*-pentane solution of **2a** at -30 °C. Yield: 209.5 mg, 0.180 mmol, 90%. UV-vis (THF, *λ*_max_ (nm), *ε* (M^−1^·cm^−1^)): 320 (5130.7), 480 (8662), 688 (10783.9).

__

**2b**: KC_8_ (54.0 mg, 0.4 mmol) was added to a THF (10.0 mL) solution of precursor **1b** (273.5 mg, 0.2 mmol) at -30 ºC. The reaction mixture was brought to room temperature and further stirred overnight. Filtration through a plug of Celite afforded a dark green solution. The volatiles were removed, and extraction with diethyl ether and removal of volatiles afforded **2b** as a black powder. Yield: 226.7 mg, 0.188mmol, 94%. UV-vis (THF, *λ*_max_ (nm), *ε* (M^-1^·cm^-1^)): 365 (7773.6), 471 (11032.1), 657 (7711.2).

__

**2c**: KC_8_ (54.0 mg, 0.4 mmol) was added to a THF (10.0 mL) solution of precursor **1c** (321.1 mg, 0.2 mmol) at -30 ºC. The reaction mixture was brought to room temperature and further stirred overnight. Filtration through a plug of Celite afforded a dark green solution. The volatiles were removed, and extraction with diethyl ether and removal of volatiles afforded **2c** as a black powder. Yield: 227.1 mg, 0.168 mmol, 84%. UV-vis (THF, *λ*_max_ (nm), *ε* (M^-1^·cm^-1^)): 316 (13393.3), 467 (20513.3), 655 (16443.5).

__

**2d**: KC_8_ (54.0 mg, 0.4 mmol) was added to a THF (10.0 mL) solution of precursor **1d** (274.3 mg, 0.2 mmol) at -30 ºC. The reaction mixture was brought to room temperature and further stirred overnight. Filtration through a plug of Celite afforded a dark green solution. The volatiles were removed, and extraction with diethyl ether and removal of volatiles afforded **2d** as a black powder. Single crystals were grown by cooling a saturated THF and *n*-pentane solution of **2d** at -30 °C. Yield: 215.3 mg, 0.178 mmol, 89%. UV-vis (THF, *λ*_max_ (nm), *ε* (M^-1^·cm^-1^)): 326 (17500.6), 438 (6743.3), 680 (7193.2).

Oxidation of **2a**: AgOTf (5.0 mg, 0.02 mmol) was added to a solution of **2a** (11.1 mg, 0.01 mmol) in THF (5 mL). The reaction mixture was stirred at 25 °C for 1 h to afford [**1a**][OTf]_2_. The reaction mixture was then diluted with acetonitrile for MS analysis. HRMS (ESI, positive ions): *m*/*z* = 581.3861 (calcd for [C_84_H_98_N_4_]^2+^ 581.3890).

Oxidation of **2b**: AgOTf (5.0 mg, 0.02 mmol) was added to a solution of **2b** (12.1 mg, 0.01 mmol) in THF (5 mL). The reaction mixture was stirred at 25 °C for 1 h to afford [**1b**][OTf]_2_. The reaction mixture was then diluted with acetonitrile for MS analysis. HRMS (ESI, positive ions): *m*/*z* = 603.3733 (calcd for [C_88_H_94_N_4_]^2+^ 603.3734).

Oxidation of **2c**: AgOTf (5.0 mg, 0.02 mmol) was added to a solution of **2c** (13.5 mg, 0.01 mmol) in THF (5 mL). The reaction mixture was stirred at 25 °C for 1 h to afford [**1c**][OTf]_2_. The reaction mixture was then diluted with acetonitrile for MS analysis. HRMS (ESI, positive ions): *m*/*z* = 675.3376 (calcd for [C_88_H_86_F_8_N_4_]^2+^ 675.3357).

Oxidation of **2d**: AgOTf (5.0 mg, 0.02 mmol) was added to a solution of **2d** (12.1 mg, 0.01 mmol) in THF (5 mL). The reaction mixture was stirred at 25 °C for 1 h to afford [**1d**][OTf]_2_. The reaction mixture was then diluted with acetonitrile for MS analysis. HRMS (ESI, positive ions): *m*/*z* = 605.3654 (calcd for [C_84_H_90_N_8_]^2+^ 605.3639).


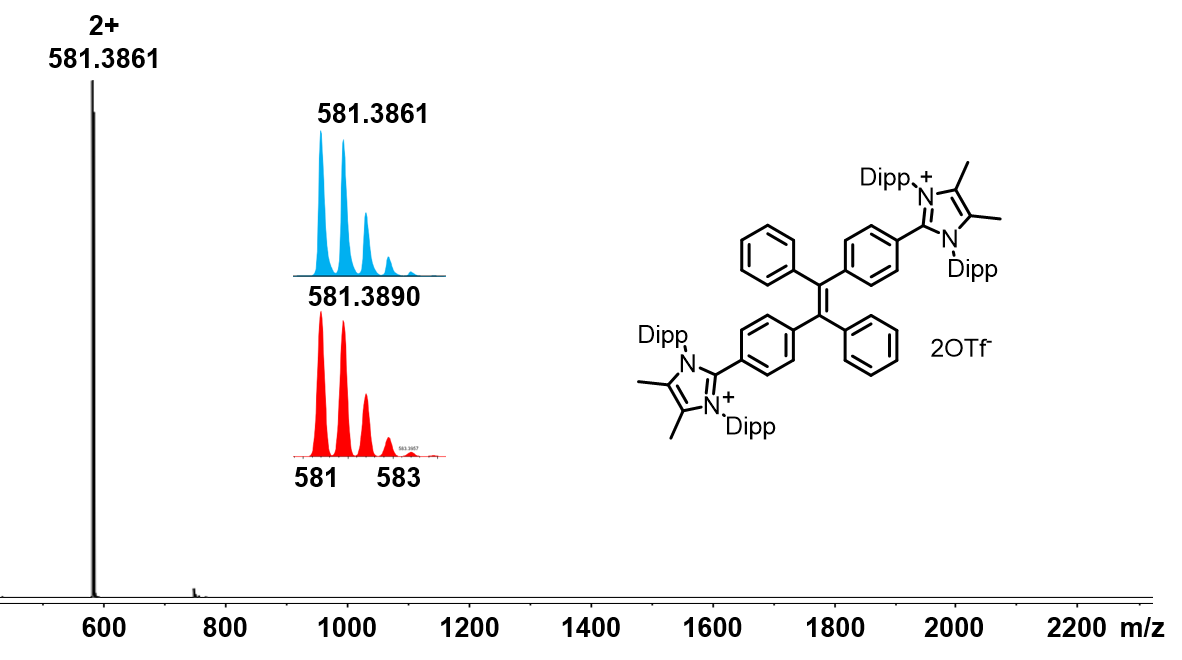


**Figure S25**. HR ESI mass spectrum of [**1a**][OTf]_2_ with the inset showing the isotope pattern of the molecular ion (experimental, top and calculated, bottom) and the structure of the product.


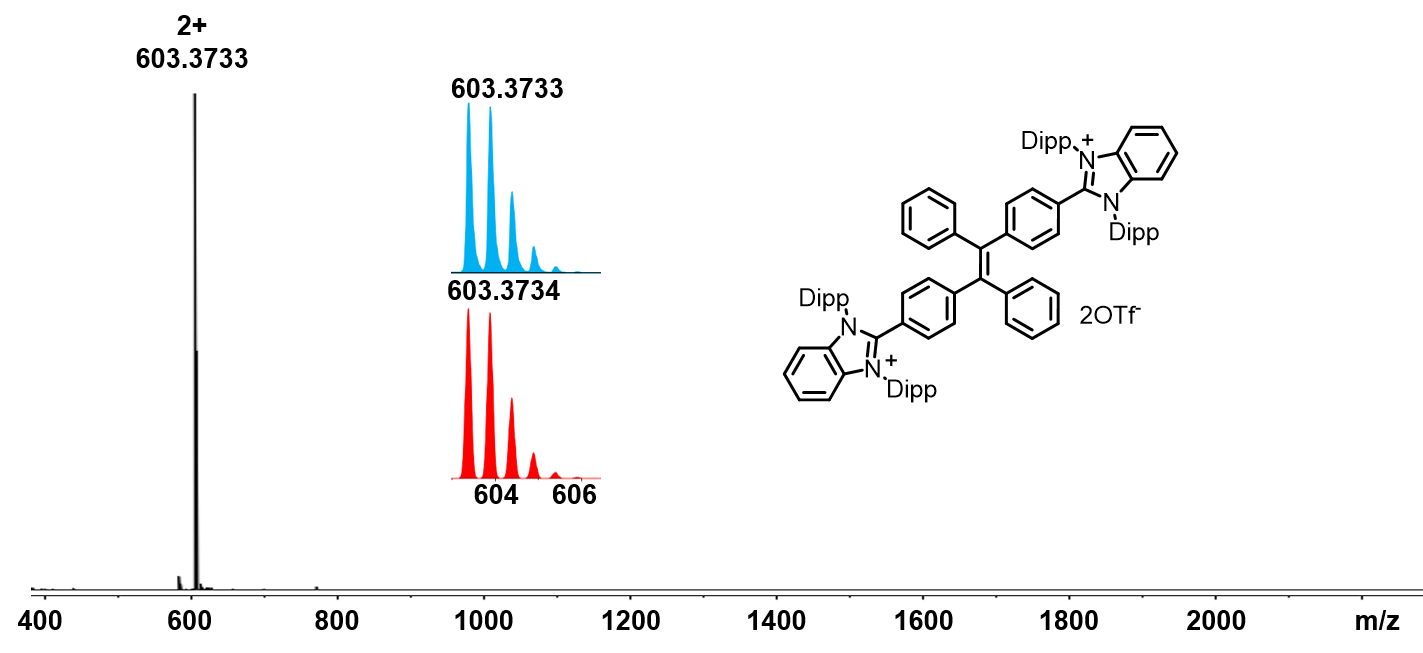


**Figure S26**. HR ESI mass spectrum of [**1b**][OTf]_2_ with the inset showing the isotope pattern of the molecular ion (experimental, top and calculated, bottom) and the structure of the product.


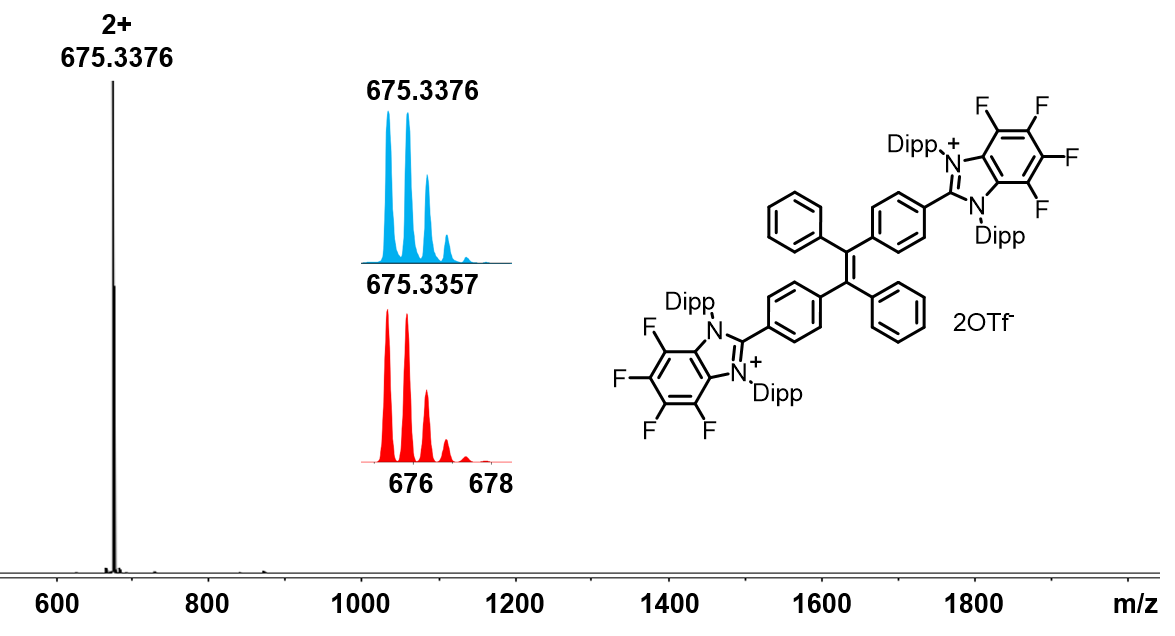


**Figure S27**. HR ESI mass spectrum of [**1c**][OTf]_2_ with the inset showing the isotope pattern of the molecular ion (experimental, top and calculated, bottom) and the structure of the product.


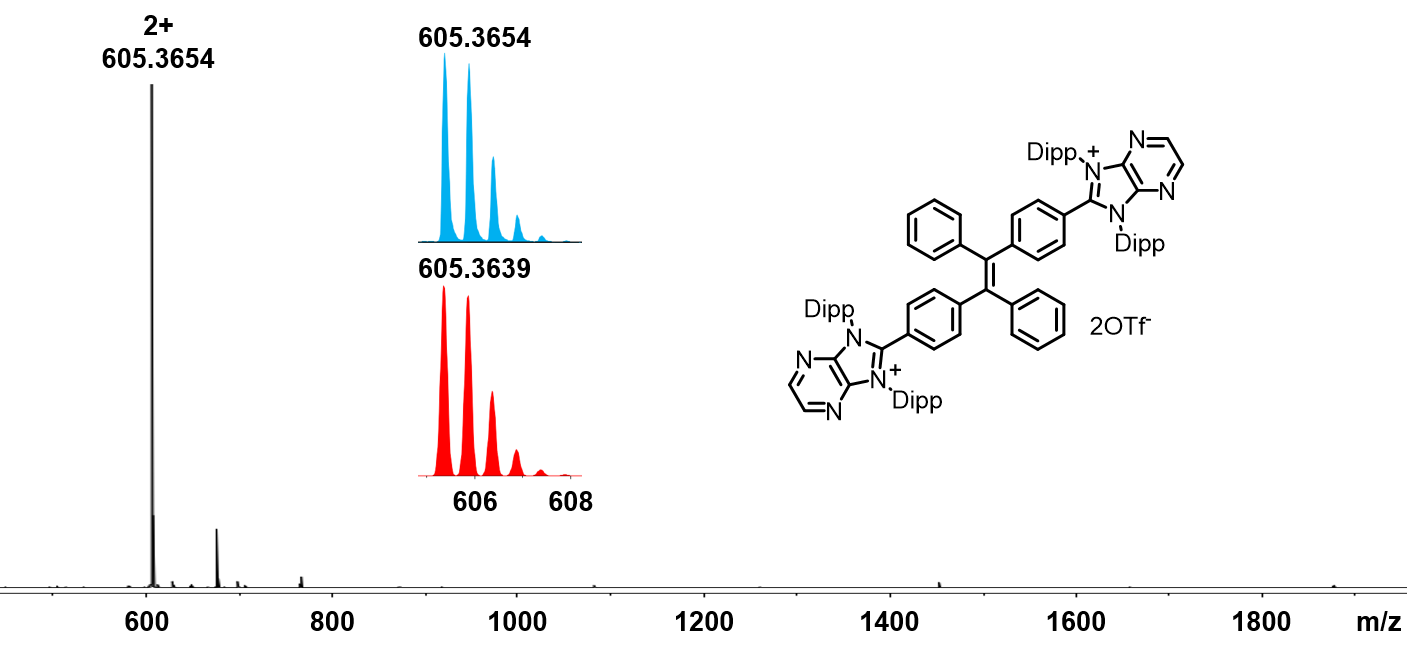


**Figure S28**. HR ESI mass spectrum of [**1d**][OTf]_2_ with the inset showing the isotope pattern of the molecular ion (experimental, top and calculated, bottom) and the structure of the product.
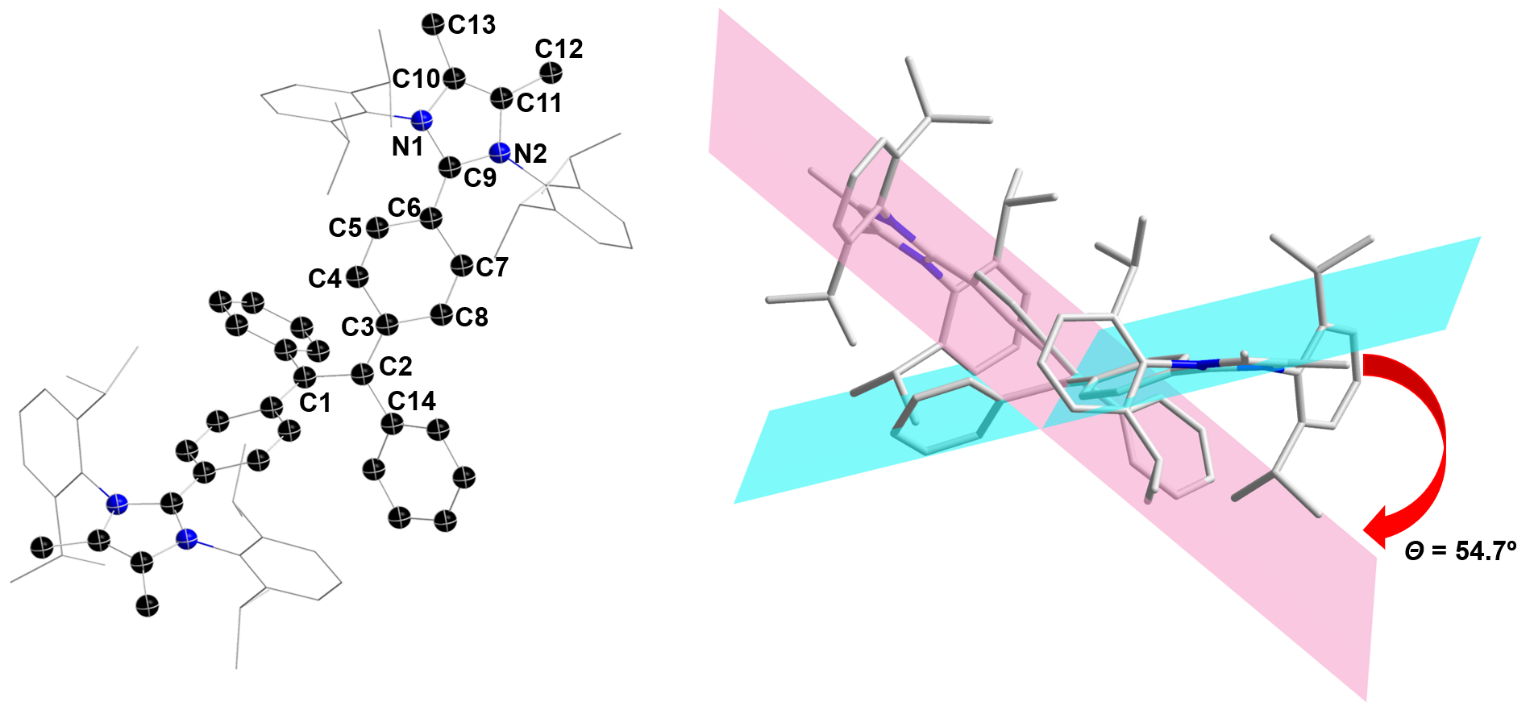


**Figure S29.** Molecular structure of **2a** (hydrogen atoms and solvates have been omitted for clarity). Selected bond lengths [Å], bond angles [°] and dihedral angle (*θ*) [°]: C1–C2 1.479(4), C2–C3 1.397(4), C6–C9 1.408(4), C3–C4 1.441(4), C4–C5 1.348(4), C5–C6 1.427(4), C6–C7 1.436(4), C7–C8 1.349(4), C3–C8 1.439(4), C9–N1 1.385(3), C9–N2 1.374(4), C10–N1 1.415(4), C11–N2 1.414(3), C10–C11 1.340(4), C10–C13 1.484(4), C11–C12 1.487(4), ∠C3-C2-C14 122.7(2), ∠N1-C9-N2 104.2(2) , *θ* 54.7.


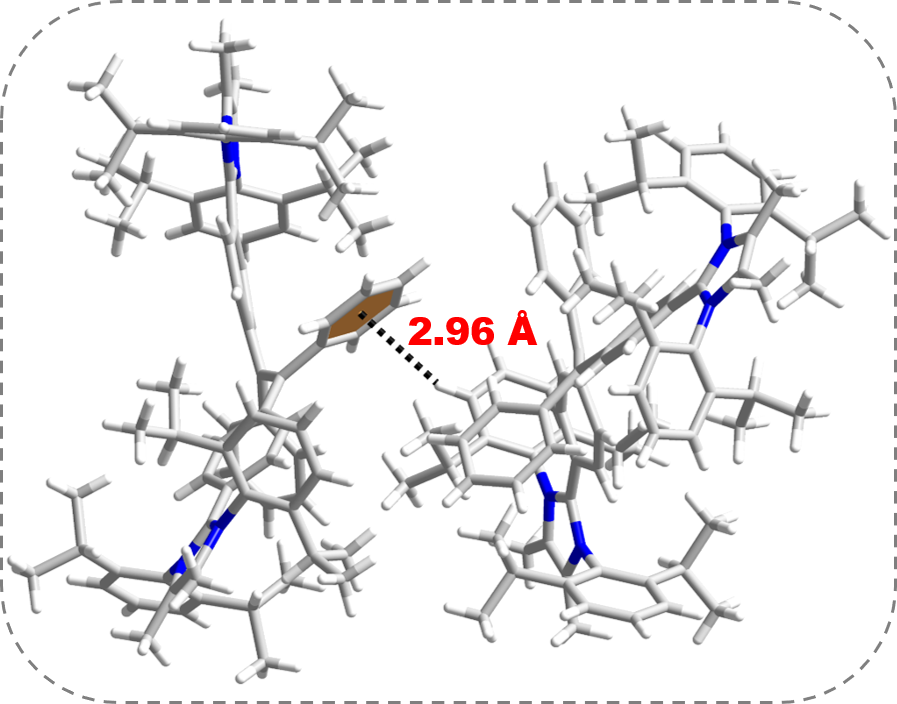


**Figure S30.** Packing of **2a** (solvates have been omitted for clarity).


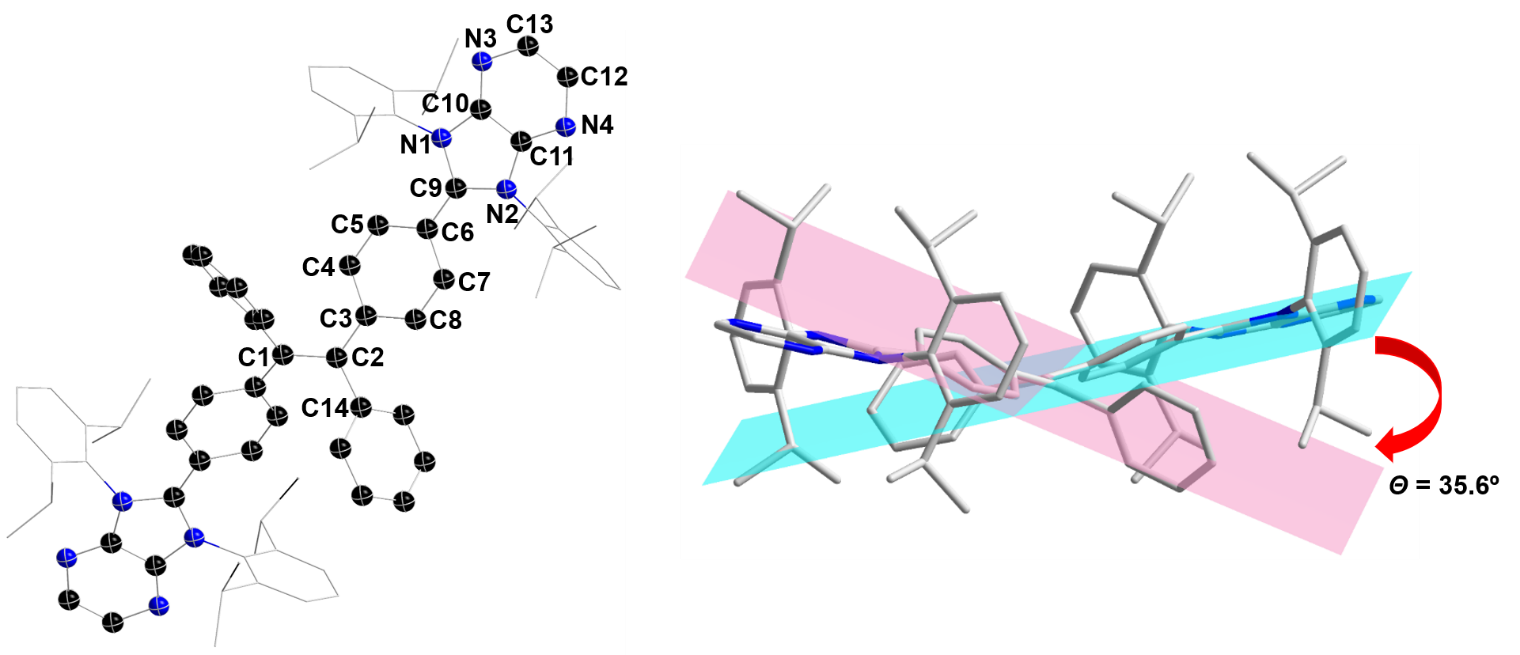


**Figure S31.** Molecular structure of **2d** (hydrogen atoms and solvates have been omitted for clarity). Selected bond lengths [Å], bond angles [°] and dihedral angle (*θ*) [°]: C1–C2 1.453(4), C2–C3 1.400(4), C6–C9 1.378(4), C3–C4 1.440(4), C4–C5 1.349(4), C5–C6 1.438(4), C6–C7 1.438(4), C7–C8 1.352(4), C3–C8 1.434(4), C9–N1 1.408(3), C9–N2 1.413(3), C10–N1 1.376(4), C11–N2 1.385(4), C10–C11 1.396(4), C10–N3 1.309(4), N3–C13 1.366(4), C12–C13 1.360(4), C12–N4 1.362(4), N4–C11 1.307(4), ∠C3-C2-C14 118.9(3), ∠N1-C9-N2 105.1(2) , *θ* 35.6.


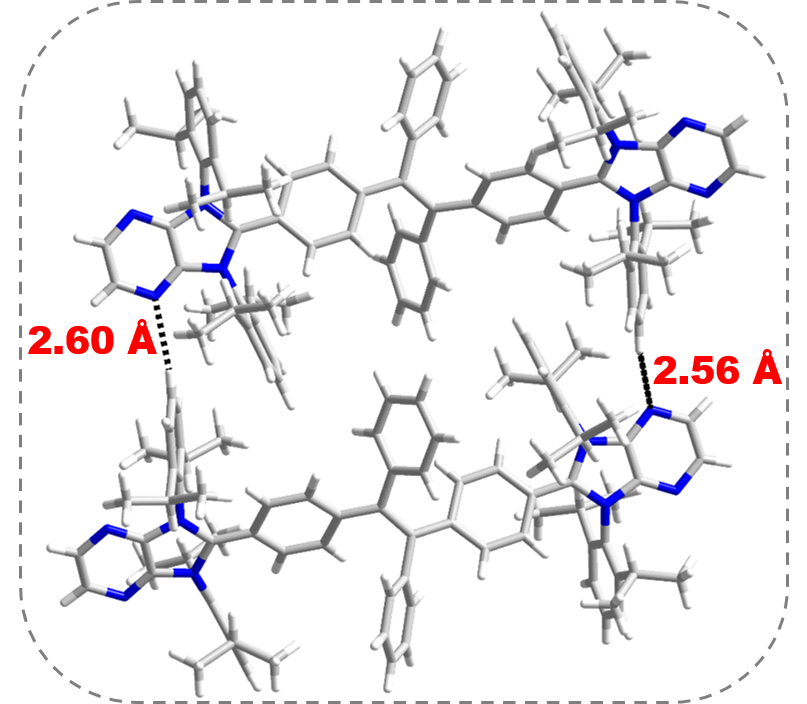


**Figure S32.** Packing of **2d** (solvates have been omitted for clarity).

**Table S2**. Selective bond lengths, angles and dihedral angle (*θ*) from X-ray single-crystal diffraction of **1a**, **1d**, **2a** and **2d**.

|  | **1a** | **2a** | **1d** | **2d** |
| --- | --- | --- | --- | --- |
| C1-C2**/**Å | 1.349(4) | 1.479(4) | 1.334(6) | 1.453(4) |
| C2-C3**/**Å | 1.494(3) | 1.397(4) | 1.504(3) | 1.400(4) |
| C6-C9**/**Å | 1.472(3) | 1.408(4) | 1.462(3) | 1.378(4) |
| C3-C4**/**Å | 1.391(4) | 1.441(4) | 1.383(4) | 1.440(4) |
| C4-C5**/**Å | 1.382(4) | 1.348(4) | 1.376(4) | 1.349(4) |
| C5-C6**/**Å | 1.389(4) | 1.427(4) | 1.396(4) | 1.438(4) |
| C6-C7**/**Å | 1.385(4) | 1.436(4) | 1.386(4) | 1.438(4) |
| C7-C8**/**Å | 1.388(4) | 1.349(4) | 1.379(4) | 1.352(4) |
| C3-C8**/**Å | 1.381(4) | 1.439(4) | 1.395(4) | 1.434(4) |
| C9-N1**/**Å | 1.341(3) | 1.385(3) | 1.349(3) | 1.408(3) |
| C9-N2**/**Å | 1.340(3) | 1.374(4) | 1.358(3) | 1.413(3) |
| C10-N1**/**Å | 1.392(4) | 1.415(4) | 1.389(3) | 1.376(4) |
| C11-N2**/**Å | 1.395(3) | 1.414(3) | 1.395(3) | 1.385(4) |
| C10-C11**/**Å | 1.349(4) | 1.340(4) | 1.379(4) | 1.396(4) |
| ∠C3-C2-C14/° | 115.4(2) | 122.7(2) | 114.2(2) | 118.9(3) |
| ∠N1-C9-N2/° | 107.2(2) | 104.2(2) | 109.0(2) | 105.1(2) |
| *θ*/° | 6.5 | 54.7 | 0.0 | 35.6 |


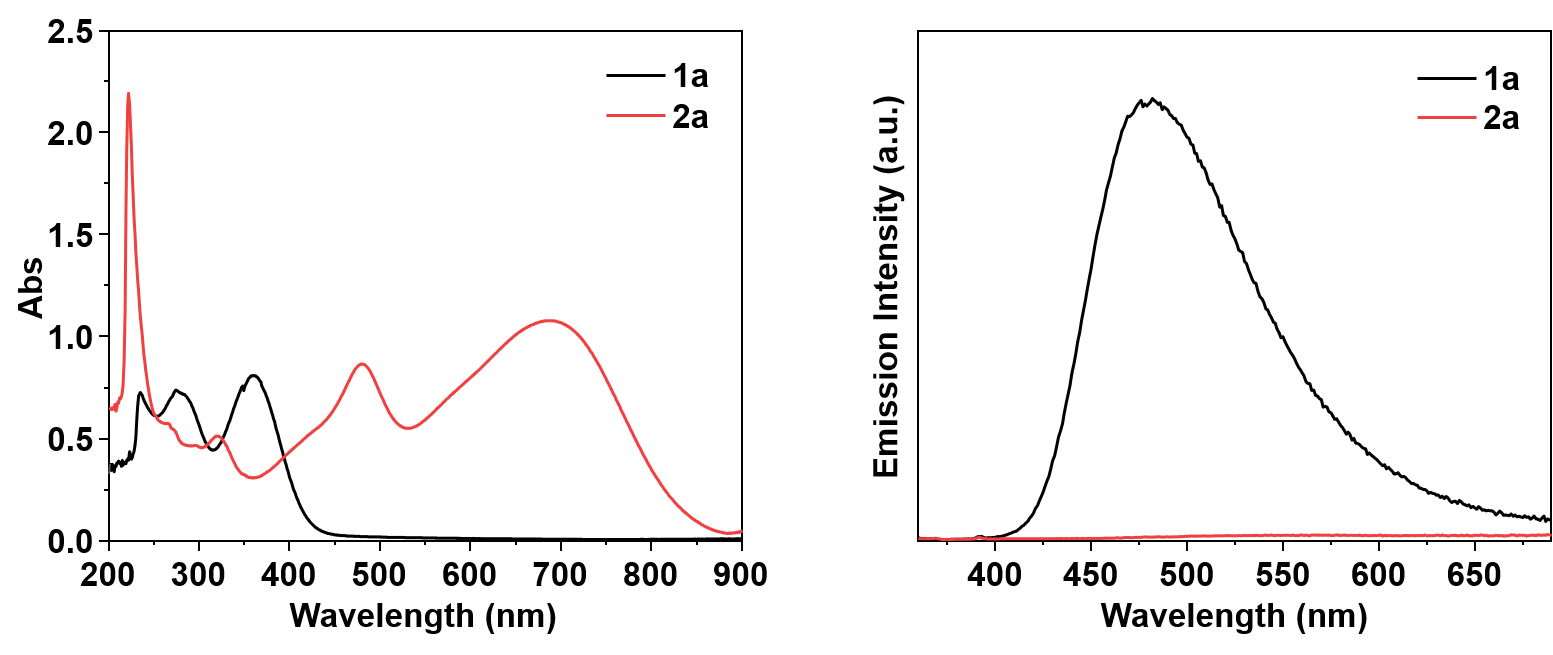


**Figure S33**. UV-vis (left) and fluorescence (right) spectra of **1a** and **2a** in THF (*T* = 298 K, *c* = 10^−4^ M, *λ*_ex_ = 350 nm).


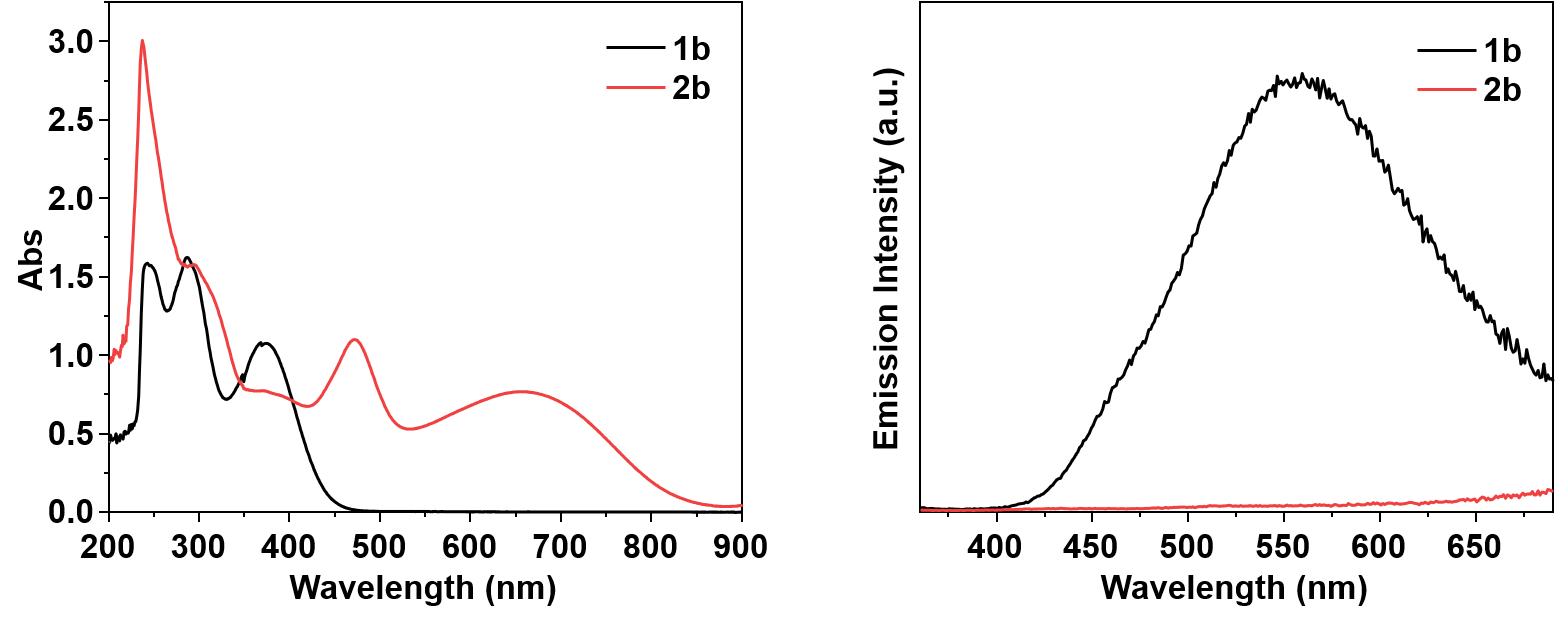


**Figure S34**. UV-vis (left) and fluorescence (right) spectra of **1b** and **2b** in THF (*T* = 298 K, *c* = 10^−4^ M, *λ*_ex_ = 350 nm).


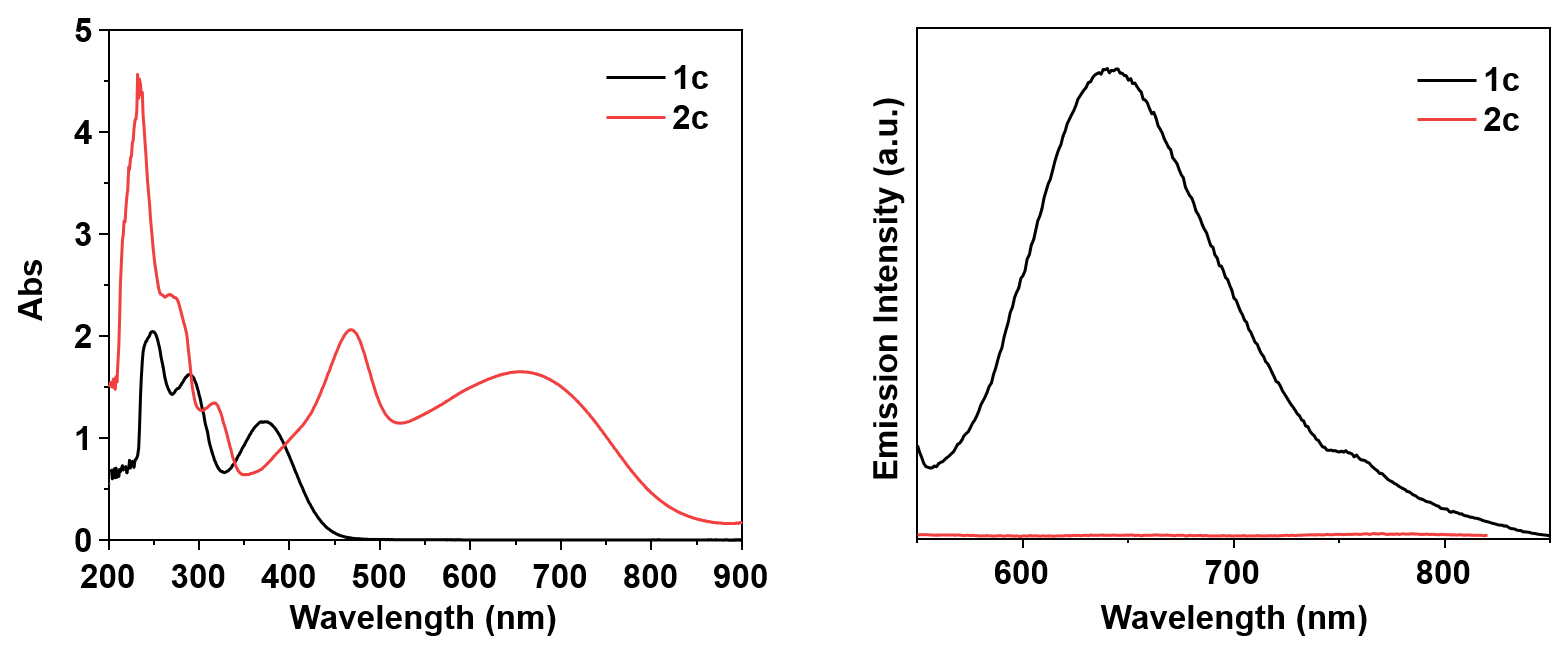


**Figure S35**. UV-vis (left) and fluorescence (right) spectra of **1c** and **2c** in THF (*T* = 298 K, *c* = 10^−4^ M, *λ*_ex_ = 470 nm).


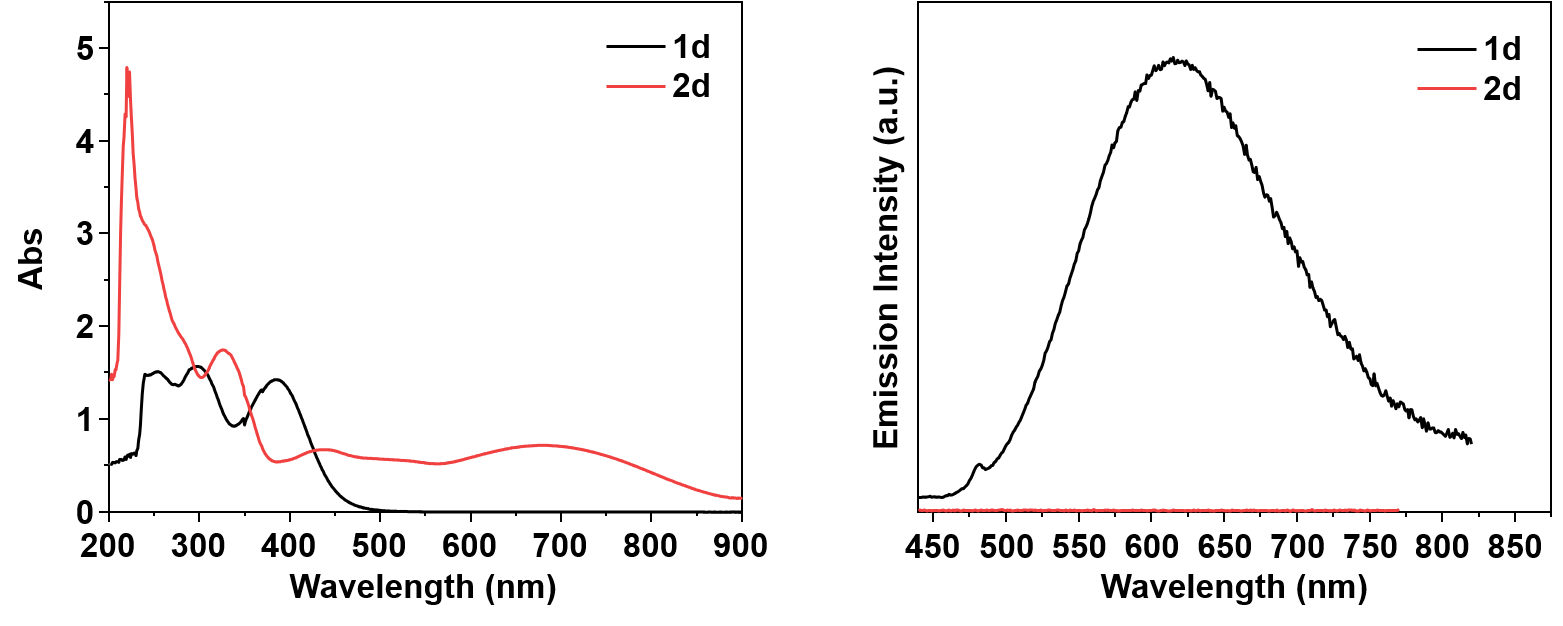


**Figure S36**. UV-vis (left) and fluorescence (right) spectra of **1d** and **2d** in THF (*T* = 298 K, *c* = 10^−4^ M, *λ*_ex_ = 420 nm).


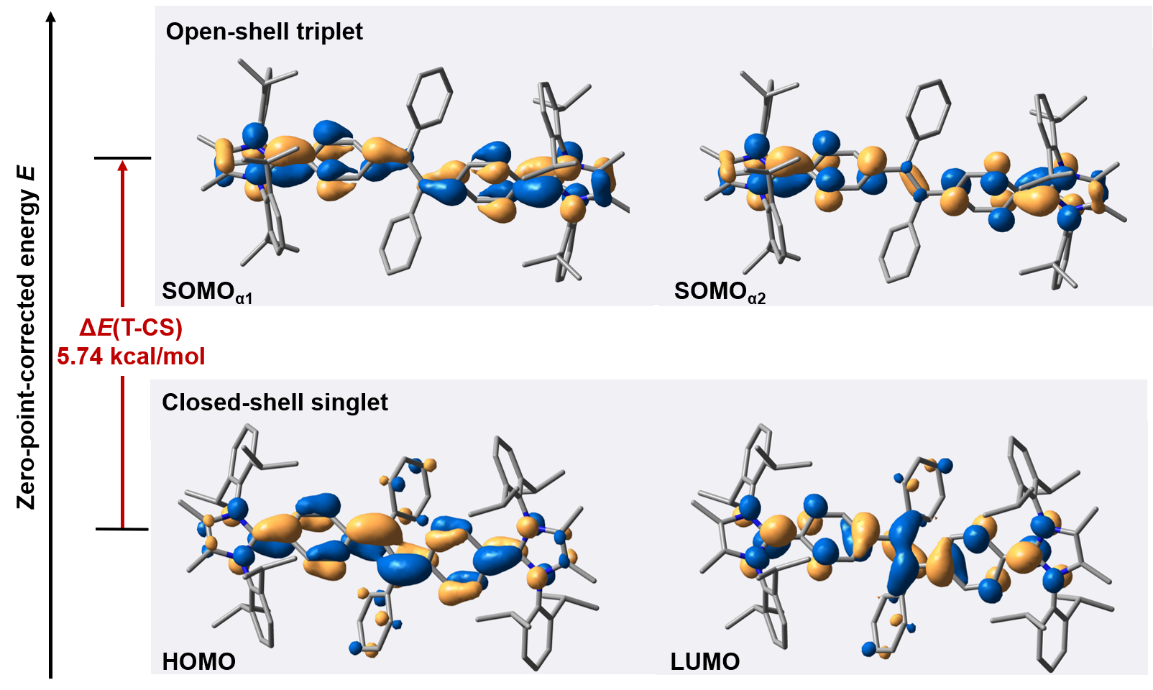


**Figure S37**. The frontier molecular orbitals (isovalue = 0.033) of **2a** in its open-shell triplet (up) and closed-shell singlet (down), and relevant adiabatic energy differences Δ*E* calculated at the UBHandHLYP/def2-SVP level of theory. Color scheme: C gray; N Klein blue.


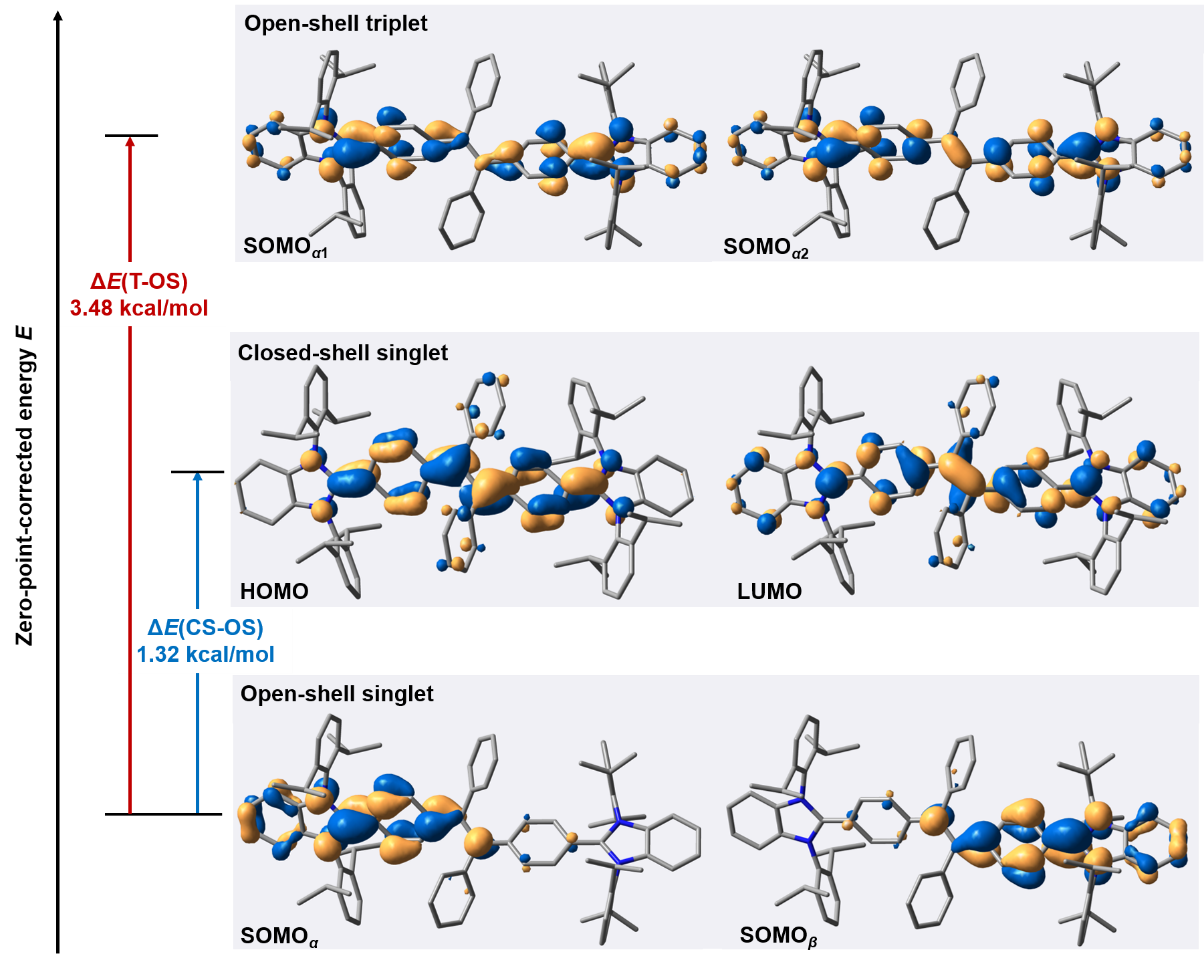


**Figure S38**. The frontier molecular orbitals (isovalue = 0.033) of **2b** in its open-shell triplet (up), closed-shell singlet (middle), and open-shell singlet (down), and relevant adiabatic energy differences Δ*E* calculated at the UBHandHLYP/def2-SVP level of theory. Color scheme: C gray; N Klein blue.


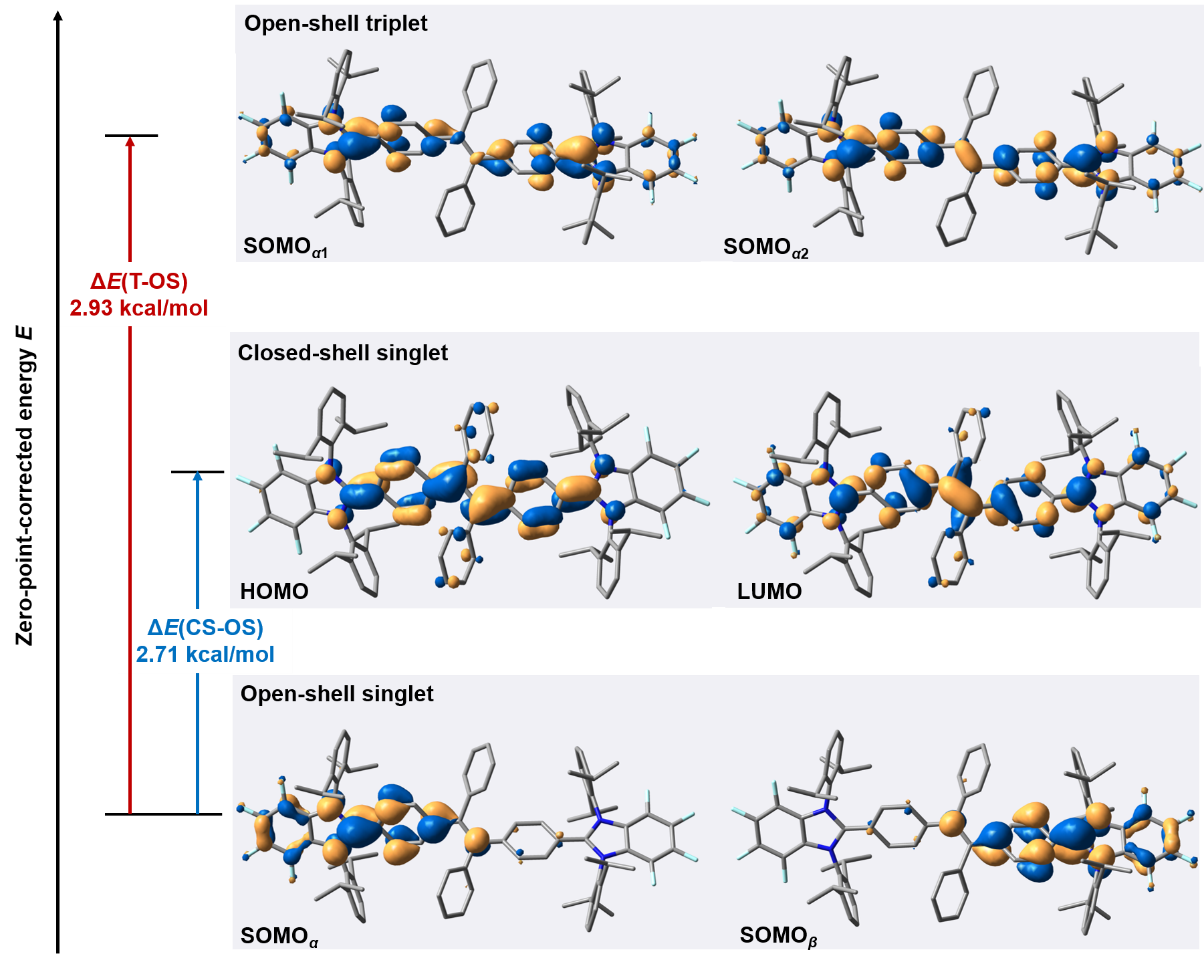


**Figure S39**. The frontier molecular orbitals (isovalue = 0.033) of **2c** in its open-shell triplet (up), closed-shell singlet (middle), and open-shell singlet (down), and relevant adiabatic energy differences Δ*E* calculated at the UBHandHLYP/def2-SVP level of theory. Color scheme: C gray; N Klein blue; F light blue.


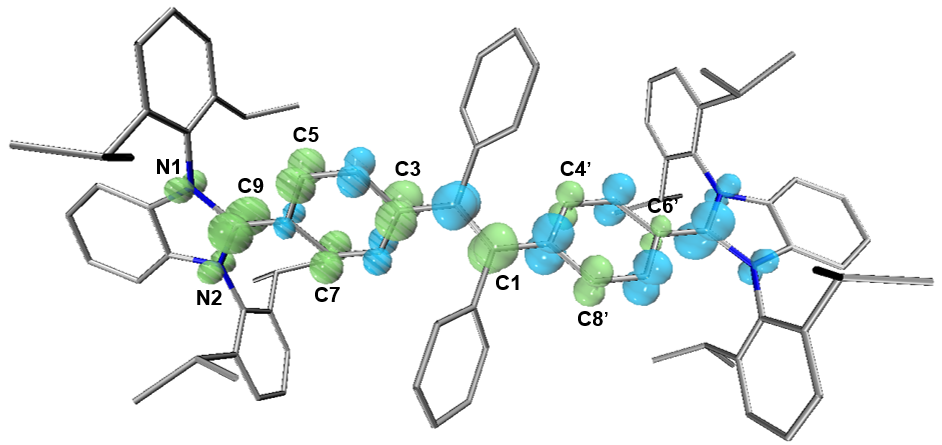


**Figure S40.** Spin densities (isovalue = 0.010) of **2b** in its open-shell singlet, calculated at the UBHandHLYP/def2-SVP level of theory. Green represents alpha spin (+), blue represents beta spin (-).


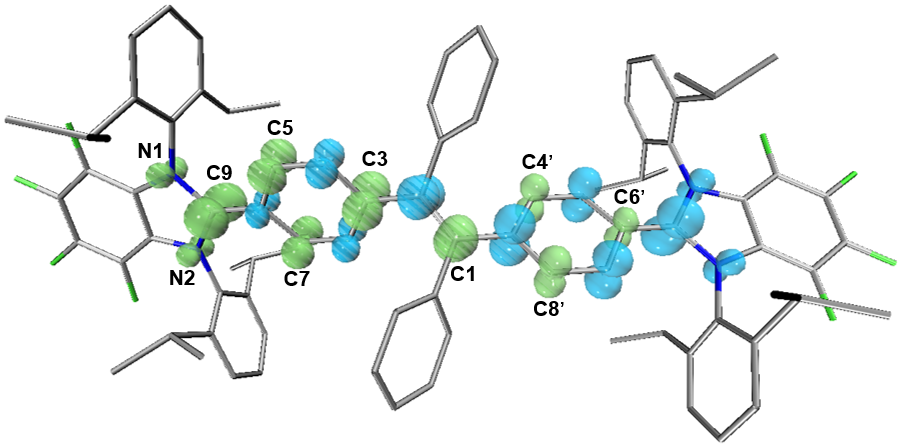


**Figure S41.** Spin densities (isovalue = 0.010) of **2c** in its open-shell singlet, calculated at the UBHandHLYP/def2-SVP level of theory. Green represents alpha spin (+), blue represents beta spin (-).


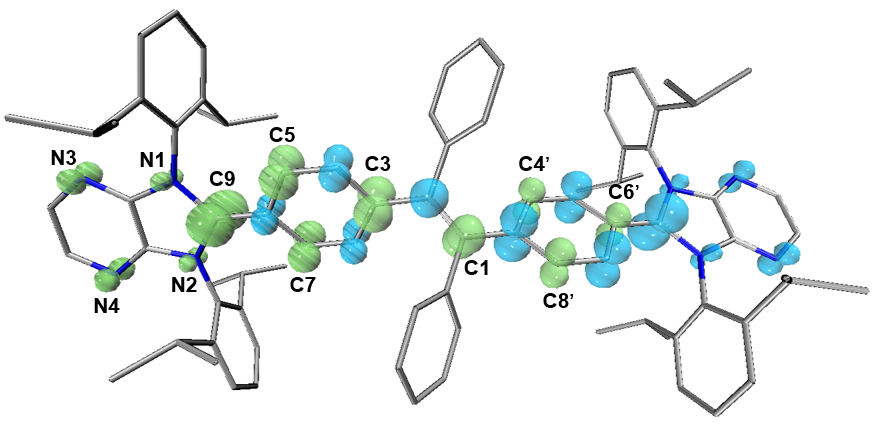


**Figure S42.** Spin densities (isovalue = 0.010) of **2d** in its open-shell singlet, calculated at the UBHandHLYP/def2-SVP level of theory. Green represents alpha spin (+), blue represents beta spin (-).


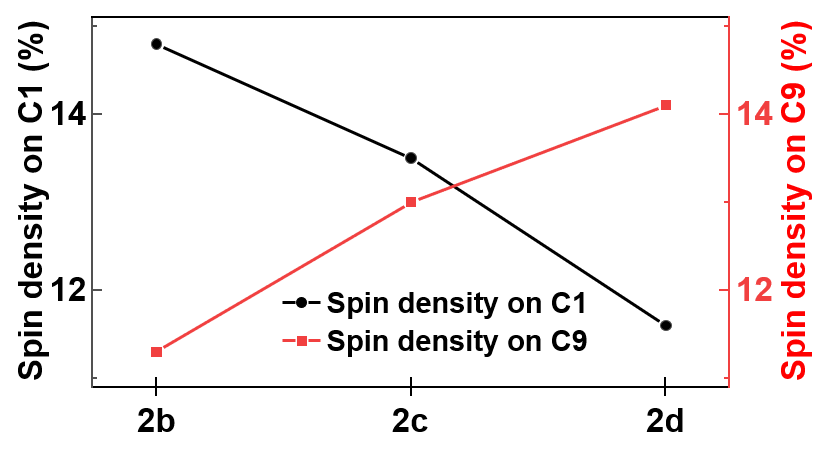


**Figure S43.** Spin density (in % of absolute spin) on C1 and C9 in open-shell singlets for **2b**–**d**, calculated at the UBHandHLYP/def2-SVP level of theory.

**Table S3.** Spin densities (in % of absolute spin) of **2b**–**d** calculated at the UBHandHLYP/def2-SVP level of theory.

| Spin densities (%) | **2b** | **2c** | **2d** |
| --- | --- | --- | --- |
| *ρ*_N1/N2_ | 1.9/1.9 | 1.9/1.9 | 0.9/0.9 |
| *ρ*_N3/N4_ | - | - | 3.0/3.0 |
| *ρ*_C1_ | 14.8 | 13.5 | 11.6 |
| *ρ*_C3_ | 13.3 | 13.0 | 12.1 |
| *ρ*_C5_ | 9.1 | 9.4 | 9.3 |
| *ρ*_C7_ | 9.5 | 9.8 | 9.4 |
| *ρ*_C9_ | 11.3 | 13.0 | 14.1 |
| *ρ*_C4’_ | 7.9 | 8.0 | 7.7 |
| *ρ*_C6’_ | 6.6 | 7.5 | 8.0 |
| *ρ*_C8’_ | 8.1 | 8.1 | 7.7 |


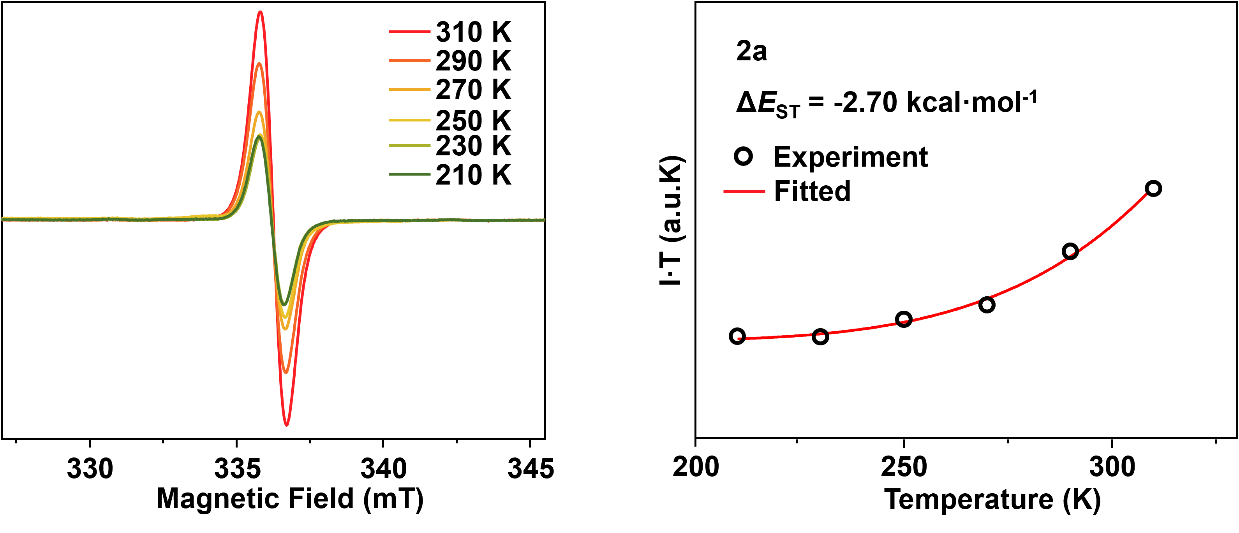


**Figure S44**. Variable-temperature EPR spectra of **2a** in the solid state (left). The product of EPR signal integrations (*I*) and temperature (*T*) versus temperature (*T*) of **2a** and the fitted curve from the Bleaney–Bowers equation (orange line) (right). Circles are the experimental results.


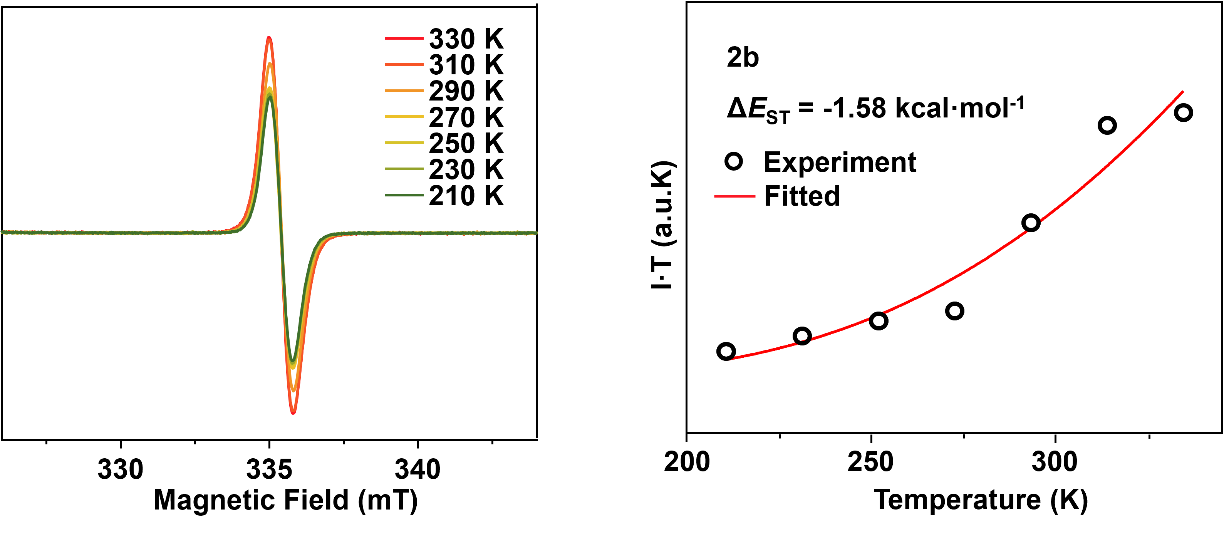


**Figure S45**. Variable-temperature EPR spectra of **2b** in the solid state (left). The product of EPR signal integrations (*I*) and temperature (*T*) versus temperature (*T*) of **2b** and the fitted curve from the Bleaney–Bowers equation (orange line) (right). Circles are the experimental results.


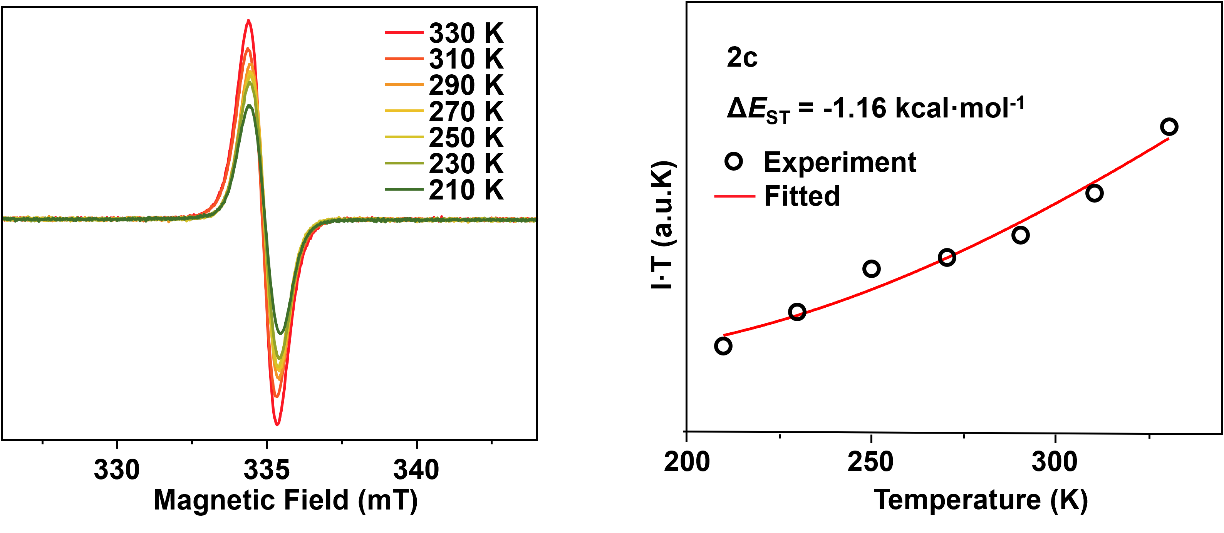


**Figure S46**. Variable-temperature EPR spectra of **2c** in the solid state (left). The product of EPR signal integrations (*I*) and temperature (*T*) versus temperature (*T*) of **2c** and the fitted curve from the Bleaney–Bowers equation (orange line) (right). Circles are the experimental results.


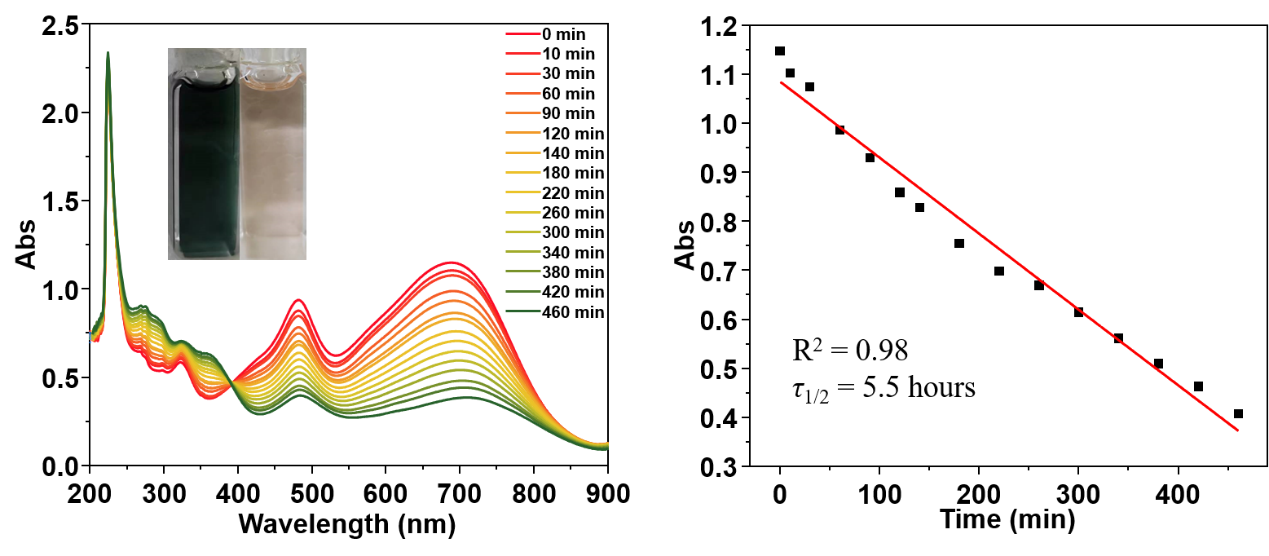


**Figure S47**. Time-dependent UV-vis spectra of **2a** in THF (*T* = 298 K, *c* = 10^−4^ M). Inset: photograph of the solution before (left) and after (right) exposure to air (left). Linear fit of Abs−time plot. The black squares represent the absorption intensity (*λ* = 688 nm), and the red line corresponds to the linear equation (right).


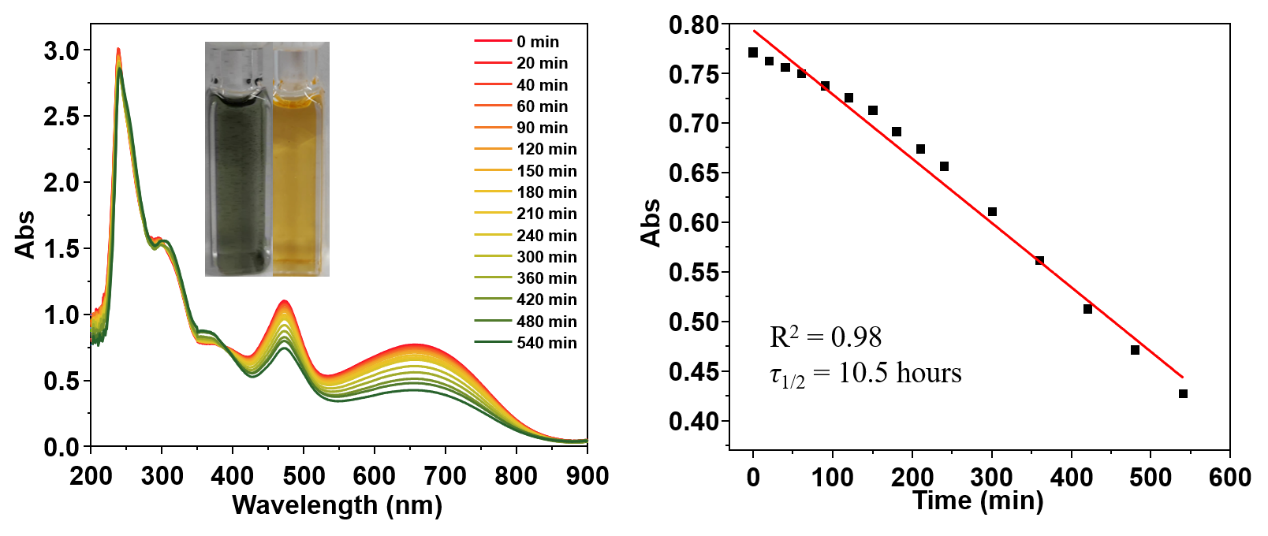


**Figure S48**. Time-dependent UV-vis spectra of **2b** in THF (*T* = 298 K, *c* = 10^−4^ M). Inset: photograph of the solution before (left) and after (right) exposure to air (left). Linear fit of Abs−time plot. The black squares represent the absorption intensity (*λ* = 688 nm), and the red line corresponds to the linear equation (right).


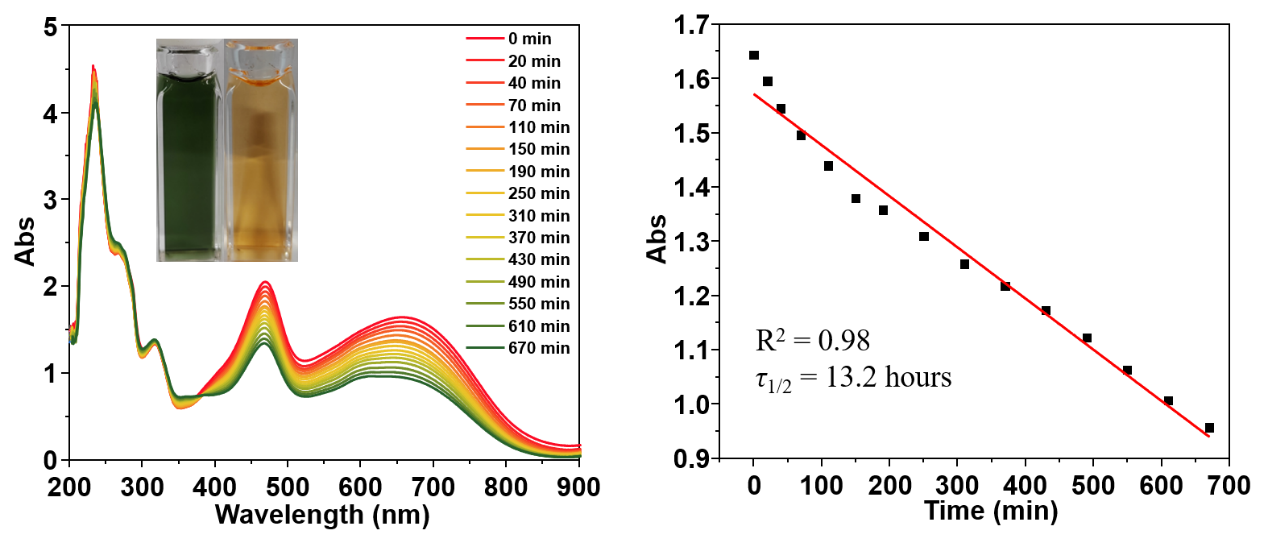


**Figure S49**. Time-dependent UV-vis spectra of **2c** in THF (*T* = 298 K, *c* = 10^−4^ M). Inset: photograph of the solution before (left) and after (right) exposure to air (left). Linear fit of Abs−time plot. The black squares represent the absorption intensity (*λ* = 688 nm), and the red line corresponds to the linear equation (right).


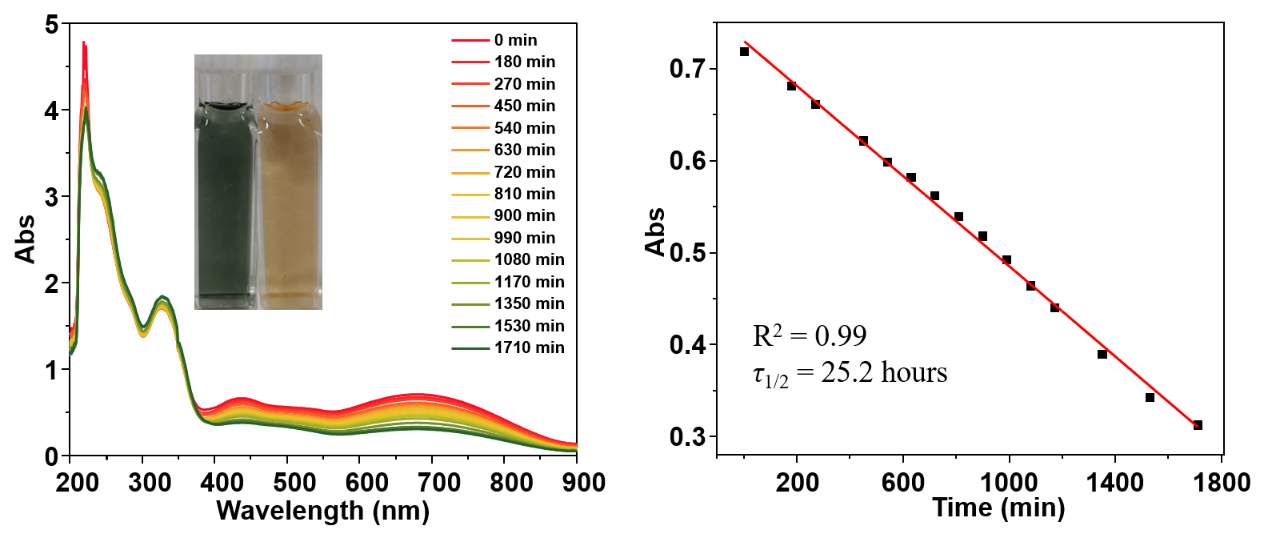


**Figure S50**. Time-dependent UV-vis spectra of **2d** in THF (*T* = 298 K, *c* = 10^−4^ M). Inset: photograph of the solution before (left) and after (right) exposure to air (left). Linear fit of Abs−time plot. The black squares represent the absorption intensity (*λ* = 688 nm), and the red line corresponds to the linear equation (right).


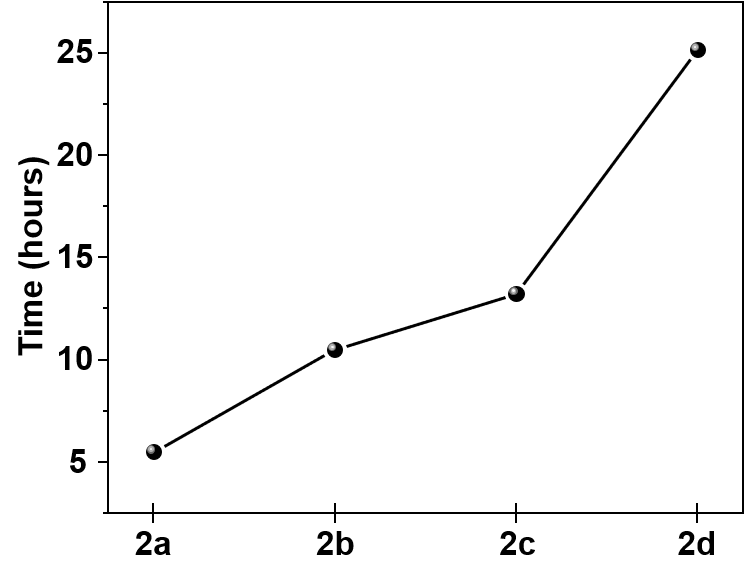


**Figure S51**. Stability of diradicals **2a**–**d** upon exposure to air.


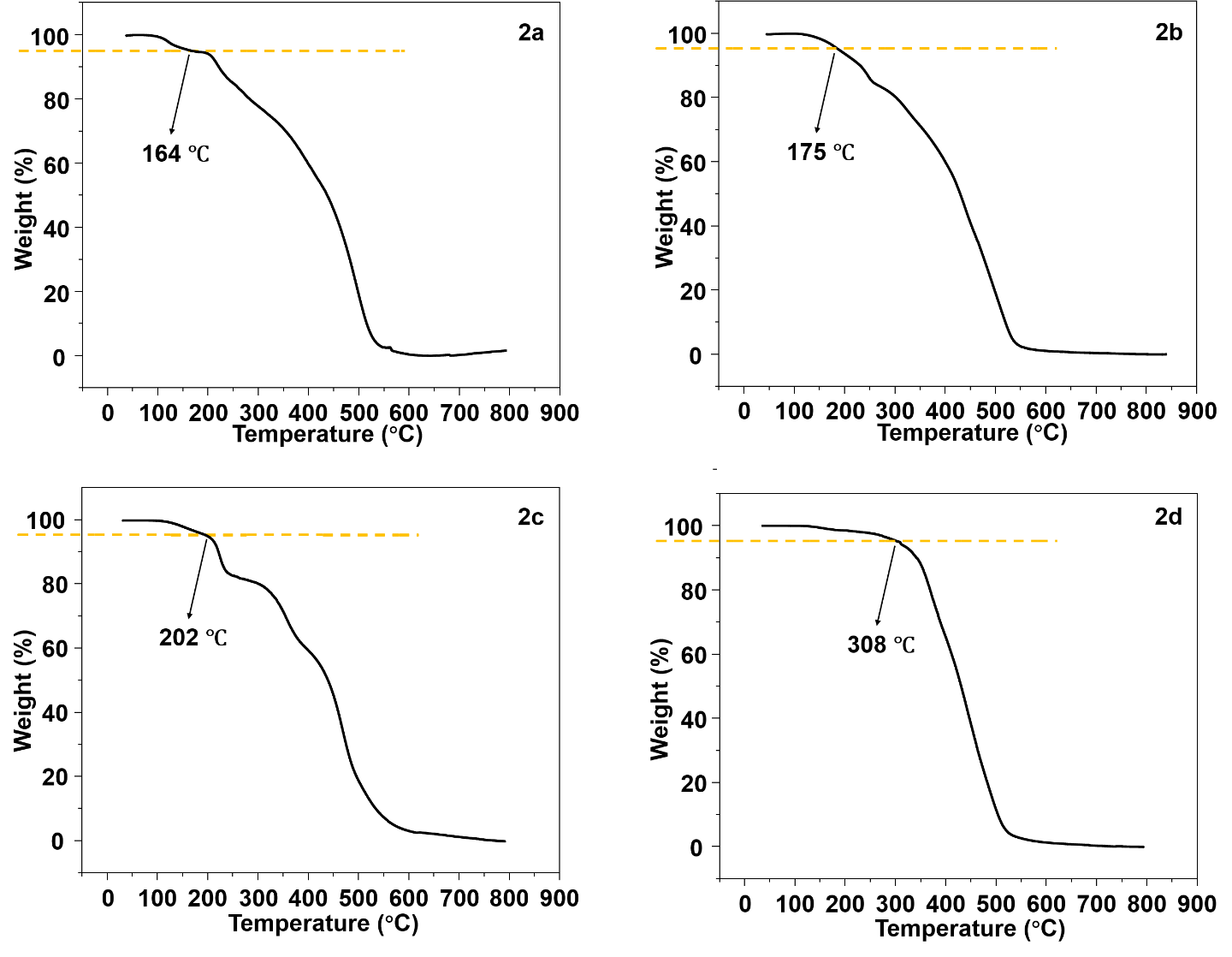


**Figure S52**. TGA of diradicals **2a**–**d** under nitrogen.


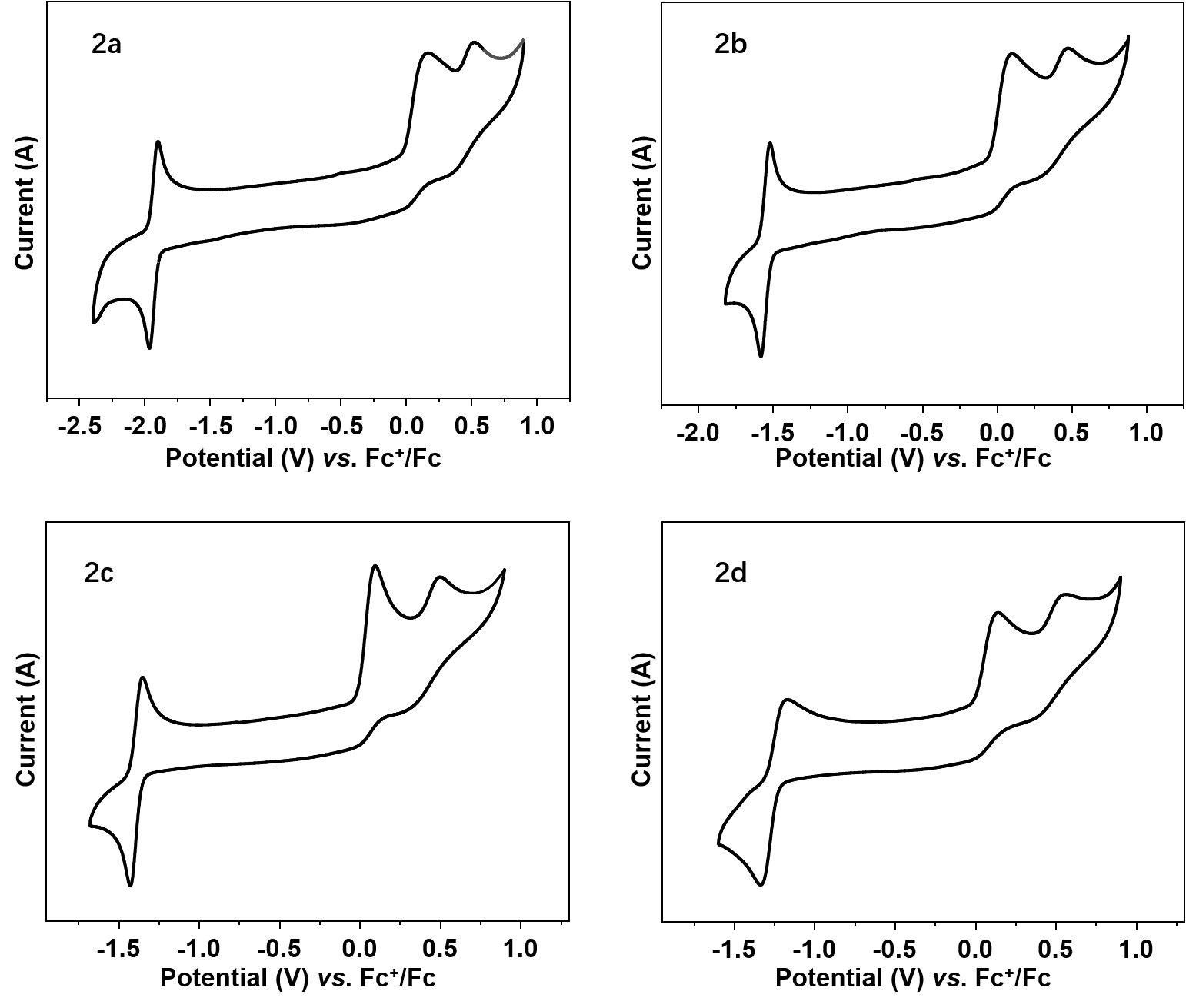


**Figure S53**. Cyclic voltammogram curves of **2a**–**d** in THF with 0.1 M *n*Bu_4_NPF_6_ at a scan rate of 100 mV·s^−1^.


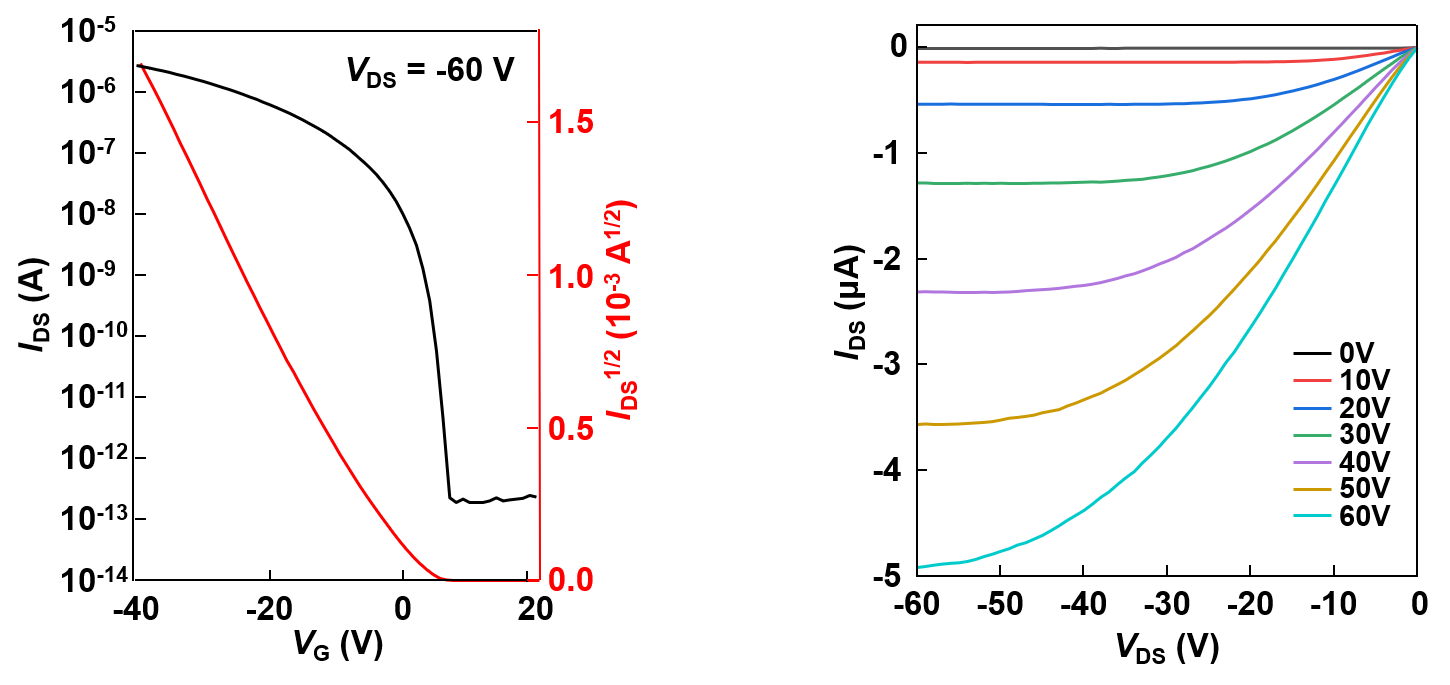


**Figure S54**. Transfer (left) and output (right) curves of the OFET device based on **2b** with a mobility of 1.42 cm^2^·V^-1^·s^-1^.


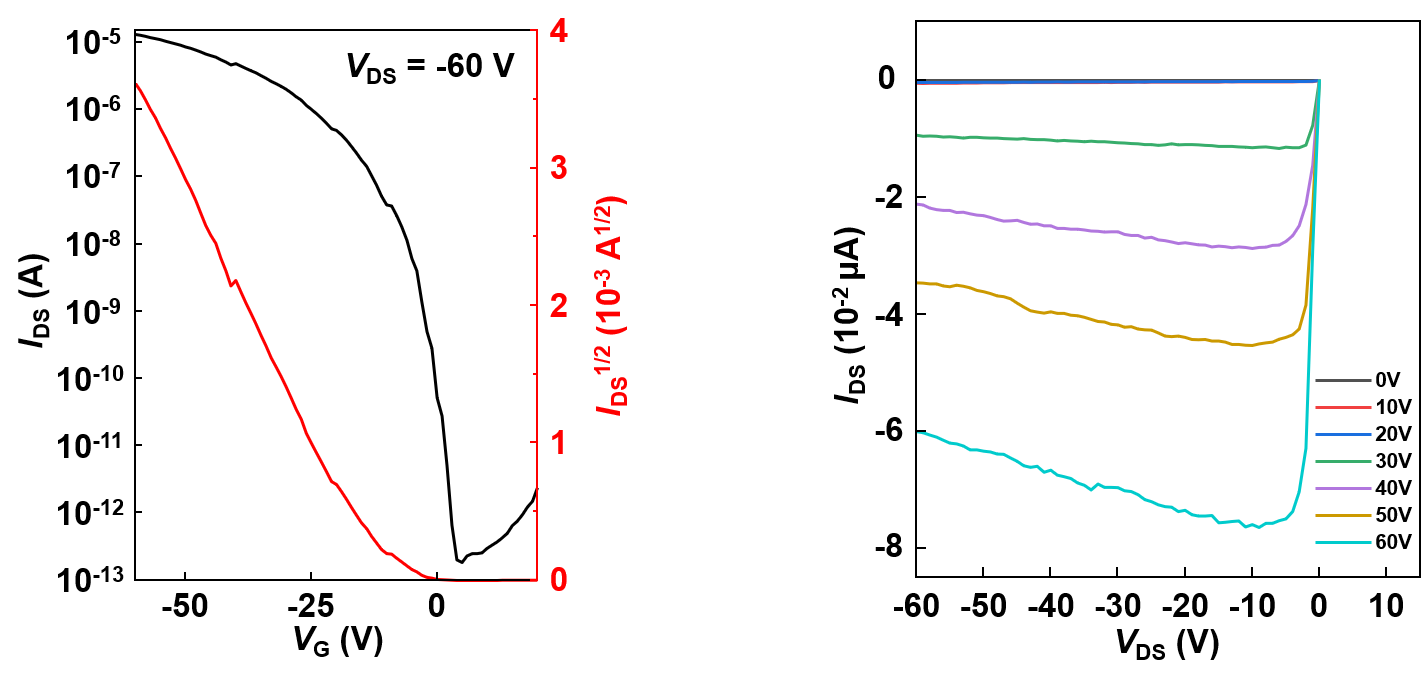


**Figure S55**. Transfer (left) and output (right) curves of the OFET device based on **2c** with a mobility of 1.75 cm^2^·V^-1^·s^-1^.


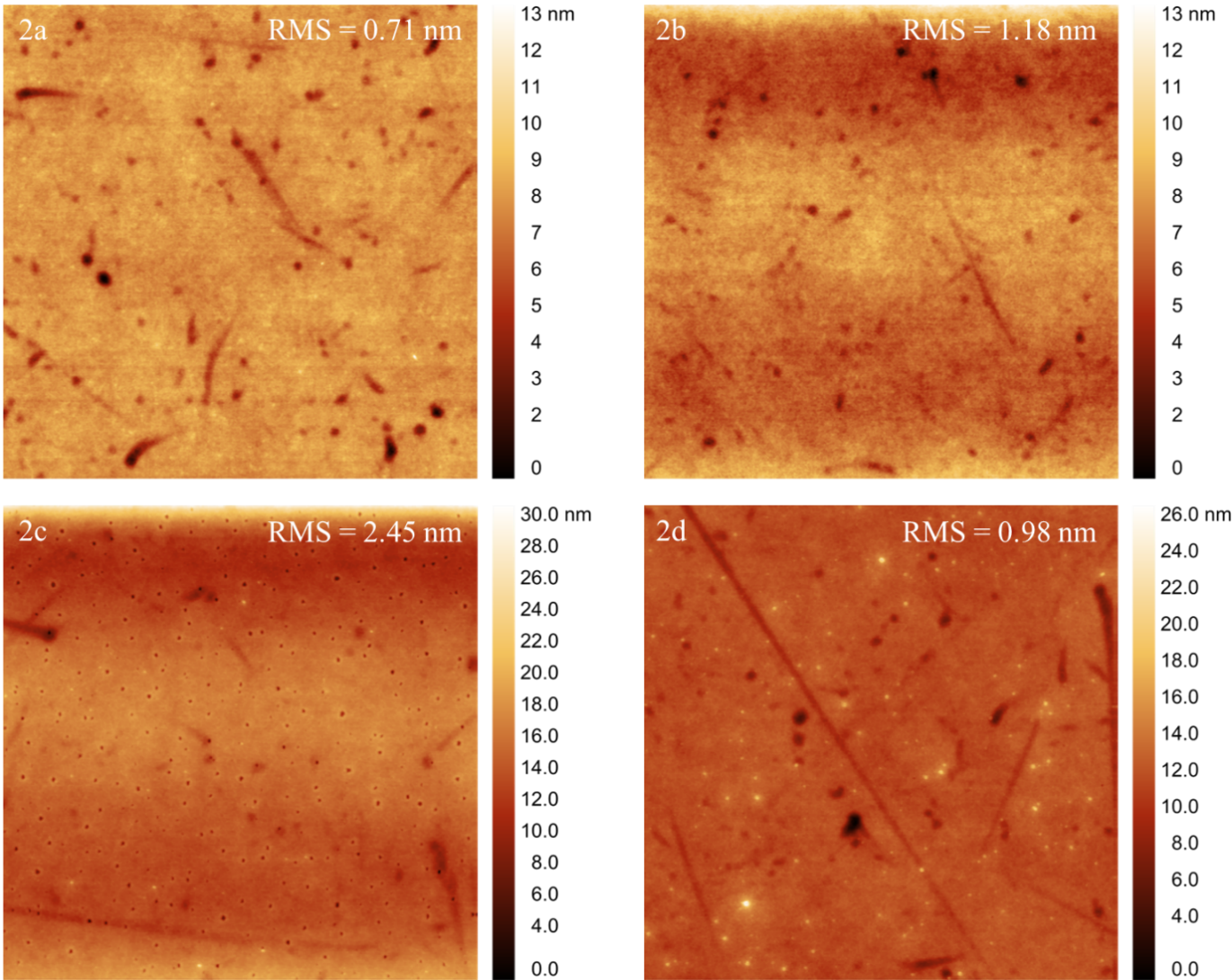


**Figure S56**. Atomic force microscopy (AFM) images (5 × 5 µm) of compounds **2a**–**d** spin-coated onto SiO_2_ substrates.

**
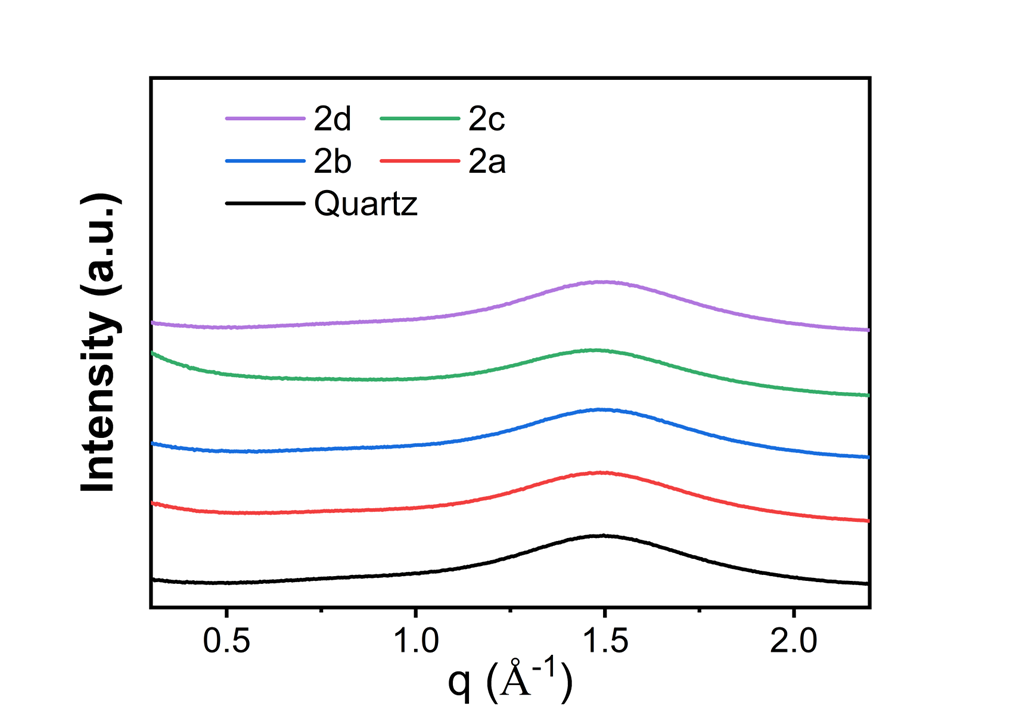
**

**Figure S57**. GIWAXS patterns for **2a**–**d** spin-coated on quartz substrates.


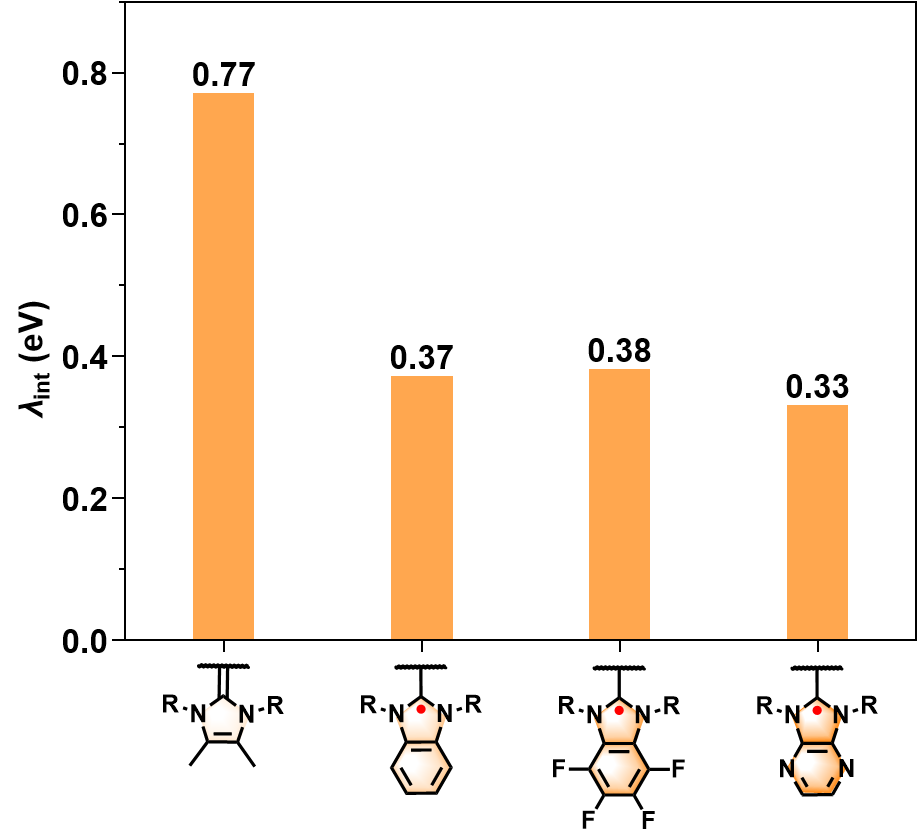


**Figure S58.** Internal reorganization energy (*λ*_int_) for **2a**–**d**, calculated at the UBHandHLYP/def2-SVP level of theory.


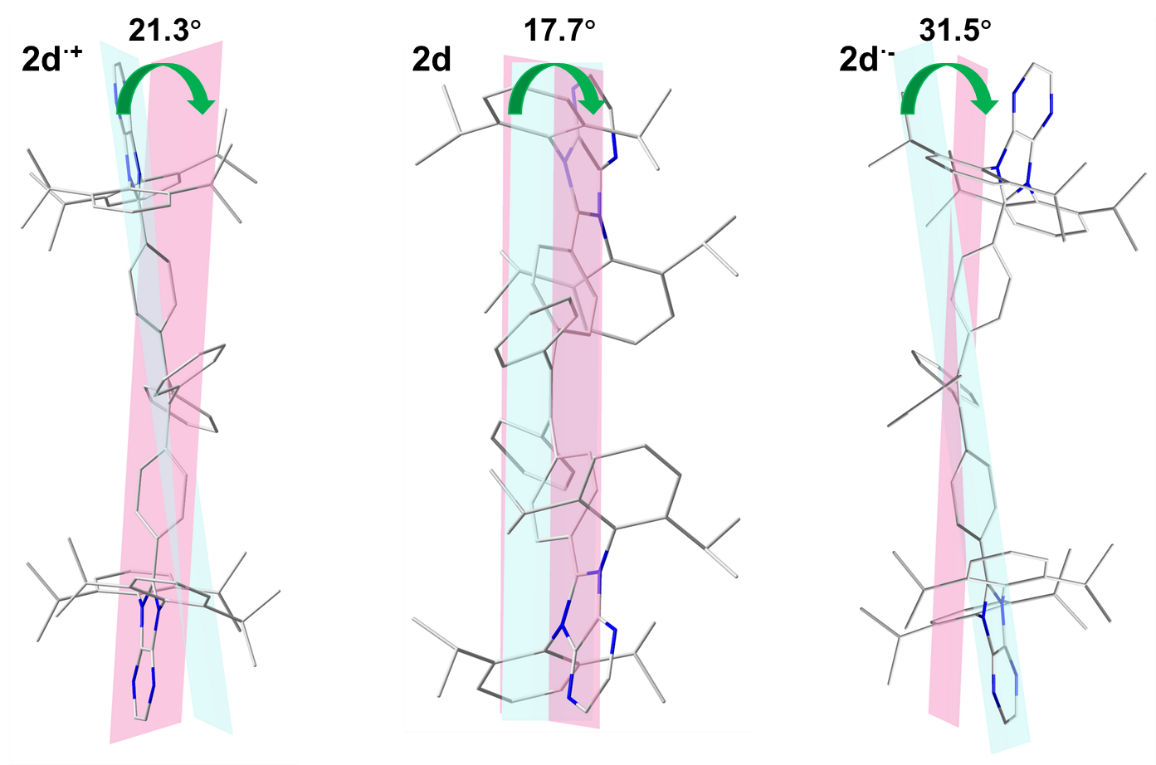


**Figure S59.** Optimized structures for **2d**^·+^, **2d**, and **2d**^·-^, calculated at the UBHandHLYP/def2-SVP level of theory.

**Table S4.** Comparison of device parameters between this work and references.

| **Semiconductor** | ***μ*_h_/cm^2^ V^-1^ s^-1^** | **On/off ratio** | **Ref.** |
| --- | --- | --- | --- |
| Ph_2_-IDPL | 0.72 | 10^3^ | ^1^ |
| DIAn  **1ab** | 2*10^-3^ | 10 | ^2^ |
|  | 1.9*10^-3^ | 10^2^ | ^3^ |
| PP | 1.4 | 10^3^ | ^4^ |
| HDIP | 0.4 | 10 | ^5^ |
| QTBDT-3H | 0.16 | 10^2^ | ^6^ |
| TBP5 | 1.2*10^-2^ | 10^5^ | ^7^ |
| **1** (4,5:12,13-DBHZ derivative) | 0.15 | 10^6^ | ^8^ |
| DFPy | 2*10^-3^ | N/A | ^9^ |
| DBOZ2 | 3.5 | 10^5^ | ^10^ |
| OR5 | 2.1*10^-2^ | 10^6^ | ^11^ |
| OFQ-8CN | 1.3*10^-2^ | 10^3^ | ^12^ |
| SD-Th | 1.5 | 10^7^ | ^13^ |
| RPH | 0.41 | 10^2^ | ^14^ |
| **2d** | 4.53 | 10^9^ | This work |

**Table S5.** Calculated internal reorganization energies (*λ*_int_​) of representative compounds for charge transport.

| **Compound** | ***λ*_int_(hole) (eV)** | ***λ*_int_(electron) (eV)** |  |
| --- | --- | --- | --- |
| **1a** | N/A | 0.76 |  |
| **1b** | N/A | 0.8 |  |
| **1c** | N/A | 0.78 |  |
| **1d** | N/A | 0.67 |  |
| **2a** | 0.77 | N/A |  |
| **2b** | 0.37 | N/A |  |
| **2c** | 0.38 | N/A |  |
| **2d** | 0.33 | N/A | |

# Single-crystal X-ray diffraction studies

**Table S6**. Crystal data for **1a**

| Empirical formula | C_84_H_98_N_4_·Cl_2_ |
| --- | --- |
| Formula weight | 1234.56 |
| Temperature/K | 230.0 |
| Crystal system | Triclinic |
| Space group | *P* |
| *a*/Å | 13.4709(13) |
| *b*/Å | 15.4859(16) |
| *c*/Å | 18.644(2) |
| *α*/° | 89.051(4) |
| *β*/° | 76.714(4) |
| *γ*/° | 88.670(3) |
| Volume/Å^3^ | 3783.9(7) |
| Z | 2 |
| *ρ*_calc_g/cm^3^ | 1.084 |
| *μ*/mm^‑1^ | 0.130 |
| *F*(000) | 1328.0 |
| Crystal size/mm^3^ | 0.36 × 0.34 × 0.29 |
| Radiation | MoKα (*λ* = 0.71073) |
| 2Θ range for data collection/° | 3.478 to 52.748 |
| Index ranges | -14 ≤ *h* ≤ 16, -19 ≤ *k* ≤ 19, -22 ≤ *l* ≤ 23 |
| Reflections collected | 52674 |
| Independent reflections | 15391 [*R*_int_ = 0.0448, *R*_sigma_ = 0.0494] |
| Data/restraints/parameters | 15391/111/849 |
| Goodness-of-fit on *F*^2^ | 1.070 |
| Final *R* indexes [*I*>=2*σ* (*I*)] | *R*_1_ = 0.0882, *wR*_2_ = 0.2594 |
| Final *R* indexes [all data] | *R*_1_ = 0.1313, *wR*_2_ = 0.2948 |
| Largest diff. peak/hole / e Å^-3^ | 1.34/-0.89 |
| CCDC number | 2498013 |

**Table S7**. Crystal data for **1b**

| Empirical formula | C_88_H_94_N_4_·Br_2_ |
| --- | --- |
| Formula weight | 1367.49 |
| Temperature/K | 207.0 |
| Crystal system | monoclinic |
| Space group | *P2_1_/n* |
| *a*/Å | 13.8043(7) |
| *b*/Å | 32.138(2) |
| *c*/Å | 17.7691(11) |
| *α*/° | 90 |
| *β*/° | 106.445(2) |
| *γ*/° | 90 |
| Volume/Å^3^ | 7560.8(8) |
| Z | 4 |
| *ρ*_calc_g/cm^3^ | 1.201 |
| *μ*/mm^‑1^ | 1.119 |
| *F*(000) | 2880.0 |
| Crystal size/mm^3^ | 0.4 × 0.38 × 0.35 |
| Radiation | MoKα (*λ* = 0.71073) |
| 2Θ range for data collection/° | 3.318 to 52.722 |
| Index ranges | -17 ≤ *h* ≤ 16, -40 ≤ *k* ≤ 34, -22 ≤ *l* ≤ 22 |
| Reflections collected | 48432 |
| Independent reflections | 15346 [*R*_int_ = 0.0457, *R*_sigma_ = 0.0601] |
| Data/restraints/parameters | 15346/0/863 |
| Goodness-of-fit on *F*^2^ | 1.031 |
| Final *R* indexes [*I*>=2*σ* (*I*)] | *R*_1_ = 0.0524, *wR*_2_ = 0.1260 |
| Final *R* indexes [all data] | *R*_1_ = 0.1014, *wR*_2_ = 0.1491 |
| Largest diff. peak/hole / e Å^-3^ | 0.41/-0.90 |
| CCDC number | 2498014 |

**Table S8**. Crystal data for **1c**

| Empirical formula | C_88_H_86_F_8_N_4_·I_2_ |
| --- | --- |
| Formula weight | 1605.40 |
| Temperature/K | 200.0 |
| Crystal system | triclinic |
| Space group | *P* |
| *a*/Å | 10.6712(4) |
| *b*/Å | 19.6218(8) |
| *c*/Å | 21.5533(9) |
| *α*/° | 72.0180(10) |
| *β*/° | 80.6320(10) |
| *γ*/° | 89.6750(10) |
| Volume/Å^3^ | 4230.5(3) |
| Z | 2 |
| *ρ*_calc_g/cm^3^ | 1.260 |
| *μ*/mm^‑1^ | 0.804 |
| *F*(000) | 1640.0 |
| Crystal size/mm^3^ | 0.34 × 0.32 × 0.29 |
| Radiation | MoKα (*λ* = 0.71073) |
| 2Θ range for data collection/° | 4.202 to 52.804 |
| Index ranges | -11 ≤ *h* ≤ 13, -24 ≤ *k* ≤ 24, -26 ≤ *l* ≤ 26 |
| Reflections collected | 67222 |
| Independent reflections | 17219 [*R*_int_ = 0.0576, *R*_sigma_ = 0.0624] |
| Data/restraints/parameters | 17219/90/959 |
| Goodness-of-fit on *F*^2^ | 1.025 |
| Final *R* indexes [*I*>=2*σ* (*I*)] | *R*_1_ = 0.0761, *wR*_2_ = 0.2203 |
| Final *R* indexes [all data] | *R*_1_ = 0.1257, *wR*_2_ = 0.2535 |
| Largest diff. peak/hole / e Å^-3^ | 1.45/-0.77 |
| CCDC number | 2498015 |

**Table S9**. Crystal data for **1d***2CH_3_CN

| Empirical formula | C_84_H_90_N_8_·2CF_3_O_3_S·2C_2_H_3_N |
| --- | --- |
| Formula weight | 1591.88 |
| Temperature/K | 299.0 |
| Crystal system | triclinic |
| Space group | *P* |
| *a*/Å | 10.783(7) |
| *b*/Å | 13.344(10) |
| *c*/Å | 16.912(12) |
| *α*/° | 88.06(2) |
| *β*/° | 78.79(2) |
| *γ*/° | 68.279(19) |
| Volume/Å^3^ | 2216(3) |
| Z | 1 |
| *ρ*_calc_g/cm^3^ | 1.193 |
| *μ*/mm^‑1^ | 0.129 |
| *F*(000) | 840.0 |
| Crystal size/mm^3^ | 0.32 × 0.3 × 0.28 |
| Radiation | MoKα (*λ* = 0.71073) |
| 2Θ range for data collection/° | 4.54 to 51.386 |
| Index ranges | -13 ≤ *h* ≤ 11, -16 ≤ *k* ≤ 16, -20 ≤ *l* ≤ 20 |
| Reflections collected | 27453 |
| Independent reflections | 8226 [*R*_int_ = 0.0368, *R*_sigma_ = 0.0388] |
| Data/restraints/parameters | 8226/3/523 |
| Goodness-of-fit on *F*^2^ | 1.064 |
| Final *R* indexes [*I*>=2*σ* (*I*)] | *R*_1_ = 0.0880, *wR*_2_ = 0.2500 |
| Final *R* indexes [all data] | *R*_1_ = 0.1164, *wR*_2_ = 0.2822 |
| Largest diff. peak/hole / e Å^-3^ | 0.98/-0.68 |
| CCDC number | 2498016 |

**Table S10**. Crystal data for **2a**

| Empirical formula | C_84_H_98_N_4_ |
| --- | --- |
| Formula weight | 1163.66 |
| Temperature/K | 220.0 |
| Crystal system | monoclinic |
| Space group | *P*2_1_*/n* |
| *a*/Å | 16.867(2) |
| *b*/Å | 19.0991(18) |
| *c*/Å | 26.118(4) |
| *α*/° | 90 |
| *β*/° | 96.146(5) |
| *γ*/° | 90 |
| Volume/Å^3^ | 8365.7(17) |
| Z | 4 |
| *ρ*_calc_g/cm^3^ | 0.924 |
| *μ*/mm^‑1^ | 0.053 |
| *F*(000) | 2520.0 |
| Crystal size/mm^3^ | 0.24 × 0.23 × 0.08 |
| Radiation | MoKα (*λ* = 0.71073) |
| 2Θ range for data collection/° | 4.266 to 51.412 |
| Index ranges | -20 ≤ *h* ≤ 19, -23 ≤ *k* ≤ 23, -31 ≤ *l* ≤ 26 |
| Reflections collected | 55902 |
| Independent reflections | 15728 [*R*_int_ = 0.0675, *R*_sigma_ = 0.0810] |
| Data/restraints/parameters | 15728/54/801 |
| Goodness-of-fit on *F*^2^ | 1.032 |
| Final *R* indexes [*I*>=2*σ* (*I*)] | *R*_1_ = 0.0823, *wR*_2_ = 0.2455 |
| Final *R* indexes [all data] | *R*_1_ = 0.1397, *wR*_2_ = 0.2916 |
| Largest diff. peak/hole / e Å^-3^ | 0.30/-0.33 |
| CCDC number | 2498017 |

**Table S11**. Crystal data for **2d***THF*Pentane

| Empirical formula | C_84_H_90_N_8_·C_4_H_10_O·C_5_H_12_ |
| --- | --- |
| Formula weight | 1357.90 |
| Temperature/K | 220.0 |
| Crystal system | monoclinic |
| Space group | *P*2_1_*/c* |
| *a*/Å | 23.4865(18) |
| *b*/Å | 18.8499(16) |
| *c*/Å | 21.0472(15) |
| *α*/° | 90 |
| *β*/° | 115.812(2) |
| *γ*/° | 90 |
| Volume/Å^3^ | 8388.3(11) |
| Z | 4 |
| *ρ*_calc_g/cm^3^ | 1.075 |
| *μ*/mm^‑1^ | 0.063 |
| *F*(000) | 2936.0 |
| Crystal size/mm^3^ | 0.32 × 0.11 × 0.11 |
| Radiation | MoKα (*λ* = 0.71073) |
| 2Θ range for data collection/° | 4.348 to 50.744 |
| Index ranges | -26 ≤ *h* ≤ 28, -22 ≤ *k* ≤ 16, -25 ≤ *l* ≤ 22 |
| Reflections collected | 38837 |
| Independent reflections | 15155 [*R*_int_ = 0.0721, *R*_sigma_ = 0.1073] |
| Data/restraints/parameters | 15155/50/939 |
| Goodness-of-fit on *F*^2^ | 1.015 |
| Final *R* indexes [*I*>=2*σ* (*I*)] | *R*_1_ = 0.0745, *wR*_2_ = 0.1782 |
| Final *R* indexes [all data] | *R*_1_ = 0.1686, *wR*_2_ = 0.2234 |
| Largest diff. peak/hole / e Å^-3^ | 0.41/-0.32 |
| CCDC number | 2498019 |

# Cartesian coordinates of the optimized geometries from DFT calculations

| **1a**^2+^ (B3LYP/6-31g*) | | | |  | **1b**^2+^ (B3LYP/6-31g*) | | | |
| --- | --- | --- | --- | --- | --- | --- | --- | --- |
| Atom | X | Y | Z |  | Atom | X | Y | Z |
| N | -7.114972 | 1.004691 | -0.015355 |  | N | -6.897297 | -1.144357 | -0.299292 |
| N | -6.92818 | -1.132504 | 0.377788 |  | N | -7.078874 | 1.036655 | -0.007018 |
| N | 6.892193 | 0.134955 | 1.306506 |  | N | 7.0789 | -1.036643 | -0.007031 |
| N | 7.147793 | -0.036619 | -0.852577 |  | N | 6.89726 | 1.144366 | -0.299293 |
| C | 4.750563 | -0.104252 | -0.028887 |  | C | -6.196788 | 0.001932 | -0.080137 |
| C | 1.920047 | -0.322838 | -0.334823 |  | C | -8.26125 | -0.840168 | -0.355058 |
| C | -6.21216 | -0.009421 | 0.093835 |  | C | -4.739512 | 0.108442 | 0.055322 |
| C | -1.916916 | 0.336704 | -0.295314 |  | C | 6.196785 | -0.001941 | -0.08014 |
| C | -4.750703 | 0.094613 | -0.053703 |  | C | -8.373995 | 0.544269 | -0.196383 |
| C | 0.017899 | -1.903259 | -0.779738 |  | C | -4.166455 | 0.990118 | 0.990459 |
| C | 6.210678 | -0.003229 | 0.135276 |  | H | -4.796974 | 1.587071 | 1.634538 |
| C | -0.439715 | 0.535564 | -0.378062 |  | C | 8.37401 | -0.544213 | -0.196358 |
| C | 2.805772 | -0.976919 | -1.209582 |  | C | -6.402528 | -2.51034 | -0.37192 |
| H | 2.409018 | -1.588524 | -2.013522 |  | C | 6.796883 | -2.458091 | 0.122037 |
| C | 0.444874 | -0.502083 | -0.478338 |  | C | 4.739501 | -0.108468 | 0.055324 |
| C | 3.86498 | 0.538192 | 0.860249 |  | C | -2.78704 | 1.103762 | 1.104186 |
| H | 4.243571 | 1.124197 | 1.68466 |  | H | -2.373745 | 1.796097 | 1.830245 |
| C | -0.005445 | 1.966821 | -0.372467 |  | C | 1.91775 | -0.337277 | 0.305562 |
| C | -6.844004 | 2.414444 | -0.258336 |  | C | 0.440218 | -0.523092 | 0.413645 |
| C | 4.183631 | -0.861919 | -1.073028 |  | C | -0.003629 | 1.945772 | 0.561184 |
| H | 4.813482 | -1.376885 | -1.783077 |  | C | -1.917755 | 0.337231 | 0.305539 |
| C | 2.48799 | 0.432304 | 0.706127 |  | C | 8.261228 | 0.84022 | -0.35502 |
| H | 1.842525 | 0.94317 | 1.411882 |  | C | -6.796838 | 2.458084 | 0.122182 |
| C | 6.344938 | 0.16137 | 2.654931 |  | C | -2.498175 | -0.538059 | -0.631149 |
| C | -8.404724 | 0.518025 | 0.21236 |  | H | -1.860467 | -1.127432 | -1.280281 |
| C | -8.29023 | -0.824604 | 0.435182 |  | C | 0.003627 | -1.945816 | 0.561275 |
| C | -2.789191 | 1.167837 | -1.020896 |  | C | 7.010681 | -3.076161 | 1.37458 |
| H | -2.379567 | 1.921764 | -1.685318 |  | C | 3.876903 | 0.655361 | -0.753986 |
| C | -4.169146 | 1.046369 | -0.914853 |  | H | 4.281641 | 1.33112 | -1.494946 |
| H | -4.79011 | 1.704551 | -1.504265 |  | C | -9.384234 | -1.655586 | -0.515713 |
| C | -6.427591 | -2.492566 | 0.514204 |  | H | -9.294253 | -2.731864 | -0.606291 |
| C | 8.424698 | 0.091913 | -0.299844 |  | C | -6.137684 | -3.189228 | 0.835137 |
| C | 8.266043 | 0.175891 | 1.054185 |  | C | 4.166458 | -0.990148 | 0.990464 |
| C | -7.075753 | 2.934365 | -1.552382 |  | H | 4.796992 | -1.587097 | 1.634533 |
| C | -6.403969 | 3.206286 | 0.822033 |  | C | 9.610659 | -1.191942 | -0.251243 |
| C | 0.433438 | -2.970931 | 0.035003 |  | H | 9.69089 | -2.268799 | -0.157976 |
| H | 1.042231 | -2.768749 | 0.912532 |  | C | -0.440215 | 0.523044 | 0.413614 |
| C | 6.245417 | 1.404328 | 3.321009 |  | C | -3.876925 | -0.655405 | -0.753985 |
| C | -3.879221 | -0.737277 | 0.678196 |  | H | -4.281675 | -1.331169 | -1.494936 |
| H | -4.27303 | -1.478186 | 1.358209 |  | C | 2.498156 | 0.538004 | -0.631142 |
| C | -0.749962 | -2.190534 | -1.920357 |  | H | 1.860439 | 1.127357 | -1.280285 |
| H | -1.056806 | -1.378818 | -2.573622 |  | C | -6.296817 | -3.120349 | -1.641957 |
| C | -2.500035 | -0.616988 | 0.557725 |  | C | -9.610626 | 1.192032 | -0.251288 |
| H | -1.864449 | -1.266101 | 1.14975 |  | H | -9.690836 | 2.268889 | -0.157986 |
| C | 0.785123 | 2.482383 | -1.412785 |  | C | -7.010586 | 3.076049 | 1.374787 |
| H | 1.097081 | 1.825356 | -2.219468 |  | C | 6.137583 | 3.189096 | 0.835321 |
| C | -6.15315 | 4.559095 | 0.560491 |  | C | -10.61862 | -1.015805 | -0.549833 |
| H | -5.812338 | 5.200621 | 1.367125 |  | H | -11.51912 | -1.608668 | -0.673525 |
| C | -6.802061 | 4.293588 | -1.747272 |  | C | 9.384191 | 1.655672 | -0.515631 |
| H | -6.958638 | 4.730223 | -2.728533 |  | H | 9.294182 | 2.731947 | -0.60621 |
| C | -6.137969 | -3.215736 | -0.661278 |  | C | -5.881214 | -4.457073 | -1.669802 |
| C | 5.981116 | -1.063194 | 3.251764 |  | H | -5.788726 | -4.964225 | -2.625024 |
| C | -0.427289 | 2.840643 | 0.644995 |  | C | 6.402468 | 2.510347 | -0.371808 |
| H | -1.051457 | 2.462651 | 1.450637 |  | C | 6.401013 | -3.166486 | -1.031168 |
| C | -6.340982 | 5.097302 | -0.708222 |  | C | 0.411669 | -2.920603 | -0.365905 |
| H | -6.139203 | 6.149471 | -0.886463 |  | H | 1.024475 | -2.628443 | -1.214795 |
| C | 0.066842 | -4.281847 | -0.265334 |  | C | 10.728792 | -0.385095 | -0.432462 |
| H | 0.386698 | -5.093126 | 0.382702 |  | H | 11.710941 | -0.844224 | -0.480249 |
| C | -0.699306 | -4.553484 | -1.401464 |  | C | 6.182246 | -4.542215 | -0.888457 |
| H | -0.970636 | -5.576915 | -1.644292 |  | H | 5.877142 | -5.123518 | -1.753058 |
| C | -9.612738 | 1.394551 | 0.225012 |  | C | 2.787044 | -1.103796 | 1.104211 |
| H | -10.47255 | 0.837222 | 0.602182 |  | H | 2.373753 | -1.79612 | 1.830282 |
| H | -9.863576 | 1.765818 | -0.773067 |  | C | 10.618596 | 1.015927 | -0.549722 |
| H | -9.464417 | 2.264393 | 0.872682 |  | H | 11.519083 | 1.608821 | -0.673376 |
| C | 6.67566 | 2.739851 | 2.709096 |  | C | -5.722233 | -4.522741 | 0.736876 |
| H | 7.163366 | 2.540788 | 1.748309 |  | H | -5.510968 | -5.0803 | 1.644109 |
| C | 6.920344 | -0.113591 | -2.28831 |  | C | -6.315305 | -2.557779 | 2.213099 |
| C | -6.304427 | -3.044133 | 1.809748 |  | H | -6.580947 | -1.503342 | 2.082294 |
| C | -7.595586 | 2.103435 | -2.72871 |  | C | 6.315211 | 2.557517 | 2.21322 |
| H | -7.855412 | 1.104639 | -2.360285 |  | H | 6.580997 | 1.503131 | 2.082312 |
| C | 5.463478 | -1.009974 | 4.552018 |  | C | -10.72878 | 0.38522 | -0.432552 |
| H | 5.172514 | -1.932537 | 5.044783 |  | H | -11.71091 | 0.84438 | -0.480361 |
| C | 9.664694 | 0.154518 | -1.128115 |  | C | -6.400978 | 3.166571 | -1.030973 |
| H | 9.577307 | 0.908704 | -1.916683 |  | C | 7.489322 | -2.330007 | 2.621802 |
| H | 9.886983 | -0.801753 | -1.610564 |  | H | 7.547898 | -1.261355 | 2.385738 |
| H | 10.517815 | 0.42119 | -0.500918 |  | C | -7.48924 | 2.3298 | 2.621938 |
| C | -6.336685 | -2.6559 | -2.0678 |  | H | -7.547874 | 1.26117 | 2.385778 |
| H | -6.686569 | -1.621404 | -1.9855 |  | C | -0.770551 | -2.349039 | 1.661934 |
| C | -9.347015 | -1.852431 | 0.670036 |  | H | -1.070641 | -1.611394 | 2.400638 |
| H | -10.32864 | -1.438969 | 0.429516 |  | C | -0.411669 | 2.920514 | -0.366047 |
| H | -9.370367 | -2.189014 | 1.711024 |  | H | -1.024457 | 2.628306 | -1.214933 |
| H | -9.189007 | -2.734379 | 0.041852 |  | C | 6.779859 | -4.455205 | 1.447603 |
| C | 5.716008 | 1.387978 | 4.61715 |  | H | 6.932623 | -4.970001 | 2.391037 |
| H | 5.616935 | 2.322254 | 5.160927 |  | C | 0.770542 | 2.349054 | 1.661827 |
| C | -5.822637 | -4.356051 | 1.896312 |  | H | 1.070615 | 1.611449 | 2.400579 |
| H | -5.706117 | -4.814376 | 2.873374 |  | C | 6.239805 | -2.521007 | -2.404899 |
| C | -1.100712 | -3.504623 | -2.230745 |  | H | 6.408274 | -1.443252 | -2.30674 |
| H | -1.678973 | -3.708904 | -3.127552 |  | C | -5.593551 | -5.150633 | -0.497796 |
| C | 5.324442 | 0.198774 | 5.226019 |  | H | -5.278225 | -6.18882 | -0.54739 |
| H | 4.921664 | 0.214101 | 6.234578 |  | C | 6.296779 | 3.120492 | -1.641778 |
| C | -5.500986 | -5.087626 | 0.756658 |  | C | 6.366111 | -5.180509 | 0.333916 |
| H | -5.133999 | -6.105329 | 0.852652 |  | H | 6.197069 | -6.250142 | 0.417379 |
| C | -5.665749 | -4.524813 | -0.50444 |  | C | -6.77971 | 4.455076 | 1.447924 |
| H | -5.431364 | -5.113103 | -1.386148 |  | H | -6.932401 | 4.969796 | 2.391411 |
| C | 9.288243 | 0.261909 | 2.138375 |  | C | -6.36599 | 5.18047 | 0.334285 |
| H | 9.089221 | -0.46755 | 2.929592 |  | H | -6.196917 | 6.250091 | 0.417843 |
| H | 9.31515 | 1.25254 | 2.602477 |  | C | 6.626035 | 2.411702 | -2.957189 |
| H | 10.280176 | 0.055165 | 1.731507 |  | H | 6.850874 | 1.361533 | -2.738395 |
| C | -6.213376 | 2.670408 | 2.238879 |  | C | 0.029896 | -4.252593 | -0.213636 |
| H | -6.44298 | 1.599486 | 2.240483 |  | H | 0.342721 | -4.990478 | -0.947117 |
| C | 6.144046 | -2.418162 | 2.566933 |  | C | -1.137697 | -3.685081 | 1.822793 |
| H | 6.535834 | -2.254013 | 1.557479 |  | H | -1.723449 | -3.981134 | 2.688509 |
| C | -6.52974 | 1.922504 | -3.830549 |  | C | -6.62598 | -2.411396 | -2.957306 |
| H | -6.208001 | 2.888904 | -4.233944 |  | H | -6.850756 | -1.361237 | -2.738411 |
| H | -6.944904 | 1.339408 | -4.659845 |  | C | 1.137675 | 3.685105 | 1.822626 |
| H | -5.641308 | 1.396386 | -3.466699 |  | H | 1.723422 | 3.981203 | 2.68833 |
| C | 1.155005 | 3.827413 | -1.427571 |  | C | -7.475122 | -3.223725 | 2.981801 |
| H | 1.753964 | 4.211706 | -2.248538 |  | H | -8.416128 | -3.157767 | 2.425397 |
| C | 0.747821 | 4.679342 | -0.399121 |  | H | -7.61871 | -2.733509 | 3.950956 |
| H | 1.035879 | 5.726645 | -0.410774 |  | H | -7.271716 | -4.283317 | 3.171261 |
| C | -0.043435 | 4.180734 | 0.638626 |  | C | 5.881117 | 4.457202 | -1.669489 |
| H | -0.366882 | 4.838207 | 1.440841 |  | H | 5.788642 | 4.964458 | -2.624655 |
| C | 7.12707 | -1.350267 | -2.941685 |  | C | -6.182194 | 4.542285 | -0.888156 |
| C | -4.755673 | 2.825903 | 2.716107 |  | H | -5.877116 | 5.123664 | -1.752715 |
| H | -4.055437 | 2.340104 | 2.028748 |  | C | 5.722073 | 4.5226 | 0.737195 |
| H | -4.633777 | 2.376017 | 3.707697 |  | H | 5.510758 | 5.080052 | 1.644482 |
| H | -4.472857 | 3.881343 | 2.796319 |  | C | -0.02991 | 4.252514 | -0.213837 |
| C | -5.017354 | -2.629108 | -2.86445 |  | H | -0.342735 | 4.990364 | -0.947353 |
| H | -4.238137 | -2.07213 | -2.334574 |  | C | 4.81596 | -2.707513 | -2.964824 |
| H | -5.177334 | -2.15784 | -3.84055 |  | H | 4.588767 | -3.764459 | -3.141603 |
| H | -4.641713 | -3.642362 | -3.045749 |  | H | 4.718162 | -2.186273 | -3.923504 |
| C | 6.323719 | 0.960376 | -4.346929 |  | H | 4.060189 | -2.309329 | -2.279288 |
| H | 6.032277 | 1.844694 | -4.905227 |  | C | -5.012201 | -2.596874 | 3.035194 |
| C | 6.483263 | -0.248463 | -5.016041 |  | H | -4.719457 | -3.625726 | 3.272599 |
| H | 6.307059 | -0.303216 | -6.086343 |  | H | -5.151451 | -2.069 | 3.985021 |
| C | -7.188565 | 3.342479 | 3.22712 |  | H | -4.183284 | -2.125689 | 2.496416 |
| H | -6.998852 | 4.417991 | 3.310222 |  | C | 8.902778 | -2.786939 | 3.040433 |
| H | -7.074415 | 2.90721 | 4.225951 |  | H | 8.911075 | -3.847373 | 3.314869 |
| H | -8.23191 | 3.212537 | 2.919121 |  | H | 9.240632 | -2.214886 | 3.911212 |
| C | 6.54204 | 1.062768 | -2.967227 |  | H | 9.632901 | -2.641626 | 2.238881 |
| C | -7.422885 | -3.43975 | -2.83281 |  | C | -0.744137 | -4.639544 | 0.883062 |
| H | -7.130386 | -4.483781 | -2.988186 |  | H | -1.028922 | -5.680163 | 1.009195 |
| H | -7.588872 | -2.991501 | -3.818551 |  | C | 0.744112 | 4.639522 | 0.882849 |
| H | -8.378202 | -3.438908 | -2.296541 |  | H | 1.028882 | 5.68015 | 1.008941 |
| C | -8.87238 | 2.717617 | -3.341293 |  | C | -8.902677 | 2.786744 | 3.040621 |
| H | -9.646879 | 2.904188 | -2.59036 |  | H | -8.910955 | 3.847175 | 3.31508 |
| H | -9.286931 | 2.042619 | -4.097469 |  | H | -9.240515 | 2.214678 | 3.911396 |
| H | -8.660852 | 3.671728 | -3.835443 |  | H | -9.63282 | 2.641455 | 2.239086 |
| C | 5.473157 | 3.666444 | 2.429283 |  | C | 5.593385 | 5.150617 | -0.497417 |
| H | 4.769321 | 3.229602 | 1.713817 |  | H | 5.278007 | 6.188794 | -0.546909 |
| H | 5.823351 | 4.617456 | 2.013207 |  | C | 7.298188 | -3.052985 | -3.393002 |
| H | 4.922899 | 3.889954 | 3.349927 |  | H | 8.314353 | -2.881321 | -3.022359 |
| C | 4.795613 | -3.150276 | 2.417273 |  | H | 7.199902 | -2.549224 | -4.360874 |
| H | 4.363057 | -3.39668 | 3.393256 |  | H | 7.181074 | -4.128575 | -3.56399 |
| H | 4.937315 | -4.090563 | 1.873163 |  | C | -6.516353 | 2.486146 | 3.809027 |
| H | 4.0684 | -2.542855 | 1.868599 |  | H | -5.50666 | 2.134623 | 3.570844 |
| C | -6.668095 | -2.299531 | 3.097022 |  | H | -6.877845 | 1.905831 | 4.664642 |
| H | -7.116867 | -1.336929 | 2.827026 |  | H | -6.437288 | 3.529841 | 4.130519 |
| C | 6.88643 | -1.385178 | -4.320568 |  | C | 7.474903 | 3.22353 | 2.982057 |
| H | 7.024368 | -2.317191 | -4.859871 |  | H | 8.415931 | 3.157839 | 2.425656 |
| C | 6.39234 | 2.420645 | -2.284816 |  | H | 7.618562 | 2.733164 | 3.951125 |
| H | 6.582509 | 2.295745 | -1.213444 |  | H | 7.271308 | 4.283054 | 3.171705 |
| C | 7.167343 | -3.299882 | 3.311863 |  | C | -6.23976 | 2.521192 | -2.404753 |
| H | 8.144122 | -2.80979 | 3.38993 |  | H | -6.408231 | 1.443435 | -2.306676 |
| H | 7.306528 | -4.24812 | 2.781304 |  | C | 7.876503 | 3.021509 | -3.625241 |
| H | 6.831494 | -3.534086 | 4.32776 |  | H | 7.710797 | 4.071022 | -3.891936 |
| C | 7.591373 | -2.626295 | -2.23397 |  | H | 8.115361 | 2.47781 | -4.545648 |
| H | 7.839373 | -2.377401 | -1.196075 |  | H | 8.75205 | 2.97516 | -2.971116 |
| C | 7.697144 | 3.474339 | 3.603282 |  | C | 5.442216 | 2.431173 | -3.946065 |
| H | 7.240907 | 3.80936 | 4.540792 |  | H | 4.539301 | 1.975717 | -3.525143 |
| H | 8.075195 | 4.363381 | 3.087561 |  | H | 5.705384 | 1.874811 | -4.852069 |
| H | 8.552206 | 2.842474 | 3.86407 |  | H | 5.187998 | 3.451768 | -4.250664 |
| C | -5.431084 | -1.999043 | 3.970022 |  | C | -5.44211 | -2.430836 | -3.946124 |
| H | -4.913289 | -2.921434 | 4.255093 |  | H | -4.539172 | -1.975541 | -3.525074 |
| H | -5.73876 | -1.493492 | 4.891876 |  | H | -5.70517 | -1.874306 | -4.852054 |
| H | -4.710639 | -1.350986 | 3.46094 |  | H | -5.187982 | -3.451408 | -4.250871 |
| C | -7.707914 | -3.080369 | 3.929074 |  | C | 5.012062 | 2.596343 | 3.035258 |
| H | -8.587341 | -3.359601 | 3.340208 |  | H | 4.7191 | 3.625131 | 3.272666 |
| H | -8.043498 | -2.47292 | 4.776313 |  | H | 5.151378 | 2.068479 | 3.985081 |
| H | -7.282283 | -4.003484 | 4.336556 |  | H | 4.183267 | 2.124992 | 2.496438 |
| C | 6.491326 | -3.708315 | -2.195444 |  | C | 6.516463 | -2.4865 | 3.808895 |
| H | 5.605877 | -3.381264 | -1.641212 |  | H | 5.506781 | -2.134876 | 3.570823 |
| H | 6.873369 | -4.611844 | -1.707912 |  | H | 6.878014 | -1.906341 | 4.664591 |
| H | 6.173251 | -3.986364 | -3.206203 |  | H | 6.437347 | -3.530241 | 4.130221 |
| C | 4.964836 | 2.983551 | -2.430706 |  | C | -7.876458 | -3.021047 | -3.625475 |
| H | 4.718697 | 3.179875 | -3.48029 |  | H | -7.710841 | -4.070568 | -3.892192 |
| H | 4.878959 | 3.931726 | -1.888441 |  | H | -8.115191 | -2.477294 | -4.545882 |
| H | 4.215961 | 2.293253 | -2.029967 |  | H | -8.75205 | -2.974624 | -2.971415 |
| C | 7.434273 | 3.426977 | -2.815264 |  | C | -4.815902 | 2.707745 | -2.964635 |
| H | 8.457996 | 3.060361 | -2.681432 |  | H | -4.588743 | 3.764703 | -3.141387 |
| H | 7.344552 | 4.380177 | -2.282836 |  | H | -4.71806 | 2.186526 | -3.923322 |
| H | 7.290994 | 3.627557 | -3.882439 |  | H | -4.060136 | 2.309577 | -2.279081 |
| C | 8.863946 | -3.207472 | -2.8866 |  | C | -7.298115 | 3.053251 | -3.392835 |
| H | 9.661907 | -2.463683 | -2.979439 |  | H | -8.314291 | 2.881509 | -3.022254 |
| H | 8.658879 | -3.593187 | -3.890794 |  | H | -7.199777 | 2.549607 | -4.360763 |
| H | 9.243846 | -4.041637 | -2.287353 |  | H | -7.181032 | 4.128863 | -3.563696 |

| **1c**^2+^ (B3LYP/6-31g*) | | | |  | **1d**^2+^ (B3LYP/6-31g*) | | | |
| --- | --- | --- | --- | --- | --- | --- | --- | --- |
| Atom | X | Y | Z |  | Atom | X | Y | Z |
| F | -9.686555 | 2.52535 | 0.751447 |  | N | -6.881245 | -1.161577 | 0.123045 |
| F | 9.233833 | -0.841663 | 3.049981 |  | N | -7.066649 | 1.057312 | 0.136837 |
| F | 9.802111 | 0.923557 | -2.140885 |  | N | -9.269177 | -1.686796 | 0.279363 |
| F | -9.35065 | -2.914875 | -0.073875 |  | C | -4.733112 | 0.137265 | -0.043728 |
| F | 11.962095 | 0.557096 | -0.532959 |  | C | -0.434068 | 0.598754 | -0.306052 |
| F | -11.74519 | -1.697785 | 0.3464 |  | N | -9.503719 | 1.17806 | 0.350868 |
| F | 11.684883 | -0.31927 | 2.00022 |  | C | -1.9119 | 0.397911 | -0.240714 |
| F | -11.90695 | 0.958758 | 0.764232 |  | C | -6.186846 | 0.013847 | 0.068547 |
| N | -7.053741 | 1.098086 | 0.197718 |  | C | -8.2378 | -0.858692 | 0.220912 |
| N | 6.860513 | -0.336961 | 1.206698 |  | C | 0.003339 | 2.023113 | -0.18297 |
| N | 7.099144 | 0.237799 | -0.917234 |  | C | -6.382519 | -2.522104 | -0.037003 |
| N | -6.905175 | -1.100957 | 0.000048 |  | C | -8.352842 | 0.532644 | 0.242383 |
| C | -6.729612 | 2.516291 | 0.325152 |  | C | -6.782214 | 2.484531 | 0.229983 |
| C | 8.22914 | -0.201471 | 0.974489 |  | C | -3.875513 | -0.754111 | 0.631698 |
| C | -8.349311 | 0.592914 | 0.301039 |  | H | -4.283452 | -1.540707 | 1.252231 |
| C | 6.291472 | -0.770163 | 2.479648 |  | C | -2.77589 | 1.289169 | -0.90497 |
| C | 6.192822 | -0.076139 | 0.049007 |  | H | -2.35775 | 2.091655 | -1.503731 |
| C | 8.378077 | 0.192483 | -0.362799 |  | C | -6.277779 | -3.334373 | 1.111011 |
| C | -6.923975 | 3.351317 | -0.796349 |  | C | -6.992744 | 3.283511 | -0.913058 |
| C | -9.579745 | 1.210756 | 0.541986 |  | C | -6.113186 | -2.981167 | -1.341323 |
| C | 5.985748 | -2.135847 | 2.635854 |  | C | -4.155286 | 1.162448 | -0.818046 |
| C | 9.639045 | 0.485434 | -0.889857 |  | H | -4.780793 | 1.858926 | -1.359602 |
| C | -6.190295 | 0.058311 | 0.024329 |  | C | -6.38799 | 3.003313 | 1.478968 |
| C | -8.258518 | -0.796987 | 0.1455 |  | C | -2.496899 | -0.620821 | 0.53467 |
| C | -6.29724 | 2.987088 | 1.579606 |  | H | -1.862723 | -1.310539 | 1.08006 |
| C | 9.344518 | -0.410893 | 1.790603 |  | C | -6.645554 | -2.863607 | 2.517123 |
| C | 10.598568 | -0.144681 | 1.257361 |  | H | -6.853221 | -1.787162 | 2.479485 |
| C | 4.736507 | -0.131357 | -0.128601 |  | C | -6.3202 | -2.134723 | -2.594235 |
| C | -10.63011 | -0.977369 | 0.343162 |  | H | -6.559087 | -1.109223 | -2.290352 |
| C | 10.744556 | 0.300689 | -0.070453 |  | C | 0.800313 | 2.620485 | -1.173773 |
| C | -9.40562 | -1.595744 | 0.128445 |  | H | 1.117234 | 2.031954 | -2.029952 |
| C | 6.131684 | 0.186476 | 3.505199 |  | C | -6.15788 | 4.382711 | 1.546329 |
| C | -4.733007 | 0.170668 | -0.107988 |  | H | -5.853055 | 4.824138 | 2.490058 |
| C | -7.475557 | 2.863886 | -2.137065 |  | C | -6.751617 | 4.655481 | -0.777004 |
| H | -7.581691 | 1.773012 | -2.093324 |  | H | -6.904483 | 5.30832 | -1.630524 |
| C | -10.7161 | 0.413063 | 0.548996 |  | C | -5.843574 | -4.650467 | 0.915498 |
| C | -6.005495 | 4.353133 | 1.676459 |  | H | -5.752053 | -5.311895 | 1.771318 |
| H | -5.671463 | 4.758463 | 2.626335 |  | C | -10.44429 | -1.051535 | 0.379027 |
| C | 6.269189 | -3.18776 | 1.567563 |  | H | -11.33346 | -1.672889 | 0.429804 |
| H | 6.604812 | -2.679246 | 0.656537 |  | C | -0.4251 | 2.811496 | 0.899643 |
| C | 6.823027 | 0.642487 | -2.292971 |  | H | -1.05453 | 2.369939 | 1.668025 |
| C | -6.619332 | 4.707273 | -0.630913 |  | C | 0.754611 | 4.728439 | 0.014255 |
| H | -6.756082 | 5.387407 | -1.465728 |  | H | 1.041399 | 5.773422 | 0.088378 |
| C | 6.967744 | -0.315344 | -3.319818 |  | C | -5.543468 | -5.131291 | -0.355674 |
| C | 5.452037 | -2.528146 | 3.869489 |  | H | -5.215098 | -6.158973 | -0.481078 |
| H | 5.204966 | -3.572379 | 4.032976 |  | C | -7.929743 | -3.56564 | 3.007406 |
| C | -4.153268 | 1.21755 | -0.849761 |  | H | -8.755945 | -3.419373 | 2.305967 |
| H | -4.776435 | 1.941126 | -1.355928 |  | H | -8.227348 | -3.171537 | 3.98542 |
| C | -6.15781 | 5.20287 | 0.585521 |  | H | -7.768314 | -4.643643 | 3.117302 |
| H | -5.93188 | 6.260281 | 0.687281 |  | C | -0.042069 | 4.147657 | 1.004057 |
| C | -6.402038 | -2.455949 | -0.205645 |  | H | -0.370891 | 4.738045 | 1.854636 |
| C | -6.259779 | -3.29595 | 0.919861 |  | C | -6.333446 | 5.199784 | 0.433954 |
| C | 6.551142 | 1.651477 | 3.375429 |  | H | -6.157045 | 6.268448 | 0.514088 |
| H | 6.847398 | 1.836946 | 2.335652 |  | C | -5.680497 | -4.306599 | -1.467804 |
| C | 2.490459 | 0.334501 | 0.674008 |  | H | -5.465544 | -4.702229 | -2.455428 |
| H | 1.846095 | 0.754408 | 1.43825 |  | C | -10.55892 | 0.356061 | 0.42198 |
| C | -2.773233 | 1.337092 | -0.946527 |  | H | -11.53597 | 0.821082 | 0.513783 |
| H | -2.355776 | 2.160103 | -1.517427 |  | C | -7.493162 | 2.735173 | -2.24833 |
| C | -6.205517 | 2.105592 | 2.821383 |  | H | -7.518447 | 1.640265 | -2.18582 |
| H | -6.379783 | 1.064882 | 2.525156 |  | C | 1.16854 | 3.962641 | -1.077368 |
| C | 6.704079 | 0.117376 | -4.624474 |  | H | 1.770698 | 4.412794 | -1.861471 |
| H | 6.805331 | -0.583637 | -5.446803 |  | C | -6.253166 | 2.156773 | 2.741587 |
| C | 3.867568 | 0.395621 | 0.845403 |  | H | -6.389019 | 1.102619 | 2.474072 |
| H | 4.264361 | 0.857383 | 1.738733 |  | C | -5.500923 | -3.068788 | 3.529209 |
| C | 6.482351 | 1.988404 | -2.527717 |  | H | -5.265779 | -4.129787 | 3.663377 |
| C | 0.442601 | -0.414537 | -0.622539 |  | H | -5.793818 | -2.672853 | 4.507512 |
| C | 5.597729 | -0.271863 | 4.715019 |  | H | -4.579775 | -2.559469 | 3.22378 |
| H | 5.459787 | 0.427844 | 5.533313 |  | C | -7.362213 | 2.510868 | 3.754237 |
| C | -5.797975 | -4.595239 | 0.680854 |  | H | -7.296421 | 1.860114 | 4.633211 |
| H | -5.676125 | -5.276902 | 1.516791 |  | H | -8.35753 | 2.394657 | 3.312585 |
| C | -1.909222 | 0.416069 | -0.325489 |  | H | -7.269743 | 3.546738 | 4.098054 |
| C | 4.17443 | -0.73039 | -1.27079 |  | C | -5.050987 | -2.058023 | -3.463586 |
| H | 4.811335 | -1.150141 | -2.03682 |  | H | -4.194946 | -1.683828 | -2.891749 |
| C | -6.537637 | 3.182507 | -3.319032 |  | H | -5.218533 | -1.387161 | -4.313308 |
| H | -6.967827 | 2.794986 | -4.248692 |  | H | -4.782765 | -3.039042 | -3.87066 |
| H | -5.545625 | 2.733771 | -3.198622 |  | C | -4.856147 | 2.280492 | 3.378702 |
| H | -6.402162 | 4.261545 | -3.446197 |  | H | -4.660653 | 3.300573 | 3.726062 |
| C | 6.329395 | 1.431633 | -4.890351 |  | H | -4.067018 | 2.008287 | 2.669234 |
| H | 6.133984 | 1.74123 | -5.912859 |  | H | -4.77988 | 1.61822 | 4.248068 |
| C | 1.920055 | -0.26858 | -0.461669 |  | C | -6.565596 | 3.09981 | -3.424519 |
| C | 6.227789 | 2.354983 | -3.854833 |  | H | -6.523598 | 4.182006 | -3.585826 |
| H | 5.965107 | 3.383758 | -4.080669 |  | H | -6.940589 | 2.646318 | -4.348262 |
| C | -6.146196 | -2.874273 | -1.525381 |  | H | -5.539775 | 2.746071 | -3.270465 |
| C | 2.795812 | -0.811774 | -1.419966 |  | C | -7.523418 | -2.655424 | -3.408102 |
| H | 2.389199 | -1.304507 | -2.29707 |  | H | -7.341905 | -3.670258 | -3.778421 |
| C | 7.439846 | -1.751405 | -3.085428 |  | H | -7.703571 | -2.01042 | -4.275189 |
| H | 7.481974 | -1.931325 | -2.004007 |  | H | -8.434807 | -2.680922 | -2.801502 |
| C | 8.864042 | -1.955041 | -3.647953 |  | C | -8.932922 | 3.215639 | -2.528782 |
| H | 8.866627 | -1.868994 | -4.740034 |  | H | -9.606126 | 2.955677 | -1.707069 |
| H | 9.233118 | -2.954054 | -3.392029 |  | H | -9.312488 | 2.759044 | -3.449616 |
| H | 9.567039 | -1.214231 | -3.259522 |  | H | -8.964453 | 4.302981 | -2.658905 |
| C | -5.684706 | -4.185327 | -1.695123 |  | N | 7.088929 | 0.151941 | -0.910038 |
| H | -5.478855 | -4.549579 | -2.696598 |  | N | 6.869413 | -0.219701 | 1.274395 |
| C | 5.254269 | -1.609108 | 4.894956 |  | N | 9.533854 | 0.30174 | -0.968425 |
| H | 4.846299 | -1.938853 | 5.845983 |  | C | 4.738565 | -0.113938 | -0.057445 |
| C | -8.876717 | 3.456854 | -2.402579 |  | C | 0.443476 | -0.432257 | -0.497872 |
| H | -8.822463 | 4.542527 | -2.538236 |  | N | 9.25261 | -0.235965 | 1.84215 |
| H | -9.565309 | 3.263154 | -1.576287 |  | C | 1.921013 | -0.273035 | -0.35483 |
| H | -9.30124 | 3.029253 | -3.3173 |  | C | 6.192859 | -0.056668 | 0.099624 |
| C | -3.874818 | -0.752901 | 0.520175 |  | C | 8.369937 | 0.125751 | -0.362461 |
| H | -4.280875 | -1.562256 | 1.110664 |  | C | 0.005708 | -1.80228 | -0.906353 |
| C | -5.50721 | -5.034603 | -0.607765 |  | C | 6.817378 | 0.516225 | -2.295771 |
| H | -5.155985 | -6.050128 | -0.765702 |  | C | 8.23295 | -0.120507 | 1.005472 |
| C | -6.618308 | -2.877652 | 2.346698 |  | C | 6.334446 | -0.567494 | 2.585132 |
| H | -6.886516 | -1.814177 | 2.340195 |  | C | 4.167271 | -0.763684 | -1.168148 |
| C | -4.814284 | 2.157369 | 3.479551 |  | H | 4.798318 | -1.217477 | -1.920466 |
| H | -4.580452 | 3.160014 | 3.853107 |  | C | 2.500407 | 0.382177 | 0.747785 |
| H | -4.027465 | 1.866544 | 2.77491 |  | H | 1.861927 | 0.83427 | 1.49842 |
| H | -4.778392 | 1.473768 | 4.334857 |  | C | 6.975218 | -0.467664 | -3.293472 |
| C | -0.430285 | 0.611556 | -0.388877 |  | C | 6.243943 | 0.447425 | 3.560109 |
| C | 5.410399 | 2.635111 | 3.705601 |  | C | 6.485551 | 1.857496 | -2.572057 |
| H | 4.532869 | 2.491245 | 3.065857 |  | C | 3.878621 | 0.452708 | 0.903879 |
| H | 5.757634 | 3.664494 | 3.566637 |  | H | 4.284077 | 0.952766 | 1.773568 |
| H | 5.083677 | 2.538553 | 4.746129 |  | C | 6.003873 | -1.914489 | 2.831419 |
| C | 5.014859 | -3.999015 | 1.19249 |  | C | 2.788259 | -0.856317 | -1.297907 |
| H | 5.254657 | -4.71204 | 0.396195 |  | H | 2.37343 | -1.389131 | -2.147185 |
| H | 4.207779 | -3.348805 | 0.837688 |  | C | 7.430505 | -1.897482 | -3.005457 |
| H | 4.636951 | -4.574932 | 2.043984 |  | H | 7.426509 | -2.049747 | -1.918839 |
| C | -0.000346 | -1.763516 | -1.090477 |  | C | 6.433498 | 2.951175 | -1.508769 |
| C | 0.014767 | 2.027417 | -0.20637 |  | H | 6.55696 | 2.489303 | -0.522577 |
| C | -6.409937 | -2.002059 | -2.749028 |  | C | 0.422576 | -2.932154 | -0.180617 |
| H | -6.665895 | -0.991775 | -2.410188 |  | H | 1.042425 | -2.802894 | 0.702963 |
| C | 6.458396 | 3.051644 | -1.433562 |  | C | 5.515637 | -2.221841 | 4.10713 |
| H | 6.598636 | 2.559527 | -0.464445 |  | H | 5.249249 | -3.248036 | 4.340141 |
| C | 7.419376 | -4.112653 | 2.019047 |  | C | 5.75428 | 0.072977 | 4.816684 |
| H | 7.133193 | -4.702392 | 2.896652 |  | H | 5.670666 | 0.821175 | 5.598585 |
| H | 8.313349 | -3.538342 | 2.284694 |  | C | 6.736118 | -0.070608 | -4.614262 |
| H | 7.682934 | -4.812007 | 1.218188 |  | H | 6.848655 | -0.794203 | -5.415244 |
| C | -2.495624 | -0.627836 | 0.413341 |  | C | 10.577865 | 0.198272 | -0.135659 |
| H | -1.862713 | -1.344988 | 0.923881 |  | H | 11.564445 | 0.332603 | -0.569146 |
| C | -7.315172 | 2.483133 | 3.825637 |  | C | -0.779694 | -1.994682 | -2.05514 |
| H | -7.307783 | 1.797593 | 4.680098 |  | H | -1.086319 | -1.133471 | -2.641825 |
| H | -8.305409 | 2.443743 | 3.3592 |  | C | -0.745447 | -4.390234 | -1.716727 |
| H | -7.172162 | 3.498766 | 4.210237 |  | H | -1.031005 | -5.38966 | -2.031964 |
| C | -5.171245 | -1.87037 | -3.654527 |  | C | 6.371745 | 1.237146 | -4.921581 |
| H | -4.888515 | -2.833669 | -4.09287 |  | H | 6.195272 | 1.520314 | -5.955173 |
| H | -4.30901 | -1.484815 | -3.099835 |  | C | 8.877618 | -2.112998 | -3.497823 |
| H | -5.382815 | -1.185232 | -4.482709 |  | H | 9.560142 | -1.378214 | -3.061621 |
| C | 7.775303 | 1.949795 | 4.268896 |  | H | 9.224587 | -3.116623 | -3.227817 |
| H | 7.514472 | 1.860549 | 5.329125 |  | H | 8.936129 | -2.021569 | -4.588021 |
| H | 8.128832 | 2.972321 | 4.098161 |  | C | -1.148357 | -3.278151 | -2.458339 |
| H | 8.599846 | 1.259474 | 4.07514 |  | H | -1.74234 | -3.40883 | -3.358462 |
| C | -7.846087 | -3.663372 | 2.857138 |  | C | 5.387187 | -1.242317 | 5.086856 |
| H | -8.695754 | -3.580414 | 2.175007 |  | H | 5.01414 | -1.507925 | 6.071726 |
| H | -8.151537 | -3.291247 | 3.840987 |  | C | 6.254625 | 2.18804 | -3.912691 |
| H | -7.612089 | -4.728317 | 2.962812 |  | H | 5.998382 | 3.210842 | -4.170715 |
| C | 5.114642 | 3.799817 | -1.366012 |  | C | 10.440142 | -0.074111 | 1.243996 |
| H | 5.130275 | 4.522981 | -0.543211 |  | H | 11.320743 | -0.158578 | 1.873813 |
| H | 4.277994 | 3.113082 | -1.199751 |  | C | 6.68795 | 1.889792 | 3.322275 |
| H | 4.918256 | 4.358159 | -2.2879 |  | H | 6.928686 | 2.00744 | 2.258745 |
| C | 6.487066 | -2.80473 | -3.68601 |  | C | 0.03965 | -4.213076 | -0.574873 |
| H | 5.470028 | -2.729663 | -3.286134 |  | H | 0.359904 | -5.074119 | 0.004978 |
| H | 6.858993 | -3.810137 | -3.46199 |  | C | 6.20197 | -3.032873 | 1.812352 |
| H | 6.423232 | -2.716121 | -4.775409 |  | H | 6.521406 | -2.589519 | 0.862283 |
| C | 0.814138 | 2.661156 | -1.172329 |  | C | 6.492462 | -2.957953 | -3.615062 |
| H | 1.127318 | 2.106703 | -2.052278 |  | H | 6.487177 | -2.913092 | -4.709048 |
| C | -0.791607 | -1.901723 | -2.243127 |  | H | 6.831139 | -3.960161 | -3.331834 |
| H | -1.097957 | -1.013958 | -2.788892 |  | H | 5.457191 | -2.845425 | -3.273334 |
| C | -0.408195 | 2.772562 | 0.908456 |  | C | 7.328761 | -3.984905 | 2.264952 |
| H | -1.039882 | 2.30276 | 1.657989 |  | H | 7.50881 | -4.750386 | 1.502057 |
| C | -7.624724 | -2.537665 | -3.536326 |  | H | 8.264252 | -3.441976 | 2.436 |
| H | -8.507816 | -2.629501 | -2.894941 |  | H | 7.065934 | -4.497341 | 3.196698 |
| H | -7.418364 | -3.527828 | -3.957019 |  | C | 5.08168 | 3.688731 | -1.48997 |
| H | -7.868952 | -1.865743 | -4.366332 |  | H | 4.247083 | 2.998886 | -1.326497 |
| C | -1.166687 | -3.164421 | -2.70191 |  | H | 5.07193 | 4.431353 | -0.684562 |
| H | -1.76537 | -3.252544 | -3.60417 |  | H | 4.902093 | 4.222904 | -2.429438 |
| C | 0.416067 | -2.926501 | -0.418604 |  | C | 4.898461 | -3.804565 | 1.531708 |
| H | 1.040085 | -2.839032 | 0.467107 |  | H | 4.535626 | -4.321253 | 2.426717 |
| C | 0.781566 | 4.718123 | 0.10245 |  | H | 4.104276 | -3.13651 | 1.180836 |
| H | 1.074151 | 5.757562 | 0.21958 |  | H | 5.070707 | -4.564831 | 0.762007 |
| C | 7.636952 | 4.031091 | -1.619092 |  | C | 5.585047 | 2.915109 | 3.651141 |
| H | 7.531582 | 4.605819 | -2.545583 |  | H | 5.314529 | 2.892671 | 4.711838 |
| H | 8.5935 | 3.49997 | -1.667788 |  | H | 5.939138 | 3.926819 | 3.42607 |
| H | 7.677358 | 4.742097 | -0.786732 |  | H | 4.671621 | 2.743781 | 3.070015 |
| C | 1.189901 | 3.996181 | -1.020687 |  | C | 7.605277 | 3.938426 | -1.692817 |
| H | 1.793393 | 4.475445 | -1.786319 |  | H | 7.530417 | 4.468322 | -2.648506 |
| C | -5.442486 | -3.044855 | 3.330342 |  | H | 7.599679 | 4.687709 | -0.893482 |
| H | -5.137064 | -4.092727 | 3.417534 |  | H | 8.570163 | 3.420947 | -1.673921 |
| H | -5.742623 | -2.70733 | 4.328125 |  | C | 7.970921 | 2.199714 | 4.122596 |
| H | -4.56251 | -2.464271 | 3.032931 |  | H | 8.766997 | 1.487369 | 3.887804 |
| C | 0.027801 | -4.187171 | -0.868885 |  | H | 8.326506 | 3.210071 | 3.892018 |
| H | 0.348323 | -5.074587 | -0.330379 |  | H | 7.781617 | 2.150766 | 5.200599 |
| C | -0.763178 | -4.310196 | -2.013809 |  |  |  |  |  |
| H | -1.052734 | -5.293553 | -2.372975 |  |  |  |  |  |
| C | -0.017499 | 4.101058 | 1.06808 |  |  |  |  |  |
| H | -0.342182 | 4.657464 | 1.94285 |  |  |  |  |  |

| **2a** (UBHandHLYP/def2-SVP)  Closed-shell singlet  *E* = -3474.262464 Hartree | | | |  | **2a** (UBHandHLYP/def2-SVP)  Open-shell triplet  *E* = -3474.253314 Hartree | | | |
| --- | --- | --- | --- | --- | --- | --- | --- | --- |
| Atom | X | Y | Z |  | Atom | X | Y | Z |
| N | 9.480977 | 16.075306 | 20.292631 |  | N | 8.927365 | 16.330035 | 21.143772 |
| N | 10.642191 | 14.279014 | 20.731314 |  | N | 10.67218 | 14.995347 | 20.892434 |
| N | 3.702373 | 4.956246 | 14.650937 |  | N | 2.897709 | 4.807522 | 14.898512 |
| N | 3.443631 | 6.666887 | 13.317902 |  | N | 3.426733 | 6.05925 | 13.159662 |
| C | 5.111444 | 9.917009 | 18.878821 |  | C | 6.037426 | 9.849455 | 18.103212 |
| C | 4.814844 | 9.026384 | 17.866046 |  | C | 5.384 | 8.885515 | 17.189767 |
| C | 8.531885 | 17.026791 | 19.810375 |  | C | 7.697051 | 17.027292 | 20.96696 |
| C | 4.544919 | 7.616344 | 18.053682 |  | C | 4.747554 | 7.728243 | 17.671615 |
| H | 4.5508 | 7.212453 | 19.057198 |  | H | 4.716503 | 7.549134 | 18.741275 |
| C | 4.700088 | 9.447981 | 16.486179 |  | C | 5.396554 | 9.033653 | 15.79303 |
| H | 4.913249 | 10.481593 | 16.249336 |  | H | 5.892852 | 9.893779 | 15.358659 |
| C | 8.51276 | 17.33707 | 18.44268 |  | C | 7.448695 | 17.689879 | 19.754792 |
| C | 5.431683 | 9.463311 | 20.251926 |  | C | 6.857051 | 9.229914 | 19.183944 |
| C | 11.435255 | 15.385095 | 21.063535 |  | C | 11.104603 | 16.159673 | 21.541626 |
| C | 6.078219 | 12.212617 | 18.989808 |  | C | 6.78522 | 12.166571 | 18.682523 |
| C | 4.118446 | 7.204541 | 15.66512 |  | C | 4.140352 | 6.948814 | 15.417044 |
| C | 4.384307 | 8.608649 | 15.473987 |  | C | 4.816405 | 8.121433 | 14.943863 |
| H | 4.360255 | 9.032089 | 14.483886 |  | H | 4.894839 | 8.319052 | 13.888793 |
| C | 5.025102 | 11.383763 | 18.656669 |  | C | 5.914379 | 11.198721 | 17.978245 |
| C | 7.398789 | 11.692943 | 19.273258 |  | C | 8.18087 | 12.02228 | 18.752575 |
| H | 7.567503 | 10.632282 | 19.144449 |  | H | 8.645719 | 11.160306 | 18.288178 |
| C | 9.413991 | 14.694114 | 20.249023 |  | C | 9.297737 | 15.078143 | 20.636447 |
| C | 4.093346 | 4.074972 | 15.704254 |  | C | 2.780077 | 4.186957 | 16.175625 |
| C | 10.725992 | 16.488172 | 20.786115 |  | C | 10.049553 | 16.975299 | 21.680744 |
| C | 3.101552 | 3.492976 | 16.510749 |  | C | 1.607617 | 4.381031 | 16.923981 |
| C | 3.780162 | 6.336852 | 14.617748 |  | C | 3.533661 | 6.007709 | 14.555313 |
| C | 12.331641 | 12.959957 | 18.762726 |  | C | 12.45905 | 14.781387 | 18.605496 |
| H | 12.024449 | 14.006678 | 18.802198 |  | H | 11.833257 | 15.620678 | 18.913879 |
| C | 11.088723 | 12.949865 | 21.004033 |  | C | 11.51773 | 13.851337 | 20.799106 |
| C | 4.22073 | 6.781042 | 17.038854 |  | C | 4.144716 | 6.811327 | 16.843965 |
| H | 4.002854 | 5.760194 | 17.305126 |  | H | 3.669992 | 5.96718 | 17.313348 |
| C | 8.343578 | 13.896562 | 19.82069 |  | C | 8.474544 | 14.121338 | 20.00296 |
| C | 8.44117 | 12.467945 | 19.648856 |  | C | 8.991673 | 12.943334 | 19.372762 |
| H | 9.38309 | 11.968574 | 19.801264 |  | H | 10.051101 | 12.752178 | 19.36032 |
| C | 11.932941 | 12.313937 | 20.079144 |  | C | 12.405695 | 13.744801 | 19.715042 |
| C | 7.674264 | 17.658239 | 20.725924 |  | C | 6.77849 | 17.07076 | 22.029415 |
| C | 3.24951 | 7.964737 | 12.753907 |  | C | 3.835602 | 7.090719 | 12.265516 |
| C | 5.989427 | 13.649456 | 19.142196 |  | C | 6.261847 | 13.324893 | 19.283638 |
| H | 5.033534 | 14.133173 | 18.992114 |  | H | 5.190913 | 13.49644 | 19.256197 |
| C | 12.795978 | 15.235088 | 21.638327 |  | C | 12.513578 | 16.337745 | 21.976162 |
| H | 12.776769 | 14.65704 | 22.566083 |  | H | 12.827382 | 15.54282 | 22.658324 |
| H | 13.219101 | 16.214858 | 21.858646 |  | H | 12.630889 | 17.290795 | 22.491306 |
| H | 13.473897 | 14.718961 | 20.954499 |  | H | 13.206135 | 16.323257 | 21.130958 |
| C | 4.265304 | 8.518191 | 11.95714 |  | C | 5.083756 | 6.988256 | 11.629404 |
| C | 11.089853 | 17.922371 | 20.908613 |  | C | 9.96774 | 18.341308 | 22.25795 |
| H | 11.024537 | 18.435697 | 19.945522 |  | H | 9.598701 | 19.065762 | 21.526685 |
| H | 12.111706 | 18.01872 | 21.274673 |  | H | 10.952571 | 18.668812 | 22.590053 |
| H | 10.43122 | 18.454149 | 21.599052 |  | H | 9.291273 | 18.382692 | 23.115267 |
| C | 3.161688 | 5.510338 | 12.579205 |  | C | 2.746033 | 4.930077 | 12.68898 |
| C | 10.708993 | 12.334813 | 22.205788 |  | C | 11.473896 | 12.87955 | 21.810667 |
| C | 3.330086 | 4.459369 | 13.394322 |  | C | 2.429147 | 4.166742 | 13.744847 |
| C | 7.031912 | 14.420642 | 19.531692 |  | C | 7.05051 | 14.251671 | 19.922808 |
| H | 6.833093 | 15.472394 | 19.652286 |  | H | 6.553822 | 15.097021 | 20.367505 |
| C | 5.454052 | 3.781773 | 15.876937 |  | C | 3.815466 | 3.356993 | 16.633879 |
| C | 4.934573 | 10.155558 | 21.36605 |  | C | 6.697744 | 9.603029 | 20.520638 |
| H | 4.308328 | 11.025158 | 21.203925 |  | H | 5.988453 | 10.383674 | 20.768792 |
| C | 3.733491 | 11.91613 | 18.165415 |  | C | 4.877703 | 11.813834 | 17.1002 |
| C | 3.653042 | 12.956379 | 17.227954 |  | C | 5.213041 | 12.835173 | 16.207238 |
| H | 4.568592 | 13.380923 | 16.832772 |  | H | 6.2461 | 13.157616 | 16.1395 |
| C | 2.432653 | 13.432902 | 16.773326 |  | C | 4.249515 | 13.435266 | 15.410091 |
| H | 2.410863 | 14.236374 | 16.043455 |  | H | 4.537326 | 14.218316 | 14.716154 |
| C | 1.244798 | 12.880087 | 17.233004 |  | C | 2.921262 | 13.041889 | 15.503138 |
| H | 0.289416 | 13.252616 | 16.87859 |  | H | 2.164687 | 13.516707 | 14.887372 |
| C | 1.300452 | 11.834089 | 18.146021 |  | C | 2.568964 | 12.041289 | 16.399757 |
| H | 0.382988 | 11.38471 | 18.512995 |  | H | 1.532533 | 11.733257 | 16.49037 |
| C | 2.521276 | 11.357686 | 18.596858 |  | C | 3.536251 | 11.433574 | 17.186099 |
| H | 2.546998 | 10.533788 | 19.300596 |  | H | 3.253894 | 10.652699 | 17.882165 |
| C | 6.267576 | 8.366811 | 20.50991 |  | C | 7.765649 | 8.208879 | 18.892573 |
| H | 6.712496 | 7.835723 | 19.676545 |  | H | 7.888972 | 7.892007 | 17.862912 |
| C | 5.59674 | 7.820492 | 11.733393 |  | C | 6.0441 | 5.847426 | 11.915391 |
| H | 5.504332 | 6.80204 | 12.115101 |  | H | 5.517556 | 5.128993 | 12.545876 |
| C | 3.509017 | 2.625243 | 17.519661 |  | C | 1.502534 | 3.741861 | 18.15621 |
| H | 2.766276 | 2.171543 | 18.165717 |  | H | 0.611915 | 3.884464 | 18.757873 |
| C | 9.439016 | 16.668731 | 17.443253 |  | C | 8.436237 | 17.667682 | 18.602429 |
| H | 10.077968 | 15.977141 | 17.994118 |  | H | 9.303517 | 17.088253 | 18.921097 |
| C | 12.373132 | 11.027854 | 20.376542 |  | C | 13.230601 | 12.625534 | 19.651345 |
| H | 13.015647 | 10.507532 | 19.675557 |  | H | 13.916022 | 12.513972 | 18.818738 |
| C | 7.66677 | 17.315293 | 22.20614 |  | C | 7.021056 | 16.342747 | 23.340667 |
| H | 8.524938 | 16.668981 | 22.399043 |  | H | 8.049151 | 15.977102 | 23.325233 |
| C | 7.592233 | 18.283594 | 18.001085 |  | C | 6.246456 | 18.37836 | 19.616792 |
| H | 7.548813 | 18.533982 | 16.947085 |  | H | 6.028238 | 18.889409 | 18.685668 |
| C | 1.624944 | 3.813081 | 16.346363 |  | C | 0.487144 | 5.288804 | 16.448573 |
| H | 1.511027 | 4.379258 | 15.41995 |  | H | 0.72473 | 5.592709 | 15.427696 |
| C | 2.035461 | 8.630779 | 12.975829 |  | C | 2.962365 | 8.157887 | 12.00398 |
| C | 4.848973 | 2.340105 | 17.720674 |  | C | 2.520499 | 2.932132 | 18.630438 |
| H | 5.145919 | 1.667444 | 18.518045 |  | H | 2.422064 | 2.446436 | 19.595362 |
| C | 6.720776 | 8.497588 | 12.52048 |  | C | 7.265699 | 6.329594 | 12.699376 |
| H | 6.871388 | 9.527938 | 12.184161 |  | H | 7.853006 | 7.045618 | 12.116075 |
| H | 7.662653 | 7.959574 | 12.376342 |  | H | 7.918636 | 5.486092 | 12.943516 |
| H | 6.506474 | 8.523839 | 13.590414 |  | H | 6.975376 | 6.815188 | 13.633107 |
| C | 6.775168 | 18.599599 | 20.233378 |  | C | 5.592801 | 17.775888 | 21.843146 |
| H | 6.092733 | 19.094325 | 20.914991 |  | H | 4.863261 | 17.8157 | 22.644128 |
| C | 2.730251 | 5.566814 | 11.159555 |  | C | 2.470381 | 4.725887 | 11.243874 |
| H | 1.824802 | 6.167911 | 11.041118 |  | H | 1.874489 | 5.542377 | 10.827333 |
| H | 2.520367 | 4.562492 | 10.792474 |  | H | 1.922099 | 3.796282 | 11.093256 |
| H | 3.494084 | 6.010048 | 10.516518 |  | H | 3.391367 | 4.674764 | 10.657533 |
| C | 5.808687 | 2.911095 | 16.903753 |  | C | 3.663444 | 2.741479 | 17.873252 |
| H | 6.85481 | 2.677389 | 17.066528 |  | H | 4.454288 | 2.104564 | 18.253634 |
| C | 13.842662 | 12.936694 | 18.534991 |  | C | 13.872641 | 15.316661 | 18.378554 |
| H | 14.389003 | 13.382104 | 19.370462 |  | H | 14.314248 | 15.70773 | 19.29863 |
| H | 14.096733 | 13.49596 | 17.630069 |  | H | 13.857594 | 16.125141 | 17.641967 |
| H | 14.217356 | 11.917978 | 18.401967 |  | H | 14.542446 | 14.541203 | 17.996035 |
| C | 6.535688 | 4.385291 | 14.999807 |  | C | 5.088022 | 3.138014 | 15.836172 |
| H | 6.04656 | 5.007937 | 14.249635 |  | H | 4.977648 | 3.669963 | 14.890071 |
| C | 11.173166 | 11.04562 | 22.452836 |  | C | 12.314726 | 11.775636 | 21.69745 |
| H | 10.881961 | 10.541221 | 23.367044 |  | H | 12.288979 | 11.005187 | 22.459896 |
| C | 11.994191 | 10.395546 | 21.548785 |  | C | 13.183096 | 11.645266 | 20.628104 |
| H | 12.340828 | 9.389297 | 21.757898 |  | H | 13.828107 | 10.776163 | 20.555831 |
| C | 6.729334 | 18.90801 | 18.884376 |  | C | 5.324374 | 18.420831 | 20.647754 |
| H | 6.016307 | 19.639672 | 18.520042 |  | H | 4.392057 | 18.96036 | 20.519994 |
| C | 3.206599 | 3.004449 | 13.125297 |  | C | 1.7212 | 2.862392 | 13.804429 |
| H | 4.142571 | 2.47815 | 13.331393 |  | H | 2.335761 | 2.090088 | 14.274503 |
| H | 2.949362 | 2.83887 | 12.079418 |  | H | 1.466275 | 2.526369 | 12.799652 |
| H | 2.434742 | 2.537802 | 13.741575 |  | H | 0.796604 | 2.927474 | 14.383843 |
| C | 5.962926 | 7.722619 | 10.252783 |  | C | 6.470904 | 5.116365 | 10.643091 |
| H | 5.169732 | 7.253304 | 9.665093 |  | H | 5.610786 | 4.75576 | 10.072968 |
| H | 6.872185 | 7.127996 | 10.127111 |  | H | 7.094028 | 4.252777 | 10.892849 |
| H | 6.156759 | 8.706919 | 9.817741 |  | H | 7.057802 | 5.761259 | 9.982724 |
| C | 0.92436 | 8.042339 | 13.825867 |  | C | 1.611047 | 8.281596 | 12.683796 |
| H | 1.255559 | 7.062606 | 14.173272 |  | H | 1.473467 | 7.393826 | 13.302918 |
| C | 4.043432 | 9.773151 | 11.398341 |  | C | 5.45057 | 7.993925 | 10.739787 |
| H | 4.813656 | 10.230745 | 10.788126 |  | H | 6.41378 | 7.944193 | 10.244445 |
| C | 5.224981 | 9.754206 | 22.660334 |  | C | 7.427918 | 8.987548 | 21.526376 |
| H | 4.810595 | 10.30779 | 23.497089 |  | H | 7.280285 | 9.289572 | 22.557983 |
| C | 9.807206 | 13.013154 | 23.220517 |  | C | 10.544934 | 12.995518 | 23.00513 |
| H | 9.572432 | 14.008933 | 22.841638 |  | H | 9.999894 | 13.935316 | 22.909367 |
| C | 1.866446 | 9.886084 | 12.397818 |  | C | 3.375681 | 9.139073 | 11.106945 |
| H | 0.943423 | 10.429526 | 12.564647 |  | H | 2.72349 | 9.979616 | 10.897935 |
| C | 6.561837 | 7.964924 | 21.804139 |  | C | 8.507429 | 7.601837 | 19.895138 |
| H | 7.217185 | 7.114114 | 21.96354 |  | H | 9.214998 | 6.819136 | 19.641626 |
| C | 7.469656 | 5.292543 | 15.800655 |  | C | 6.30491 | 3.728202 | 16.549395 |
| H | 6.912944 | 6.075813 | 16.318721 |  | H | 6.16254 | 4.788854 | 16.76612 |
| H | 8.19441 | 5.771544 | 15.135696 |  | H | 7.198023 | 3.624034 | 15.92593 |
| H | 8.032352 | 4.724372 | 16.547926 |  | H | 6.500245 | 3.213825 | 17.495494 |
| C | 1.112994 | 4.698778 | 17.48423 |  | C | 0.40336 | 6.560868 | 17.293856 |
| H | 1.19668 | 4.188572 | 18.44867 |  | H | 0.153009 | 6.328761 | 18.333756 |
| H | 0.058229 | 4.944071 | 17.32844 |  | H | -0.374822 | 7.224858 | 16.905499 |
| H | 1.670385 | 5.63469 | 17.551462 |  | H | 1.348628 | 7.107064 | 17.289539 |
| C | 11.599794 | 12.317317 | 17.582661 |  | C | 11.87744 | 14.236661 | 17.29958 |
| H | 11.868771 | 11.261469 | 17.480929 |  | H | 12.462973 | 13.389182 | 16.929837 |
| H | 11.868644 | 12.820695 | 16.64902 |  | H | 11.88926 | 15.010743 | 16.526261 |
| H | 10.516062 | 12.375644 | 17.698955 |  | H | 10.846348 | 13.901834 | 17.426861 |
| C | 6.411664 | 16.529834 | 22.591843 |  | C | 6.108445 | 15.122851 | 23.47975 |
| H | 5.508716 | 17.12707 | 22.432389 |  | H | 5.054919 | 15.418403 | 23.502957 |
| H | 6.446302 | 16.256274 | 23.650574 |  | H | 6.324488 | 14.590131 | 24.410852 |
| H | 6.313452 | 15.612239 | 22.009065 |  | H | 6.242392 | 14.423726 | 22.652111 |
| C | 0.767589 | 2.553602 | 16.222513 |  | C | -0.863042 | 4.573578 | 16.413848 |
| H | 1.119237 | 1.894656 | 15.424403 |  | H | -0.826368 | 3.662852 | 15.810506 |
| H | -0.269568 | 2.823296 | 16.004289 |  | H | -1.627849 | 5.228869 | 15.987188 |
| H | 0.763243 | 1.973924 | 17.149814 |  | H | -1.197866 | 4.292126 | 17.416324 |
| C | 6.037871 | 8.651139 | 22.891538 |  | C | 8.341002 | 7.987363 | 21.218484 |
| H | 6.266248 | 8.336813 | 23.904553 |  | H | 8.912729 | 7.506579 | 22.005269 |
| C | 10.512025 | 13.19452 | 24.564997 |  | C | 11.323073 | 13.054475 | 24.319731 |
| H | 10.75185 | 12.232176 | 25.026488 |  | H | 11.87483 | 12.127977 | 24.503996 |
| H | 9.86837 | 13.740542 | 25.260773 |  | H | 10.638961 | 13.202248 | 25.160546 |
| H | 11.445691 | 13.754286 | 24.460949 |  | H | 12.043985 | 13.876469 | 24.325187 |
| C | 2.858315 | 10.454505 | 11.61882 |  | C | 4.608189 | 9.062166 | 10.481935 |
| H | 2.708078 | 11.435604 | 11.181447 |  | H | 4.914275 | 9.838871 | 9.789322 |
| C | 7.817585 | 18.552955 | 23.090418 |  | C | 6.875801 | 17.262008 | 24.552915 |
| H | 8.704902 | 19.136378 | 22.831392 |  | H | 7.513761 | 18.145956 | 24.473699 |
| H | 7.903362 | 18.258602 | 24.140217 |  | H | 7.151374 | 16.728785 | 25.467257 |
| H | 6.952159 | 19.21664 | 23.009436 |  | H | 5.846183 | 17.609108 | 24.677449 |
| C | 7.319589 | 3.307792 | 14.250368 |  | C | 5.308054 | 1.661846 | 15.506688 |
| H | 7.871167 | 2.658528 | 14.936495 |  | H | 5.463943 | 1.064774 | 16.410037 |
| H | 8.048325 | 3.767798 | 13.576678 |  | H | 6.194411 | 1.541009 | 14.877099 |
| H | 6.66101 | 2.673241 | 13.651021 |  | H | 4.455712 | 1.235649 | 14.970975 |
| C | 10.353305 | 17.68312 | 16.756233 |  | C | 8.92973 | 19.072227 | 18.255407 |
| H | 9.782921 | 18.396445 | 16.154141 |  | H | 8.115925 | 19.707826 | 17.893967 |
| H | 11.052275 | 17.173141 | 16.086984 |  | H | 9.686066 | 19.025937 | 17.466357 |
| H | 10.938987 | 18.255227 | 17.48089 |  | H | 9.377764 | 19.567402 | 19.12104 |
| C | 8.48352 | 12.266414 | 23.38657 |  | C | 9.510407 | 11.870906 | 23.026285 |
| H | 7.965029 | 12.150868 | 22.433115 |  | H | 8.938329 | 11.837954 | 22.09731 |
| H | 7.825135 | 12.812361 | 24.068804 |  | H | 8.810173 | 12.015115 | 23.854869 |
| H | 8.636165 | 11.267143 | 23.804649 |  | H | 9.984719 | 10.893982 | 23.160033 |
| C | 8.660485 | 15.844184 | 16.417921 |  | C | 7.851993 | 16.969665 | 17.374165 |
| H | 8.020998 | 15.105968 | 16.905846 |  | H | 7.515589 | 15.959468 | 17.616302 |
| H | 9.351174 | 15.312994 | 15.75638 |  | H | 8.604913 | 16.899127 | 16.583451 |
| H | 8.027827 | 16.480873 | 15.791783 |  | H | 6.998667 | 17.52251 | 16.969341 |
| C | -0.350648 | 7.825755 | 13.010403 |  | C | 0.465213 | 8.315469 | 11.672664 |
| H | -0.763473 | 8.772293 | 12.649608 |  | H | 0.513887 | 9.204182 | 11.03658 |
| H | -1.11792 | 7.345906 | 13.624908 |  | H | -0.497998 | 8.336185 | 12.190767 |
| H | -0.171088 | 7.189197 | 12.139526 |  | H | 0.475437 | 7.439548 | 11.018618 |
| C | 0.650619 | 8.895197 | 15.064814 |  | C | 1.559563 | 9.495386 | 13.611736 |
| H | 1.554179 | 9.040516 | 15.659655 |  | H | 2.357053 | 9.465459 | 14.356458 |
| H | -0.100112 | 8.412087 | 15.697196 |  | H | 0.601562 | 9.528423 | 14.139642 |
| H | 0.26754 | 9.883626 | 14.794449 |  | H | 1.661167 | 10.430526 | 13.052671 |

| **2b** (UBHandHLYP/def2-SVP)  Open-shell singlet  *E* = -3624.125848 Hartree | | | |  | **2b** (UBHandHLYP/def2-SVP)  Closed-shell singlet  *E* = -3624.123749 Hartree | | | |
| --- | --- | --- | --- | --- | --- | --- | --- | --- |
| Atom | X | Y | Z |  | Atom | X | Y | Z |
| N | 19.559509 | 13.025082 | 10.404409 | BzIPr自由基 | N | 19.363825 | 13.542388 | 10.081734 |
| N | 18.169036 | 14.198944 | 11.681194 | OS | N | 17.777886 | 14.355218 | 11.394618 |
| N | 8.738342 | 14.490056 | 1.718259 |  | N | 9.204901 | 14.6136 | 1.982318 |
| N | 7.220881 | 13.588957 | 3.071777 |  | N | 7.506008 | 14.132631 | 3.316889 |
| C | 7.512392 | 14.570027 | 1.079947 |  | C | 8.048553 | 15.032603 | 1.343328 |
| C | 18.2394 | 13.496271 | 10.469206 |  | C | 17.979449 | 13.656343 | 10.205389 |
| C | 20.264784 | 13.427113 | 11.525575 |  | C | 19.992084 | 14.15503 | 11.155024 |
| C | 20.373226 | 10.949467 | 9.419466 |  | C | 20.697233 | 11.772534 | 9.065037 |
| C | 20.233833 | 12.34423 | 9.347915 |  | C | 20.136974 | 13.05702 | 8.983827 |
| C | 6.559472 | 14.018748 | 1.933885 |  | C | 6.981922 | 14.740385 | 2.186526 |
| C | 19.390687 | 14.154668 | 12.329983 |  | C | 18.995089 | 14.654733 | 11.986092 |
| C | 14.056437 | 12.731332 | 6.581867 |  | C | 13.984339 | 11.695963 | 6.640334 |
| C | 8.590301 | 13.874119 | 2.969404 |  | C | 8.894515 | 14.045426 | 3.217312 |
| C | 6.47773 | 13.098226 | 4.185895 |  | C | 6.652905 | 13.820687 | 4.418438 |
| C | 17.216607 | 13.304809 | 9.529513 |  | C | 17.011792 | 13.174206 | 9.334469 |
| C | 15.09371 | 12.916825 | 7.56903 |  | C | 14.968965 | 12.163907 | 7.484078 |
| C | 12.683753 | 12.801318 | 6.875519 |  | C | 12.546893 | 11.824359 | 6.992314 |
| C | 17.051625 | 14.820582 | 12.313303 |  | C | 16.559584 | 14.645376 | 12.081092 |
| C | 9.929847 | 14.855541 | 1.024072 |  | C | 10.458069 | 14.676424 | 1.300176 |
| C | 14.941179 | 13.750092 | 8.709866 |  | C | 14.672952 | 13.052744 | 8.588829 |
| H | 14.013673 | 14.289795 | 8.846579 |  | H | 13.652841 | 13.38885 | 8.715282 |
| C | 14.507868 | 12.390894 | 5.203432 |  | C | 14.30709 | 10.963551 | 5.391936 |
| C | 16.365826 | 12.295216 | 7.455904 |  | C | 16.370278 | 11.806848 | 7.392727 |
| H | 16.554909 | 11.633687 | 6.619266 |  | H | 16.678994 | 11.096215 | 6.637847 |
| C | 17.363273 | 12.463091 | 8.376049 |  | C | 17.30916 | 12.270476 | 8.246425 |
| H | 18.279945 | 11.923149 | 8.210553 |  | H | 18.314199 | 11.90624 | 8.112241 |
| C | 15.932905 | 13.941995 | 9.628957 |  | C | 15.612315 | 13.521653 | 9.43717 |
| H | 15.725114 | 14.620092 | 10.439285 |  | H | 15.283894 | 14.207555 | 10.200136 |
| C | 16.208178 | 14.046641 | 13.123428 |  | C | 15.980296 | 13.657615 | 12.890161 |
| C | 9.591202 | 13.603635 | 3.912564 |  | C | 9.776581 | 13.506883 | 4.144573 |
| C | 20.802087 | 13.092875 | 8.306742 |  | C | 20.372874 | 13.902866 | 7.890611 |
| C | 22.000503 | 13.75966 | 13.115482 |  | C | 21.646365 | 14.987751 | 12.640872 |
| H | 23.026978 | 13.614311 | 13.432605 |  | H | 22.687536 | 15.131527 | 12.906398 |
| C | 10.633984 | 13.874063 | 0.311503 |  | C | 10.897977 | 13.558083 | 0.577768 |
| C | 21.081595 | 10.311715 | 8.404912 |  | C | 21.489463 | 11.3404 | 8.005382 |
| H | 21.197428 | 9.233954 | 8.431136 |  | H | 21.929283 | 10.349938 | 8.037304 |
| C | 12.203433 | 12.545213 | 8.262367 |  | C | 12.121333 | 11.261958 | 8.296645 |
| C | 10.322739 | 16.203332 | 1.015052 |  | C | 11.17633 | 15.882228 | 1.315866 |
| C | 10.930151 | 14.109446 | 3.796453 |  | C | 11.21273 | 13.631212 | 4.03303 |
| H | 11.198752 | 14.746407 | 2.970475 |  | H | 11.642122 | 14.190917 | 3.21906 |
| C | 11.668191 | 13.059026 | 5.881814 |  | C | 11.643289 | 12.365341 | 6.102774 |
| C | 21.125841 | 14.471373 | 13.923826 |  | C | 20.650426 | 15.465836 | 13.482583 |
| H | 21.474486 | 14.874007 | 14.868114 |  | H | 20.922671 | 15.973952 | 14.400691 |
| C | 6.107276 | 11.744356 | 4.210038 |  | C | 5.949334 | 12.606199 | 4.407419 |
| C | 15.497379 | 13.137677 | 4.556938 |  | C | 15.323566 | 11.376679 | 4.519943 |
| H | 15.924726 | 13.995819 | 5.063805 |  | H | 15.88767 | 12.272306 | 4.753027 |
| C | 16.402782 | 12.55279 | 13.302058 |  | C | 16.566275 | 12.264511 | 13.021756 |
| H | 17.255809 | 12.255869 | 12.690526 |  | H | 17.444726 | 12.211276 | 12.377416 |
| C | 11.900112 | 13.844833 | 4.720736 |  | C | 12.070127 | 13.100706 | 4.930275 |
| H | 12.875239 | 14.289276 | 4.57319 |  | H | 13.129343 | 13.267084 | 4.788851 |
| C | 21.635898 | 11.030789 | 7.359593 |  | C | 21.718718 | 12.15166 | 6.907051 |
| H | 22.18062 | 10.514977 | 6.576135 |  | H | 22.334135 | 11.793894 | 6.088655 |
| C | 6.076243 | 13.993605 | 5.187984 |  | C | 6.486741 | 14.766626 | 5.440065 |
| C | 19.759571 | 10.129745 | 10.53993 |  | C | 20.445007 | 10.846803 | 10.24204 |
| H | 19.249849 | 10.821967 | 11.211638 |  | H | 19.807513 | 11.379658 | 10.949257 |
| C | 5.813517 | 15.016233 | -0.52217 |  | C | 6.540384 | 15.945896 | -0.246907 |
| H | 5.509614 | 15.399485 | -1.489696 |  | H | 6.352559 | 16.414743 | -1.206135 |
| C | 10.202712 | 12.419797 | 0.2914 |  | C | 10.122174 | 12.254886 | 0.540002 |
| H | 9.322495 | 12.326516 | 0.928604 |  | H | 9.235038 | 12.378346 | 1.162306 |
| C | 16.873933 | 16.205212 | 12.16682 |  | C | 16.017692 | 15.936139 | 11.978537 |
| C | 11.290146 | 13.401491 | 8.884951 |  | C | 11.185854 | 11.902043 | 9.120941 |
| H | 10.945546 | 14.281064 | 8.352474 |  | H | 10.765343 | 12.849819 | 8.805407 |
| C | 9.36426 | 12.807316 | 5.084494 |  | C | 9.346033 | 12.760061 | 5.304985 |
| H | 8.400339 | 12.359549 | 5.25881 |  | H | 8.297411 | 12.567741 | 5.459636 |
| C | 4.861281 | 14.489056 | 0.338068 |  | C | 5.477307 | 15.673733 | 0.60446 |
| H | 3.819525 | 14.468896 | 0.038586 |  | H | 4.469372 | 15.93727 | 0.304719 |
| C | 21.497278 | 12.407296 | 7.314335 |  | C | 21.165879 | 13.41925 | 6.853453 |
| H | 21.938396 | 12.960479 | 6.492766 |  | H | 21.35606 | 14.047879 | 5.990842 |
| C | 10.342442 | 12.565132 | 6.008875 |  | C | 10.203933 | 12.237855 | 6.208843 |
| H | 10.09256 | 11.943304 | 6.859905 |  | H | 9.790778 | 11.654418 | 7.020717 |
| C | 5.335622 | 11.298279 | 5.27922 |  | C | 5.079031 | 12.348908 | 5.462728 |
| H | 5.040892 | 10.25591 | 5.326364 |  | H | 4.527562 | 11.415685 | 5.484073 |
| C | 15.156967 | 14.68965 | 13.771415 |  | C | 14.819867 | 13.986104 | 13.585911 |
| H | 14.483198 | 14.113668 | 14.395602 |  | H | 14.343429 | 13.238586 | 14.209879 |
| C | 4.942992 | 12.162624 | 6.287072 |  | C | 4.911872 | 13.262174 | 6.489924 |
| H | 4.345608 | 11.794558 | 7.114254 |  | H | 4.232799 | 13.040733 | 7.306219 |
| C | 13.977499 | 11.28818 | 4.528392 |  | C | 13.564441 | 9.835754 | 5.01696 |
| H | 13.212981 | 10.692803 | 5.01405 |  | H | 12.753523 | 9.505001 | 5.655535 |
| C | 11.766791 | 14.268051 | -0.395493 |  | C | 12.098679 | 13.665878 | -0.118554 |
| H | 12.335838 | 13.527374 | -0.945847 |  | H | 12.469004 | 12.813022 | -0.675872 |
| C | 20.674689 | 14.602774 | 8.230422 |  | C | 19.794887 | 15.302968 | 7.804353 |
| H | 20.076153 | 14.927462 | 9.082425 |  | H | 19.213448 | 15.479179 | 8.710031 |
| C | 12.628022 | 11.417976 | 8.970822 |  | C | 12.682823 | 10.07014 | 8.774516 |
| H | 13.332337 | 10.738987 | 8.504187 |  | H | 13.428888 | 9.563533 | 8.173258 |
| C | 10.828847 | 13.148889 | 10.168399 |  | C | 10.809437 | 11.364908 | 10.342807 |
| H | 10.127473 | 13.834619 | 10.632408 |  | H | 10.086852 | 11.8909 | 10.958707 |
| C | 5.310331 | 13.496164 | 6.238752 |  | C | 5.609312 | 14.45748 | 6.475922 |
| H | 4.994183 | 14.165289 | 7.031043 |  | H | 5.467955 | 15.166107 | 7.284229 |
| C | 15.811433 | 16.801142 | 12.839997 |  | C | 14.861124 | 16.215101 | 12.700652 |
| H | 15.646879 | 17.868125 | 12.739451 |  | H | 14.416057 | 17.201693 | 12.635791 |
| C | 11.457998 | 16.546681 | 0.286589 |  | C | 12.366601 | 15.939706 | 0.596881 |
| H | 11.787899 | 17.57942 | 0.267116 |  | H | 12.946259 | 16.855923 | 0.597059 |
| C | 14.957917 | 16.05225 | 13.632404 |  | C | 14.264265 | 15.25015 | 13.494229 |
| H | 14.132664 | 16.534247 | 14.145467 |  | H | 13.359412 | 15.485083 | 14.044254 |
| C | 6.541641 | 10.764168 | 3.134847 |  | C | 6.129461 | 11.571217 | 3.311267 |
| H | 7.13063 | 11.319507 | 2.403388 |  | H | 6.867272 | 11.961259 | 2.60839 |
| C | 9.571192 | 17.273597 | 1.7863 |  | C | 10.71618 | 17.093334 | 2.10754 |
| H | 8.727199 | 16.790089 | 2.280501 |  | H | 9.771954 | 16.832944 | 2.58834 |
| C | 20.825713 | 9.401979 | 11.358135 |  | C | 21.739959 | 10.492556 | 10.972484 |
| H | 21.366809 | 8.669748 | 10.751307 |  | H | 22.422172 | 9.928773 | 10.329254 |
| H | 20.364197 | 8.863467 | 12.190906 |  | H | 21.524851 | 9.872721 | 11.847615 |
| H | 21.55574 | 10.10004 | 11.774225 |  | H | 22.264008 | 11.387059 | 11.316474 |
| C | 12.177178 | 15.589619 | -0.409141 |  | C | 12.826976 | 14.8426 | -0.111366 |
| H | 13.062437 | 15.8764 | -0.966504 |  | H | 13.760166 | 14.905235 | -0.660656 |
| C | 11.26199 | 12.024358 | 10.858131 |  | C | 11.362005 | 10.171026 | 10.786296 |
| H | 10.898624 | 11.822429 | 11.86015 |  | H | 11.068323 | 9.749917 | 11.74194 |
| C | 15.930953 | 12.804126 | 3.282339 |  | C | 15.603703 | 10.682348 | 3.35261 |
| H | 16.693619 | 13.406011 | 2.799093 |  | H | 16.394252 | 11.03428 | 2.697394 |
| C | 17.778865 | 17.05043 | 11.289052 |  | C | 16.628105 | 17.007754 | 11.092771 |
| H | 18.525518 | 16.38599 | 10.851541 |  | H | 17.516845 | 16.581652 | 10.624598 |
| C | 12.161929 | 11.157913 | 10.250667 |  | C | 12.305419 | 9.529529 | 9.993666 |
| H | 12.500983 | 10.271124 | 10.775912 |  | H | 12.752233 | 8.598475 | 10.327257 |
| C | 9.792527 | 11.980551 | -1.11408 |  | C | 9.639753 | 11.935182 | -0.874916 |
| H | 8.994231 | 12.612391 | -1.511408 |  | H | 9.026995 | 12.74456 | -1.280166 |
| H | 9.430782 | 10.948194 | -1.100278 |  | H | 9.036541 | 11.022623 | -0.874016 |
| H | 10.635399 | 12.025938 | -1.810334 |  | H | 10.479172 | 11.774746 | -1.557986 |
| C | 16.740457 | 12.202697 | 14.751147 |  | C | 17.037791 | 11.991706 | 14.45018 |
| H | 17.630718 | 12.736359 | 15.093064 |  | H | 17.768259 | 12.735487 | 14.778726 |
| H | 16.931956 | 11.130079 | 14.848937 |  | H | 17.507743 | 11.005917 | 14.513611 |
| H | 15.917186 | 12.453793 | 15.42677 |  | H | 16.203208 | 12.00508 | 15.157528 |
| C | 6.450271 | 15.463694 | 5.159752 |  | C | 7.227693 | 16.090475 | 5.452389 |
| H | 7.070035 | 15.630719 | 4.278047 |  | H | 7.85821 | 16.126882 | 4.563177 |
| C | 14.415358 | 10.946698 | 3.257738 |  | C | 13.844681 | 9.138247 | 3.852473 |
| H | 13.993524 | 10.080367 | 2.759011 |  | H | 13.256826 | 8.261843 | 3.599209 |
| C | 15.193395 | 11.762413 | 12.803865 |  | C | 15.589112 | 11.192133 | 12.541018 |
| H | 14.301018 | 11.974156 | 13.400542 |  | H | 14.696743 | 11.149004 | 13.172426 |
| H | 15.38893 | 10.688214 | 12.874353 |  | H | 16.063511 | 10.207011 | 12.5749 |
| H | 14.962222 | 11.99869 | 11.763011 |  | H | 15.263722 | 11.377285 | 11.515219 |
| C | 18.70824 | 9.152868 | 10.012561 |  | C | 19.693888 | 9.584418 | 9.816701 |
| H | 17.925925 | 9.670952 | 9.453312 |  | H | 18.746442 | 9.824636 | 9.329365 |
| H | 18.234219 | 8.621884 | 10.843251 |  | H | 19.474629 | 8.962778 | 10.689607 |
| H | 19.153338 | 8.401779 | 9.35298 |  | H | 20.285829 | 8.980554 | 9.122557 |
| C | 15.392195 | 11.705562 | 2.625914 |  | C | 14.870102 | 9.553855 | 3.012512 |
| H | 15.7336 | 11.440314 | 1.630952 |  | H | 15.088131 | 9.010044 | 2.099503 |
| C | 11.278232 | 11.502999 | 0.872439 |  | C | 10.929893 | 11.099671 | 1.130897 |
| H | 12.183555 | 11.505756 | 0.258016 |  | H | 11.821181 | 10.885237 | 0.533841 |
| H | 10.913189 | 10.472513 | 0.91639 |  | H | 10.323928 | 10.189233 | 1.155951 |
| H | 11.559678 | 11.808808 | 1.882243 |  | H | 11.255988 | 11.318815 | 2.149778 |
| C | 9.002505 | 18.347458 | 0.859183 |  | C | 10.451158 | 18.297149 | 1.20403 |
| H | 9.797391 | 18.894739 | 0.343589 |  | H | 11.365726 | 18.635246 | 0.707822 |
| H | 8.420004 | 19.074618 | 1.43222 |  | H | 10.06621 | 19.13569 | 1.791477 |
| H | 8.345512 | 17.914697 | 0.10143 |  | H | 9.715391 | 18.06417 | 0.431037 |
| C | 18.528612 | 18.1066 | 12.100476 |  | C | 17.077665 | 18.225105 | 11.899952 |
| H | 17.84109 | 18.827798 | 12.552783 |  | H | 16.230056 | 18.723514 | 12.379753 |
| H | 19.214991 | 18.664309 | 11.456767 |  | H | 17.562857 | 18.956207 | 11.246854 |
| H | 19.115292 | 17.651992 | 12.902044 |  | H | 17.790639 | 17.947877 | 12.679697 |
| C | 22.03742 | 15.28515 | 8.347471 |  | C | 20.897542 | 16.360893 | 7.7644 |
| H | 22.546153 | 15.00174 | 9.272143 |  | H | 21.556774 | 16.282457 | 8.632645 |
| H | 21.92009 | 16.372779 | 8.346593 |  | H | 20.461926 | 17.364326 | 7.761003 |
| H | 22.690783 | 15.022847 | 7.509907 |  | H | 21.513545 | 16.266075 | 6.865182 |
| C | 10.44016 | 17.896862 | 2.879083 |  | C | 11.706681 | 17.448896 | 3.217264 |
| H | 10.820096 | 17.140106 | 3.568988 |  | H | 11.875824 | 16.605476 | 3.890326 |
| H | 9.859093 | 18.620109 | 3.458879 |  | H | 11.325271 | 18.284077 | 3.812036 |
| H | 11.297995 | 18.427808 | 2.455633 |  | H | 12.675004 | 17.751327 | 2.807505 |
| C | 5.345517 | 10.163939 | 2.396801 |  | C | 4.834598 | 11.339941 | 2.532844 |
| H | 4.716284 | 9.570516 | 3.067031 |  | H | 4.051086 | 10.924095 | 3.173491 |
| H | 5.686866 | 9.503248 | 1.594651 |  | H | 5.003182 | 10.631919 | 1.716315 |
| H | 4.721631 | 10.940418 | 1.948575 |  | H | 4.457066 | 12.268235 | 2.098312 |
| C | 19.936587 | 15.044425 | 6.967068 |  | C | 18.839108 | 15.446599 | 6.620415 |
| H | 20.499544 | 14.790906 | 6.063692 |  | H | 19.362618 | 15.326602 | 5.66701 |
| H | 19.792802 | 16.128863 | 6.971554 |  | H | 18.380489 | 16.439688 | 6.621822 |
| H | 18.95437 | 14.571674 | 6.896891 |  | H | 18.040295 | 14.703138 | 6.664126 |
| C | 17.007699 | 17.688415 | 10.133337 |  | C | 15.676383 | 17.416879 | 9.967739 |
| H | 16.497393 | 16.934977 | 9.529138 |  | H | 15.375418 | 16.558121 | 9.363667 |
| H | 17.691171 | 18.239012 | 9.4802 |  | H | 16.161297 | 18.141097 | 9.306596 |
| H | 16.255811 | 18.396552 | 10.494537 |  | H | 14.769375 | 17.885555 | 10.360859 |
| C | 7.442297 | 9.667225 | 3.703666 |  | C | 6.680668 | 10.255556 | 3.862238 |
| H | 8.320926 | 10.086989 | 4.198479 |  | H | 7.621908 | 10.406331 | 4.395428 |
| H | 7.789248 | 9.008587 | 2.902086 |  | H | 6.865561 | 9.551664 | 3.045662 |
| H | 6.909613 | 9.048059 | 4.431572 |  | H | 5.975044 | 9.781952 | 4.551238 |
| C | 5.213659 | 16.351078 | 5.018176 |  | C | 6.261815 | 17.272294 | 5.369862 |
| H | 5.505805 | 17.402694 | 4.943587 |  | H | 6.816111 | 18.214062 | 5.320453 |
| H | 4.548327 | 16.253617 | 5.881438 |  | H | 5.609013 | 17.3195 | 6.246552 |
| H | 4.641486 | 16.096004 | 4.122741 |  | H | 5.627559 | 17.207242 | 4.482208 |
| C | 7.284378 | 15.858858 | 6.3778 |  | C | 8.151654 | 16.207484 | 6.664238 |
| H | 6.71139 | 15.762982 | 7.305087 |  | H | 7.584826 | 16.218298 | 7.600156 |
| H | 7.604746 | 16.901552 | 6.295531 |  | H | 8.724955 | 17.137804 | 6.615427 |
| H | 8.176492 | 15.234689 | 6.46519 |  | H | 8.857542 | 15.375112 | 6.705683 |
| C | 21.581207 | 13.222858 | 11.895942 |  | C | 21.331243 | 14.320099 | 11.456928 |
| C | 19.799089 | 14.682309 | 13.541114 |  | C | 19.300857 | 15.307472 | 13.166066 |
| C | 7.162266 | 15.066179 | -0.162294 |  | C | 7.850912 | 15.62846 | 0.111311 |
| C | 5.221638 | 13.977513 | 1.586916 |  | C | 5.682089 | 15.062025 | 1.841642 |
| H | 22.259802 | 12.667361 | 11.261005 |  | H | 22.103555 | 13.944437 | 10.79779 |
| H | 19.11299 | 15.238792 | 14.16699 |  | H | 18.520126 | 15.680335 | 13.816836 |
| H | 7.908748 | 15.477681 | -0.829875 |  | H | 8.683876 | 15.838572 | -0.547469 |
| H | 4.482764 | 13.562588 | 2.260807 |  | H | 4.856868 | 14.846358 | 2.508397 |

| **2b** (UBHandHLYP/def2-SVP)  Open-shell triplet  *E* = -3624.120297 Hartree | | | |  | **2c** (UBHandHLYP/def2-SVP)  Open-shell singlet  *E* = -4417.047220 Hartree | | | |
| --- | --- | --- | --- | --- | --- | --- | --- | --- |
| Atom | X | Y | Z |  | Atom | X | Y | Z |
| N | 19.497595 | 12.776651 | 10.533064 |  | N | 19.572806 | 13.022976 | 10.397764 |
| N | 18.29122 | 14.293414 | 11.63446 |  | N | 18.173987 | 14.147981 | 11.732218 |
| N | 8.623872 | 14.570823 | 1.74056 |  | N | 8.7589 | 14.403078 | 1.644354 |
| N | 7.256289 | 13.294309 | 2.956569 |  | N | 7.222796 | 13.590498 | 3.052369 |
| C | 7.404206 | 14.525811 | 1.088538 |  | C | 7.536012 | 14.462649 | 1.009995 |
| C | 18.26207 | 13.448515 | 10.510704 |  | C | 18.243989 | 13.478364 | 10.495767 |
| C | 20.246003 | 13.197808 | 11.617548 |  | C | 20.279319 | 13.404744 | 11.519188 |
| C | 20.015384 | 10.509407 | 9.806695 |  | C | 20.437961 | 11.056091 | 9.242571 |
| C | 20.071267 | 11.892641 | 9.572928 |  | C | 20.224728 | 12.442197 | 9.263249 |
| C | 6.543472 | 13.742892 | 1.857966 |  | C | 6.573469 | 13.967694 | 1.895147 |
| C | 19.485722 | 14.136705 | 12.31408 |  | C | 19.400458 | 14.093746 | 12.360815 |
| C | 14.06472 | 12.937126 | 6.56273 |  | C | 14.040184 | 12.794653 | 6.612593 |
| C | 8.567353 | 13.800547 | 2.914837 |  | C | 8.601275 | 13.852379 | 2.930598 |
| C | 6.585707 | 12.626723 | 4.023504 |  | C | 6.496496 | 13.216077 | 4.228083 |
| C | 17.225096 | 13.312123 | 9.56223 |  | C | 17.211449 | 13.302516 | 9.560191 |
| C | 15.115147 | 13.049964 | 7.601926 |  | C | 15.083324 | 12.956308 | 7.612234 |
| C | 12.735323 | 12.991176 | 6.838885 |  | C | 12.680864 | 12.909893 | 6.892577 |
| C | 17.259234 | 15.108859 | 12.184464 |  | C | 17.008184 | 14.641413 | 12.400959 |
| C | 9.751786 | 15.176097 | 1.112104 |  | C | 9.977503 | 14.698741 | 0.953588 |
| C | 15.083779 | 14.035439 | 8.600613 |  | C | 14.976394 | 13.853709 | 8.702561 |
| H | 14.251854 | 14.72909 | 8.632404 |  | H | 14.085789 | 14.460131 | 8.801596 |
| C | 14.577883 | 12.765277 | 5.1734 |  | C | 14.504561 | 12.439144 | 5.24173 |
| C | 16.237213 | 12.205416 | 7.605803 |  | C | 16.30618 | 12.247006 | 7.536628 |
| H | 16.318106 | 11.429711 | 6.851867 |  | H | 16.452779 | 11.533967 | 6.734201 |
| C | 17.24022 | 12.312009 | 8.541159 |  | C | 17.310544 | 12.394899 | 8.456267 |
| H | 18.05212 | 11.607236 | 8.480338 |  | H | 18.188312 | 11.78439 | 8.331656 |
| C | 16.083175 | 14.171667 | 9.535998 |  | C | 15.975553 | 14.026794 | 9.621189 |
| H | 15.984663 | 14.969766 | 10.252176 |  | H | 15.815154 | 14.761804 | 10.391235 |
| C | 16.304591 | 14.526078 | 13.030737 |  | C | 16.18682 | 13.739937 | 13.089356 |
| C | 9.599702 | 13.585797 | 3.853357 |  | C | 9.60216 | 13.612354 | 3.886056 |
| C | 20.73853 | 12.42833 | 8.461428 |  | C | 20.666989 | 13.278366 | 8.230567 |
| C | 21.999263 | 13.444871 | 13.206029 |  | C | 22.035232 | 13.718419 | 13.110047 |
| H | 22.989442 | 13.184404 | 13.562553 |  | C | 10.67626 | 13.656721 | 0.331648 |
| C | 10.600141 | 14.387726 | 0.321091 |  | C | 21.09054 | 10.515517 | 8.139577 |
| C | 20.630589 | 9.665949 | 8.885873 |  | H | 21.267071 | 9.4468 | 8.094779 |
| H | 20.596678 | 8.593063 | 9.039039 |  | C | 12.177888 | 12.755992 | 8.287021 |
| C | 12.214515 | 12.876665 | 8.231474 |  | C | 10.410168 | 16.031046 | 0.886583 |
| C | 9.940692 | 16.561285 | 1.245998 |  | C | 10.931861 | 14.136843 | 3.770044 |
| C | 10.813207 | 14.339525 | 3.851154 |  | H | 11.196883 | 14.765762 | 2.937603 |
| H | 10.971677 | 15.112912 | 3.118278 |  | C | 11.66698 | 13.132309 | 5.87457 |
| C | 11.694037 | 13.173989 | 5.800694 |  | C | 21.160354 | 14.375057 | 13.95346 |
| C | 21.234998 | 14.363147 | 13.910025 |  | C | 6.085853 | 11.882992 | 4.373821 |
| H | 21.631339 | 14.811089 | 14.814275 |  | C | 15.513299 | 13.169859 | 4.607797 |
| C | 6.37948 | 11.240641 | 3.933312 |  | H | 15.944798 | 14.024833 | 5.116554 |
| C | 15.590913 | 13.592199 | 4.680823 |  | C | 16.500427 | 12.258665 | 13.17651 |
| H | 15.98292 | 14.381484 | 5.312756 |  | H | 17.366998 | 12.063083 | 12.543369 |
| C | 16.300057 | 13.042277 | 13.346678 |  | C | 11.899682 | 13.90046 | 4.707905 |
| H | 17.1258 | 12.585631 | 12.799639 |  | H | 12.870474 | 14.35498 | 4.560642 |
| C | 11.804656 | 14.133941 | 4.782437 |  | C | 21.517237 | 11.320506 | 7.096775 |
| H | 12.693312 | 14.751999 | 4.733028 |  | H | 22.02075 | 10.879426 | 6.243353 |
| C | 21.280748 | 10.174624 | 7.774334 |  | C | 6.180258 | 14.199714 | 5.173736 |
| H | 21.750658 | 9.500869 | 7.065915 |  | C | 19.981923 | 10.145025 | 10.367721 |
| C | 6.092997 | 13.377987 | 5.100935 |  | H | 19.458007 | 10.762957 | 11.099158 |
| C | 19.290692 | 9.918024 | 11.002384 |  | C | 5.828587 | 14.84846 | -0.6174 |
| H | 18.883959 | 10.747704 | 11.582228 |  | C | 10.175213 | 12.225402 | 0.339779 |
| C | 5.662673 | 14.878499 | -0.493239 |  | H | 9.299968 | 12.183027 | 0.989681 |
| H | 5.308252 | 15.313886 | -1.420726 |  | C | 16.748931 | 16.019629 | 12.380238 |
| C | 10.386721 | 12.896612 | 0.140576 |  | C | 11.292876 | 13.683975 | 8.842811 |
| H | 9.520493 | 12.611422 | 0.738581 |  | H | 10.990185 | 14.54284 | 8.253958 |
| C | 17.269844 | 16.48754 | 11.919093 |  | C | 9.376439 | 12.833151 | 5.067132 |
| C | 11.273916 | 13.787828 | 8.718839 |  | H | 8.418874 | 12.372953 | 5.240388 |
| H | 10.943593 | 14.599303 | 8.07976 |  | C | 4.874555 | 14.381418 | 0.265515 |
| C | 9.504268 | 12.608758 | 4.890845 |  | C | 21.308571 | 12.687424 | 7.145382 |
| H | 8.634042 | 11.978495 | 4.96673 |  | H | 21.656478 | 13.308769 | 6.327968 |
| C | 4.799024 | 14.123486 | 0.285828 |  | C | 10.352742 | 12.622481 | 6.00462 |
| H | 3.773628 | 13.978396 | -0.03495 |  | H | 10.106009 | 12.015301 | 6.867349 |
| C | 21.332823 | 11.542171 | 7.567042 |  | C | 5.367301 | 11.546039 | 5.516286 |
| H | 21.846806 | 11.929527 | 6.694466 |  | H | 5.03915 | 10.522547 | 5.6583 |
| C | 10.502782 | 12.429892 | 5.819871 |  | C | 15.063222 | 14.247661 | 13.735622 |
| H | 10.361953 | 11.676321 | 6.58724 |  | H | 14.404976 | 13.572499 | 14.270343 |
| C | 5.682111 | 10.61521 | 4.963046 |  | C | 5.064644 | 12.496955 | 6.476268 |
| H | 5.515026 | 9.544655 | 4.921033 |  | H | 4.506771 | 12.213332 | 7.362137 |
| C | 15.333398 | 15.35393 | 13.587455 |  | C | 13.97314 | 11.336213 | 4.568942 |
| H | 14.577288 | 14.926749 | 14.236588 |  | H | 13.19631 | 10.751404 | 5.047816 |
| C | 5.204033 | 11.336682 | 6.043699 |  | C | 11.853678 | 13.974107 | -0.339884 |
| H | 4.667393 | 10.830328 | 6.83882 |  | H | 12.420173 | 13.187626 | -0.825095 |
| C | 14.107424 | 11.740746 | 4.349319 |  | C | 20.491571 | 14.784436 | 8.271293 |
| H | 13.332236 | 11.080037 | 4.719075 |  | H | 19.930028 | 15.030644 | 9.17362 |
| C | 11.667866 | 15.014292 | -0.316236 |  | C | 12.543677 | 11.653564 | 9.06307 |
| H | 12.34475 | 14.426007 | -0.925443 |  | H | 13.222149 | 10.918114 | 8.646417 |
| C | 20.823388 | 13.922105 | 8.210094 |  | C | 10.805422 | 13.526256 | 10.13173 |
| H | 20.272018 | 14.422672 | 9.006935 |  | H | 10.127561 | 14.265965 | 10.544695 |
| C | 12.605116 | 11.826831 | 9.065459 |  | C | 5.464974 | 13.809984 | 6.302759 |
| H | 13.322311 | 11.101354 | 8.700154 |  | H | 5.210918 | 14.54842 | 7.054829 |
| C | 10.763579 | 13.671108 | 10.003186 |  | C | 15.617605 | 16.477576 | 13.047353 |
| H | 10.043309 | 14.398134 | 10.36356 |  | H | 15.389234 | 17.537412 | 13.045375 |
| C | 5.40817 | 12.704001 | 6.108529 |  | C | 11.58877 | 16.297017 | 0.197386 |
| H | 5.025063 | 13.26015 | 6.956813 |  | H | 11.949931 | 17.317009 | 0.130374 |
| C | 16.281368 | 17.27297 | 12.505106 |  | C | 14.77727 | 15.601112 | 13.71302 |
| H | 16.261739 | 18.339483 | 12.310306 |  | H | 13.897099 | 15.977203 | 14.223146 |
| C | 11.017418 | 17.140919 | 0.58124 |  | C | 6.403105 | 10.815728 | 3.341971 |
| H | 11.189028 | 18.207696 | 0.671897 |  | H | 6.98088 | 11.289873 | 2.546494 |
| C | 15.318544 | 16.713272 | 13.327943 |  | C | 9.642109 | 17.169022 | 1.533214 |
| H | 14.553093 | 17.341183 | 13.771143 |  | H | 8.782242 | 16.734814 | 2.046664 |
| C | 6.911081 | 10.414499 | 2.775466 |  | C | 21.172276 | 9.499193 | 11.078374 |
| H | 7.421894 | 11.096532 | 2.094281 |  | H | 21.709563 | 8.818151 | 10.411585 |
| C | 9.031604 | 17.42556 | 2.10154 |  | H | 20.832902 | 8.917699 | 11.940479 |
| H | 8.280732 | 16.770373 | 2.54573 |  | H | 21.883888 | 10.250177 | 11.426123 |
| C | 20.240392 | 9.144735 | 11.916791 |  | C | 12.308961 | 15.279015 | -0.40466 |
| H | 20.671933 | 8.277926 | 11.407183 |  | H | 13.228165 | 15.506004 | -0.933757 |
| H | 19.704858 | 8.776715 | 12.796718 |  | C | 11.181012 | 12.425957 | 10.89076 |
| H | 21.062067 | 9.77495 | 12.264527 |  | H | 10.795584 | 12.297713 | 11.896599 |
| C | 11.877144 | 16.376271 | -0.189087 |  | C | 15.96164 | 12.823086 | 3.341876 |
| H | 12.714184 | 16.845549 | -0.6948 |  | H | 16.73862 | 13.412374 | 2.866236 |
| C | 11.166758 | 12.625433 | 10.822881 |  | C | 17.650881 | 17.008018 | 11.662906 |
| H | 10.761804 | 12.527478 | 11.824474 |  | H | 18.447806 | 16.437062 | 11.182926 |
| C | 16.095683 | 13.419967 | 3.400547 |  | C | 12.049266 | 11.487604 | 10.348129 |
| H | 16.87366 | 14.082391 | 3.035629 |  | H | 12.341255 | 10.618303 | 10.927854 |
| C | 18.298608 | 17.129657 | 11.005523 |  | C | 9.724901 | 11.804362 | -1.059941 |
| H | 18.95906 | 16.337935 | 10.648808 |  | H | 8.985967 | 12.500017 | -1.465726 |
| C | 12.085506 | 11.700229 | 10.34521 |  | H | 9.279527 | 10.805416 | -1.03726 |
| H | 12.398515 | 10.871938 | 10.972059 |  | H | 10.568385 | 11.776767 | -1.756181 |
| C | 10.064777 | 12.55221 | -1.31352 |  | C | 16.885384 | 11.87173 | 14.605203 |
| H | 9.188387 | 13.100785 | -1.66753 |  | H | 17.709816 | 12.487398 | 14.974221 |
| H | 9.856963 | 11.482878 | -1.414466 |  | H | 17.193757 | 10.823097 | 14.650401 |
| H | 10.90148 | 12.789983 | -1.977331 |  | H | 16.044031 | 12.001097 | 15.292496 |
| C | 16.546278 | 12.787282 | 14.833601 |  | C | 6.565508 | 15.655529 | 4.993134 |
| H | 17.48771 | 13.233429 | 15.163597 |  | H | 7.155513 | 15.733267 | 4.078963 |
| H | 16.594126 | 11.712634 | 15.032749 |  | C | 14.42732 | 10.980547 | 3.307773 |
| H | 15.74298 | 13.20173 | 15.450198 |  | H | 14.005504 | 10.114106 | 2.809443 |
| C | 6.278428 | 14.880879 | 5.195158 |  | C | 15.35805 | 11.394686 | 12.646893 |
| H | 6.863226 | 15.197767 | 4.331044 |  | H | 14.458398 | 11.49519 | 13.260995 |
| C | 14.621047 | 11.557743 | 3.073913 |  | H | 15.646087 | 10.339333 | 12.6568 |
| H | 14.245101 | 10.750094 | 2.454786 |  | H | 15.096144 | 11.66884 | 11.622674 |
| C | 15.014538 | 12.36803 | 12.869644 |  | C | 18.991154 | 9.087015 | 9.882266 |
| H | 14.139049 | 12.746897 | 13.405499 |  | H | 18.119014 | 9.541295 | 9.40627 |
| H | 15.0658 | 11.289002 | 13.043837 |  | H | 18.639326 | 8.483851 | 10.724298 |
| H | 14.849262 | 12.533041 | 11.802924 |  | H | 19.451844 | 8.40587 | 9.160881 |
| C | 18.111952 | 9.045151 | 10.570382 |  | C | 15.420638 | 11.725564 | 2.685625 |
| H | 17.408753 | 9.602734 | 9.947589 |  | H | 15.774636 | 11.44922 | 1.698188 |
| H | 17.569662 | 8.679456 | 11.447292 |  | C | 11.203742 | 11.254156 | 0.91469 |
| H | 18.446535 | 8.172062 | 10.002158 |  | H | 12.098016 | 11.195017 | 0.28742 |
| C | 15.613526 | 12.400335 | 2.590835 |  | H | 10.779461 | 10.24764 | 0.975733 |
| H | 16.013992 | 12.258187 | 1.592743 |  | H | 11.517076 | 11.5549 | 1.916705 |
| C | 11.576376 | 12.08854 | 0.656557 |  | C | 9.107107 | 18.144712 | 0.484029 |
| H | 12.480726 | 12.286536 | 0.073254 |  | H | 9.923628 | 18.660294 | -0.030397 |
| H | 11.365976 | 11.017215 | 0.584246 |  | H | 8.480246 | 18.907355 | 0.955259 |
| H | 11.794838 | 12.32366 | 1.700292 |  | H | 8.513401 | 17.62924 | -0.273324 |
| C | 8.291274 | 18.469754 | 1.266033 |  | C | 18.307517 | 17.977242 | 12.646872 |
| H | 8.985305 | 19.17898 | 0.804739 |  | H | 17.563767 | 18.624027 | 13.121871 |
| H | 7.603749 | 19.042528 | 1.895145 |  | H | 19.022309 | 18.62251 | 12.127898 |
| H | 7.706639 | 18.003926 | 0.469798 |  | H | 18.835708 | 17.444094 | 13.439452 |
| C | 19.163617 | 18.14436 | 11.752531 |  | C | 21.845551 | 15.487925 | 8.372452 |
| H | 18.566873 | 18.982025 | 12.126017 |  | H | 22.420176 | 15.12207 | 9.227241 |
| H | 19.928672 | 18.555032 | 11.087226 |  | H | 21.710115 | 16.567309 | 8.487499 |
| H | 19.671495 | 17.686371 | 12.604281 |  | H | 22.448177 | 15.323042 | 7.474466 |
| C | 22.267588 | 14.418412 | 8.277241 |  | C | 10.47362 | 17.896901 | 2.589281 |
| H | 22.726192 | 14.177635 | 9.239458 |  | H | 10.830204 | 17.21216 | 3.362327 |
| H | 22.303802 | 15.504201 | 8.148539 |  | H | 9.872926 | 18.670996 | 3.075701 |
| H | 22.882407 | 13.971824 | 7.489909 |  | H | 11.345092 | 18.388225 | 2.14682 |
| C | 9.794449 | 18.086283 | 3.250182 |  | C | 5.129302 | 10.251772 | 2.711101 |
| H | 10.297544 | 17.345698 | 3.875826 |  | H | 4.527255 | 9.710818 | 3.447252 |
| H | 9.106452 | 18.65192 | 3.88543 |  | H | 5.379199 | 9.549835 | 1.91029 |
| H | 10.551069 | 18.785253 | 2.88127 |  | H | 4.506785 | 11.04504 | 2.293146 |
| C | 5.783722 | 9.742977 | 1.991842 |  | C | 19.678227 | 15.299633 | 7.085248 |
| H | 5.239903 | 9.021967 | 2.609586 |  | H | 20.197361 | 15.129124 | 6.137487 |
| H | 6.188781 | 9.201214 | 1.132242 |  | H | 19.511024 | 16.376587 | 7.178946 |
| H | 5.065153 | 10.475258 | 1.617421 |  | H | 18.704619 | 14.807203 | 7.030961 |
| C | 20.160737 | 14.311675 | 6.889086 |  | C | 16.910953 | 17.76334 | 10.558987 |
| H | 20.688167 | 13.88074 | 6.032709 |  | H | 16.476753 | 17.080284 | 9.825257 |
| H | 20.168766 | 15.398794 | 6.767121 |  | H | 17.59808 | 18.4316 | 10.031879 |
| H | 19.122928 | 13.972897 | 6.851061 |  | H | 16.102614 | 18.377827 | 10.965745 |
| C | 17.645735 | 17.763707 | 9.776906 |  | C | 7.270469 | 9.699253 | 3.922984 |
| H | 17.055105 | 17.034947 | 9.217259 |  | H | 8.202708 | 10.08934 | 4.33779 |
| H | 18.411028 | 18.164792 | 9.10591 |  | H | 7.525225 | 8.974091 | 3.144605 |
| H | 16.985305 | 18.590649 | 10.054447 |  | H | 6.748925 | 9.158285 | 4.717966 |
| C | 7.939758 | 9.383939 | 3.242503 |  | C | 5.323898 | 16.528191 | 4.805655 |
| H | 8.770893 | 9.857032 | 3.77028 |  | H | 5.609871 | 17.561485 | 4.588283 |
| H | 8.350488 | 8.843154 | 2.384846 |  | H | 4.703085 | 16.538575 | 5.706393 |
| H | 7.491663 | 8.645096 | 3.913729 |  | H | 4.704021 | 16.164553 | 3.98206 |
| C | 4.937418 | 15.611587 | 5.126615 |  | C | 7.443218 | 16.163918 | 6.135243 |
| H | 5.092245 | 16.694526 | 5.138815 |  | H | 6.905926 | 16.159033 | 7.088181 |
| H | 4.299198 | 15.359397 | 5.978829 |  | H | 7.759273 | 17.193079 | 5.94163 |
| H | 4.393309 | 15.35898 | 4.213165 |  | H | 8.338431 | 15.548201 | 6.248528 |
| C | 7.067195 | 15.278047 | 6.442319 |  | C | 21.604775 | 13.240514 | 11.875658 |
| H | 6.520941 | 15.033199 | 7.35823 |  | C | 19.828332 | 14.554589 | 13.591712 |
| H | 7.250548 | 16.356522 | 6.447134 |  | C | 7.172452 | 14.880041 | -0.256605 |
| H | 8.032448 | 14.768141 | 6.478646 |  | C | 5.238228 | 13.949672 | 1.537885 |
| C | 21.513568 | 12.843456 | 12.041678 |  | F | 22.480118 | 12.622561 | 11.098342 |
| C | 19.958506 | 14.726396 | 13.472053 |  | F | 23.294487 | 13.55418 | 13.465284 |
| C | 6.986657 | 15.093667 | -0.101142 |  | F | 21.580927 | 14.822464 | 15.120408 |
| C | 5.228855 | 13.539824 | 1.480691 |  | F | 19.024572 | 15.182572 | 14.435287 |
| H | 22.105635 | 12.124719 | 11.489244 |  | F | 8.051607 | 15.331139 | -1.137303 |
| H | 19.358156 | 15.443812 | 14.017159 |  | F | 5.470742 | 15.254949 | -1.81968 |
| H | 7.663887 | 15.684586 | -0.704744 |  | F | 3.604738 | 14.356186 | -0.089281 |
| H | 4.558353 | 12.948834 | 2.091651 |  | F | 4.288947 | 13.515617 | 2.351826 |

| **2c** (UBHandHLYP/def2-SVP)  Closed-shell singlet  *E* = -4417.042906 Hartree | | | |  | **2c** (UBHandHLYP/def2-SVP)  Open-shell triplet  *E* = -4417.042554 Hartree | | | |
| --- | --- | --- | --- | --- | --- | --- | --- | --- |
| Atom | X | Y | Z |  | Atom | X | Y | Z |
| N | 19.363265 | 13.566302 | 10.119739 |  | N | 19.495094 | 12.797446 | 10.526185 |
| N | 17.755589 | 14.327059 | 11.453907 |  | N | 18.254237 | 14.24076 | 11.708751 |
| N | 9.193721 | 14.57077 | 1.923423 |  | N | 8.676806 | 14.523244 | 1.670905 |
| N | 7.503698 | 14.13925 | 3.302423 |  | N | 7.252906 | 13.357077 | 2.95135 |
| C | 8.033729 | 14.984662 | 1.298958 |  | C | 7.460733 | 14.485562 | 1.022136 |
| C | 17.971866 | 13.665481 | 10.239607 |  | C | 18.237851 | 13.437612 | 10.549551 |
| C | 19.973626 | 14.148811 | 11.213055 |  | C | 20.239717 | 13.199359 | 11.614475 |
| C | 20.6782 | 11.832171 | 9.015033 |  | C | 20.089153 | 10.616147 | 9.610056 |
| C | 20.126142 | 13.120918 | 8.991488 |  | C | 20.065028 | 12.011967 | 9.474371 |
| C | 6.97422 | 14.723996 | 2.168982 |  | C | 6.566254 | 13.775469 | 1.831232 |
| C | 18.964127 | 14.615952 | 12.055801 |  | C | 19.45809 | 14.086988 | 12.362297 |
| C | 14.013139 | 11.755852 | 6.613246 |  | C | 14.037388 | 12.97781 | 6.601133 |
| C | 8.894643 | 14.03422 | 3.181256 |  | C | 8.58512 | 13.814997 | 2.886621 |
| C | 6.691733 | 13.879174 | 4.454062 |  | C | 6.583732 | 12.76232 | 4.067928 |
| C | 17.015942 | 13.204089 | 9.350868 |  | C | 17.192175 | 13.310291 | 9.606031 |
| C | 14.988844 | 12.218077 | 7.468798 |  | C | 15.083267 | 13.075998 | 7.647906 |
| C | 12.573192 | 11.869796 | 6.956873 |  | C | 12.708573 | 13.054113 | 6.867118 |
| C | 16.512719 | 14.559299 | 12.128797 |  | C | 17.168526 | 14.966421 | 12.294188 |
| C | 10.449671 | 14.564397 | 1.23286 |  | C | 9.860527 | 15.012143 | 1.031877 |
| C | 14.683788 | 13.097802 | 8.578427 |  | C | 15.074261 | 14.076283 | 8.630571 |
| H | 13.66428 | 13.438911 | 8.695742 |  | H | 14.262179 | 14.793472 | 8.648516 |
| C | 14.350676 | 11.038212 | 5.35931 |  | C | 14.560762 | 12.788963 | 5.217692 |
| C | 16.391182 | 11.863923 | 7.383149 |  | C | 16.178129 | 12.198497 | 7.668771 |
| H | 16.706057 | 11.16277 | 6.622013 |  | H | 16.237276 | 11.407415 | 6.929146 |
| C | 17.322498 | 12.314684 | 8.249975 |  | C | 17.180856 | 12.291203 | 8.606484 |
| H | 18.326668 | 11.948679 | 8.120673 |  | H | 17.96869 | 11.558896 | 8.565423 |
| C | 15.613832 | 13.555823 | 9.441048 |  | C | 16.076301 | 14.199831 | 9.566245 |
| H | 15.279319 | 14.23928 | 10.202612 |  | H | 16.002117 | 15.013453 | 10.267361 |
| C | 15.9399 | 13.519149 | 12.870517 |  | C | 16.226515 | 14.276213 | 13.067055 |
| C | 9.782687 | 13.50944 | 4.104737 |  | C | 9.60761 | 13.614232 | 3.842601 |
| C | 20.334733 | 14.003801 | 7.925062 |  | C | 20.614584 | 12.659843 | 8.360715 |
| C | 21.616982 | 14.954141 | 12.745358 |  | C | 22.013049 | 13.439495 | 13.20047 |
| C | 10.855474 | 13.3965 | 0.57568 |  | C | 10.687615 | 14.110743 | 0.349998 |
| C | 21.433564 | 11.431389 | 7.918069 |  | C | 20.657301 | 9.872483 | 8.58078 |
| H | 21.870796 | 10.439512 | 7.904611 |  | H | 20.687049 | 8.791426 | 8.656837 |
| C | 12.147284 | 11.304904 | 8.261205 |  | C | 12.173113 | 12.96952 | 8.256193 |
| C | 11.207576 | 15.743568 | 1.200675 |  | C | 10.135306 | 16.387189 | 1.076075 |
| C | 11.219816 | 13.64952 | 3.990649 |  | C | 10.815268 | 14.373921 | 3.848031 |
| H | 11.641524 | 14.215361 | 3.177778 |  | H | 10.976935 | 15.144568 | 3.113529 |
| C | 11.665901 | 12.395213 | 6.063542 |  | C | 11.677387 | 13.227983 | 5.815756 |
| C | 20.616431 | 15.396654 | 13.591186 |  | C | 21.231935 | 14.2945 | 13.952299 |
| C | 5.969921 | 12.678731 | 4.514655 |  | C | 6.347793 | 11.379418 | 4.056329 |
| C | 15.363527 | 11.475822 | 4.495979 |  | C | 15.580414 | 13.607747 | 4.725612 |
| H | 15.911986 | 12.378498 | 4.73926 |  | H | 15.969141 | 14.403122 | 5.352016 |
| C | 16.583681 | 12.151344 | 12.991171 |  | C | 16.328897 | 12.786083 | 13.330041 |
| H | 17.472142 | 12.143311 | 12.357911 |  | H | 17.160749 | 12.400399 | 12.738905 |
| C | 12.083079 | 13.132627 | 4.888602 |  | C | 11.797609 | 14.179533 | 4.7925 |
| H | 13.139627 | 13.314393 | 4.746501 |  | H | 12.684127 | 14.800949 | 4.748615 |
| C | 21.634781 | 12.278673 | 6.841344 |  | C | 21.185245 | 10.490968 | 7.460067 |
| H | 22.223489 | 11.945576 | 5.993505 |  | H | 21.620406 | 9.893541 | 6.666253 |
| C | 6.608723 | 14.852139 | 5.457432 |  | C | 6.15122 | 13.580796 | 5.118943 |
| C | 20.47482 | 10.879143 | 10.178889 |  | C | 19.520161 | 9.906252 | 10.825253 |
| H | 19.825356 | 11.375999 | 10.902123 |  | H | 19.111259 | 10.669247 | 11.489961 |
| C | 6.496937 | 15.880449 | -0.292611 |  | C | 5.725914 | 14.813727 | -0.590746 |
| C | 10.017886 | 12.132203 | 0.564221 |  | C | 10.3537 | 12.636377 | 0.227237 |
| H | 9.141403 | 12.306184 | 1.190142 |  | H | 9.473197 | 12.441156 | 0.840849 |
| C | 15.936225 | 15.835402 | 12.059309 |  | C | 17.106702 | 16.355784 | 12.110595 |
| C | 11.212799 | 11.945709 | 9.08499 |  | C | 11.246147 | 13.906196 | 8.720405 |
| H | 10.794482 | 12.89496 | 8.770753 |  | H | 10.938512 | 14.715106 | 8.066843 |
| C | 9.362748 | 12.759642 | 5.270349 |  | C | 9.501788 | 12.647104 | 4.885977 |
| H | 8.318152 | 12.550978 | 5.425698 |  | H | 8.634773 | 12.012335 | 4.954947 |
| C | 5.44979 | 15.642743 | 0.578758 |  | C | 4.835543 | 14.1405 | 0.221956 |
| C | 21.092545 | 13.551547 | 6.848292 |  | C | 21.166219 | 11.870555 | 7.355451 |
| H | 21.265831 | 14.209781 | 6.00451 |  | H | 21.593835 | 12.344258 | 6.479207 |
| C | 10.228375 | 12.249803 | 6.171839 |  | C | 10.489506 | 12.480726 | 5.82966 |
| H | 9.824089 | 11.661081 | 6.984296 |  | H | 10.34238 | 11.73355 | 6.601932 |
| C | 5.1711 | 12.459003 | 5.63186 |  | C | 5.681934 | 10.824246 | 5.144067 |
| H | 4.603208 | 11.538885 | 5.709479 |  | H | 5.487525 | 9.757837 | 5.163233 |
| C | 14.740886 | 13.778202 | 13.528873 |  | C | 15.185648 | 15.008443 | 13.63169 |
| H | 14.267767 | 12.990766 | 14.104161 |  | H | 14.438269 | 14.499231 | 14.229307 |
| C | 5.090876 | 13.396269 | 6.648094 |  | C | 5.264095 | 11.611596 | 6.203789 |
| H | 4.46558 | 13.204186 | 7.513232 |  | H | 4.749718 | 11.159242 | 7.04475 |
| C | 13.628367 | 9.901125 | 4.974862 |  | C | 14.094426 | 11.756427 | 4.401863 |
| H | 12.821213 | 9.551409 | 5.608103 |  | H | 13.314682 | 11.101426 | 4.772341 |
| C | 12.071988 | 13.423883 | -0.10052 |  | C | 11.832923 | 14.613177 | -0.261622 |
| H | 12.417845 | 12.532721 | -0.611369 |  | H | 12.495684 | 13.937368 | -0.789926 |
| C | 19.783464 | 15.416775 | 7.915526 |  | C | 20.650421 | 14.170937 | 8.236484 |
| H | 19.203883 | 15.555776 | 8.829268 |  | H | 20.102806 | 14.587354 | 9.083016 |
| C | 12.704868 | 10.109807 | 8.733734 |  | C | 12.534821 | 11.92398 | 9.108062 |
| H | 13.449319 | 9.602327 | 8.131146 |  | H | 13.240154 | 11.178605 | 8.760095 |
| C | 10.833845 | 11.405883 | 10.304933 |  | C | 10.720591 | 13.817581 | 10.000779 |
| H | 10.112115 | 11.931459 | 10.92195 |  | H | 10.011339 | 14.563498 | 10.34375 |
| C | 5.800786 | 14.580471 | 6.558125 |  | C | 5.495415 | 12.975555 | 6.187667 |
| H | 5.721674 | 15.311487 | 7.354673 |  | H | 5.154064 | 13.583553 | 7.017782 |
| C | 14.742548 | 16.044509 | 12.741783 |  | C | 16.051787 | 17.042927 | 12.701834 |
| H | 14.270427 | 17.019715 | 12.705425 |  | H | 15.976624 | 18.117077 | 12.575617 |
| C | 12.411639 | 15.720292 | 0.505043 |  | C | 11.287429 | 16.840288 | 0.44203 |
| H | 13.022207 | 16.615221 | 0.464391 |  | H | 11.527142 | 17.897437 | 0.462522 |
| C | 14.145781 | 15.025519 | 13.464968 |  | C | 15.095114 | 16.376924 | 13.449533 |
| H | 13.211992 | 15.206766 | 13.986035 |  | H | 14.277393 | 16.930181 | 13.898603 |
| C | 6.037798 | 11.629899 | 3.419426 |  | C | 6.79394 | 10.486228 | 2.912692 |
| H | 6.749189 | 11.981587 | 2.66979 |  | H | 7.326101 | 11.112952 | 2.194792 |
| C | 10.76467 | 17.017641 | 1.896668 |  | C | 9.226946 | 17.378258 | 1.782038 |
| H | 9.813531 | 16.810567 | 2.390842 |  | H | 8.411578 | 16.811327 | 2.234986 |
| C | 21.796902 | 10.571971 | 10.883496 |  | C | 20.610585 | 9.16109 | 11.596211 |
| H | 22.475957 | 10.01851 | 10.228099 |  | H | 21.023159 | 8.338415 | 11.004608 |
| H | 21.622422 | 9.957999 | 11.771764 |  | H | 20.203055 | 8.733021 | 12.516682 |
| H | 22.307395 | 11.486726 | 11.190357 |  | H | 21.4357 | 9.82528 | 11.8599 |
| C | 12.844446 | 14.571097 | -0.134931 |  | C | 12.134111 | 15.96263 | -0.214001 |
| H | 13.788947 | 14.571755 | -0.668071 |  | H | 13.03066 | 16.334889 | -0.697769 |
| C | 11.382522 | 10.208702 | 10.744061 |  | C | 11.094375 | 12.775332 | 10.838593 |
| H | 11.086338 | 9.785117 | 11.697773 |  | H | 10.676785 | 12.699208 | 11.836834 |
| C | 15.659352 | 10.795399 | 3.324306 |  | C | 16.096185 | 13.419603 | 3.452101 |
| H | 16.446287 | 11.165011 | 2.674717 |  | H | 16.879243 | 14.075552 | 3.086571 |
| C | 16.560693 | 16.971527 | 11.269762 |  | C | 18.140799 | 17.11574 | 11.300024 |
| H | 17.462831 | 16.584967 | 10.791681 |  | H | 18.840506 | 16.38382 | 10.892458 |
| C | 12.324118 | 9.565909 | 9.95048 |  | C | 11.999356 | 11.825528 | 10.383884 |
| H | 12.766518 | 8.631842 | 10.281154 |  | H | 12.288336 | 10.999556 | 11.025057 |
| C | 9.512498 | 11.827688 | -0.846604 |  | C | 9.988352 | 12.285865 | -1.216016 |
| H | 8.968453 | 12.677926 | -1.266521 |  | H | 9.180505 | 12.9225 | -1.586059 |
| H | 8.841791 | 10.963604 | -0.835808 |  | H | 9.664151 | 11.243569 | -1.28912 |
| H | 10.339104 | 11.59701 | -1.524974 |  | H | 10.8439 | 12.416215 | -1.885404 |
| C | 17.048059 | 11.894214 | 14.425437 |  | C | 16.653076 | 12.518338 | 14.800569 |
| H | 17.710362 | 12.689159 | 14.778364 |  | H | 17.552195 | 13.055528 | 15.113195 |
| H | 17.587866 | 10.944949 | 14.489581 |  | H | 16.815514 | 11.449938 | 14.970634 |
| H | 16.200316 | 11.840447 | 15.114727 |  | H | 15.834713 | 12.838031 | 15.452507 |
| C | 7.342475 | 16.176771 | 5.372152 |  | C | 6.34781 | 15.084819 | 5.11256 |
| H | 7.939724 | 16.170121 | 4.459156 |  | H | 6.927959 | 15.341759 | 4.225224 |
| C | 13.9251 | 9.21693 | 3.806468 |  | C | 14.619808 | 11.557046 | 3.133516 |
| H | 13.354155 | 8.332074 | 3.544539 |  | H | 14.248306 | 10.742677 | 2.520689 |
| C | 15.666491 | 11.037876 | 12.488291 |  | C | 15.077557 | 12.033144 | 12.883902 |
| H | 14.767317 | 10.947598 | 13.104476 |  | H | 14.199154 | 12.331221 | 13.463727 |
| H | 16.186393 | 10.076068 | 12.523728 |  | H | 15.213757 | 10.956743 | 13.024655 |
| H | 15.349184 | 11.216139 | 11.458602 |  | H | 14.859042 | 12.215203 | 11.829456 |
| C | 19.766738 | 9.593817 | 9.750772 |  | C | 18.370129 | 8.970149 | 10.454019 |
| H | 18.80258 | 9.802483 | 9.281527 |  | H | 17.568995 | 9.5037 | 9.937104 |
| H | 19.586957 | 8.954608 | 10.619919 |  | H | 17.946105 | 8.515268 | 11.353971 |
| H | 20.369619 | 9.020241 | 9.040905 |  | H | 18.707873 | 8.158196 | 9.803463 |
| C | 14.946019 | 9.65724 | 2.97384 |  | C | 15.618766 | 12.391525 | 2.650223 |
| H | 15.176851 | 9.124036 | 2.057795 |  | H | 16.028381 | 12.23656 | 1.657821 |
| C | 10.765718 | 10.942899 | 1.164454 |  | C | 11.472571 | 11.743267 | 0.75792 |
| H | 11.638052 | 10.670118 | 0.563508 |  | H | 12.38523 | 11.843007 | 0.163225 |
| H | 10.110543 | 10.067952 | 1.207719 |  | H | 11.16872 | 10.692984 | 0.719019 |
| H | 11.112853 | 11.159483 | 2.177071 |  | H | 11.719172 | 11.990537 | 1.792641 |
| C | 10.523947 | 18.147442 | 0.894671 |  | C | 8.606861 | 18.36674 | 0.793314 |
| H | 11.454737 | 18.448646 | 0.405023 |  | H | 9.370129 | 19.009367 | 0.344351 |
| H | 10.119141 | 19.027843 | 1.402146 |  | H | 7.887756 | 19.015138 | 1.302344 |
| H | 9.822772 | 17.845618 | 0.114243 |  | H | 8.091673 | 17.849329 | -0.017787 |
| C | 16.983872 | 18.120772 | 12.18558 |  | C | 18.94105 | 18.075936 | 12.181259 |
| H | 16.11633 | 18.593265 | 12.655803 |  | H | 18.304842 | 18.872663 | 12.578358 |
| H | 17.5095 | 18.891094 | 11.613904 |  | H | 19.740645 | 18.548826 | 11.603651 |
| H | 17.642648 | 17.77274 | 12.983481 |  | H | 19.388807 | 17.557023 | 13.03073 |
| C | 20.914359 | 16.445628 | 7.937238 |  | C | 22.087078 | 14.687618 | 8.326172 |
| H | 21.586503 | 16.279373 | 8.783131 |  | H | 22.576491 | 14.340765 | 9.239874 |
| H | 20.509521 | 17.458774 | 8.015815 |  | H | 22.103761 | 15.781458 | 8.322602 |
| H | 21.514871 | 16.399145 | 7.024057 |  | H | 22.687614 | 14.343161 | 7.478988 |
| C | 11.750542 | 17.445782 | 2.983921 |  | C | 9.946787 | 18.11425 | 2.91205 |
| H | 11.898667 | 16.65787 | 3.726 |  | H | 10.352482 | 17.420004 | 3.651543 |
| H | 11.37838 | 18.333569 | 3.503489 |  | H | 9.254295 | 18.786724 | 3.426621 |
| H | 12.727741 | 17.697576 | 2.561707 |  | H | 10.773276 | 18.721944 | 2.532326 |
| C | 4.684959 | 11.461536 | 2.726207 |  | C | 5.59762 | 9.868284 | 2.187374 |
| H | 3.937012 | 11.052519 | 3.411931 |  | H | 5.050722 | 9.18087 | 2.839689 |
| H | 4.771597 | 10.770959 | 1.882424 |  | H | 5.932427 | 9.299335 | 1.31517 |
| H | 4.305028 | 12.415142 | 2.354692 |  | H | 4.89602 | 10.634045 | 1.852175 |
| C | 18.831454 | 15.650825 | 6.743744 |  | C | 19.954316 | 14.663224 | 6.969057 |
| H | 19.350089 | 15.570977 | 5.783767 |  | H | 20.475855 | 14.330003 | 6.067008 |
| H | 18.398727 | 16.653808 | 6.801186 |  | H | 19.932524 | 15.756719 | 6.949555 |
| H | 18.013699 | 14.926604 | 6.748012 |  | H | 18.925019 | 14.300765 | 6.91701 |
| C | 15.639612 | 17.465108 | 10.154047 |  | C | 17.516788 | 17.848645 | 10.112747 |
| H | 15.360917 | 16.655892 | 9.475122 |  | H | 16.976866 | 17.162377 | 9.456169 |
| H | 16.139066 | 18.24149 | 9.56717 |  | H | 18.294456 | 18.339442 | 9.52032 |
| H | 14.719269 | 17.898534 | 10.555955 |  | H | 16.816532 | 18.622799 | 10.439647 |
| C | 6.56042 | 10.291011 | 3.939898 |  | C | 7.771162 | 9.406267 | 3.377039 |
| H | 7.543386 | 10.395917 | 4.404702 |  | H | 8.649942 | 9.839645 | 3.860258 |
| H | 6.650869 | 9.575644 | 3.117435 |  | H | 8.114339 | 8.814358 | 2.523501 |
| H | 5.882697 | 9.855917 | 4.680023 |  | H | 7.302314 | 8.717958 | 4.086268 |
| C | 6.355708 | 17.339498 | 5.259278 |  | C | 5.004618 | 15.807665 | 5.00298 |
| H | 6.88905 | 18.282571 | 5.108518 |  | H | 5.155227 | 16.887531 | 4.913914 |
| H | 5.753298 | 17.44101 | 6.166749 |  | H | 4.384072 | 15.630615 | 5.88637 |
| H | 5.66835 | 17.197073 | 4.421299 |  | H | 4.441034 | 15.467663 | 4.130355 |
| C | 8.311403 | 16.371343 | 6.537085 |  | C | 7.146776 | 15.565599 | 6.3225 |
| H | 7.782885 | 16.432534 | 7.493024 |  | H | 6.60847 | 15.38555 | 7.257676 |
| H | 8.872624 | 17.301817 | 6.411861 |  | H | 7.331106 | 16.641538 | 6.251902 |
| H | 9.026591 | 15.547958 | 6.59769 |  | H | 8.111888 | 15.057699 | 6.385427 |
| C | 21.304104 | 14.33726 | 11.539062 |  | C | 21.52816 | 12.896754 | 12.013503 |
| C | 19.277714 | 15.219509 | 13.259739 |  | C | 19.939257 | 14.614368 | 13.546031 |
| C | 7.799782 | 15.540608 | 0.054701 |  | C | 7.053512 | 14.976566 | -0.203969 |
| C | 5.681099 | 15.073454 | 1.826106 |  | C | 5.245413 | 13.629693 | 1.450681 |
| F | 22.293814 | 13.935712 | 10.759652 |  | F | 22.316677 | 12.089315 | 11.320781 |
| F | 22.880615 | 15.129893 | 13.075644 |  | F | 23.236062 | 13.144221 | 13.596115 |
| F | 20.930782 | 15.979801 | 14.730346 |  | F | 21.703301 | 14.803376 | 15.074048 |
| F | 18.355862 | 15.65514 | 14.101195 |  | F | 19.225566 | 15.435091 | 14.301426 |
| F | 8.767528 | 15.78213 | -0.812836 |  | F | 7.873763 | 15.620618 | -1.019948 |
| F | 6.260081 | 16.421248 | -1.470766 |  | F | 5.326399 | 15.2924 | -1.752919 |
| F | 4.221215 | 15.971216 | 0.2331 |  | F | 3.580506 | 13.991982 | -0.155313 |
| F | 4.6504 | 14.870417 | 2.629074 |  | F | 4.355099 | 12.99728 | 2.199646 |

| **2d** (UBHandHLYP/def2-SVP)  Open-shell singlet  *E* = -3688.207415 Hartree | | | |  | **2d** (UBHandHLYP/def2-SVP)  Closed-shell singlet  *E* = -3688.199522 Hartree | | | |
| --- | --- | --- | --- | --- | --- | --- | --- | --- |
| Atom | X | Y | Z |  | Atom | X | Y | Z |
| N | 19.560163 | 12.990523 | 10.412892 |  | N | 19.362165 | 13.574044 | 10.111532 |
| N | 18.177157 | 14.124846 | 11.759918 |  | N | 17.761735 | 14.304231 | 11.490262 |
| N | 8.762292 | 14.370893 | 1.616285 |  | N | 9.211962 | 14.537449 | 1.878703 |
| N | 7.233106 | 13.568438 | 3.040887 |  | N | 7.510722 | 14.158156 | 3.277061 |
| N | 19.756349 | 14.530785 | 13.535283 |  | N | 19.220418 | 15.161114 | 13.210797 |
| N | 7.26064 | 14.815555 | -0.215627 |  | N | 7.906686 | 15.441953 | 0.062148 |
| N | 5.317207 | 13.833711 | 1.602528 |  | N | 5.756003 | 14.981186 | 1.840449 |
| N | 21.517477 | 13.124132 | 11.812879 |  | N | 21.245707 | 14.258204 | 11.45535 |
| C | 7.546254 | 14.412182 | 0.985692 |  | C | 8.059463 | 14.938141 | 1.247897 |
| C | 18.240067 | 13.461775 | 10.522063 |  | C | 17.969709 | 13.665674 | 10.258889 |
| C | 20.27065 | 13.351093 | 11.52842 |  | C | 19.979158 | 14.130629 | 11.205782 |
| C | 20.475867 | 11.059574 | 9.237987 |  | C | 20.754782 | 11.980145 | 8.897876 |
| C | 20.216831 | 12.436799 | 9.268818 |  | C | 20.11753 | 13.227533 | 8.945683 |
| C | 6.582568 | 13.913291 | 1.884446 |  | C | 6.989588 | 14.700912 | 2.127818 |
| C | 19.398735 | 14.060077 | 12.378725 |  | C | 18.97259 | 14.588159 | 12.073432 |
| C | 14.037538 | 12.883932 | 6.62101 |  | C | 13.991327 | 11.810205 | 6.644389 |
| C | 8.606297 | 13.841065 | 2.909195 |  | C | 8.903812 | 14.040934 | 3.154264 |
| C | 6.49911 | 13.226514 | 4.220523 |  | C | 6.698393 | 14.002573 | 4.445415 |
| C | 17.199521 | 13.307512 | 9.582681 |  | C | 17.007325 | 13.215168 | 9.378028 |
| C | 15.081454 | 13.015917 | 7.63918 |  | C | 14.970883 | 12.257244 | 7.504455 |
| C | 12.689181 | 13.001686 | 6.890855 |  | C | 12.552209 | 11.940182 | 6.978822 |
| C | 17.020571 | 14.593076 | 12.460205 |  | C | 16.540948 | 14.465175 | 12.22101 |
| C | 9.971901 | 14.638238 | 0.90046 |  | C | 10.451022 | 14.469757 | 1.164505 |
| C | 15.004183 | 13.943398 | 8.699973 |  | C | 14.675249 | 13.141069 | 8.613757 |
| H | 14.139557 | 14.589984 | 8.775627 |  | H | 13.660627 | 13.496825 | 8.729043 |
| C | 14.524559 | 12.568175 | 5.247815 |  | C | 14.327266 | 11.089664 | 5.39089 |
| C | 16.26228 | 12.245412 | 7.585538 |  | C | 16.367555 | 11.881354 | 7.415918 |
| H | 16.377675 | 11.510282 | 6.79779 |  | H | 16.669027 | 11.173323 | 6.65581 |
| C | 17.267965 | 12.367266 | 8.511915 |  | C | 17.306234 | 12.321985 | 8.279103 |
| H | 18.120004 | 11.71454 | 8.416213 |  | H | 18.308351 | 11.94542 | 8.155822 |
| C | 16.007288 | 14.091995 | 9.623663 |  | C | 15.611329 | 13.585676 | 9.476423 |
| H | 15.884676 | 14.851654 | 10.378145 |  | H | 15.293835 | 14.275596 | 10.240948 |
| C | 16.203387 | 13.663595 | 13.116402 |  | C | 16.017174 | 13.366977 | 12.915021 |
| C | 9.614171 | 13.624559 | 3.87163 |  | C | 9.784661 | 13.536862 | 4.090258 |
| C | 20.626355 | 13.294424 | 8.239717 |  | C | 20.243572 | 14.169649 | 7.916618 |
| C | 21.904643 | 13.617517 | 13.009505 |  | C | 21.518603 | 14.857654 | 12.63014 |
| H | 22.938088 | 13.453371 | 13.294789 |  | H | 22.565122 | 14.985903 | 12.884291 |
| C | 10.667899 | 13.56863 | 0.323094 |  | C | 10.825126 | 13.258333 | 0.569413 |
| C | 21.136348 | 10.547431 | 8.125487 |  | C | 21.510296 | 11.680603 | 7.768539 |
| H | 21.348846 | 9.485673 | 8.073329 |  | H | 22.013564 | 10.722823 | 7.702266 |
| C | 12.170336 | 12.892468 | 8.28412 |  | C | 12.113635 | 11.400005 | 8.289662 |
| C | 10.392225 | 15.96726 | 0.754045 |  | C | 11.222257 | 15.633292 | 1.039752 |
| C | 10.913353 | 14.208808 | 3.769259 |  | C | 11.221011 | 13.683979 | 3.981857 |
| H | 11.150485 | 14.862843 | 2.946358 |  | H | 11.640948 | 14.247318 | 3.165069 |
| C | 11.671202 | 13.198183 | 5.857039 |  | C | 11.651414 | 12.455415 | 6.071971 |
| C | 21.049479 | 14.29407 | 13.846945 |  | C | 20.531773 | 15.292538 | 13.487845 |
| H | 21.398316 | 14.668757 | 14.803107 |  | H | 20.789488 | 15.766495 | 14.428704 |
| C | 6.052696 | 11.907686 | 4.381889 |  | C | 5.906916 | 12.852753 | 4.570478 |
| C | 15.53519 | 13.327019 | 4.651777 |  | C | 15.34158 | 11.523647 | 4.528082 |
| H | 15.949299 | 14.172368 | 5.190335 |  | H | 15.891959 | 12.425533 | 4.770123 |
| C | 16.510184 | 12.178037 | 13.132158 |  | C | 16.692727 | 12.008791 | 12.927963 |
| H | 17.345354 | 12.000797 | 12.453329 |  | H | 17.540257 | 12.047948 | 12.242392 |
| C | 11.885523 | 13.996348 | 4.713185 |  | C | 12.078145 | 13.181137 | 4.893102 |
| H | 12.839616 | 14.490745 | 4.584458 |  | H | 13.135119 | 13.367613 | 4.761109 |
| C | 21.527576 | 11.371923 | 7.084532 |  | C | 21.629034 | 12.58552 | 6.72758 |
| H | 22.037726 | 10.952917 | 6.223886 |  | H | 22.218838 | 12.331243 | 5.85352 |
| C | 6.201808 | 14.232225 | 5.149255 |  | C | 6.680649 | 15.028304 | 5.398865 |
| C | 20.070752 | 10.130133 | 10.36665 |  | C | 20.649384 | 10.968535 | 10.023742 |
| H | 19.488064 | 10.714842 | 11.080725 |  | H | 19.935433 | 11.359113 | 10.751349 |
| C | 5.94839 | 14.728158 | -0.525076 |  | C | 6.629724 | 15.730896 | -0.253681 |
| H | 5.657639 | 15.053812 | -1.517921 |  | H | 6.451105 | 16.155005 | -1.23577 |
| C | 10.171557 | 12.137667 | 0.406059 |  | C | 9.96861 | 12.008946 | 0.648061 |
| H | 9.33974 | 12.112886 | 1.111328 |  | H | 9.123347 | 12.221105 | 1.304314 |
| C | 16.772669 | 15.971688 | 12.513194 |  | C | 15.940669 | 15.730725 | 12.262622 |
| C | 11.292329 | 13.848406 | 8.801369 |  | C | 11.182787 | 12.06546 | 9.097422 |
| H | 11.007872 | 14.693785 | 8.184467 |  | H | 10.778134 | 13.014747 | 8.765805 |
| C | 9.405742 | 12.816045 | 5.028969 |  | C | 9.353285 | 12.804108 | 5.261994 |
| H | 8.463639 | 12.315049 | 5.179192 |  | H | 8.306039 | 12.596197 | 5.406033 |
| C | 5.005483 | 14.25827 | 0.358313 |  | C | 5.582501 | 15.511203 | 0.614438 |
| H | 3.96016 | 14.210689 | 0.073084 |  | H | 4.567125 | 15.761654 | 0.327162 |
| C | 21.276561 | 12.731321 | 7.145143 |  | C | 21.002857 | 13.81698 | 6.804422 |
| H | 21.599308 | 13.369505 | 6.330483 |  | H | 21.112501 | 14.522319 | 5.988468 |
| C | 10.384748 | 12.630244 | 5.973172 |  | C | 10.213727 | 12.30846 | 6.175625 |
| H | 10.159981 | 12.001763 | 6.826839 |  | H | 9.807354 | 11.728265 | 6.993137 |
| C | 5.314272 | 11.60694 | 5.522361 |  | C | 5.107901 | 12.735681 | 5.70346 |
| H | 4.956095 | 10.595154 | 5.674469 |  | H | 4.487051 | 11.856105 | 5.829718 |
| C | 15.093829 | 14.146346 | 13.804592 |  | C | 14.841442 | 13.557349 | 13.63536 |
| H | 14.439457 | 13.449828 | 14.316145 |  | H | 14.407073 | 12.723847 | 14.175214 |
| C | 5.028896 | 12.579063 | 6.465729 |  | C | 5.093581 | 13.724189 | 6.672728 |
| H | 4.455008 | 12.323808 | 7.350071 |  | H | 4.467522 | 13.611918 | 7.551329 |
| C | 14.019058 | 11.475284 | 4.540665 |  | C | 13.603593 | 9.952419 | 5.010744 |
| H | 13.242886 | 10.868255 | 4.992083 |  | H | 12.796064 | 9.605451 | 5.645062 |
| C | 11.83175 | 13.857642 | -0.383667 |  | C | 12.024764 | 13.22677 | -0.136039 |
| H | 12.395215 | 13.050245 | -0.83681 |  | H | 12.346102 | 12.300996 | -0.599138 |
| C | 20.414754 | 14.795565 | 8.296117 |  | C | 19.609043 | 15.546082 | 7.984218 |
| H | 19.823305 | 15.017809 | 9.185221 |  | H | 19.026852 | 15.603499 | 8.904774 |
| C | 12.508888 | 11.805216 | 9.092454 |  | C | 12.65291 | 10.204722 | 8.781682 |
| H | 13.1795 | 11.047506 | 8.704008 |  | H | 13.393433 | 9.678337 | 8.19053 |
| C | 10.788421 | 13.73422 | 10.088491 |  | C | 10.790576 | 11.549176 | 10.323284 |
| H | 10.117213 | 14.49499 | 10.472844 |  | H | 10.072656 | 12.093147 | 10.928527 |
| C | 5.467429 | 13.877859 | 6.277544 |  | C | 5.870216 | 14.858891 | 6.517977 |
| H | 5.226749 | 14.6337 | 7.016605 |  | H | 5.842004 | 15.632399 | 7.276951 |
| C | 15.65586 | 16.403714 | 13.221802 |  | C | 14.772361 | 15.870336 | 13.005191 |
| H | 15.437693 | 17.464014 | 13.279517 |  | H | 14.284454 | 16.837046 | 13.05454 |
| C | 11.557843 | 16.203785 | 0.03175 |  | C | 12.407394 | 15.550949 | 0.315641 |
| H | 11.908456 | 17.221453 | -0.096728 |  | H | 13.026479 | 16.43368 | 0.202884 |
| C | 14.818895 | 15.501375 | 13.85568 |  | C | 14.222555 | 14.794043 | 13.68026 |
| H | 13.950458 | 15.857774 | 14.399102 |  | H | 13.30791 | 14.921323 | 14.249174 |
| C | 6.340422 | 10.821494 | 3.362543 |  | C | 5.895892 | 11.755509 | 3.522845 |
| H | 7.014768 | 11.242815 | 2.614754 |  | H | 6.657595 | 12.001774 | 2.780771 |
| C | 9.618429 | 17.133487 | 1.33952 |  | C | 10.808316 | 16.955104 | 1.658859 |
| H | 8.81682 | 16.72043 | 1.954607 |  | H | 9.913757 | 16.773057 | 2.257379 |
| C | 21.30048 | 9.601131 | 11.10555 |  | C | 21.990514 | 10.806262 | 10.740004 |
| H | 21.913401 | 8.972095 | 10.452474 |  | H | 22.74489 | 10.378246 | 10.072624 |
| H | 20.999153 | 8.993365 | 11.964107 |  | H | 21.887357 | 10.134705 | 11.597661 |
| H | 21.920454 | 10.424937 | 11.464055 |  | H | 22.359937 | 11.769853 | 11.095814 |
| C | 12.275987 | 15.160275 | -0.526508 |  | C | 12.810117 | 14.358865 | -0.261633 |
| H | 13.184408 | 15.364608 | -1.082889 |  | H | 13.740476 | 14.313445 | -0.817368 |
| C | 11.138204 | 12.649514 | 10.881574 |  | C | 11.321656 | 10.351756 | 10.782818 |
| H | 10.739659 | 12.555473 | 11.886017 |  | H | 11.015536 | 9.946917 | 11.741485 |
| C | 16.007852 | 13.018914 | 3.384779 |  | C | 15.636571 | 10.839706 | 3.358248 |
| H | 16.785114 | 13.629359 | 2.937313 |  | H | 16.42414 | 11.206207 | 2.707827 |
| C | 17.678486 | 16.986643 | 11.841048 |  | C | 16.519104 | 16.93086 | 11.536634 |
| H | 18.416969 | 16.43245 | 11.258784 |  | H | 17.368633 | 16.581499 | 10.9469 |
| C | 11.996546 | 11.6824 | 10.375497 |  | C | 12.258438 | 9.684192 | 10.004295 |
| H | 12.267449 | 10.824927 | 10.982267 |  | H | 12.686469 | 8.749501 | 10.351498 |
| C | 9.632089 | 11.679095 | -0.949892 |  | C | 9.394286 | 11.658564 | -0.725282 |
| H | 8.851864 | 12.352697 | -1.312506 |  | H | 8.831984 | 12.496944 | -1.144246 |
| H | 9.210577 | 10.67207 | -0.875481 |  | H | 8.723153 | 10.797648 | -0.651355 |
| H | 10.426397 | 11.653519 | -1.702123 |  | H | 10.186901 | 11.401286 | -1.434243 |
| C | 16.958922 | 11.735356 | 14.525593 |  | C | 17.250463 | 11.695261 | 14.317066 |
| H | 17.815979 | 12.320494 | 14.867976 |  | H | 17.935054 | 12.477617 | 14.654473 |
| H | 17.244206 | 10.679045 | 14.519479 |  | H | 17.792971 | 10.745191 | 14.306735 |
| H | 16.156192 | 11.859256 | 15.258676 |  | H | 16.449112 | 11.611959 | 15.057232 |
| C | 6.619906 | 15.676577 | 4.948678 |  | C | 7.484868 | 16.305043 | 5.242068 |
| H | 7.248653 | 15.723263 | 4.058625 |  | H | 8.087209 | 16.214221 | 4.337211 |
| C | 14.499268 | 11.157423 | 3.278835 |  | C | 13.900029 | 9.264268 | 3.844599 |
| H | 14.09793 | 10.298304 | 2.751976 |  | H | 13.328334 | 8.379316 | 3.584925 |
| C | 15.340454 | 11.338712 | 12.623303 |  | C | 15.768418 | 10.900129 | 12.427975 |
| H | 14.471532 | 11.413945 | 13.283622 |  | H | 14.911805 | 10.758505 | 13.093138 |
| H | 15.625165 | 10.283513 | 12.575846 |  | H | 16.308899 | 9.950146 | 12.382801 |
| H | 15.030774 | 11.654747 | 11.624694 |  | H | 15.384044 | 11.121179 | 11.429825 |
| C | 19.176137 | 8.989299 | 9.883925 |  | C | 20.108234 | 9.622485 | 9.543523 |
| H | 18.282072 | 9.365431 | 9.380304 |  | H | 19.137398 | 9.730614 | 9.053721 |
| H | 18.852633 | 8.378196 | 10.731566 |  | H | 19.983366 | 8.942525 | 10.391104 |
| H | 19.701189 | 8.32946 | 9.187422 |  | H | 20.78976 | 9.140993 | 8.836453 |
| C | 15.491886 | 11.931529 | 2.692523 |  | C | 14.921561 | 9.70161 | 3.011245 |
| H | 15.865555 | 11.685037 | 1.704499 |  | H | 15.15173 | 9.165761 | 2.096613 |
| C | 11.233144 | 11.180013 | 0.941609 |  | C | 10.722404 | 10.830738 | 1.262153 |
| H | 12.082838 | 11.09276 | 0.258162 |  | H | 11.558657 | 10.513626 | 0.63234 |
| H | 10.809501 | 10.178919 | 1.064229 |  | H | 10.053501 | 9.972508 | 1.374789 |
| H | 11.614272 | 11.510729 | 1.910289 |  | H | 11.122646 | 11.082183 | 2.246804 |
| C | 8.964386 | 17.961721 | 0.232866 |  | C | 10.435823 | 17.972346 | 0.579838 |
| H | 9.718749 | 18.436058 | -0.40256 |  | H | 11.302782 | 18.231055 | -0.035889 |
| H | 8.345714 | 18.755214 | 0.662986 |  | H | 10.065629 | 18.895765 | 1.035471 |
| H | 8.334172 | 17.333714 | -0.39959 |  | H | 9.660299 | 17.574633 | -0.077578 |
| C | 18.440015 | 17.809428 | 12.880951 |  | C | 17.047182 | 17.96725 | 12.528989 |
| H | 17.755607 | 18.41821 | 13.479833 |  | H | 16.235102 | 18.387664 | 13.130157 |
| H | 19.145414 | 18.487366 | 12.390902 |  | H | 17.530077 | 18.794015 | 11.999317 |
| H | 18.996459 | 17.158192 | 13.557459 |  | H | 17.774173 | 17.517942 | 13.208231 |
| C | 21.750008 | 15.525516 | 8.447814 |  | C | 20.677455 | 16.637201 | 8.06 |
| H | 22.294466 | 15.172295 | 9.326998 |  | H | 21.349976 | 16.473573 | 8.905788 |
| H | 21.588428 | 16.602448 | 8.55369 |  | H | 20.212307 | 17.620492 | 8.177152 |
| H | 22.389752 | 15.369547 | 7.574013 |  | H | 21.285518 | 16.664739 | 7.151027 |
| C | 10.480737 | 18.00416 | 2.25224 |  | C | 11.873418 | 17.508331 | 2.60457 |
| H | 10.928875 | 17.419408 | 3.059534 |  | H | 12.121378 | 16.792355 | 3.392067 |
| H | 9.873077 | 18.792717 | 2.705458 |  | H | 11.514843 | 18.424468 | 3.082738 |
| H | 11.289906 | 18.492179 | 1.70146 |  | H | 12.796604 | 17.756933 | 2.073398 |
| C | 5.061228 | 10.400741 | 2.638229 |  | C | 4.550652 | 11.706424 | 2.797738 |
| H | 4.352314 | 9.933318 | 3.328696 |  | H | 3.743299 | 11.426954 | 3.481583 |
| H | 5.287588 | 9.67378 | 1.852358 |  | H | 4.576165 | 10.96453 | 1.993943 |
| H | 4.573138 | 11.264162 | 2.18241 |  | H | 4.30721 | 12.678742 | 2.365202 |
| C | 19.623788 | 15.312569 | 7.095972 |  | C | 18.63767 | 15.78701 | 6.829944 |
| H | 20.174613 | 15.176382 | 6.160672 |  | H | 19.153 | 15.790762 | 5.864904 |
| H | 19.424641 | 16.382514 | 7.205838 |  | H | 18.146957 | 16.757954 | 6.943829 |
| H | 18.665597 | 14.796045 | 7.002477 |  | H | 17.863567 | 15.016925 | 6.797436 |
| C | 16.921039 | 17.886057 | 10.865401 |  | C | 15.520782 | 17.550674 | 10.559756 |
| H | 16.397105 | 17.302824 | 10.103986 |  | H | 15.161125 | 16.817153 | 9.833913 |
| H | 17.616621 | 18.557943 | 10.354264 |  | H | 15.991358 | 18.368901 | 10.006882 |
| H | 16.183034 | 18.509644 | 11.377923 |  | H | 14.651249 | 17.964395 | 11.078309 |
| C | 7.051767 | 9.619347 | 3.981997 |  | C | 6.265015 | 10.392878 | 4.107691 |
| H | 7.980075 | 9.913323 | 4.478231 |  | H | 7.241658 | 10.416852 | 4.597418 |
| H | 7.30169 | 8.888508 | 3.207424 |  | H | 6.305127 | 9.640583 | 3.314675 |
| H | 6.423071 | 9.111393 | 4.718932 |  | H | 5.528657 | 10.054171 | 4.842078 |
| C | 5.400839 | 16.561645 | 4.686344 |  | C | 6.562709 | 17.509436 | 5.048989 |
| H | 5.711231 | 17.589183 | 4.474326 |  | H | 7.148987 | 18.415093 | 4.867165 |
| H | 4.734371 | 16.587904 | 5.553608 |  | H | 5.946364 | 17.686153 | 5.935438 |
| H | 4.824201 | 16.194475 | 3.833791 |  | H | 5.890697 | 17.360035 | 4.200173 |
| C | 7.458466 | 16.201753 | 6.112274 |  | C | 8.456095 | 16.518575 | 6.401702 |
| H | 6.882968 | 16.23549 | 7.041994 |  | H | 7.927048 | 16.667311 | 7.347694 |
| H | 7.802328 | 17.218815 | 5.90288 |  | H | 9.06796 | 17.407784 | 6.224668 |
| H | 8.3365 | 15.57372 | 6.280753 |  | H | 9.125408 | 15.663281 | 6.518952 |

| **2d** (UBHandHLYP/def2-SVP)  Open-shell triplet  *E* = -3688.204036 Hartree | | | |  | **2a**^·+^ (UBHandHLYP/def2-SVP)  Open-shell doublet  *E* = -3317.085807 Hartree | | | |
| --- | --- | --- | --- | --- | --- | --- | --- | --- |
| Atom | X | Y | Z |  | Atom | X | Y | Z |
| N | 19.52176 | 12.87134 | 10.48533 |  | N | -7.078517 | 0.916096 | 0.071797 |
| N | 18.21225 | 14.18539 | 11.73966 |  | N | -6.785695 | -1.154988 | 0.637735 |
| N | 8.710756 | 14.43866 | 1.636235 |  | N | 7.078534 | -0.915934 | 0.071734 |
| N | 7.251355 | 13.43245 | 3.004867 |  | N | 6.785624 | 1.155109 | 0.637846 |
| N | 19.7962 | 14.58265 | 13.5124 |  | C | 0.457358 | -0.5353 | -0.715225 |
| N | 7.167236 | 14.9089 | -0.1543 |  | C | 1.86901 | -0.37025 | -0.492776 |
| N | 5.30767 | 13.67346 | 1.600188 |  | C | -6.906302 | 2.298416 | -0.276156 |
| N | 21.47047 | 12.95064 | 11.90129 |  | C | 2.824997 | -1.275228 | -1.023951 |
| C | 7.48925 | 14.44255 | 1.015183 |  | H | 2.487544 | -2.093035 | -1.647163 |
| C | 18.2395 | 13.44315 | 10.54686 |  | C | 2.416023 | 0.682402 | 0.287828 |
| C | 20.24562 | 13.2489 | 11.58595 |  | H | 1.750479 | 1.39187 | 0.758891 |
| C | 20.30625 | 10.81232 | 9.439181 |  | C | -6.496498 | 3.196323 | 0.718474 |
| C | 20.14417 | 12.20366 | 9.383867 |  | C | -0.006068 | -1.913066 | -1.041492 |
| C | 6.568419 | 13.8167 | 1.880603 |  | C | -8.14916 | -0.887951 | 0.751127 |
| C | 19.41852 | 14.07011 | 12.37957 |  | C | -1.869051 | 0.369849 | -0.492985 |
| C | 14.05512 | 12.96348 | 6.594151 |  | C | 4.71161 | -0.090046 | -0.028246 |
| C | 8.60033 | 13.80789 | 2.886693 |  | C | 3.759839 | 0.815122 | 0.509259 |
| C | 6.562125 | 12.96578 | 4.168386 |  | H | 4.076157 | 1.629366 | 1.139047 |
| C | 17.19583 | 13.31211 | 9.594503 |  | C | -0.457354 | 0.53482 | -0.715373 |
| C | 15.10119 | 13.06881 | 7.641226 |  | C | -2.41613 | -0.682518 | 0.287934 |
| C | 12.72766 | 13.06308 | 6.85702 |  | H | -1.750639 | -1.391865 | 0.759249 |
| C | 17.07982 | 14.76779 | 12.39166 |  | C | -6.124733 | -0.043666 | 0.214552 |
| C | 9.901202 | 14.81143 | 0.93546 |  | C | 6.906412 | -2.298251 | -0.276243 |
| C | 15.09349 | 14.08305 | 8.608752 |  | C | -8.329305 | 0.403851 | 0.412253 |
| H | 14.28711 | 14.80645 | 8.612211 |  | C | 7.192837 | -2.705557 | -1.589334 |
| C | 14.57651 | 12.73758 | 5.215598 |  | C | 6.12467 | 0.043752 | 0.214658 |
| C | 16.18946 | 12.18449 | 7.671808 |  | C | -6.239956 | -2.031576 | 3.379924 |
| H | 16.24406 | 11.3851 | 6.941113 |  | H | -6.746767 | -1.121318 | 3.051912 |
| C | 17.1907 | 12.28388 | 8.612328 |  | C | -6.263088 | -2.476459 | 0.845489 |
| H | 17.97939 | 11.54946 | 8.590442 |  | C | 4.171032 | -1.142097 | -0.810167 |
| C | 16.09457 | 14.21033 | 9.546121 |  | H | 4.816627 | -1.865196 | -1.278869 |
| H | 16.03436 | 15.03363 | 10.23916 |  | C | -4.711631 | 0.089998 | -0.028425 |
| C | 16.1909 | 13.93166 | 13.07909 |  | C | -3.759975 | -0.815084 | 0.509362 |
| C | 9.631718 | 13.60427 | 3.83996 |  | H | -4.076394 | -1.629114 | 1.139381 |
| C | 20.61746 | 12.9657 | 8.307893 |  | C | -6.018023 | -2.910415 | 2.158459 |
| C | 21.8788 | 13.48727 | 13.07316 |  | C | -7.192919 | 2.705808 | -1.589179 |
| H | 22.89458 | 13.26742 | 13.38336 |  | C | 6.263031 | 2.476582 | 0.84557 |
| C | 10.66084 | 13.81333 | 0.311597 |  | C | -2.824946 | 1.274712 | -1.02449 |
| C | 20.93753 | 10.18875 | 8.367281 |  | H | -2.487409 | 2.092301 | -1.647942 |
| H | 21.07688 | 9.113796 | 8.382387 |  | C | -9.135445 | -1.925495 | 1.14495 |
| C | 12.18767 | 13.01751 | 8.246189 |  | H | -9.084402 | -2.791834 | 0.481288 |
| C | 10.24124 | 16.16878 | 0.850849 |  | H | -10.14437 | -1.518298 | 1.092981 |
| C | 10.8468 | 14.34192 | 3.814327 |  | H | -8.967132 | -2.28504 | 2.161485 |
| H | 11.01024 | 15.09329 | 3.058855 |  | C | 6.017982 | 2.910565 | 2.158532 |
| C | 11.69805 | 13.22541 | 5.800946 |  | C | -9.560742 | 1.233211 | 0.399801 |
| C | 21.06631 | 14.27173 | 13.85614 |  | H | -9.448834 | 2.116982 | 1.03221 |
| H | 21.43154 | 14.67669 | 14.79367 |  | H | -10.40351 | 0.65359 | 0.774135 |
| C | 6.188447 | 11.61658 | 4.238756 |  | H | -9.808632 | 1.581923 | -0.604252 |
| C | 15.59897 | 13.53894 | 4.701622 |  | C | 8.14912 | 0.888137 | 0.751106 |
| H | 15.99368 | 14.34752 | 5.30708 |  | C | -6.058174 | -3.299293 | -0.270016 |
| C | 16.39969 | 12.43301 | 13.18741 |  | C | 8.329318 | -0.403624 | 0.412135 |
| H | 17.25349 | 12.16673 | 12.56306 |  | C | -4.171006 | 1.141738 | -0.810694 |
| C | 11.83279 | 14.15128 | 4.757299 |  | H | -4.816553 | 1.864716 | -1.279644 |
| H | 12.72996 | 14.75542 | 4.696518 |  | C | 6.496853 | -3.19625 | 0.718407 |
| C | 21.39198 | 10.91873 | 7.282347 |  | C | -0.771676 | -2.155857 | -2.184993 |
| H | 21.87812 | 10.41346 | 6.454756 |  | H | -1.038549 | -1.322576 | -2.825108 |
| C | 6.240524 | 13.88068 | 5.17942 |  | C | 0.006118 | 1.912526 | -1.041788 |
| C | 19.8298 | 9.984093 | 10.61777 |  | C | -0.331771 | 3.007026 | -0.239177 |
| H | 19.27729 | 10.64853 | 11.28464 |  | H | -0.924636 | 2.843494 | 0.654309 |
| C | 5.859459 | 14.75632 | -0.46205 |  | C | 0.084298 | 4.290109 | -0.561393 |
| H | 5.538237 | 15.13068 | -1.42798 |  | H | -0.185464 | 5.120879 | 0.08228 |
| C | 10.2482 | 12.35353 | 0.3203 |  | C | 0.843061 | 4.512898 | -1.703053 |
| H | 9.416521 | 12.2446 | 1.017527 |  | H | 1.162501 | 5.516909 | -1.960805 |
| C | 16.92251 | 16.16056 | 12.36398 |  | C | 1.18226 | 3.438429 | -2.51496 |
| C | 11.28464 | 13.9864 | 8.690837 |  | H | 1.763989 | 3.601033 | -3.416214 |
| H | 10.99933 | 14.79125 | 8.022259 |  | C | 0.772017 | 2.155116 | -2.185139 |
| C | 9.509718 | 12.66105 | 4.896558 |  | H | 1.039065 | 1.321722 | -2.825033 |
| H | 8.628944 | 12.04476 | 4.976619 |  | C | 0.331619 | -3.007422 | -0.238601 |
| C | 4.958105 | 14.16467 | 0.389941 |  | H | 0.924268 | -2.84373 | 0.654998 |
| H | 3.91555 | 14.06891 | 0.106623 |  | C | 6.239837 | 2.031692 | 3.37998 |
| C | 21.23436 | 12.29314 | 7.256653 |  | H | 6.746726 | 1.121475 | 3.051976 |
| H | 21.60551 | 12.85638 | 6.407953 |  | C | 7.021345 | -4.052189 | -1.894769 |
| C | 10.50014 | 12.49689 | 5.839272 |  | H | 7.223029 | -4.401043 | -2.90077 |
| H | 10.34655 | 11.76979 | 6.629003 |  | C | -6.220903 | 2.773155 | 2.150411 |
| C | 5.501393 | 11.19097 | 5.371608 |  | H | -6.432087 | 1.705344 | 2.233217 |
| H | 5.199825 | 10.15297 | 5.454095 |  | C | -5.522548 | -4.199841 | 2.325637 |
| C | 15.10062 | 14.5229 | 13.71141 |  | H | -5.313376 | -4.565275 | 3.32414 |
| H | 14.39139 | 13.90034 | 14.24477 |  | C | -7.645298 | 1.744447 | -2.677518 |
| C | 5.195511 | 12.07163 | 6.394877 |  | H | -7.847779 | 0.778355 | -2.210081 |
| H | 4.663099 | 11.7194 | 7.271798 |  | C | -6.339715 | 4.531494 | 0.355317 |
| C | 14.10218 | 11.68785 | 4.427035 |  | H | -6.016104 | 5.251238 | 1.098225 |
| H | 13.32028 | 11.04609 | 4.815749 |  | C | 7.644887 | -1.744071 | -2.677698 |
| C | 11.80695 | 14.20324 | -0.37559 |  | H | 7.84733 | -0.777989 | -2.210227 |
| H | 12.41766 | 13.45211 | -0.86298 |  | C | 6.058109 | 3.299411 | -0.269943 |
| C | 20.51085 | 14.47858 | 8.271026 |  | C | 6.593397 | -4.955956 | -0.936767 |
| H | 19.92324 | 14.79486 | 9.133807 |  | H | 6.46288 | -5.999992 | -1.199 |
| C | 12.52079 | 11.97816 | 9.116607 |  | C | 4.917178 | 1.606537 | 4.021793 |
| H | 13.20729 | 11.20871 | 8.783428 |  | H | 4.35208 | 2.474881 | 4.372504 |
| C | 10.75513 | 13.93458 | 9.971452 |  | H | 5.106822 | 0.96422 | 4.886358 |
| H | 10.06532 | 14.70484 | 10.29977 |  | H | 4.28319 | 1.053517 | 3.326433 |
| C | 5.55984 | 13.40278 | 6.295692 |  | C | -7.021347 | 4.052438 | -1.894575 |
| H | 5.302724 | 14.08678 | 7.096304 |  | H | -7.223179 | 4.401361 | -2.900523 |
| C | 15.82145 | 16.70285 | 13.01943 |  | C | 9.135358 | 1.925687 | 1.145032 |
| H | 15.6724 | 17.77659 | 13.01341 |  | H | 9.083845 | 2.792365 | 0.481855 |
| C | 11.391 | 16.5071 | 0.143867 |  | H | 10.144349 | 1.518729 | 1.092413 |
| H | 11.6792 | 17.54882 | 0.061085 |  | H | 8.967418 | 2.284658 | 2.161839 |
| C | 14.91316 | 15.89324 | 13.67992 |  | C | 6.340128 | -4.531416 | 0.355213 |
| H | 14.05793 | 16.33458 | 14.18024 |  | H | 6.016685 | -5.251223 | 1.098135 |
| C | 6.496776 | 10.62831 | 3.129491 |  | C | -7.137437 | -2.705193 | 4.419021 |
| H | 7.125851 | 11.13997 | 2.398803 |  | H | -8.084518 | -3.041463 | 3.990633 |
| C | 9.397414 | 17.25939 | 1.483803 |  | H | -7.363001 | -2.008326 | 5.230541 |
| H | 8.622432 | 16.77152 | 2.077977 |  | H | -6.652323 | -3.577023 | 4.865626 |
| C | 21.01457 | 9.426775 | 11.40755 |  | C | 6.221361 | -2.773147 | 2.150385 |
| H | 21.59291 | 8.719344 | 10.80508 |  | H | 6.432636 | -1.705357 | 2.233237 |
| H | 20.66495 | 8.895943 | 12.29822 |  | C | -5.557115 | -4.578435 | -0.042975 |
| H | 21.6818 | 10.23121 | 11.72231 |  | H | -5.376443 | -5.236384 | -0.884894 |
| C | 12.17211 | 15.53525 | -0.45752 |  | C | -5.287451 | -5.024382 | 1.238201 |
| H | 13.06723 | 15.81917 | -1.00038 |  | H | -4.896149 | -6.02384 | 1.392273 |
| C | 11.10027 | 12.89782 | 10.82824 |  | C | -6.59315 | 4.956117 | -0.936603 |
| H | 10.67953 | 12.85092 | 11.82691 |  | H | -6.462585 | 6.000155 | -1.198805 |
| C | 16.10968 | 13.31723 | 3.431506 |  | C | 9.560804 | -1.23291 | 0.399551 |
| H | 16.89498 | 13.95986 | 3.047776 |  | H | 9.449178 | -2.116461 | 1.032317 |
| C | 17.90988 | 17.07608 | 11.66431 |  | H | 10.403671 | -0.6531 | 0.77338 |
| H | 18.61643 | 16.44421 | 11.12292 |  | H | 9.808354 | -1.581977 | -0.60446 |
| C | 11.98038 | 11.91648 | 10.39277 |  | C | 7.137173 | 2.705306 | 4.419202 |
| H | 12.24692 | 11.09528 | 11.04941 |  | H | 8.084263 | 3.041674 | 3.99091 |
| C | 9.740795 | 11.93304 | -1.06009 |  | H | 7.362717 | 2.008408 | 5.230702 |
| H | 8.924637 | 12.57847 | -1.39378 |  | H | 6.651954 | 3.577073 | 4.865816 |
| H | 9.376575 | 10.90144 | -1.0379 |  | C | 6.357833 | 2.856175 | -1.690715 |
| H | 10.53764 | 11.98945 | -1.80787 |  | H | 6.751084 | 1.838623 | -1.652243 |
| C | 16.75155 | 12.03895 | 14.62251 |  | C | 5.522526 | 4.199999 | 2.325704 |
| H | 17.62759 | 12.58731 | 14.97746 |  | H | 5.313354 | 4.56544 | 3.324205 |
| H | 16.96843 | 10.96826 | 14.68384 |  | C | -1.181827 | -3.43923 | -2.514695 |
| H | 15.92483 | 12.25039 | 15.30736 |  | H | -1.763312 | -3.601997 | -3.416078 |
| C | 6.570312 | 15.35796 | 5.076778 |  | C | -6.357953 | -2.856082 | -1.690784 |
| H | 7.191156 | 15.5025 | 4.191796 |  | H | -6.751377 | -1.838595 | -1.652294 |
| C | 14.62232 | 11.45527 | 3.162142 |  | C | 5.557083 | 4.578566 | -0.042912 |
| H | 14.24451 | 10.62862 | 2.570062 |  | H | 5.376414 | 5.236507 | -0.884839 |
| C | 15.20314 | 11.64335 | 12.66187 |  | C | -0.084373 | -4.290562 | -0.560692 |
| H | 14.30905 | 11.81314 | 13.26896 |  | H | 0.185228 | -5.121219 | 0.083195 |
| H | 15.41734 | 10.57075 | 12.68623 |  | C | 4.753257 | -2.975044 | 2.526352 |
| H | 14.96681 | 11.9219 | 11.63257 |  | H | 4.089554 | -2.440615 | 1.843533 |
| C | 18.86961 | 8.873115 | 10.19494 |  | H | 4.568224 | -2.608095 | 3.539804 |
| H | 18.00641 | 9.271146 | 9.655418 |  | H | 4.479887 | -4.033911 | 2.504266 |
| H | 18.49871 | 8.339501 | 11.07486 |  | C | 6.553162 | -1.518718 | -3.726359 |
| H | 19.35851 | 8.137772 | 9.549532 |  | H | 6.295269 | -2.452313 | -4.234473 |
| C | 15.62386 | 12.27287 | 2.656154 |  | H | 6.900652 | -0.813631 | -4.486646 |
| H | 16.02888 | 12.09249 | 1.666225 |  | H | 5.639832 | -1.113794 | -3.286685 |
| C | 11.36169 | 11.43344 | 0.814632 |  | C | -4.917352 | -1.606531 | 4.021929 |
| H | 12.21912 | 11.43248 | 0.135316 |  | H | -4.352333 | -2.47493 | 4.372627 |
| H | 10.99772 | 10.40401 | 0.881902 |  | H | -5.107077 | -0.96429 | 4.886532 |
| H | 11.71675 | 11.73565 | 1.802317 |  | H | -4.283255 | -1.053465 | 3.326707 |
| C | 8.694148 | 18.0933 | 0.412053 |  | C | -6.553826 | 1.519015 | -3.726423 |
| H | 9.41821 | 18.64127 | -0.19896 |  | H | -6.295848 | 2.452619 | -4.234474 |
| H | 8.027116 | 18.82709 | 0.874657 |  | H | -6.901612 | 0.814093 | -4.486728 |
| H | 8.10472 | 17.45527 | -0.24877 |  | H | -5.640486 | 1.113849 | -3.286992 |
| C | 18.71039 | 17.88964 | 12.68215 |  | C | 8.937709 | -2.205405 | -3.35177 |
| H | 18.06065 | 18.57325 | 13.23736 |  | H | 9.733374 | -2.399755 | -2.628636 |
| H | 19.47236 | 18.49136 | 12.17746 |  | H | 9.293628 | -1.440941 | -4.047438 |
| H | 19.20527 | 17.23217 | 13.39917 |  | H | 8.786768 | -3.122353 | -3.927313 |
| C | 21.89166 | 15.12182 | 8.40546 |  | C | -0.842839 | -4.513554 | -1.70251 |
| H | 22.39665 | 14.78356 | 9.313579 |  | H | -1.162204 | -5.517611 | -1.96017 |
| H | 21.80455 | 16.21171 | 8.446489 |  | C | -7.436226 | -3.729624 | -2.332855 |
| H | 22.53178 | 14.87115 | 7.554288 |  | H | -7.102326 | -4.76496 | -2.442762 |
| C | 10.20317 | 18.14262 | 2.435271 |  | H | -7.68291 | -3.356428 | -3.330588 |
| H | 10.68564 | 17.55465 | 3.220122 |  | H | -8.355292 | -3.739605 | -1.740893 |
| H | 9.546974 | 18.87242 | 2.918262 |  | C | 5.287443 | 5.024536 | 1.238262 |
| H | 10.98134 | 18.70286 | 1.909039 |  | H | 4.896161 | 6.024004 | 1.392326 |
| C | 5.216867 | 10.20101 | 2.410284 |  | C | -8.938188 | 2.206021 | -3.351305 |
| H | 4.552831 | 9.64804 | 3.081948 |  | H | -9.733646 | 2.400553 | -2.627993 |
| H | 5.452514 | 9.548009 | 1.564446 |  | H | -9.294416 | 1.441619 | -4.046882 |
| H | 4.675716 | 11.07197 | 2.036273 |  | H | -8.787187 | 3.122931 | -3.926893 |
| C | 19.77637 | 14.9753 | 7.027277 |  | C | 7.146419 | -3.490081 | 3.134172 |
| H | 20.32952 | 14.74402 | 6.112296 |  | H | 6.96873 | -4.568871 | 3.141444 |
| H | 19.65213 | 16.06128 | 7.069103 |  | H | 6.977864 | -3.122827 | 4.150184 |
| H | 18.7855 | 14.52204 | 6.946344 |  | H | 8.199664 | -3.329938 | 2.889039 |
| C | 17.2362 | 17.98242 | 10.63505 |  | C | -7.145995 | 3.489962 | 3.13426 |
| H | 16.68256 | 17.4051 | 9.89027 |  | H | -6.968446 | 4.568775 | 3.141517 |
| H | 17.98727 | 18.57774 | 10.10779 |  | H | -6.977333 | 3.122726 | 4.150261 |
| H | 16.53837 | 18.68096 | 11.10537 |  | H | -8.199232 | 3.32968 | 2.889186 |
| C | 7.284545 | 9.418869 | 3.630951 |  | C | -5.093572 | -2.819599 | -2.548873 |
| H | 8.21456 | 9.718572 | 4.120814 |  | H | -4.326129 | -2.182377 | -2.105317 |
| H | 7.542585 | 8.763167 | 2.794276 |  | H | -5.325109 | -2.435146 | -3.54628 |
| H | 6.70614 | 8.82474 | 4.344145 |  | H | -4.666808 | -3.819103 | -2.670809 |
| C | 5.297899 | 16.18199 | 4.87377 |  | C | -4.752803 | 2.975152 | 2.526341 |
| H | 5.543914 | 17.23867 | 4.731837 |  | H | -4.089079 | 2.440853 | 1.843443 |
| H | 4.633749 | 16.10847 | 5.740261 |  | H | -4.567702 | 2.608109 | 3.539745 |
| H | 4.74185 | 15.83874 | 3.997883 |  | H | -4.479528 | 4.034046 | 2.504367 |
| C | 7.379535 | 15.85679 | 6.272142 |  | C | 7.436258 | 3.729565 | -2.33274 |
| H | 6.805052 | 15.8015 | 7.201412 |  | H | 7.102534 | 4.764967 | -2.442562 |
| H | 7.664469 | 16.9027 | 6.126 |  | H | 7.682867 | 3.356398 | -3.330503 |
| H | 8.292096 | 15.27051 | 6.403659 |  | H | 8.355331 | 3.739337 | -1.740787 |
|  |  |  |  |  | C | 5.093465 | 2.819907 | -2.54883 |
|  |  |  |  |  | H | 4.325911 | 2.182805 | -2.105291 |
|  |  |  |  |  | H | 5.324967 | 2.435411 | -3.546229 |
|  |  |  |  |  | H | 4.66686 | 3.819477 | -2.67079 |

| **2b**^·+^ (UBHandHLYP/def2-SVP)  Open-shell doublet  *E* = -3623.990668 Hartree | | | | | **2c**^·+^ (UBHandHLYP/def2-SVP)  Open-shell doublet  *E* = -4416.896998 Hartree | | | |
| --- | --- | --- | --- | --- | --- | --- | --- | --- |
| Atom | X | Y | Z |  | Atom | X | Y | Z |
| N | -7.060344 | -0.977824 | 0.002331 |  | N | 7.05518 | 1.046658 | -0.082929 |
| N | -6.787347 | 1.125249 | 0.574717 |  | N | 6.809964 | -1.069879 | 0.480975 |
| N | 6.787333 | -1.125257 | 0.57468 |  | N | -6.809566 | 1.069887 | 0.481943 |
| N | 7.060316 | 0.977816 | 0.002297 |  | N | -7.055252 | -1.046365 | -0.083716 |
| C | 8.144747 | -0.852178 | 0.671431 |  | C | -8.159515 | 0.780422 | 0.566111 |
| C | -6.121621 | 0.004772 | 0.159581 |  | C | 6.126884 | 0.049347 | 0.077508 |
| C | -8.315215 | -0.479801 | 0.324126 |  | C | 8.31247 | 0.559892 | 0.22542 |
| C | -7.158115 | -2.79597 | -1.622493 |  | C | 7.076443 | 2.853624 | -1.725719 |
| C | -6.883695 | -2.366054 | -0.314715 |  | C | 6.837522 | 2.43176 | -0.410052 |
| C | 8.315207 | 0.479773 | 0.323998 |  | C | -8.312416 | -0.559419 | 0.224757 |
| C | -8.144748 | 0.852149 | 0.671562 |  | C | 8.159864 | -0.780243 | 0.565627 |
| C | -0.454207 | -0.533614 | -0.691003 |  | C | 0.449025 | 0.535632 | -0.72177 |
| C | 6.121604 | -0.004777 | 0.159582 |  | C | -6.126345 | -0.049355 | 0.077676 |
| C | 6.883677 | 2.366076 | -0.314646 |  | C | -6.838171 | -2.4314 | -0.411154 |
| C | -4.715194 | -0.116604 | -0.063845 |  | C | 4.719888 | 0.156451 | -0.132646 |
| C | -1.872411 | -0.371664 | -0.491221 |  | C | 1.871675 | 0.383728 | -0.535032 |
| C | 0.454192 | 0.533606 | -0.691016 |  | C | -0.448554 | -0.536315 | -0.721045 |
| C | -6.29576 | 2.459621 | 0.767786 |  | C | 6.306394 | -2.403478 | 0.684577 |
| C | 6.295745 | -2.459631 | 0.767751 |  | C | -6.306222 | 2.403436 | 0.685899 |
| C | -2.432864 | 0.67144 | 0.291646 |  | C | 2.446599 | -0.646638 | 0.252516 |
| H | -1.776998 | 1.378469 | 0.779403 |  | H | 1.801353 | -1.353557 | 0.754341 |
| C | 0.012771 | -1.917248 | -0.989742 |  | C | -0.02863 | 1.918708 | -1.006575 |
| C | -2.810644 | -1.281705 | -1.043696 |  | C | 2.79512 | 1.296939 | -1.104391 |
| H | -2.455527 | -2.090825 | -1.668443 |  | H | 2.426619 | 2.097031 | -1.733051 |
| C | -4.159598 | -1.160783 | -0.848511 |  | C | 4.14664 | 1.189147 | -0.922794 |
| H | -4.798718 | -1.878829 | -1.334626 |  | H | 4.773254 | 1.905941 | -1.42553 |
| C | -3.778961 | 0.793475 | 0.496535 |  | C | 3.794911 | -0.755947 | 0.446592 |
| H | -4.113914 | 1.595017 | 1.133628 |  | H | 4.140073 | -1.545976 | 1.091644 |
| C | -6.126379 | 3.282467 | -0.353468 |  | C | 6.113318 | -3.222814 | -0.433036 |
| C | 4.715165 | 0.1166 | -0.063827 |  | C | -4.719669 | -0.156687 | -0.132228 |
| C | -6.512427 | -3.249453 | 0.707486 |  | C | 6.445857 | 3.307363 | 0.608496 |
| C | -10.64147 | -0.286599 | 0.707611 |  | C | 10.65636 | 0.385871 | 0.588668 |
| H | -11.63424 | -0.720254 | 0.737076 |  | C | -6.113845 | 3.223388 | -0.431399 |
| C | 6.126286 | -3.282432 | -0.353523 |  | C | 6.876197 | 4.200024 | -2.010727 |
| C | -7.023696 | -4.154325 | -1.893028 |  | H | 7.048883 | 4.563803 | -3.016895 |
| H | -7.223463 | -4.520929 | -2.893289 |  | C | 0.029339 | -1.919675 | -1.004194 |
| C | -0.012774 | 1.917234 | -0.989795 |  | C | -6.0808 | 2.841533 | 1.998665 |
| C | 6.074526 | -2.912233 | 2.077979 |  | C | -3.79443 | 0.756904 | 0.445239 |
| C | 3.778938 | -0.793467 | 0.496568 |  | H | -4.139486 | 1.548068 | 1.088927 |
| H | 4.113886 | -1.594998 | 1.133678 |  | C | -1.871064 | -0.38438 | -0.534403 |
| C | 1.872398 | 0.371659 | -0.491221 |  | C | 10.507607 | -0.962613 | 0.900211 |
| C | -10.47384 | 1.06227 | 1.027982 |  | C | -7.076178 | -2.8527 | -1.727161 |
| H | -11.34005 | 1.656965 | 1.293559 |  | C | 0.29937 | 2.985819 | -0.165615 |
| C | 7.158075 | 2.796054 | -1.622406 |  | H | 0.892841 | 2.798081 | 0.722704 |
| C | -0.321985 | -2.992186 | -0.160971 |  | C | 6.435967 | -2.777877 | -1.846962 |
| H | -0.913198 | -2.809835 | 0.729972 |  | H | 6.709184 | -1.721299 | -1.816728 |
| C | -6.402758 | 2.814506 | -1.770353 |  | C | -2.44621 | 0.647455 | 0.251207 |
| H | -6.729882 | 1.774262 | -1.725464 |  | H | -1.801066 | 1.355453 | 0.751657 |
| C | 2.432838 | -0.671427 | 0.291675 |  | C | 6.462666 | 5.085226 | -1.029074 |
| H | 1.776967 | -1.378439 | 0.779448 |  | H | 6.311277 | 6.130612 | -1.274082 |
| C | -6.636351 | -5.045788 | -0.906671 |  | C | -6.447975 | -3.307695 | 0.607381 |
| H | -6.534661 | -6.099751 | -1.140567 |  | C | 7.5396 | 1.913757 | -2.825704 |
| C | 6.512514 | 3.249436 | 0.707629 |  | H | 7.586927 | 0.905049 | -2.408818 |
| C | -7.571468 | -1.847575 | -2.73571 |  | C | -10.50736 | 0.96306 | 0.90047 |
| H | -7.598929 | -0.837008 | -2.323213 |  | C | -6.436955 | 2.778954 | -1.845376 |
| C | 10.473862 | -1.062338 | 1.027672 |  | H | -6.710048 | 1.722344 | -1.815393 |
| H | 11.340088 | -1.657048 | 1.293171 |  | C | 6.081462 | -2.842025 | 1.997278 |
| C | 6.402584 | -2.814413 | -1.770405 |  | C | -0.298309 | -2.985769 | -0.161818 |
| H | 6.729692 | -1.774165 | -1.725489 |  | H | -0.891762 | -2.797005 | 0.726291 |
| C | -6.074468 | 2.91218 | 2.078015 |  | C | -4.146005 | -1.190859 | -0.920516 |
| C | 0.322035 | 2.992205 | -0.161089 |  | H | -4.772397 | -1.908754 | -1.421924 |
| H | 0.913273 | 2.809884 | 0.729845 |  | C | -10.65642 | -0.384999 | 0.587662 |
| C | 4.159583 | 1.160768 | -0.848505 |  | C | 6.254134 | 4.64305 | 0.265119 |
| H | 4.798714 | 1.878811 | -1.334614 |  | H | 5.946746 | 5.350293 | 1.026609 |
| C | 10.641488 | 0.286537 | 0.70731 |  | C | -2.794549 | -1.298809 | -1.101922 |
| H | 11.634266 | 0.720175 | 0.736709 |  | H | -2.426059 | -2.100055 | -1.729125 |
| C | -6.3857 | -4.59644 | 0.377774 |  | C | -6.876493 | -4.199109 | -2.012529 |
| H | -6.09263 | -5.306457 | 1.142397 |  | H | -7.048508 | -4.562391 | -3.018995 |
| C | 2.810629 | 1.281694 | -1.043701 |  | C | 5.640632 | -4.512615 | -0.206499 |
| H | 2.455515 | 2.090811 | -1.668454 |  | H | 5.4749 | -5.174639 | -1.048183 |
| C | 7.023738 | 4.154435 | -1.892851 |  | C | -6.46437 | -5.084939 | -1.030854 |
| H | 7.223496 | 4.521088 | -2.893096 |  | H | -6.313391 | -6.130326 | -1.276121 |
| C | -5.69065 | 4.586523 | -0.133248 |  | C | -0.789805 | 2.188815 | -2.145805 |
| H | -5.54063 | 5.247311 | -0.979078 |  | H | -1.044661 | 1.375683 | -2.816026 |
| C | 6.636494 | 5.045864 | -0.906424 |  | C | -5.641386 | 4.513209 | -0.204523 |
| H | 6.534875 | 6.099849 | -1.140249 |  | H | -5.476221 | 5.175648 | -1.045997 |
| C | 0.77265 | -2.181569 | -2.13147 |  | C | 6.260865 | 2.867078 | 2.048388 |
| H | 1.03469 | -1.362571 | -2.791688 |  | H | 6.415838 | 1.787343 | 2.095731 |
| C | 5.69055 | -4.586489 | -0.133326 |  | C | 0.790356 | -2.191049 | -2.143217 |
| H | 5.540466 | -5.247241 | -0.979172 |  | H | 1.044827 | -1.378723 | -2.814563 |
| C | -6.254286 | -2.799016 | 2.133665 |  | C | 0.132397 | -4.2745 | -0.439276 |
| H | -6.417502 | -1.720965 | 2.182468 |  | H | -0.125717 | -5.085143 | 0.233714 |
| C | -0.772682 | 2.18152 | -2.131512 |  | C | -6.256745 | -4.64337 | 0.2637 |
| H | -1.034767 | 1.362494 | -2.791679 |  | H | -5.950475 | -5.351084 | 1.025208 |
| C | -0.097197 | 4.280974 | -0.455364 |  | C | 5.616499 | -4.141945 | 2.165093 |
| H | 0.167674 | 5.097816 | 0.207582 |  | H | 5.430253 | -4.516925 | 3.16468 |
| C | 6.385869 | 4.596453 | 0.378003 |  | C | -5.616079 | 4.141492 | 2.166879 |
| H | 6.092891 | 5.306442 | 1.142686 |  | H | -5.429494 | 4.516061 | 3.166561 |
| C | -5.64916 | 4.227283 | 2.239059 |  | C | 5.391509 | -4.967369 | 1.075978 |
| H | -5.465443 | 4.609768 | 3.236388 |  | H | 5.02773 | -5.977164 | 1.230076 |
| C | 5.649207 | -4.227337 | 2.238997 |  | C | -7.537769 | -1.912083 | -2.827133 |
| H | 5.465541 | -4.609855 | 3.236322 |  | H | -7.584899 | -0.903577 | -2.409765 |
| C | -5.453809 | 5.055514 | 1.146556 |  | C | -6.327465 | 1.967693 | 3.216634 |
| H | -5.118398 | 6.075942 | 1.295229 |  | H | -6.636608 | 0.980472 | 2.865249 |
| C | 7.571349 | 1.847701 | -2.735688 |  | C | 8.945682 | 2.275569 | -3.30821 |
| H | 7.59878 | 0.837112 | -2.323239 |  | H | 8.95571 | 3.253491 | -3.797503 |
| C | 6.263371 | -2.029965 | 3.301189 |  | H | 9.300107 | 1.539665 | -4.034965 |
| H | 6.563084 | -1.038097 | 2.956519 |  | H | 9.657309 | 2.315223 | -2.481762 |
| C | -8.974106 | -2.164966 | -3.25518 |  | C | -5.391782 | 4.967466 | 1.078042 |
| H | -9.014875 | -3.157098 | -3.713336 |  | H | -5.028192 | 5.977288 | 1.232422 |
| H | -9.26971 | -1.438011 | -4.016404 |  | C | 0.889625 | -4.527798 | -1.575484 |
| H | -9.717919 | -2.13382 | -2.456368 |  | H | 1.220069 | -5.536512 | -1.797915 |
| C | 5.453782 | -5.055525 | 1.146475 |  | C | -0.131074 | 4.2743 | -0.444652 |
| H | 5.118363 | -6.075953 | 1.295131 |  | H | 0.127294 | 5.085734 | 0.227287 |
| C | -0.852102 | 4.526614 | -1.594826 |  | C | 6.328932 | -1.968845 | 3.215579 |
| H | -1.174047 | 5.535165 | -1.830342 |  | H | 6.63789 | -0.981408 | 2.864593 |
| C | 0.097272 | -4.280959 | -0.455196 |  | C | 1.21284 | -3.481071 | -2.428801 |
| H | -0.167557 | -5.097776 | 0.207798 |  | H | 1.791914 | -3.670571 | -3.3262 |
| C | -6.263254 | 2.029862 | 3.301197 |  | C | -7.647841 | 3.542451 | -2.384966 |
| H | -6.562921 | 1.037991 | 2.956493 |  | H | -8.508566 | 3.447988 | -1.717906 |
| C | -1.18456 | 3.471165 | -2.433743 |  | H | -7.933757 | 3.163037 | -3.369859 |
| H | -1.762941 | 3.653356 | -3.333195 |  | H | -7.4295 | 4.608781 | -2.490335 |
| C | 7.539453 | -3.612786 | -2.408816 |  | C | 7.646601 | -3.541297 | -2.387239 |
| H | 8.452311 | -3.558803 | -1.810405 |  | H | 8.507586 | -3.447137 | -1.720468 |
| H | 7.765169 | -3.222003 | -3.404826 |  | H | 7.932178 | -3.161585 | -3.372112 |
| H | 7.275108 | -4.667915 | -2.521329 |  | H | 7.428122 | -4.60757 | -2.492876 |
| C | -7.539641 | 3.612921 | -2.408684 |  | C | -6.264078 | -2.868009 | 2.047593 |
| H | -8.452474 | 3.55893 | -1.810236 |  | H | -6.418131 | -1.788152 | 2.095039 |
| H | -7.765404 | 3.22218 | -3.404699 |  | C | -1.212062 | 3.478559 | -2.432946 |
| H | -7.275285 | 4.668051 | -2.521169 |  | H | -1.791336 | 3.667024 | -3.330433 |
| C | 6.25442 | 2.798946 | 2.133799 |  | C | 5.23314 | -2.898658 | -2.780227 |
| H | 6.417661 | 1.720897 | 2.182567 |  | H | 4.927684 | -3.940639 | -2.90905 |
| C | 1.184551 | -3.47122 | -2.43365 |  | H | 5.482955 | -2.506384 | -3.769896 |
| H | 1.762906 | -3.653441 | -3.333113 |  | H | 4.374189 | -2.342577 | -2.397767 |
| C | -5.141465 | 2.853567 | -2.632581 |  | C | 6.556991 | 1.86714 | -3.996161 |
| H | -4.779574 | 3.877392 | -2.762871 |  | H | 5.555612 | 1.570041 | -3.675573 |
| H | -5.350458 | 2.448741 | -3.626811 |  | H | 6.897108 | 1.145997 | -4.744296 |
| H | -4.333772 | 2.268766 | -2.187166 |  | H | 6.476496 | 2.8383 | -4.491796 |
| C | -6.559811 | -1.840279 | -3.882997 |  | C | -0.888452 | 4.526317 | -1.581038 |
| H | -5.557352 | -1.573762 | -3.54052 |  | H | -1.21876 | 5.534808 | -1.804676 |
| H | -6.858136 | -1.112885 | -4.642875 |  | C | -5.23446 | 2.900196 | -2.779012 |
| H | -6.4991 | -2.817159 | -4.370577 |  | H | -4.929116 | 3.942254 | -2.907518 |
| C | 0.852147 | -4.526635 | -1.59467 |  | H | -5.484583 | 2.508292 | -3.768754 |
| H | 1.174113 | -5.535189 | -1.830145 |  | H | -4.375347 | 2.344015 | -2.397063 |
| C | 5.141253 | -2.853457 | -2.632577 |  | C | -7.464014 | 2.523962 | 4.076905 |
| H | 4.779373 | -3.877283 | -2.7629 |  | H | -7.190026 | 3.486308 | 4.518225 |
| H | 5.350197 | -2.448584 | -3.626797 |  | H | -7.691467 | 1.83787 | 4.897218 |
| H | 4.333569 | -2.268689 | -2.187101 |  | H | -8.373083 | 2.677969 | 3.493124 |
| C | 7.37784 | -2.553176 | 4.208189 |  | C | 7.465999 | -2.525643 | 4.074835 |
| H | 7.130458 | -3.536858 | 4.617141 |  | H | 7.192215 | -3.488219 | 4.51577 |
| H | 7.529676 | -1.874216 | 5.051562 |  | H | 7.693969 | -1.840028 | 4.8954 |
| H | 8.326465 | -2.640792 | 3.674664 |  | H | 8.374716 | -2.679391 | 3.490451 |
| C | -7.377742 | 2.552989 | 4.208225 |  | C | 7.314522 | 3.511027 | 2.950858 |
| H | -7.130417 | 3.536687 | 4.617172 |  | H | 8.325601 | 3.311769 | 2.586493 |
| H | -7.529512 | 1.874016 | 5.051598 |  | H | 7.234984 | 3.123628 | 3.970298 |
| H | -8.326384 | 2.640539 | 3.67472 |  | H | 7.189388 | 4.596268 | 2.998145 |
| C | -7.240654 | -3.442703 | 3.108289 |  | C | -5.061615 | 1.768595 | 4.050557 |
| H | -8.275309 | -3.22925 | 2.828429 |  | H | -4.250913 | 1.332255 | 3.462311 |
| H | -7.078796 | -3.060645 | 4.11998 |  | H | -5.265432 | 1.097954 | 4.889743 |
| H | -7.120628 | -4.52902 | 3.143576 |  | H | -4.703099 | 2.713556 | 4.46738 |
| C | 4.96305 | -1.860512 | 4.088447 |  | C | -8.943603 | -2.272872 | -3.311094 |
| H | 4.162947 | -1.44828 | 3.469576 |  | H | -8.953761 | -3.250574 | -3.800834 |
| H | 5.119107 | -1.180453 | 4.930334 |  | H | -9.29696 | -1.536435 | -4.037837 |
| H | 4.614365 | -2.812841 | 4.49714 |  | H | -9.655987 | -2.31247 | -2.485289 |
| C | 8.973987 | 2.165047 | -3.255189 |  | C | 4.844987 | 3.137233 | 2.554326 |
| H | 9.014786 | 3.15718 | -3.713339 |  | H | 4.625726 | 4.208216 | 2.578894 |
| H | 9.269538 | 1.438087 | -4.016429 |  | H | 4.727091 | 2.755423 | 3.572177 |
| H | 9.717822 | 2.13386 | -2.4564 |  | H | 4.097767 | 2.653967 | 1.920233 |
| C | -4.807044 | -3.055953 | 2.553021 |  | C | 5.063671 | -1.770186 | 4.050514 |
| H | -4.580151 | -4.12579 | 2.568192 |  | H | 4.252517 | -1.333502 | 3.463151 |
| H | -4.62974 | -2.667818 | 3.559868 |  | H | 5.268115 | -1.100016 | 4.889918 |
| H | -4.103479 | -2.571335 | 1.872197 |  | H | 4.705446 | -2.715361 | 4.467086 |
| C | -4.962919 | 1.860438 | 4.088439 |  | C | -6.553977 | -1.865453 | -3.996586 |
| H | -4.162793 | 1.448291 | 3.46954 |  | H | -5.552758 | -1.569144 | -3.674766 |
| H | -5.118934 | 1.180318 | 4.930283 |  | H | -6.892892 | -1.143701 | -4.744687 |
| H | -4.614288 | 2.812761 | 4.497193 |  | H | -6.473574 | -2.836408 | -4.492652 |
| C | 6.559658 | 1.840515 | -3.882946 |  | C | -7.319247 | -3.51131 | 2.94876 |
| H | 5.557199 | 1.574002 | -3.540466 |  | H | -7.240507 | -3.124246 | 3.968393 |
| H | 6.857942 | 1.113171 | -4.642886 |  | H | -7.195068 | -4.596672 | 2.995883 |
| H | 6.498962 | 2.817435 | -4.370448 |  | H | -8.329761 | -3.311122 | 2.583344 |
| C | 7.240788 | 3.442612 | 3.108436 |  | C | -4.848986 | -3.139557 | 2.554968 |
| H | 7.078984 | 3.06046 | 4.1201 |  | H | -4.630685 | -4.21074 | 2.579483 |
| H | 7.120701 | 4.528919 | 3.143827 |  | H | -4.731837 | -2.758122 | 3.573049 |
| H | 8.275446 | 3.229243 | 2.828524 |  | H | -4.10067 | -2.656772 | 1.921799 |
| C | 4.807182 | 3.055857 | 2.553194 |  | C | 9.560287 | 1.165527 | 0.261788 |
| H | 4.580288 | 4.125694 | 2.568377 |  | C | 9.261023 | -1.564137 | 0.878547 |
| H | 4.629907 | 2.667711 | 3.560042 |  | C | -9.260522 | 1.564227 | 0.879574 |
| H | 4.1036 | 2.571245 | 1.872383 |  | C | -9.560404 | -1.164635 | 0.260278 |
| C | -9.563627 | -1.083315 | 0.34775 |  | F | 9.738396 | 2.439784 | -0.017845 |
| C | -9.219832 | 1.656768 | 1.017777 |  | F | 11.85149 | 0.925457 | 0.619723 |
| C | 9.219846 | -1.65682 | 1.017551 |  | F | 11.56746 | -1.673591 | 1.203294 |
| C | 9.56363 | 1.083272 | 0.347539 |  | F | 9.160427 | -2.845124 | 1.166435 |
| H | -9.688427 | -2.12889 | 0.097358 |  | F | -9.159715 | 2.844953 | 1.168784 |
| H | -9.083346 | 2.701236 | 1.266298 |  | F | -11.56703 | 1.674179 | 1.204066 |
| H | 9.083365 | -2.70129 | 1.266062 |  | F | -11.8517 | -0.924412 | 0.617927 |
| H | 9.688424 | 2.128849 | 0.097158 |  | F | -9.738919 | -2.438605 | -0.020658 |

| **2d**^·+^ (UBHandHLYP/def2-SVP)  Open-shell doublet  *E* = -3688.051985 Hartree | | | |
| --- | --- | --- | --- |
| Atom | X | Y | Z |
| N | -7.047815 | -1.012397 | 0.023812 |
| N | -6.80765 | 1.150747 | 0.450097 |
| N | 6.807685 | -1.150759 | 0.449956 |
| N | 7.047824 | 1.01243 | 0.023839 |
| N | -9.152504 | 1.6562 | 0.794477 |
| N | 9.152546 | -1.656228 | 0.794199 |
| N | 9.455285 | 1.109151 | 0.281108 |
| N | -9.455277 | -1.109124 | 0.281098 |
| C | 8.153521 | -0.861948 | 0.534505 |
| C | -6.125143 | 0.000598 | 0.133039 |
| C | -8.304213 | -0.499926 | 0.270388 |
| C | -7.080057 | -3.040348 | -1.337366 |
| C | -6.819887 | -2.426636 | -0.104582 |
| C | 8.304229 | 0.499948 | 0.270349 |
| C | -8.15349 | 0.861934 | 0.534681 |
| C | -0.451837 | -0.53079 | -0.58657 |
| C | 6.125155 | -0.000585 | 0.133002 |
| C | 6.819864 | 2.426674 | -0.104411 |
| C | -4.719874 | -0.119923 | -0.050674 |
| C | -1.878761 | -0.3674 | -0.414116 |
| C | 0.451857 | 0.530764 | -0.586624 |
| C | -6.319856 | 2.502864 | 0.496548 |
| C | 6.31984 | -2.502854 | 0.496373 |
| C | -2.452667 | 0.671333 | 0.362027 |
| H | -1.807307 | 1.374485 | 0.868621 |
| C | 0.014156 | -1.921795 | -0.853384 |
| C | -2.797148 | -1.282214 | -0.987547 |
| H | -2.423389 | -2.088696 | -1.604696 |
| C | -4.150441 | -1.166988 | -0.820628 |
| H | -4.78374 | -1.882436 | -1.319917 |
| C | -3.803109 | 0.793818 | 0.534866 |
| H | -4.160058 | 1.588894 | 1.169494 |
| C | -6.098671 | 3.176007 | -0.711254 |
| C | 4.719892 | 0.119933 | -0.050686 |
| C | -6.399985 | -3.141979 | 1.023768 |
| C | -10.48996 | -0.31132 | 0.554672 |
| H | -11.47428 | -0.766244 | 0.576268 |
| C | 6.098465 | -3.175897 | -0.711454 |
| C | -6.873044 | -4.413338 | -1.425512 |
| H | -7.063796 | -4.922407 | -2.362988 |
| C | -0.014159 | 1.921745 | -0.853562 |
| C | 6.142368 | -3.111163 | 1.746411 |
| C | 3.803121 | -0.793791 | 0.534882 |
| H | 4.160073 | -1.588841 | 1.169543 |
| C | 1.87877 | 0.367399 | -0.414142 |
| C | -10.3417 | 1.049717 | 0.801017 |
| H | -11.21005 | 1.664286 | 1.012169 |
| C | 7.080038 | 3.040548 | -1.337118 |
| C | -0.319553 | -2.973417 | 0.004234 |
| H | -0.909304 | -2.768759 | 0.891298 |
| C | -6.370116 | 2.55249 | -2.067794 |
| H | -6.63632 | 1.504981 | -1.91515 |
| C | 2.452684 | -0.671305 | 0.362043 |
| H | 1.807327 | -1.374433 | 0.868674 |
| C | -6.431404 | -5.14176 | -0.334246 |
| H | -6.275045 | -6.210906 | -0.425723 |
| C | 6.399914 | 3.141868 | 1.02401 |
| C | -7.582392 | -2.277276 | -2.550127 |
| H | -7.592972 | -1.214596 | -2.297124 |
| C | 10.341744 | -1.049734 | 0.800775 |
| H | 11.210094 | -1.664323 | 1.011843 |
| C | 6.369928 | -2.552313 | -2.067957 |
| H | 6.635547 | -1.504659 | -1.915302 |
| C | -6.142286 | 3.111093 | 1.746612 |
| C | 0.319491 | 2.973438 | 0.00399 |
| H | 0.909209 | 2.768861 | 0.891095 |
| C | 4.150455 | 1.166979 | -0.820666 |
| H | 4.783757 | 1.882417 | -1.319969 |
| C | 10.489988 | 0.311324 | 0.55458 |
| H | 11.474299 | 0.766261 | 0.576207 |
| C | -6.201482 | -4.511913 | 0.875902 |
| H | -5.872848 | -5.097365 | 1.726662 |
| C | 2.797163 | 1.282198 | -0.987594 |
| H | 2.423414 | 2.088659 | -1.604775 |
| C | 6.872983 | 4.413543 | -1.4251 |
| H | 7.063739 | 4.922732 | -2.362511 |
| C | -5.644558 | 4.489794 | -0.63815 |
| H | -5.459384 | 5.040732 | -1.55276 |
| C | 6.431293 | 5.141818 | -0.333755 |
| H | 6.2749 | 6.210971 | -0.425105 |
| C | 0.767487 | -2.213205 | -1.992214 |
| H | 1.025656 | -1.411786 | -2.675189 |
| C | 5.64427 | -4.489654 | -0.638393 |
| H | 5.458955 | -5.040514 | -1.553019 |
| C | -6.190526 | -2.50013 | 2.382839 |
| H | -6.349087 | -1.42492 | 2.281844 |
| C | -0.767443 | 2.213049 | -1.992448 |
| H | -1.025567 | 1.41157 | -2.67537 |
| C | -0.101477 | 4.268655 | -0.257769 |
| H | 0.159889 | 5.06846 | 0.426733 |
| C | 6.201363 | 4.511813 | 0.876307 |
| H | 5.872686 | 5.097143 | 1.727134 |
| C | -5.694776 | 4.42827 | 1.760102 |
| H | -5.547081 | 4.931466 | 2.708429 |
| C | 5.694774 | -4.428314 | 1.759859 |
| H | 5.547145 | -4.931565 | 2.708166 |
| C | -5.440534 | 5.109258 | 0.581889 |
| H | -5.090994 | 6.135199 | 0.615554 |
| C | 7.582402 | 2.277654 | -2.54998 |
| H | 7.59304 | 1.214941 | -2.297116 |
| C | 6.433352 | -2.402955 | 3.057665 |
| H | 6.665878 | -1.359719 | 2.83123 |
| C | -9.018299 | -2.679085 | -2.890573 |
| H | -9.071179 | -3.727001 | -3.199453 |
| H | -9.398749 | -2.071218 | -3.71636 |
| H | -9.676535 | -2.546467 | -2.03018 |
| C | 5.440351 | -5.109197 | 0.581627 |
| H | 5.090744 | -6.135116 | 0.615255 |
| C | -0.852762 | 4.542132 | -1.39326 |
| H | -1.176203 | 5.55583 | -1.60268 |
| C | 0.101401 | -4.268663 | -0.257406 |
| H | -0.160014 | -5.06841 | 0.427144 |
| C | -6.433116 | 2.402773 | 3.057839 |
| H | -6.66563 | 1.359548 | 2.831337 |
| C | -1.17962 | 3.509751 | -2.2623 |
| H | -1.754357 | 3.715147 | -3.158881 |
| C | 7.567525 | -3.223977 | -2.741851 |
| H | 8.455236 | -3.184539 | -2.105725 |
| H | 7.801527 | -2.728754 | -3.688489 |
| H | 7.361855 | -4.275747 | -2.959496 |
| C | -7.567178 | 3.224676 | -2.742102 |
| H | -8.455105 | 3.185705 | -2.106247 |
| H | -7.801122 | 2.729475 | -3.688765 |
| H | -7.360961 | 4.276326 | -2.959799 |
| C | 6.190412 | 2.499834 | 2.382985 |
| H | 6.349058 | 1.424648 | 2.281859 |
| C | 1.179655 | -3.509935 | -2.261943 |
| H | 1.754435 | -3.715409 | -3.158479 |
| C | -5.136436 | 2.568363 | -2.968359 |
| H | -4.839394 | 3.589154 | -3.224494 |
| H | -5.345998 | 2.044569 | -3.905066 |
| H | -4.285156 | 2.081746 | -2.486711 |
| C | -6.662816 | -2.439789 | -3.76022 |
| H | -5.639311 | -2.126116 | -3.539438 |
| H | -7.026405 | -1.832262 | -4.593353 |
| H | -6.627683 | -3.476731 | -4.104463 |
| C | 0.852738 | -4.542243 | -1.392838 |
| H | 1.17617 | -5.555963 | -1.602161 |
| C | 5.136538 | -2.568816 | -2.968894 |
| H | 4.840085 | -3.589735 | -3.225192 |
| H | 5.346122 | -2.044872 | -3.905511 |
| H | 4.284867 | -2.082642 | -2.487482 |
| C | 7.661898 | -3.007028 | 3.739765 |
| H | 7.47686 | -4.0437 | 4.035574 |
| H | 7.912909 | -2.444329 | 4.643445 |
| H | 8.525406 | -2.995774 | 3.07238 |
| C | -7.66162 | 3.006749 | 3.740102 |
| H | -7.476582 | 4.043396 | 4.035999 |
| H | -7.912547 | 2.443953 | 4.643745 |
| H | -8.525184 | 2.995548 | 3.072787 |
| C | -7.222164 | -3.009362 | 3.390508 |
| H | -8.241603 | -2.84361 | 3.033383 |
| H | -7.106837 | -2.494471 | 4.348446 |
| H | -7.104629 | -4.08085 | 3.574323 |
| C | 5.227931 | -2.397899 | 3.997232 |
| H | 4.349906 | -1.943389 | 3.531168 |
| H | 5.458565 | -1.828991 | 4.901972 |
| H | 4.954829 | -3.409159 | 4.309818 |
| C | 9.018283 | 2.679575 | -2.890402 |
| H | 9.07111 | 3.727532 | -3.199151 |
| H | 9.398741 | 2.07183 | -3.716277 |
| H | 9.676547 | 2.546873 | -2.030042 |
| C | -4.763703 | -2.691967 | 2.894668 |
| H | -4.539828 | -3.747453 | 3.072446 |
| H | -4.627174 | -2.164403 | 3.842709 |
| H | -4.030682 | -2.306599 | 2.181881 |
| C | -5.227607 | 2.397674 | 3.997294 |
| H | -4.349632 | 1.94317 | 3.531133 |
| H | -5.458167 | 1.828736 | 4.902033 |
| H | -4.954467 | 3.40892 | 4.309889 |
| C | 6.662809 | 2.440283 | -3.760045 |
| H | 5.639321 | 2.126527 | -3.539308 |
| H | 7.026426 | 1.832893 | -4.593266 |
| H | 6.627623 | 3.477272 | -4.104141 |
| C | 7.221939 | 3.008995 | 3.3908 |
| H | 7.106538 | 2.493997 | 4.348672 |
| H | 7.104364 | 4.080459 | 3.57472 |
| H | 8.241418 | 2.843307 | 3.033754 |
| C | 4.763536 | 2.691519 | 2.894732 |
| H | 4.539637 | 3.746964 | 3.072741 |
| H | 4.626931 | 2.163728 | 3.842635 |
| H | 4.030584 | 2.306313 | 2.181789 |

# References

[1] H. Koike, M. Chikamatsu, R. Azumi, J. Tsutsumi, K. Ogawa, W. Yamane, T. Nishiuchi, T. Kubo, T. Hasegawa, K. Kanai, "Stable delocalized singlet biradical hydrocarbon for organic field‐effect transistors," *Adv. Funct. Mater.* 26, (2016): 277–283, <https://doi.org/10.1002/adfm.201503650>.

[2] G. E. Rudebusch, J. L. Zafra, K. Jorner, K. Fukuda, J. L. Marshall, I. Arrechea-Marcos, G. L. Espejo, R. Ponce Ortiz, C. J. Gómez-García, L. N. Zakharov, et al., "Diindeno-fusion of an anthracene as a design strategy for stable organic biradicals," *Nat. Chem.* 8, (2016): 753–759, <https://doi.org/10.1038/nchem.2518>.

[3] Y.-C. Hsieh, C.-F. Wu, Y.-T. Chen, C.-T. Fang, C.-S. Wang, C.-H. Li, L.-Y. Chen, M.-J. Cheng, C.-C. Chueh, P.-T. Chou, et al., "5,14-Diaryldiindeno[2,1-f:1′,2′-j]picene: A new stable [7]helicene with a partial biradical character," *J. Am. Chem. Soc.* 140, (2018): 14357–14366, <https://doi.org/10.1021/jacs.8b08840>.

[4] T. Jousselin-Oba, M. Mamada, J. Marrot, A. Maignan, C. Adachi, A. Yassar, M. Frigoli, "Excellent semiconductors based on tetracenotetracene and pentacenopentacene: from stable closed-shell to singlet open-shell," *J. Am. Chem. Soc.* 141, (2019): 9373–9381, <https://doi.org/10.1021/jacs.9b03488>.

[5] T. Jousselin-Oba, M. Mamada, A. Okazawa, J. Marrot, T. Ishida, C. Adachi, A. Yassar, M. Frigoli, "Modulating the ground state, stability and charge transport in OFETs of biradicaloid hexahydro-diindenopyrene derivatives and a proposed method to estimate the biradical character," *Chem. Sci.* 11, (2020): 12194–12205, <https://doi.org/10.1039/D0SC04583G>.

[6] Z. Lin, L. Chen, Q. Xu, G. Shao, Z. Zeng, D. Wu, J. Xia, "Tuning biradical character to enable high and balanced ambipolar charge transport in a quinoidal *π*-system," *Org. Lett.* 22, (2020): 2553–2558, <https://doi.org/10.1021/acs.orglett.0c00453>.

[7] M. Mamada, R. Nakamura, C. Adachi, "Synthesis, crystal structure and charge transport characteristics of stable *peri*-tetracene analogues," *Chem. Sci.* 12, (2021): 552–558, <https://doi.org/10.1039/D0SC04699J>.

[8] C. Zong, X. Zhu, Z. Xu, L. Zhang, J. Xu, J. Guo, Q. Xiang, Z. Zeng, W. Hu, J. Wu, et al., "Isomeric dibenzoheptazethrenes for air‐stable organic field‐effect transistors," *Angew. Chem. Int. Ed.* 60, (2021): 16230–16236, <https://doi.org/10.1002/anie.202105872>.

[9] S. Mori, S. Moles Quintero, N. Tabaka, R. Kishi, R. González Núñez, A. Harbuzaru, R. Ponce Ortiz, J. Marín‐Beloqui, S. Suzuki, C. Kitamura, et al., "Medium diradical character, small hole and electron reorganization energies and ambipolar transistors in difluorenoheteroles," *Angew. Chem. Int. Ed.* 61, (2022): e202206680, <https://doi.org/10.1002/anie.202206680>.

[10] C. Zong, S. Yang, Y. Sun, L. Zhang, J. Hu, W. Hu, R. Li, Z. Sun, "Isomeric dibenzooctazethrene diradicals for high-performance air-stable organic field-effect transistors," *Chem. Sci.* 13, (2022): 11442–11447, <https://doi.org/10.1039/D2SC03667C>.

[11] P. Wang, Q. Xiang, M. Tian, S. Tao, Z. Xu, Y. Guo, W. Hu, Z. Sun, "Spin‐distribution‐directed regioselective substitution strategy for highly stable olympicenyl radicals," *Angew. Chem. Int. Ed.* 62, (2023): e202313257, <https://doi.org/10.1002/anie.202313257>.

[12] T. Luo, Y. Wang, J. Hao, P. Chen, Y. Hu, B. Chen, J. Zhang, K. Yang, Z. Zeng, "Furan‐extended helical rylenes with fjord edge topology and tunable optoelectronic properties," *Angew. Chem. Int. Ed.* 62, (2023): e202214653, <https://doi.org/10.1002/anie.202214653>.

[13] W. Xiao, L. Tian, X. Wu, P. Dai, S. Xie, Y. Yao, K. Yang, Z. Zeng, "Diverse postfunctionalization of open-shell diradicaloids and impact of conjugated functional substituents on optoelectronic properties," *Org. Lett.* 27, (2025): 3204–3209, <https://doi.org/10.1021/acs.orglett.5c00482>.

[14] L. Zhao, H. Liu, W. Li, Y. Yang, X. He, Z. Zhang, Y. Zhao, Y. Yao, L. Sun, K. Yang, et al., "Stable diradical‐derived conjugated radical polymers," *Angew. Chem. Int. Ed.* 64, (2025): e202507603, <https://doi.org/10.1002/anie.202507603>.
